# Entamoeba histolytica Phagocytosis of Human Erythrocytes Involves PATMK, a Member of the Transmembrane Kinase Family

**DOI:** 10.1371/journal.ppat.0040008

**Published:** 2008-01-18

**Authors:** Douglas R Boettner, Christopher D Huston, Alicia S Linford, Sarah N Buss, Eric Houpt, Nicholas E Sherman, William A Petri

**Affiliations:** 1 Department of Microbiology, University of Virginia, Charlottesville, Virginia, United States of America; 2 Department of Medicine, University of Vermont, Burlington, Vermont, United States of America; 3 Department of Microbiology, University of Vermont, Burlington, Vermont, United States of America; 4 Department of Medicine, University of Virginia, Charlottesville, Virginia, United States of America; 5 Department of Pathology, University of Virginia, Charlottesville, Virginia, United States of America; Washington University School of Medicine, United States of America

## Abstract

Entamoeba histolytica is the cause of amebic colitis and liver abscess. This parasite induces apoptosis in host cells and utilizes exposed ligands such as phosphatidylserine to ingest the apoptotic corpses and invade deeper into host tissue. The purpose of this work was to identify amebic proteins involved in the recognition and ingestion of dead cells. A member of the transmembrane kinase family, phagosome-associated TMK96 (PATMK), was identified in a proteomic screen for early phagosomal proteins. Anti-peptide affinity-purified antibody produced against PATMK demonstrated that it was a type I integral membrane protein that was expressed on the trophozoite surface, and that co-localized with human erythrocytes at the site of contact. The role of PATMK in erythrophagocytosis *in vitro* was demonstrated by: (i) incubation of ameba with anti-PATMK antibodies; (ii) PATMK mRNA knock-down using a novel shRNA expression system; and (iii) expression of a carboxy-truncation of PATMK (PATMK_Δ932_). Expression of the carboxy-truncation of PATMK_Δ932_ also caused a specific reduction in the ability of E. histolytica to establish infection in the intestinal model of amebiasis, however these amebae retained the ability to cause hepatic abscesses when directly injected in the liver. In conclusion, PATMK was identified as a member of the TMK family that participates in erythrophagocytosis and is uniquely required for intestinal infection.

## Author Summary

There is a highly ordered process by which the parasite Entamoeba histolytica interacts with human cells. Adherence via a parasite lectin is followed in seconds by killing, with only the corpse and not a living cell ingested by the ameba. This process is so central to pathogenesis that clinicians use the presence of ingested erythrocytes to identify E. histolytica and distinguish it from harmless commensal amebae of the gut. We hypothesized that identification of molecules involved in the ingestion of the corpse might provide insight into how amebae cause colitis. We identified a member of the transmembrane kinase family as an early component of the phagosome. Inhibition of this kinase blocked red cell ingestion and prevented amebae from colonizing and invading the gut. There was no impact on dominant-negative parasites to cause liver abscess, suggesting the pathogenesis program differs between anatomic sites. Future studies of the transmembrane kinanse in erythrophagocytosis may provide insight into how amebae colonize and invade the gut, with the ultimate goal of preventing disease.

## Introduction

*Entamoeba histolytica,* the causative agent of amebiasis, is estimated to be the second leading cause of morbidity and mortality among protozoan parasites worldwide [1]. Phagocytosis has been one of the most recognized behaviors of E. histolytica. Erythrophagocytosis has even been used as a diagnostic indicator of invasive E. histolytica infection by microscopy [2]. Still, little is known concerning why host cells are ingested and/or what affect this has on the course of disease.

Invasive infection by E. histolytica leads to dramatic tissue destruction [3–6], including hallmarks of both apoptotic and necrotic host cell death [7–9]. Previous work has demonstrated that following contact by *E. histolytica,* host cells display many features of apoptosis including DNA laddering, caspase 3 activation and phosphatidylserine (PS) exposure. Apoptotic host cells are subsequently ingested by the ameba [10], an interaction which has been shown to involve exposed phosphatidylserine (PS) on the host cell surface [10,11]. In vitro, calcium treatment of erythrocytes causes externalization of phosphatidylserine and an increase in amebic uptake, providing a convenient and physiological model for analysis of this process [11]. Although little is known concerning the role of this behavior in disease, phagocytosis has been suggested as a virulence determining factor [12]. Amebic clones [13], and engineered mutants by either expression of dominant negative constructs [14], or by chemical mutagenesis [15] which display defective *in vitro* phagocytosis are less virulent *in vivo*. In addition, use of pan-caspase inhibitors to interfere with apoptotic induction *in vivo* has also reduced infection by this parasite [16]. Given these results we hypothesized that the identification of proteins which participate in the ingestion of the apoptotic corpse would be key to understanding virulence.

Many individual groups have used the process of ingestion of beads to identify essential proteins required for phagocytosis in organisms ranging from amebae to man [17–19]. Criticisms concerning the physiological relevance of bead ingestion have recently been dispelled by data demonstrating that bead ingestion is sensitive to inhibition by Annexin V, similar to uptake of apoptotic cells [20]. Although large scale proteomic analysis has revealed many interesting proteins, there appears to be much more left to discover. Controversies concerning both the PS receptor [21] as well as the role of the endoplasmic reticulum in phagocytosis [22,23] indicate that these efforts have not been exhaustive.

Two independent groups have published work using latex beads that were either carboxylated or opsonized with IgG to identify the constituents of the E. histolytica phagosome [24–28]. These proteomic screens taken together with the E. histolytica genome [26] have identified homologues of phagosome maturation proteins seen in metazoans. Rab7, Rab11, Rap2, PI3K, Rac1 and Rho all appear, consistent with other systems. However, some metazoan proteins including EEA1, RIN1, and LAMPs do not have discernable homologues in the E. histolytica genome. These recent screens have identified only a small number of surface proteins that could act early in the phagocytic pathway, including Hgl, Igl, ABC transporter, p-glycoprotein-2 and 6, and M17. Of these, only Hgl and Igl have been confirmed as constituents of phagosomes [24].

Amebic adherence to apoptotic host cells has been shown to require receptors in addition to the amebic Gal/GalNAc adherence lectin that mediates adherence to and killing of the live cell [11]. Our previous work identified the exposure of PS on the host cell surface as one of the recognized ligands leading to ingestion. The goals of these studies were to identify possible candidate amebic surface proteins with a role in the process of host cell ingestion.

## Results

### Sequencing of Magnetic Bead Preparations Revealed Proteins with Conserved Function in Phagocytosis

Ingested carboxylated magnetic beads within intact phagosomes were separated from amebic lysate and subjected to delipidation and mass spectrometry sequencing. Phagosome preparations were performed at 0, 5, 10, and 60 minutes following centrifugation of the beads into contact with trophozoites. In addition lysed ameba were incubated with beads and subjected to the same steps to account for background binding to the carboxylated beads. This resultant proteome identified many proteins previously associated with phagocytosis in the literature, including: the galactose binding lectin, small GTPases, hydrolytic proteins, cytoskeleton proteins, and endoplasmic reticulum proteins (Tables 1 and S1–S4). Our prediction was that molecules important for regulating ingestion of the host cell would appear in early phagosomes, but would not be present in late preparations. Therefore, the data were sorted for proteins that appeared at 0, 5 and/or 10 minutes. A member of the transmembrane kinase family stood out in this analysis, which we named PATMK (Phagosome-associated transmembrane kinase). This putative receptor kinase appeared at 5 and 10 minutes but in no other preparations.

**Table 1:**
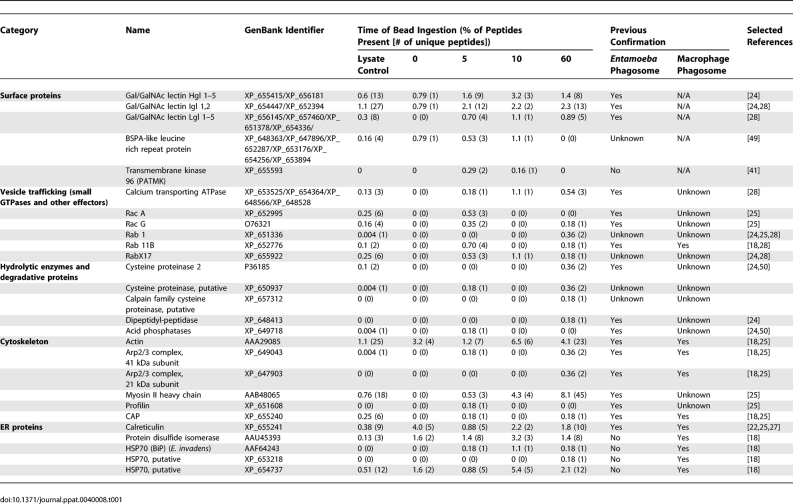
Examples of Proteins Identified in the Amebic Phagosome

**Figure 1:**
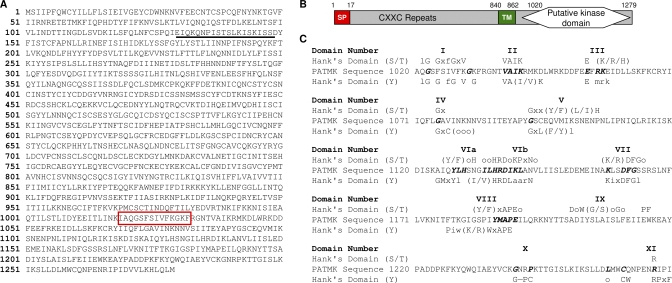
Sequence and Domain Structure of PATMK (NCBI ID: XP_655593) (A) Amino acid sequence with the sequence used to produce anti-peptide antibodies underlined, and the boxed sequence showing the peptide found in 5- and 10-minute phagosome preparations. (B) Domains of PATMK. The transmembrane domain begins at amino acid 841. (C) Alignment of PATMK with Hank's consensus of conserved residues for serine/threonine or tyrosine kinase. Upper cased residues are conserved, and positions requiring any amino acid are denotated by “X,” whereas positions requiring hydrophobic residues are denoted by “O”.

The amino acid sequence of PATMK (Figure 1A) predicted a 146 kDa protein, containing a 21 amino acid signal peptide sequence, an ectodomain containing 25 CXXC repeats, a 22 amino acid membrane spanning domain and an intracellular domain with the catalytic residues of a kinase (Figure 1B and 1C) [29]. The kinase specificity for PATMK could not be predicted given that its sequence contained the necessary residues for both serine/threonine and tyrosine kinase family members. Attempts to biochemically define the in vitro kinase activity by expressing the kinase domain in E. coli or by immunoprecipitating PATMK from trophozoites were unsuccessful (data not shown). Whether PATMK is a pseudokinase, as the lack of a conserved ATP-orienting glycine-rich motif suggests (domain I, Figure 1C), will require additional studies. We concluded that the presence of PATMK in the early phagosome proteome was consistent with it having a role in phagocytosis of the apoptotic corpse.

### Antibodies against the Ectodomain of PATMK Reveal a Surface Protein on E. histolytica

**Figure 2:**
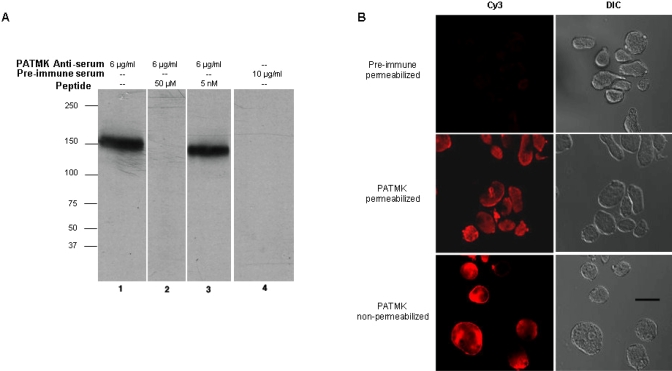
PATMK Is Expressed on the Surface of Trophozoites as a Type 1 Integral Membrane Protein (A) Western blot with affinity-purified anti-PATMK peptide antibodies was performed on amebic lysates in the absence (lane 1) or presence of 50-μM (lane 2) and 5-nM (lane 3) competing peptide, or pre-immune sera (lane 4). (B) Confocal microscopy of E. histolytica trophozoites stained with pre-immune or affinity purified anti-PATMK antibodies with or without cell permeabilization with 0.2% Triton X-100. Magnified 40×.

Rabbit anti-serum against a peptide specific for the ectodomain of PATMK (EIQKQNPISTSLKISKISSD) (underlined in Figure 1A) revealed a single band at ∼140 kDa. This band disappeared when antibody was pre-absorbed against 50 μM but not 5 nM of the antigen peptide (Figure 2A). Affinity-purified anti-PATMK antibodies stained the surface of both permeabilized and non-permeabilized trophozoites, whereas pre-immune serum yielded little staining (Figure 2B). We concluded that PATMK was expressed in E. histolytica trophozoites as a plasma membrane protein of the expected mass and with the amino-terminus extra-cellular.

### PATMK Co-Localizes with Ingested Beads

**Figure 3:**
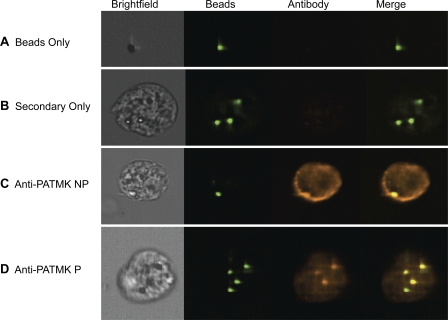
PATMK Co-Localizes with Carboxylate-Modified 2.0-mm Beads during Phagocytosis (A) Anti-PATMK did not directly bind to beads in the absence of amebae. 2 μm carboxylate-modified fluorescent beads were stained with anti-PATMK and RPE-conjugated goat-anti-rabbit antibodies as described and representative images are shown. (B) Ingested beads did not co-localize with anti-rabbit IgG:PE in a permeabilized cell. Ameba were allowed to ingest beads, fixed and stained with RPE-conjugated goat-anti rabbit antibodies. (C) PATMK co-localized with an ingested bead at the surface of an unpermeabilized cell. (D) PATMK co-localized with ingested beads in a permeabilized cell. Amebae were allowed to ingest beads, left unpermeabilized (C) or permeabilized (D) and stained as described with anti-PATMK and secondary antibody.

In order to understand the role PATMK played in ingestion, E. histolytica trophozoites were stained with anti-PATMK after ingestion of 2 μm carboxylate-modified fluorescent beads. To ensure that the PATMK antibody did not directly bind beads, we used identical methods to stain beads and bead containing cells and imaged the cells using the Amnis Imagestream imaging cytometer (Amnis Corporation; Seattle, WA). There was no evidence of non-specific binding of anti-PATMK to the carboxylate-modified beads (Figure 3A). Additionally, no staining was observed with secondary antibody alone (Figure 3B). However, the location of PATMK aggregates in both permeabilized and non-permeabilized trophozoites did correlate with that of the ingested bead (Figure 3C and 3D), suggesting that PATMK may directly interact with cargo during ingestion.

### Anti-PATMK Pre-Incubation of Ameba Blocks Ingestion of Human Erythrocytes

**Figure 4:**
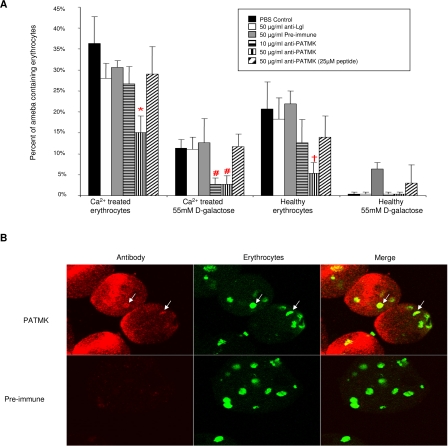
Affinity-Purified Anti-PATMK Antipeptide Antibodies Block Erythrophagocytosis by E. histolytica (A) Phagocytosis of calcium-treated or healthy erythrocytes by amebae was assayed in the presence of PBS (black), 50 μg/ml anti-Gal/GalNAc lectin light subunit (Lgl) (white), pre-immune (gray), 10 μg/ml anti-PATMK (horizontal hatch), 50 μg/ml anti-PATMK (vertical hatch), and 50 μg/ml anti-PATMK serum pre-absorbed with 25 μM of peptide (diagonal hatch). Data are reported as means ± SD. *p* Values were determined by a two-tailed t-test compared to controls (*, *p* < 0.003 compared with pre-immune in M199s; #, *p* < 0.046 compared to pre-immune in 50 mM D-galactose; †, *p* < 0.002 compared to pre-immune in M199s [healthy erythrocytes], *n* = 6). (B) E. histolytica trophozoites interacting with CFSE-labeled erythrocytes were stained with pre-immune or anti-PATMK serum, magnified 100×.

Amebae were tested for their ability to ingest healthy or calcium-treated (apoptotic) erythrocytes following a 20 minute pre-incubation on ice in medium containing 50 μg/ml of anti-PATMK antibodies (Figure 4A). Anti-PATMK antibodies reduced the ingestion of calcium-treated (15.0% ± 4.0% *vs.* 30.7 ± 1.5%, *p* ≤ 0.003), as well as healthy erythrocytes (5.3% ± 2.5% *vs.* 22.0% ± 3.0%, *p* ≤ 0.002). Inhibition was also observed for the ingestion of calcium treated erythrocytes in the presence of 55 mM galactose (2.7% ± 2.1% *vs.* 12.7 ± 5.7, *p* ≤ 0.046). The effect of anti-PATMK antibodies could be reversed by pre-absorbing the antibodies against the antigen peptide at a concentration of 25 μM (Figure 4A). As expected, anti-Lgl antibodies (used as a control) had no affect on ingestion of either healthy or calcium-treated erythrocytes. We concluded that the ability of the anti-PATMK antibodies to block phagocytosis of not only healthy, but also calcium-treated, erythrocytes indicated a role for PATMK in erythrophagocytosis at a step after apoptotic killing of the red cell by the amebae.

### PATMK Localizes to the Interface of Ameba with Erythrocytes

In order to understand the role PATMK played in ingestion, E. histolytica trophozoites were stained with anti-PATMK in the presence of fluorescently labeled (CFSE) erythrocytes (Figure 4B). PATMK staining on non-permeabilized trophozoites enriched at the site of contact with CFSE labeled erythrocytes (arrows, Figure 4B), suggesting that PATMK may directly interact with the erythrocyte during ingestion.

### ShRNA Knockdown of PATMK Led to a Reduction in Erythrophagocytosis

**Figure 5:**
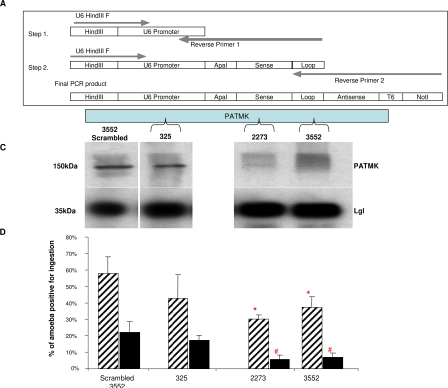
Expression of Short Hairpin RNA against PATMK Reduced PATMK Protein Levels and the Rate of Amebic Ingestion of Erythrocytes (A) Short hairpin RNAs (shRNA) were created using a two-step PCR strategy that utilized overlapping 3′ primers to create a hairpin loop controlled by an RNA polymerase III (U6) promoter. (B) The shRNAs corresponded to nucleotides 325–354 (325), 2273–2302 (2273), and 3552–3581 (3552), as well as a control which contained the same nucleotide makeup of 3552 in random order (scrambled). (C) Amebic cell lysate (105 cells per lane) was separated on an 8% SDS polyacrylamide gel, transferred to PVDF, and blotted with anti-PATMK or anti-Lgl serum (as a loading control). (D) Phagocytosis of calcium-treated erythrocytes by amebae transfected with the shRNAs were assayed in M199S (black bars) or M199S competed with 55 mM D-galactose (hatched bars). Data are reported as means ± SD. *p* Values were determined by a two-tailed t-test compared to scrambled controls (*, *p* < 0.039, compared to Scrambled 3552; #, *p* < 0.021 compared with Scrambled 3552 in 50 mM D-galactose, *n* = 6).

In order to further address the role of PATMK in phagocytosis, RNA interference using a novel short hairpin RNA (shRNA) system was used to knock down its expression. ShRNAs were stably expressed in amebae using the E. histolytica U6 promoter (RNA polymerase III) (Figure 5A). ShRNAs were expressed to three regions of PATMK (Figure 5B). Knockdown of PATMK protein was seen upon expression of shRNA to two regions of PATMK, nucleotides 2273–2302, and 3552–3581, but not to a scrambled shRNA of identical composition to nucleotides 3552–3581 (Figure 5C). Knockdown of PATMK inhibited erythrophagocytosis: in the absence of galactose, ingestion of calcium-treated erythrocytes was statistically significantly reduced by 48.3% by the 3552–3581 corresponding hairpin (58.0% ± 9.8% *vs.* 30.0% ± 2.6%, *p* ≤ 0.0176) and 35.6% by the 2273–2302 hairpin (58.0% ± 9.8% *vs.* 37.3% ± 6.4%, *p* ≤ 0.0383) compared with the scrambled control. In the presence of 55 mM galactose, both constructs (2273 and 3552) reduced ingestion of calcium treated erythrocytes by more than 65% (22.0% ± 6.6% (scrambled 3552 control) *vs.* 5.7% ± 2.5% (3552) or 7.0% ± 2.6% (2273), *p* ≤ 0.02) (Figure 5D). The amino-terminus most construct (325–354) neither reduced PATMK protein levels nor had a significant affect on ingestion of calcium treated erythrocytes. No off-target effects on mRNA levels were observed with the shRNA technique (Table 2). We concluded both that shRNA is a promising technique for gene knockdown in *E. histolytica,* and that inhibition of the ingestion of calcium-treated erythrocytes by PATMK knockdown supported a role for PATMK in the ingestion of dead red cells.

**Table 2:**
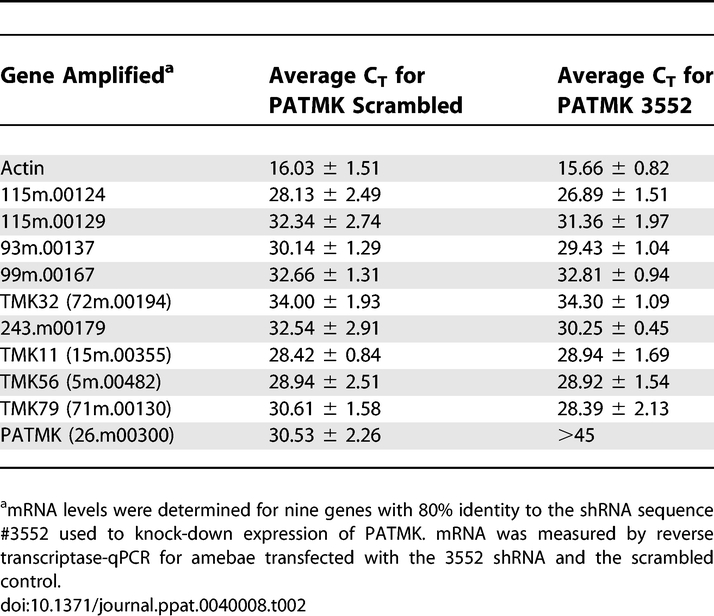
shRNA against PATMK but Not the Scrambled Control shRNA Decreased PATMK mRNA Levels without Evidence of Off-Target Effects

### Expression of a Truncated Form of PATMK at Residue 932 (PATMK_Δ932_) Caused a Reduction in Host Cell Ingestion

**Figure 6:**
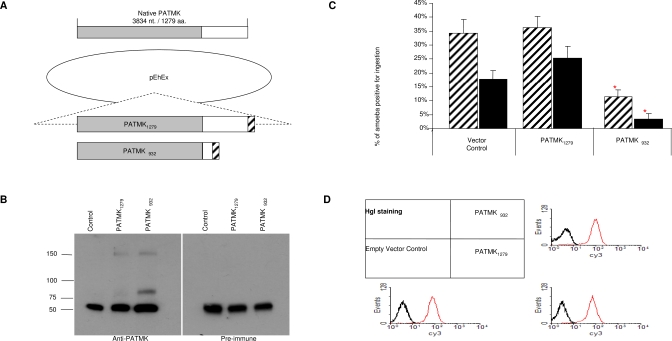
Expression of Carboxy-Truncated PATMK Reduced Ingestion of Eythrocytes by E. histolytica (A) Two constructs were assembled by PCR and cloned behind the cysteine synthase promoter in the vector pEhEx and transfected into HM1:IMSS trophozoites: a full-length, carboxy Flag epitope tagged PATMK (PATMK1279), and a truncation at residue 932, with a carboxy Flag epitope tag (PATMK_932). (B) Amebic lysates (107 cells of PATMK1279, PATMK_932, or empty vector) were subjected to immunoprecipitation using anti-Flag resin. Proteins from the IP were separated on an 8% polyacrylamide gel, transferred to PVDF and blotted with anti-PATMK or pre-immune serum. (In every lane, the heavy chain from the immunoprecipitating antibody appears at ∼50 kDa). (C) Phagocytosis of calcium-treated erythrocytes by amebae expressing PATMK_932, PATMK1279, and empty vector controls were assayed in M199S (hatched bars) or M199S competed with 55 mM D-galactose (black bars). Data are reported as means ± SD. *p* Values were determined by a two-tailed t-test compared to empty vector controls (*, *p* < 0.003, *n* = 6). (D) Amebic surface staining was performed on non-permeablized fixed E. histolytica trophozoites using pre-immune (bold line) or anti-Gal/GalNAc Hgl specific serum (thin line) and analyzed by flow cytometry.

Because gene replacement is not currently possible in *E. histolytica,* we sought a fourth (in addition to shRNA knockdown, anti-PATMK antibodies and proteomics identification of PATMK in the early phagosome) independent approach to test the role of PATMK in erythrophagocytosis. PATMK_1279_ (full length carboxy-FLAG epitope-tagged PATMK), PATMK_Δ932_ (a truncated, carboxy-FLAG epitope-tagged PATMK), and empty vector control (Figure 6A) were stably transfected into trophozoites. Immunoprecipitations were performed using anti-FLAG binding resin, and Western blots revealed that both PATMK_1279_, and PATMK_Δ932_ were expressed (Figure 6B). Interestingly, the truncated form of PATMK_Δ932_ was co-immunoprecipitated with native PATMK, as indicated by western blots of the immunoprecipitate indicating interaction of the truncated and wild type proteins (Figure 6B, lane 3). The expression of the carboxy-terminal truncated form of PATMK reduced erythrophagocytosis by 67% (11.33 ± 2.52 *vs.* 34.33 ± 4.93, *p* ≤ 0.002), and by 81% in the presence of 55 mM D-galactose (3.33 ± 2.08 *vs.* 17.67 ± 3.06, *p* ≤ 0.003) compared with the empty vector control (Figure 6C). Expression of PATMK_1279_ had no statistical impact on erythrophagocytosis. To ensure that the expression of PATMK_Δ932_ did not dramatically alter the amebic surface, transfectants were stained with anti-Gal/GalNAc lectin heavy subunit polyclonal serum, and analyzed using flow cytometry (Figure 6D). There were no differences observed in lectin staining between any of the transfectants. We concluded that the ability of the truncated form of PATMK to inhibit erythrophagocytosis was consistent with all of the previous experiments implicating PATMK in ingestion of the dead red cell. The co-immunoprecipitation with anti-FLAG antibody of the carboxy-truncated PATMK with the native full-length PATMK suggested that the truncated protein was interfering with PATMK via a direct interaction.

### Expression of PATMK_Δ932_ Reduces Virulence of *E. Histolytica* in the Intestinal Model of Amebiasis, but Has No Affect on Formation of Liver Abscesses

Because parasite erythrophagocytosis is pathognomonic of amebic colitis, we tested the ability of amebae expressing carboxy-truncated PATMK to infect the colon in the murine model of amebic colitis. As expected, the infection rate of trophozoites transfected with PATMK_Δ*932*_ (Table 3) was significantly decreased (2/24 PATMK_Δ932_ *vs.* 9/24 empty vector, *p* ≤ 0.0157). Amebae cultured from the sacrificed animals still contained the transfected plasmids. The decreased virulence in the intestine was not due to a general enfeeblement of these amebae, as no reduction was seen in the ability to cause liver abscesses in gerbils (Table 4).

**Table 3:**
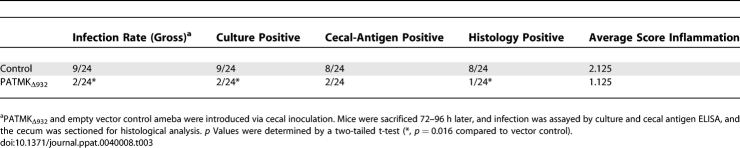
Expression of a PATMK Truncation Reduced E. histolytica Infection and Inflammation in the Intestine

**Table 4:**
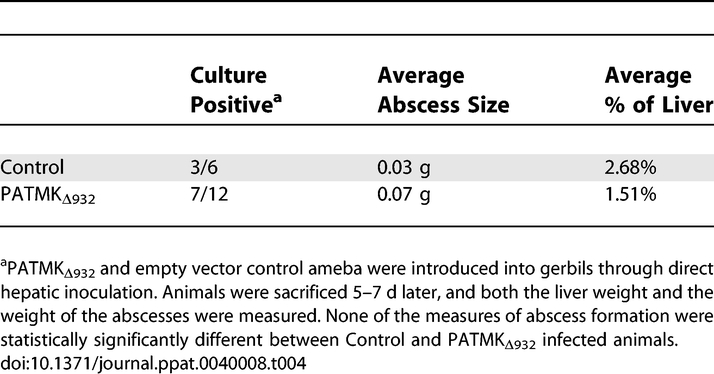
Expression of a PATMK Truncation Did Not Reduce Liver Abscess Formation

## Discussion

The most important finding of this study was the identification of PATMK as a member of the TMK family that participates in erythrophagocytosis and is uniquely required for intestinal but not hepatic infection. In addition this manuscript introduced a novel technique of gene knockdown using shRNA in E. histolytica that could find general usefulness in a parasite for which there is not the ability to replace genes.

The participation of PATMK in ingestion of the dead human red cell was demonstrated not only by shRNA but by identification in the early phagosome proteome, co-localization with erythrocytes, and inhibition with anti-peptide antibodies and by expression of a carboxy-truncated mutant. Enzymatic activity of the kinase domain was not demonstrated when PATMK was expressed and purified from E. coli or immunoprecipitated from *E. histolytica,* Further studies will therefore be required to understand if PATMK is acting as a receptor for apoptotic red cells or as a regulator of ingestion via its kinase domain.

A comprehensive analysis of the phagosome proteome was not the goal of this work, as several groups have accomplished this for *E. histolytica*. However it is interesting that each group has identified new molecules of interest. The first analysis of this type in E. histolytica, published by Okada *et al.*, revealed many of the GTP binding proteins that are important for maturation of phagosomes [24]. However, this screen did not reveal any of the endoplasmic reticulum resident proteins identified in metazoan systems [17,22]. More recent work by Marion *et al*. identified ER resident proteins in E. histolytica phagosomes such as calreticulin as well as significant involvement by myosin IB, actin and actin accessory proteins [25]. Later this group identified a small number of surface proteins which were suggested as possible receptors [27]. The screen reported here was focused on early time points following ingestion with the goal of identifying new surface molecules with roles in this process. In addition proteins were identified such as amoebapore A and B, which were known to be involved in phagocytosis but had remained absent in other screens. The most obvious conclusion from this collection of proteomics is that not one effort has taken its screen to saturation. Incompleteness of these screens may also explain the differences that have been published between clinical isolates of E. histolytica [28].

Manipulation of PATMK by binding the ectodomain with antibody (75% reduction of ingestion of calcium treated erythrocytes in M199S with 55mM D-galactose), reducing the protein levels with interfering RNA (65% reduction in M199S with 55mM D-galactose), or by expressing a truncated protein (81%), all produced a similar reduction of erythrophagocytosis *in vitro.* The mechanism of this interference is not entirely clear. Given that anti-PATMK serum co-localizes with erythrocytes in contact with ameba, we assume that antibody against this protein blocks uptake of erythrocytes by interfering with receptor function, but it is very possible that this could be blocking interaction with a different molecule by steric hinderance or by interfering with signaling (as opposed to receptor) functions of PATMK. Clearly all of the experimental approaches supported a role for this protein in host cell ingestion by E. histolytica.

Although no kinase activity has been demonstrated, we hypothesize that PATMK is in fact a receptor kinase. The Mer family of tyrosine kinases provide a paradigm for receptor kinase involvement in phagocytosis [30–32]. Mer tyrosine kinase interacts with the bridging molecule GAS6 to recognize PS on apoptotic cells. The identification of the TMK family was one of, if not the most exciting finding from the genome project, because of its lack of precedent in a unicellular eukaryote. Implication of PATMK in erythrophagocytosis is an important step towards understanding the role of this family of kinases in parasite biology.

Virulence of E. histolytica has been long associated with the parasite's ability to ingest host cells. This work suggests that this may be more important in intestinal disease than in liver abscesses. Ameba expressing PATMK_Δ932_ had reduced ability to infect the intestine but were not impaired in causing liver abscess when directly injected into the liver. A varying requirement for virulence factors in different environments for the amebae has been seen before*.* For example cysteine protease 2 over-expression was found to reduce *in vitro* monolayer destruction but had no effect on liver abscess formation [33], and amoebapore A silencing led to inability to cause liver abscesses although these parasites still caused tissue damage in a colonic xenograft model of amebiasis [34]. The data presented here concerning a retention of virulence in the liver abscess model as well as the analysis showing that lectin expression on the surface has not been altered illustrate that the ameba are otherwise competent to attach to the host and cause disease. This may indicate that expression of PATMK_Δ932_ does not simply produce an impaired ameba, but an ameba which fails to colonize the intestine possibly because of failure to clear dead and dying host cells. It also suggests that the virulence program required for the parasite's success in the gut differ from that of the liver.

The rationale behind phagocytosis of host cells by this parasite is still a mystery, but erythrophagocytosis is a hallmark of E. histolytica infection. One could envision that this behavior provides an advantage during infection by clearing dying and dead cells and thereby reducing the infiltration of inflammatory cells and release of toxic cellular content. PATMK may have a pivotal role in allowing this parasite to persist longer in the host. Interfering with this pathway may produce a more robust immune response to E. histolytica and clearance of infection.

## Materials and Methods

### Cells and storage conditions.

E. histolytica trophozoites (HM-1:IMSS) were grown axenically in TYI-S-33 (Trypticase-yeast extract iron serum) medium supplemented with 100 U of penicillin/ml and 100 μg of streptomycin sulfate/ml at 37 °C [35]. Trophozoites were harvested during log-phase growth by incubation on ice for 10 min, centrifugation at 200 × g and 4 °C for 5 minutes, and resuspension in either HEPES buffer or medium 199 (Gibco BRL, Grand Island, NY) supplemented with 5.7 mM cysteine, 25 mM HEPES, and 0.5% bovine serum albumin at pH 6.8, (M199S) [36]. Human blood type B Rh+ was collected, heparinized and sedimented by centrifugation (1,000 × *g* ; 4 °C; 10 minutes) through Ficoll-Paque PLUS (Amersham Biosciences, Piscataway, NJ) to separate erythrocytes from other blood constituents. Pelleted erythrocytes were washed twice in HEPES buffer (10mM hydroxyethylpiperazine-N′-2-ethanesulfonic acid (HEPES) [pH 7.2], 140 mM NaCl and 0.1% bovine serum albumin with or without 2.5 mM CaCl_2_), and resuspended at 1 × 10^7^ cells per ml in HEPES buffer and stored for up to 48 hours [37,38]. Calcium-treated erythrocytes were prepared by incubation in HEPES buffer supplemented with 2.5 mM CaCl_2_ at 37 °C for 48 hours.

### Preparation of amebic phagosomes using magnetic beads.

Adherent amebae in log phase growth were washed three times with warm PBS and carboxylated 2.7 μm magnetic beads (M-270 Dynabeads, Dynal, Inc.) (2 × 10^8^ beads/ml in medium 199 (Gibco BRL) supplemented with 5.7 mM cysteine, 25 mM HEPES, and 0.5% BSA at pH 6.8 (M199s) were added to the cultures. Phagocytosis was then initiated by centrifugation (200 × *g*, 5 min, room temperature) of the beads onto amebae using a plate spinner. After incubation at 37 °C for 0, 5, or 10 min, uningested beads were removed by washing 3× with warm PBS followed immediately by addition of cold PBS and incubation on ice for 5 min to harvest the amebae. For longer time points, beads were removed after incubation for 10 min and a “chase” incubation at 37 °C was performed. Amebae were then resuspended in cold PBS supplemented with a protease inhibitor cocktail and mechanically disrupted using a Dounce homogenizer. Douncing was continued until approximately 90% of amebae were lysed, leaving the nuclei intact. Phagosomes containing ingested magnetic beads were isolated using a magnetic column, washed three times with PBS, and de-lipidated by methanol:chloroform precipitation in preparation for peptide sequencing [39].

### Peptide sequencing and analysis.

The sample was dissolved in 10–20 μl of 1% SDS and then slowly diluted to a final concentration of 0.1% SDS with 100mM ammonium bicarbonate pH 8.0. The sample was reduced with 100mM DTT for 30 minutes at room temperature and alkylated with 500mM iodoacetamide for 30 minutes at room temperature (RT) before addition of 1 μg of modified trypsin (Promega, Madison, WI) for 24 hours at RT. A second 1 μg of trypsin was added for an additional 24 hours at RT. The sample was acidified with acetic acid to 5% by volume. The resulting digest was desalted on a C18 column (10μm particle size – 10cm × 150μm id) and then SDS removed by strong cation ion exchange (10μm particle size – 10cm × 150μm id). 25% of the sample was injected into the mass spectrometer. The LC-MS system consisted of a Finnigan LCQ ion trap mass spectrometer system with a Protana nanospray ion source interfaced to a self-packed 8 cm × 75 um id Phenomenex Jupiter 10μm C18 reversed-phase capillary column. The peptides were eluted from the column by an acetonitrile/0.1 M acetic acid gradient at a flow rate of 0.5μL per minute over 2 hours. The nanospray ion source was operated at 2.8 kV. The digest was analyzed using the double play capability of the instrument acquiring a full scan mass spectrum to determine peptide molecular weights and four product ion spectra to determine amino acid sequence in sequential scans. The data were analyzed by database searching using the Sequest search algorithm against the non-redundant database from NCBI and against the E. histolytica ORFs generated from the TIGR sequenced genome database. Search results were analyzed using minimum cutoffs (Xcorr >1.5 for +1, >2.0 for +2 and >2.5 for +3). Any proteins of interest were confirmed by manual validation of the spectra.

### Production of anti-PATMK rabbit serum.

The peptide EIQKQNPISTSLKISKISSD, (amino acids 130–150 of PATMK) was synthesized, conjugated to KLH and used to immunize New Zealand White Rabbits (Covance, Princeton, NJ). Resultant serum was protein G purfied using packed protein G columns (Pierce, Rockford, IL) and affinity purified against bound peptide. The resultant serum was dialyzed against PBS and stored at −20 °C until use.

### Fluorescent labeling and antibody pre-incubation.

Prior to calcium treatment, erythrocytes were fluorescently labeled by incubation at 37 °C for 20 to 25 minutes in phosphate-buffered saline (PBS) containing 5μM 5 (and 6)-carboxyfluorescein diacetate succinimidyl ester (CFSE) (Molecular Probes, Eugene, OR). Unbound dye was quenched by incubation with an excess of fetal bovine serum at 37 °C for 20 min, and the cells were washed twice more with M199s medium before use. Where indicated, erythrocytes were washed once in M199S and re-suspended at 10^6^ cells/ml and incubated with (antibody concentration) per 25 minutes at 4 °C. The cells were then washed twice in M199S before they were added to amebae [40].

### Immunopreciptiation and western blotting.

Soluble proteins were extracted from ameba by harvesting 5 × 10^7^ trophozoites expressing FLAG epitope-tagged PATMK by incubation on ice for 10 minutes, followed by centrifugation (200 × *g* at 4 °C for 5 minutes). The amebae were lysed in Lysis Buffer (150mM NaCl, 50mM Tris-HCl, and 1% NP-40, supplemented with protease inhibitor cocktail I [Sigma, St. Louis, MO] per the manufacturer's directions). The amebic lysate was incubated with anti-FLAG-M2 affinity gel (Sigma, St. Louis, MO) for 30 minutes at 4 °C. Resin was washed once in lysis buffer and twice in PBS pH 7.4, then boiled in SDS-PAGE loading buffer. All samples were then separated on 8–10% polyacrylamide gels and subjected to immuno-blotting by standard techniques [41] with either 5 μg/ml anti-PATMK rabbit serum or pre-immune serum.

### Confocal microscopy.

E. histolytica trophozoites (1 × 10^6^) were bound to glass coverslips in a 24-well plate for 30 minutes at 37 °C in TYI-S-33 medium. Adherent amebae were washed twice in phosphate buffered saline (PBS) and fixed in 3% paraformaldehyde for 30 minutes at room temperature. Where indicated 10^7^ CFSE-labeled erythrocytes were incubated for 10 minutes with washed ameba in M199S at 37 °C, prior to formaldehyde fixation. Next, amebae were solubilized in 0.2% Triton X 100 in PBS for 1 minute. Nonspecific binding was blocked by incubation with 20% goat serum and 5% bovine serum albumin (Sigma, St. Louis, MO) in PBS for 1 h at 37 °C. Detection of kinases was performed by incubation with protein A purified anti-PATMK rabbit polyclonal antibody (anti-PATMK) diluted to 200 μg/ml and incubated with fixed cells for 1 hour at 37 °C. Detection of the Gal/GalNAc adherence lectin was detected by addition of anti-lectin rabbit polyclonal antibody [41] diluted to 6 μg/ml. Two quick washes were performed before Cy3-conjugated goat anti-rabbit secondary antibodies (Jackson Laboratories, Bar Harbor, ME) were added at a 1:160 dilution for 1 hour at 37 °C. The coverslips were washed twice more and mounted with Gel/Mount (Biomeda, Foster City, CA) and sealed to slides. Confocal images were visualized using a Zeiss LSM 510 laser scanning microscope.

### Construct assembly.

Cloning of PATMKcterm, for expression of 6XHIS tagged protein: the kinase containing region of PATMK was PCR amplified with the primers: CAATTTAGAGAAGGAATTCCT (5′ primer) and TCACATTAATTGAAGATGTTTTAAAACAACA (3′ primer). This 1000 bp fragment was then cloned into TOPO NT/T7 (Invitrogen, Carlsbad, California), in frame with an amino-terminal 6X HIS tag.

Cloning of U6 driven RNAi hairpin constructs for PATMK knockdown (Figure 2A, lane 1): short hairpin RNAs were expressed from by the E. histolytica RNA polymerase III, U6 promoter (GenBank [http://www.ncbi.nlm.nih.gov/] accession number U43841) [42]. This approach utilized a 29-base base pair stem, with a 9 bp loop. Four sequences were targeted and construction of the vector was done in 2 parts. Three experimental constructs were made (325, 2273, and 3552) each corresponding to the corresponding beginning nucleotide sequence as well as a control with the same nucleotide content as 3552, but in random order (scrambled). These constructs were made using two rounds of PCR, each using the same 5′ oligo for each round, (CTACTGAAGCTTGTTTTTATGAAAAAGTGTATTTGC) but different 3′ oligos as listed: 325 first round TCTCTTGAAAGAATCTTATCAACTGATAAGTCTCCAGGGCCCAATTTTATTTTTCTTTTTATCC, second round GAATGCGGCCGCAAAAAATGGAGACTTATCAGTTGATAAGATTCTTCTCTTGAA; 2273 first round TCTCTTGAAACACTCATATTGTTCTAAATAATACCCTTGGGCCCAATTTTATTTTTCTTTTTATCC, second round GAATGCGGCCGCAAAAAAGGGTATTATTTAGAACAATATGAGTGTTCTCTTGAA; and 3552 first round TCTCTTGAAGCCATATAAATTGGACTTCCTATTCCCTTGGGCCCAATTTTATTTTTCTTTTTATCC, second round GAATGCGGCCGCAAAAAAGGGAATAGGAAGTCCAATTTATATGGCTCTCTTGAA. PCR products were cloned into pBluescript II, sequence-verified, then subcloned into the amebic expression vector pGIR310 [43].

Cloning of carboxy-FLAG epitope tagged PATMK (PATMK_1279_) and truncated PATMK at residue 932 (PATMK_Δ932_), was performed by PCR of genomic DNA with the same forward oligo, which added a *Bgl*II site to the 5′ region of the gene (AAAGATCTTCAATGAGCATTATTCCATTTCAATGGTGCTAT). Two different 3′ oligos terminating at nucleotide 3834 to create PATMK_1279_ (sequence: AACTCGAGTTAGCCCTTGTCGTCGTCGTCCTTGTAGTCCATTAATTGAAGATGTTTTAAAACAACATCAATGGGTAT) or nucleotide2796 to createPATMK_Δ932_ (sequence: AACTCGAGTTAGCCCTTGTCGTCGTCGTCCTTGTAGTCAATTTTTAATGGATTTTTCCTTATGCTTGCTAT). Each 3′ oligo also contained the FLAG epitope tag and an *XhoI* site for detection and cloning respectively. PCR products were cloned into vector pEhEX [44] at *Bgl*II and *Xho*I sites. Vector pEhEx without an insert was used for the empty vector controls.

### Phagocytosis assays.

Phagocytosis was assayed by microscopy as previously described [11]. Extracellular erythrocytes were lysed by a wash in distilled water prior to fixation in 3% paraformaldehyde. Phagocytosis positive amebae were defined by microscopy as ameba containing one or more ingested erythrocytes. Both the numbers of positive amebae, as well as the numbers of intact, engulfed, erythrocytes were counted. These results were expressed as a phagocytic index, which was the percentage of amebic trophozoites that had engulfed erythrocytes multiplied by the average number of erythrocytes ingested per ameba [45]. Anti-PATMK blocking experiments were performed using this same approach with the addition of a 20 minute incubation on ice with the antibody, control antibody, or antibody and the peptide-antigen. Excess antibody and peptide were washed away in two rinses in M199s, prior to incubation with erythrocytes.

### Flow cytometry.

Ameba cell surface changes were assessed by anti-lectin staining (10 μg/ml anti-lectin, Rabbit IgG) of paraformaldehyde fixed trophozoites for 1 hour at RT and analyzed by using a FACScan cytometer and CellQuest 3.3 software (Becton Dickinson, Franklin Lakes, N.J.).

### ImageStream data acquisition.

2 × 10^7^ carboxylate-modified 2.0 μm fluorescent yellow-green beads (Sigma, St. Louis, MO) and 2 × 10^6^ washed E. histolytica trophozoites were mixed, centrifuged (200 × g, 4 °C, 5 min) and incubated for 10 minutes in M199S at 37 °C. The samples were washed 3 times in 110 mM D-galactose, 3 times in PBS and then fixed with 3% paraformaldehyde. If indicated, amebae were permeabilized with 0.2% Triton X 100 in PBS for 1 minute. In all samples, paraformaldehyde was neutralized with 50 mM NH_4_Cl. Nonspecific binding was blocked by incubation with 5% bovine serum albumin (Sigma, St. Louis, MO) in PBS for 1 h at 37 °C. PATMK was detected by incubation for 1 hour at 37 °C with 5 μg/ml protein A purified anti-PATMK rabbit polyclonal antibody (anti-PATMK). Five PBS washes were performed before R-PE-conjugated goat anti-rabbit secondary antibodies (Jackson Laboratories, Bar Harbor, ME) were added at a 1:200 dilution for 1 hour at 37 °C. Following the incubation, samples were washed with PBS five times, resuspended in 200 μl PBS and plunged through a 26 ^3^/_8_ -gauge needle 5 times. When indicated the procedeure was carried out in the absence of ameba (beads only) or in the absence of anti-PATMK antibody (seconday only). Analysis was performed using the Amnis Imagestream imaging cytometer (Amnis Corporation; Seattle, WA) and ImageStream Data Exploration and Analysis Software (IDEAS). Prior to data analysis, spectral compensation was performed using beads and stained cells. At least 5000 images were collected and gating was performed to generate a population of single, in-focus, bead positive cell images (usually yielding <500 images).

### Animal models.

CBA mice were challenged with 2 × 10^6^ trophozoites by cecal inoculation, by previously described methods [46]. Mice were sacrificed 72–96 hours following challenge and the cecum removed for culture in TYI-S-33 medium and paraffin embedding for histological scoring as previously described [47].

Gerbils were challenged with 5 × 10^5^ trophozoites by direct hepatic inoculation by previously described methods [48]. Gerbils were sacrificed 5–8 d following challenge and liver abscess weights were determined and cultures started in TYI-S-33.

## Supporting Information

Table S1 Phagosome Proteome - Time 0 (129 KB PDF)

Table S2 Phagosome Proteome - 5 Minutes (85 KB PDF)

Table S3 Phagosome Proteome - 10 Minutes (135 KB PDF)

Table S4 Phagosome Proteome - 30 Minutes (44 KB PDF)

### Accession Number

PATMK is number: XP_655593.

## Abbreviations

BSA: Bovine Serum Albumin
CFSE: 5-carboxyfluorescein diacetate succinimidyl ester
CMTMR: 5–4-chloromethyl- benzoylaminotetramethylrhodamine
EDTA: Ethylene Diaminetetraacetic Acid
*E. histolytica,*: *Entamoeba histolytica*
FLAG: epitope tag: DYKDDDDK
FITC: Fluorescein
Gal/GalNAc lectin: Galactose/N-acetyl galactosamine binding lectin
HEPES: 4–2-hydroxyethyl-1-piperazineethanesulfonic acid
IgG: Immunoglobin G
MER: Mer tyrosine kinase
PATMK: Phagosome Associated Transmembrane Kinase
PS: Phosphatidylserine
shRNA: short hairpin RNA
TIGR: The Institute for Genomic Research
