# Supplementary material for: Entamoeba histolytica Phagocytosis of Human Erythrocytes Involves PATMK, a Member of the Transmembrane Kinase Family
Source: PLoS Pathog. 2008 Jan 18;4(1):e8. doi: 10.1371/journal.ppat.0040008 (PMC2211552; doi:10.1371/journal.ppat.0040008)
Supplement: Supplementary file 1 [file ppat.0040008.st001.pdf]

0 MINUTES

ANNOTATION	PEPTIDE SEQUENCE	MASS	CHARGE	Xcorr
gi|1835626|gb|AAB50230.1| Gal/GalNAc lectin light subunit [Entamoeba dispar]	TLEWPTYK	1037.530226	2	1.6999
gi|20799665|gb|AAM28598.1| putative trypanothione reductase [Entamoeba histolytica]	ILGVHNLGLGAPEIIQGINVK	2155.259764	2	1.8339
gi|2326422|emb|CAA74797.1| phosphoglucomutase [Entamoeba dispar]	RYPTTPISGQTM*GTSGLRK	2067.066279	2	1.5262
gi|2350917|dbj|BAA21988.1| actin [Entamoeba histolytica]	YPLEHGIVNNWDDMEK	1959.890546	2	5.7895
	YPLEHGIVNNWDDMEK	1959.890546	2	5.3689
	YPLEHGIVNNWDDMEK	1959.890546	2	5.0675
	YPLEHGIVNNWDDMEK	1959.890546	2	4.5398
	YPLEHGIVNNWDDMEK	1959.890546	3	4.4526
gi|2498767|sp|Q24824|PFPB_ENTHI Pore-forming peptide ameobapore B precursor (EH-APP)	ILSFGVDELVK	1219.693281	2	3.1606
gi|31414729|gb|AAP50909.1| chaperonin 60 [Entamoeba dispar]	IEDAVYAVKAALA	1333.736197	3	1.8994
gi|56463309|gb|EAL42451.1| 40S ribosomal protein S6, putative [Entamoeba histolytica HM-1:IMSS]	KGEGEIEGVTNDALPMR	1815.890551	3	3.6212
	REVAKKHPK	1092.663639	2	1.5182
	VVSFADTDAVFK	1298.662708	2	3.5956
	VVSFADTDAVFK	1298.662708	2	2.4406
	VVSFADTDAVFK	1298.662708	2	2.4239
gi|56463386|gb|EAL42461.1| hypothetical protein 961.t00001 [Entamoeba histolytica HM-1:IMSS]	ELSELK	718.398159	2	1.6643
gi|56463438|gb|EAL42474.1| hypothetical protein 917.t00001 [Entamoeba histolytica HM-1:IMSS]	EIEEEIFK	1036.519722	2	1.5212
gi|56463575|gb|EAL42521.1| peroxiredoxin [Entamoeba histolytica HM-1:IMSS]	GYVIIDDK	922.4880294	2	1.9357
	LTFPLVSDIK	1132.66125	2	2.5484
	LTFPLVSDIK	1132.66125	1	1.8074
	YIQMNDDGIGR	1281.58922	2	1.832
gi|56463599|gb|EAL42527.1| hypothetical protein 804.t00001 [Entamoeba histolytica HM-1:IMSS]	FGGYVNSKVDKVGYFIK	1921.021832	2	2.1273
gi|56463685|gb|EAL42555.1| hypothetical protein 748.t00001 [Entamoeba histolytica HM-1:IMSS]	VNNILFDSDK	1164.589543	2	1.6452
gi|56463710|gb|EAL42562.1| Rab family GTPase [Entamoeba histolytica HM-1:IMSS] gi|50978618|dbj|BAD34976.1| EhRab1	AITNAYYR	971.494509	2	2.257
	AQIWDTAGQER	1274.612396	2	3.8505
	AQIWDTAGQER	1274.612396	2	2.3069
	IVIMIIGNK	1000.622352	1	2.0736
	IVLIGESGVGK	1071.640856	1	2.9272
	IVLIGESGVGK	1071.640856	1	2.3306
	SLLVEIFK	948.576457	1	1.9227
	STIGVEFATR	1080.568415	1	1.6543
	WLSELK	775.434878	2	1.6091
gi|56463818|gb|EAL42601.1| hypothetical protein 651.t00002 [Entamoeba histolytica HM-1:IMSS]	IMERGM*IEDEAKSKVLLM*K	2253.167334	2	1.5468
	SLIRIMERGM*IEDEAKSK	2122.10061	2	1.5521
gi|56463820|gb|EAL42602.1| protein kinase, putative [Entamoeba histolytica HM-1:IMSS]	ISKNGSTSAR	1020.543283	2	1.6887
gi|56463856|gb|EAL42619.1| hypothetical protein 638.t00002 [Entamoeba histolytica HM-1:IMSS]	EGKEQGNSVTDDGHSNK	1801.794759	2	1.7593
	EIEKEVIR	1015.578239	2	2.6696
	EIEKEVIR	1015.578239	2	2.5562
	EIEKEVIR	1015.578239	2	2.5395
	EIEKEVIR	1015.578239	1	2.3519
	EIEKEVIR	1015.578239	1	2.0541
gi|56463918|gb|EAL42646.1| calcium-binding protein, putative [Entamoeba histolytica HM-1:IMSS]	FLDVVTSLLE	1135.624531	1	1.7821
	FLDVVTSLLE	1135.624531	1	1.7138
	PFNPMEFGK	1066.502632	2	2.43
	SGTLEINELMMGQFPGGIR	2050.009632	2	4.2524
gi|56463954|gb|EAL42664.1| RNA pseudouridylate synthase, putative [Entamoeba histolytica HM-1:IMSS]	EFQVNVK	863.462146	2	1.8407
gi|56463986|gb|EAL42679.1| protein kinase, putative [Entamoeba histolytica HM-1:IMSS]	VIGRGGFGEVRLVVKK	1714.048647	2	1.7288
gi|56464126|gb|EAL42749.1| heat shock protein 70, putative [Entamoeba histolytica HM-1:IMSS]	IINEPTAAAIAYGLDK	1659.89522	3	1.954
gi|56464189|gb|EAL42779.1| snoRNA binding protein, putative [Entamoeba histolytica HM-1:IMSS]	KIETKAGSK	961.5676864	2	1.5892
gi|56464202|gb|EAL42786.1| 40S ribosomal protein S6, putative [Entamoeba histolytica HM-1:IMSS]	IGQEVDASSLGDEFK	1594.759539	2	3.9102

gi|56464214|gb|EAL42795.1| Myb family DNA-binding protein [Entamoeba histolytica HM-1:IMSS]	FEEAFNK	884.414862	1	1.5931
	VIEAM*NAPR	1016.520441	1	1.6079
	VIEAM*NAPR	1016.520441	1	1.5663
gi|56464235|gb|EAL42810.1| DNA polymerase, putative [Entamoeba histolytica HM-1:IMSS]	KAYLDILTNEDGVEEYLIR	2254.160164	2	2.4655
gi|56464240|gb|EAL42814.1| coatomer protein gamma subunit, putative [Entamoeba histolytica HM-1:IMSS]	PLRSPYAHVELLK	1522.874037	3	1.9213
gi|56464326|gb|EAL42866.1| serine-rich protein [Entamoeba histolytica HM-1:IMSS]	DTNIYGVFLK	1169.620106	2	2.9365
gi|56464382|gb|EAL42899.1| Truncated pyridine nucleotide transhydrogenase [Entamoeba histolytica HM-1:IMSS]	LPGHMNVLLAEANVPYK	1865.994221	2	2.377
gi|56464491|gb|EAL42972.1| translation elongation factor EF-1 alpha, putative [Entamoeba histolytica HM-1:IMSS]	FEELLSK	865.466573	2	2.7465
	LPLQDVYK	975.550961	2	2.7768
	LPLQDVYK	975.550961	2	2.7288
	LPLQDVYK	975.550961	1	2.0289
	LPLQDVYK	975.550961	1	1.8933
	M*DAIQYK	884.41933	2	1.7275
	MDAIQYK	868.423317	2	1.9352
	QTVAVGVVK	900.5512974	2	2.207
	SM*EGGEPEYIK	1255.552212	2	3.5615
	STTTGHLIYK	1120.599715	2	2.7862
	YAWVLDNLK	1121.598974	2	3.1041
	YAWVLDNLK	1121.598974	2	3.0951
	YAWVLDNLK	1121.598974	1	2.9873
	YAWVLDNLK	1121.598974	1	2.9528
	YAWVLDNLK	1121.598974	2	2.8407
	YAWVLDNLK	1121.598974	2	2.79
	YAWVLDNLK	1121.598974	2	2.2537
	YYFTIIDAPGHR	1452.727031	2	3.2694
	YYFTIIDAPGHR	1452.727031	3	3.1847
	YYFTIIDAPGHR	1452.727031	2	2.563
	YYFTIIDAPGHR	1452.727031	2	2.0768
gi|56464512|gb|EAL42989.1| hypothetical protein 467.t00003 [Entamoeba histolytica HM-1:IMSS]	NVYGFNM*TPMRAEVLK	1885.931004	2	1.5846
gi|56464518|gb|EAL42993.1| Sec61 alpha subunit, putative [Entamoeba histolytica HM-1:IMSS]	DGESSVYLLDK	1225.594701	2	3.426
gi|56464539|gb|EAL43009.1| hypothetical protein 464.t00003 [Entamoeba histolytica HM-1:IMSS]	NTIIILNKVIIF	1400.887551	2	2.51
	NTIIILNKVIIF	1400.887551	2	2.3939
	GENTQTSGQDEKETAGGK	1836.820633	2	2.043
	LTKEIFNAIR	1204.704836	2	2.1366
gi|56464782|gb|EAL43193.1| BspA-like leucine rich repeat protein, putative [Entamoeba histolytica HM-1:IMSS]	SLGQSSISFKLRKSIK	1779.04875	2	1.5776
gi|56464783|gb|EAL43194.1| coatomer beta subunit, putative [Entamoeba histolytica HM-1:IMSS]	NVTYNALDANLILNELR	1946.034172	2	1.9185
gi|56464798|gb|EAL43207.1| protein phosphatase, putative [Entamoeba histolytica HM-1:IMSS]	KTCVVVLNQILNIFTNNK	2118.152511	2	1.902
gi|56464799|gb|EAL43208.1| 40S ribosomal protein S3a, putative [Entamoeba histolytica HM-1:IMSS]	ELTQFVDYLR	1283.663032	2	3.5427
	ELTQFVDYLR	1283.663032	2	3.3623
	ELTQFVDYLR	1283.663032	2	3.3361
	ELTQFVDYLR	1283.663032	2	2.9198
	ELTQFVDYLR	1283.663032	2	2.6445
	FGAYNYK	862.4093844	2	2.3026
	IAEPFSK	791.429793	2	1.8927
	IMNNLITK	946.539015	1	1.9392
	TTDGHLIR	912.4897604	2	2.4795
	TTDGHLIR	912.4897604	2	2.1927
	TTDGHLIR	912.4897604	2	1.6767
	TTDGHLIR	912.4897604	2	1.642
	TVGQIVVNK	957.5727614	2	2.5268
	VFSMDNVYIR	1243.613982	2	3.5506
	VFSMDNVYIR	1243.613982	2	3.3098

	VVEVNLADLMENEK	1602.804354	2	5.265
	VVEVNLADLMENEK	1602.804354	2	5.1391
	VVEVNLADLMENEK	1602.804354	2	4.5859
	VVEVNLADLMENEK	1602.804354	2	4.0699
	VVEVNLADLMENEK	1602.804354	2	2.7318
gi|56464800|gb|EAL43209.1| conserved hypothetical protein [Entamoeba histolytica HM-1:IMSS]	LLKFM*LVNK	1121.676212	2	1.8556
gi|56464807|gb|EAL43215.1| hypothetical protein 424.t00004 [Entamoeba histolytica HM-1:IMSS]	INVNTNQLEILKEK	1655.932666	3	2.1282
gi|56464865|gb|EAL43254.1| hypothetical protein 416.t00005 [Entamoeba histolytica HM-1:IMSS]	ELKDINNFEGAGAVGLAISCR	2234.101951	3	2.0424
gi|56464867|gb|EAL43256.1| hypothetical protein 416.t00007 [Entamoeba histolytica HM-1:IMSS]	AKTDVNKRQASIVVISGK	1914.113112	2	2.2856
gi|56464880|gb|EAL43267.1| hypothetical protein 414.t00002 [Entamoeba histolytica HM-1:IMSS]	KINIMKK	874.55427	2	2.0023
gi|56464909|gb|EAL43286.1| hypothetical protein 409.t00003 [Entamoeba histolytica HM-1:IMSS]	IM*LSGLNVKIENISKAK	1874.079069	2	1.5707
gi|56464916|gb|EAL43292.1| putative GTPase [Entamoeba histolytica HM-1:IMSS]	HIVLVLNK	935.603664	1	1.7528
	HIVLVLNK	935.603664	1	1.6724
gi|56464960|gb|EAL43328.1| hypothetical protein 403.t00003 [Entamoeba histolytica HM-1:IMSS]	KVEELK	745.445433	2	1.9313
gi|56464971|gb|EAL43337.1| hypothetical protein 402.t00005 [Entamoeba histolytica HM-1:IMSS]	TTQIQKEEK	1104.589533	1	1.7623
gi|56464992|gb|EAL43353.1| Phosphatidylinositol-4,5-bisphosphate 3-kinase catalytic subunit, putative [Entamoeba histolytica	DMAVQVM*FNVFNVLWM*K	2104.008635	2	1.7794
gi|56465001|gb|EAL43360.1| interaptin, putative [Entamoeba histolytica HM-1:IMSS]	NDVNALLGQINDYVK	1675.864983	2	2.0403
	NDVNALLGQINDYVK	1675.864983	2	1.7463
gi|56465056|gb|EAL43402.1| hypothetical protein 392.t00002 [Entamoeba histolytica HM-1:IMSS]	M*GIYGEVRNNFRVIESK	2028.034238	3	1.6705
gi|56465058|gb|EAL43404.1| 60S ribosomal protein L7, putative [Entamoeba histolytica HM-1:IMSS]	LVDPYVTYGYPTLETVQK	2086.074308	2	4.4125
gi|56465068|gb|EAL43412.1| hypothetical protein 390.t00005 [Entamoeba histolytica HM-1:IMSS]	TQIATIIIFKGYSK	1582.920321	3	1.7688
gi|56465099|gb|EAL43433.1| phospholipid-transporting P-type ATPase, putative [Entamoeba histolytica HM-1:IMSS]	EDAIKKAISIMEK	1475.813805	2	1.8189
	EDAIKKAISIMEK	1475.813805	2	1.6394
gi|56465108|gb|EAL43438.1| acyl-CoA synthetase, putative [Entamoeba histolytica HM-1:IMSS]	TGDIGEIVDGQIKIIDRK	1970.091694	2	2.3544
gi|56465117|gb|EAL43446.1| protein kinase, putative [Entamoeba histolytica HM-1:IMSS]	RISIKEVICSLQKILQSIR	2284.331902	2	1.6494
gi|56465139|gb|EAL43464.1| hypothetical protein 380.t00006 [Entamoeba histolytica HM-1:IMSS]	YEEDDNVMFFDTYPNDK	2141.864451	2	2.9283
gi|56465148|gb|EAL43471.1| conserved hypothetical protein [Entamoeba histolytica HM-1:IMSS]	ENSGTYNNQYIIDYNK	2048.955995	2	1.5545
gi|56465157|gb|EAL43477.1| glutamate dehydrogenase, putative [Entamoeba histolytica HM-1:IMSS]	DVLEKSGVLIFKDSSANK	1950.054271	3	1.8683
gi|56465163|gb|EAL43483.1| Sar family GTPase [Entamoeba histolytica HM-1:IMSS]	MLFLGLDNAGK	1178.623813	2	1.784
gi|56465185|gb|EAL43501.1| hypothetical protein 374.t00003 [Entamoeba histolytica HM-1:IMSS]	SEEEIEMLLTWASSLPSLNVER	2533.249094	2	3.7138
gi|56465191|gb|EAL43505.1| hypothetical protein 373.t00006 [Entamoeba histolytica HM-1:IMSS]	AISKTSNTVSKASKK	1549.890841	3	1.5864
gi|56465224|gb|EAL43531.1| uridine-cytidine kinase, putative [Entamoeba histolytica HM-1:IMSS]	ESALSDVE	849.383643	2	1.5687
gi|56465229|gb|EAL43536.1| Ras family GTPase [Entamoeba histolytica HM-1:IMSS]	DSILGLR	773.4515934	2	1.7164
	GDGFVLVYSIDNK	1426.721291	3	1.9606
gi|56465250|gb|EAL43552.1| hypothetical protein 366.t00005 [Entamoeba histolytica HM-1:IMSS]	KIHQNINNVYFKPMLVHK	2223.221927	3	1.7569
gi|56465254|gb|EAL43556.1| hypothetical protein 366.t00010 [Entamoeba histolytica HM-1:IMSS]	M*LVFETKK	1011.555429	2	1.6162
gi|56465305|gb|EAL43592.1| NAD(FAD)-dependent dehydrogenase, putative [Entamoeba histolytica HM-1:IMSS]	YMEIAKTK	983.523031	1	1.8575
gi|56465344|gb|EAL43619.1| metallo-beta-lactamase superfamily protein [Entamoeba histolytica HM-1:IMSS]	LM*HNLYTPRGAKVRNGK	1971.071615	2	1.9987
gi|56465372|gb|EAL43642.1| hypothetical protein 352.t00004 [Entamoeba histolytica HM-1:IMSS]	EYIIIIIEDSEGNKFGGYVNEK	2530.271187	3	1.9756
gi|56465388|gb|EAL43650.1| 40S ribosomal protein S19, putative [Entamoeba histolytica HM-1:IMSS]	DVVAIDFIK	1019.577176	1	2.5161
	ELAPLNPDWIYIR	1599.852958	2	2.8099
gi|56465397|gb|EAL43657.1| ARP2/3 complex 41 kDa subunit, putative [Entamoeba histolytica HM-1:IMSS]	VAWSPDSLTVAAASTDFK	1865.928006	2	2.0793
gi|56465409|gb|EAL43668.1| apyrase, putative [Entamoeba histolytica HM-1:IMSS]	GFDVVSNYLYK	1304.652145	2	2.7904
	GNEMSEIISFYDK	1532.693763	2	3.9325
	YVVEFDENFQNPR	1656.765267	2	3.1723
gi|56465413|gb|EAL43672.1| protein kinase, putative [Entamoeba histolytica HM-1:IMSS]	M*FKWENLTEEQTIDLK	2040.995773	3	1.9092
	NKIDGNDNK	1017.495969	2	1.815
gi|56465421|gb|EAL43679.1| 60S ribosomal protein L26, putative [Entamoeba histolytica HM-1:IMSS]	ANGQTVPVAIR	1125.637487	2	1.5956
	KVEPITEK	943.545876	2	1.8508
	YNVIAIPIHK	1167.688457	3	2.9053
gi|56465429|gb|EAL43686.1| exosome complex exonuclease, putative [Entamoeba histolytica HM-1:IMSS]	RIDAGALMLASSEIKIEK	1945.0787	2	1.8289
gi|56465438|gb|EAL43694.1| conserved hypothetical protein [Entamoeba histolytica HM-1:IMSS]	DAVNKLAELQENHK	1608.834015	2	1.7487

gi|56465529|gb|EAL43773.1| enolase, putative [Entamoeba histolytica HM-1:IMSS]	AVENVNTIIGPALLGK	1608.931942	2	3.1697
gi|56465539|gb|EAL43782.1| DEAD/DEAH box helicase, putative [Entamoeba histolytica HM-1:IMSS]	ENELEADIK	1060.5157	2	1.6587
gi|56465545|gb|EAL43788.1| conserved hypothetical protein [Entamoeba histolytica HM-1:IMSS]	SSSLNTNK	850.42652	2	2.4232
	VFRIIDLTKENK	1475.858043	2	2.2723
gi|56465564|gb|EAL43802.1| hypothetical protein 334.t00003 [Entamoeba histolytica HM-1:IMSS]	EFYVFSSQVISNPLLTK	1972.042641	2	1.5656
gi|56465573|gb|EAL43810.1| Rab family GTPase [Entamoeba histolytica HM-1:IMSS] gi|13537439|dbj|BAB40674.1| small G	ATIGADFLTK	1036.567342	2	2.1148
gi|56465607|gb|EAL43834.1| hypothetical protein 330.t00001 [Entamoeba histolytica HM-1:IMSS]	DSTRVEARFAEEK	1537.760526	2	1.9399
gi|56465624|gb|EAL43848.1| hypothetical protein 328.t00002 [Entamoeba histolytica HM-1:IMSS]	AISAAVENGVAR	1157.627327	2	2.1693
	HVGLEDEGNFVDER	1615.734701	2	4.8677
	HVGLEDEGNFVDER	1615.734701	3	3.3499
	VAQNIVK	771.472316	1	1.5133
gi|56465626|gb|EAL43850.1| aldehyde-alcohol dehydrogenase 2, putative [Entamoeba histolytica HM-1:IMSS]	GLAVMNTFGPDNIIAIGGGSAMDAAK	2491.231984	2	4.2321
gi|56465632|gb|EAL43856.1| hypothetical protein 328.t00010 [Entamoeba histolytica HM-1:IMSS]	NQLNCTNPFDNGPTMSVK	2036.894989	3	1.5488
gi|56465640|gb|EAL43863.1| syntaxin, putative [Entamoeba histolytica HM-1:IMSS]	QLNMLSESEEMVDQR	1808.81535	2	2.5437
gi|56465658|gb|EAL43878.1| hypothetical protein 326.t00008 [Entamoeba histolytica HM-1:IMSS]	HNGIENEEKIKDELM*NVK	2156.066315	2	1.9809
gi|56465661|gb|EAL43880.1| 40S ribosomal protein S24, putative [Entamoeba histolytica HM-1:IMSS]	TSGFALIYDTLSALK	1599.862877	2	3.8073
gi|56465678|gb|EAL43895.1| transcription initiation factor TFIID, putative [Entamoeba histolytica HM-1:IMSS]	VTLLVFSTGK	1064.635037	2	1.7902
gi|56465691|gb|EAL43905.1| beta adaptin, putative [Entamoeba histolytica HM-1:IMSS]	MSKKGNMK	923.4801314	2	1.5324
gi|56465760|gb|EAL43956.1| hypothetical protein 315.t00005 [Entamoeba histolytica HM-1:IMSS]	EEPIGGIVINNVINNK	1722.938485	2	1.6911
gi|56465763|gb|EAL43959.1| P-glycoprotein 5 [Entamoeba histolytica HM-1:IMSS]	KENRVGSLVTRLSSDPTK	1987.093116	2	1.5012
gi|56465788|gb|EAL43980.1| Rab family GTPase [Entamoeba histolytica HM-1:IMSS] gi|56790094|dbj|BAD82843.1| EhRabX	QVSPEDAKDFAK	1334.658686	2	1.5147
gi|56465790|gb|EAL43981.1| 60S ribosomal protein L9, putative [Entamoeba histolytica HM-1:IMSS]	HIEMYDGVVVK	1289.655839	2	3.7287
	NMITGVTK	863.4655184	1	2.3099
	NMITGVTK	863.4655184	1	1.9692
	SIQVQNFLGEK	1262.673943	2	3.3018
	TIASHIK	769.456676	2	1.806
	VDIYASK	795.424708	1	1.8098
	VDIYASK	795.424708	2	1.5975
	VSQSAANIHAANLVR	1550.839788	2	3.9011
	VSQSAANIHAANLVR	1550.839788	3	2.8959
	VVYAHFPVTLVIADDGK	1843.995268	2	4.1482
	VVYAHFPVTLVIADDGK	1843.995268	2	3.4883
gi|56465795|gb|EAL43986.1| hypothetical protein 312.t00006 [Entamoeba histolytica HM-1:IMSS]	TIIDSIRSGR	1117.632422	2	1.8457
gi|56465811|gb|EAL43996.1| RNA polymerase I largest subunit, putative [Entamoeba histolytica HM-1:IMSS]	FGDLISDHPQSIYYR	1810.875902	2	1.833
gi|56465853|gb|EAL44029.1| hypothetical protein 306.t00004 [Entamoeba histolytica HM-1:IMSS]	ASNCSTKSFPNAK	1411.642009	2	1.7329
gi|56465915|gb|EAL44079.1| hypothetical protein 299.t00002 [Entamoeba histolytica HM-1:IMSS]	ITKM*EM*KAMK	1242.627659	2	1.8936
gi|56465916|gb|EAL44080.1| hypothetical protein 299.t00004 [Entamoeba histolytica HM-1:IMSS]	DQFKANSSFVVK	1369.711065	2	1.5368
gi|56465919|gb|EAL44083.1| hypothetical protein 299.t00007 [Entamoeba histolytica HM-1:IMSS]	NVDFITSTTSLLK	1438.778811	2	3.2671
gi|56465920|gb|EAL44084.1| Ribosome biogenesis protein BMS1, putative [Entamoeba histolytica HM-1:IMSS]	FYNPMTDLTGYEWDR	1907.826883	2	3.3898
	LFNLSGITHGSYPK	1533.806033	2	3.4357
	LVPSDIVFLK	1130.681985	1	1.9889
	VEDLTEPQDIAEDPDVDR	2055.935309	2	3.4758
gi|56465927|gb|EAL44090.1| hypothetical protein 298.t00006 [Entamoeba histolytica HM-1:IMSS]	QDEQPNETNESDK	1533.629968	2	1.8718
gi|56465934|gb|EAL44097.1| glycerophosphoryl diester phosphodiesterase, putative [Entamoeba histolytica HM-1:IMSS]	GGVYYYPENTIEAMK	1734.804359	2	1.647
gi|56465941|gb|EAL44103.1| DNA repair helicase, putative [Entamoeba histolytica HM-1:IMSS]	PLENEYK	892.441077	2	1.8777
gi|56465959|gb|EAL44117.1| Clathrin coat assembly protein, putative [Entamoeba histolytica HM-1:IMSS]	ALFSELQYGENVR	1525.76455	2	3.403
gi|56465989|gb|EAL44138.1| hypothetical protein 292.t00006 [Entamoeba histolytica HM-1:IMSS]	AQSNQTIFTAVTDKSEQKIK	2237.177229	3	1.5752
gi|56465993|gb|EAL44142.1| hypothetical protein 292.t00011 [Entamoeba histolytica HM-1:IMSS]	CKDILKETDDNICWNCK	2211.918748	3	2.7255
gi|56466009|gb|EAL44155.1| hypothetical protein 291.t00005 [Entamoeba histolytica HM-1:IMSS]	PSSLNCNNLR	1174.54189	2	1.6843
gi|56466015|gb|EAL44160.1| hypothetical protein 290.t00010 [Entamoeba histolytica HM-1:IMSS]	EIPDYIGYFGLDEDDNK	2002.891657	2	4.079
	EIPDYIGYFGLDEDDNK	2002.891657	2	3.8307
	EIPDYIGYFGLDEDDNK	2002.891657	2	3.247

gi|56466019|gb|EAL44164.1| Truncated, galactose-specific adhesin 170kD subunit [Entamoeba histolytica HM-1:IMSS]	FVQLTDDPSLDSEHFR	1905.897759	2	5.0232
	FVQLTDDPSLDSEHFR	1905.897759	2	4.7251
	IQEIVITEK	1072.624855	2	2.5725
	IQEIVITEK	1072.624855	1	2.5654
	LHLPQSEEFMK	1358.677311	3	2.9747
	LHLPQSEEFMK	1358.677311	3	2.3567
	LHLPQSEEFMK	1358.677311	2	1.7885
gi|56466022|gb|EAL44166.1| hypothetical protein 290.t00008 [Entamoeba histolytica HM-1:IMSS]	NKSNNNTQRR	1231.625034	2	1.6347
gi|56466047|gb|EAL44188.1| conserved hypothetical protein [Entamoeba histolytica HM-1:IMSS]	SLESATFK	882.456747	2	1.6812
gi|56466051|gb|EAL44191.1| 40S ribosomal protein S25, putative [Entamoeba histolytica HM-1:IMSS]	LNNAVLFDK	1033.567674	2	1.5828
	VITPAVVADR	1040.609873	2	1.8194
gi|56466055|gb|EAL44195.1| protein farnesyltransferase alpha subunit, putative [Entamoeba histolytica HM-1:IMSS]	M*EEDEEITFDSTK	1589.65344	2	1.845
	SLNLLNDLNQLK	1384.779469	2	1.7548
	SLNLLNDLNQLK	1384.779469	2	1.6682
gi|56466059|gb|EAL44199.1| hypothetical protein 288.t00011 [Entamoeba histolytica HM-1:IMSS]	RWIGLKNKIMM*LFK	1794.029199	2	2.1765
	RWIGLKNKIMM*LFK	1794.029199	2	2.033
gi|56466076|gb|EAL44212.1| hypothetical protein 286.t00003 [Entamoeba histolytica HM-1:IMSS]	EGNIEDVVIEK	1244.63688	2	1.7138
gi|56466079|gb|EAL44215.1| hypothetical protein 286.t00007 [Entamoeba histolytica HM-1:IMSS]	PELKKIDPK	1067.645924	2	1.9115
gi|56466083|gb|EAL44219.1| hypothetical protein 286.t00011 [Entamoeba histolytica HM-1:IMSS]	LTLPSGATISGTGSLSVMNLEIK	2289.237078	3	1.7959
gi|56466088|gb|EAL44223.1| Rab family GTPase [Entamoeba histolytica HM-1:IMSS]	LVIIGESGVGK	1071.640856	2	2.8477
gi|56466092|gb|EAL44227.1| 40S ribosomal protein S16, putative [Entamoeba histolytica HM-1:IMSS]	VFEPLFIVGK	1148.671412	2	2.6568
	VFEPLFIVGK	1148.671412	2	2.2198
gi|56466106|gb|EAL44239.1| hypothetical protein 284.t00011 [Entamoeba histolytica HM-1:IMSS]	IHIERSLK	995.599652	2	1.6666
gi|56466117|gb|EAL44249.1| hypothetical protein 283.t00007 [Entamoeba histolytica HM-1:IMSS]	ETGLDIINM*ERLNLK	1774.937865	2	1.7115
gi|56466127|gb|EAL44258.1| Ras guanine nucleotide exchange factor, putative [Entamoeba histolytica HM-1:IMSS]	EAFDLSLK	922.488037	2	1.6872
gi|56466131|gb|EAL44262.1| beta-adaptin, putative [Entamoeba histolytica HM-1:IMSS]	NVLDFDEDTSKRNDSMK	2013.918237	2	1.6219
gi|56466152|gb|EAL44279.1| 60S ribosomal protein L13, putative [Entamoeba histolytica HM-1:IMSS]	FIAEHNK	858.44683	2	1.8058
	KFIAEHNK	986.541793	2	2.3694
	LPVQQK	712.435202	1	1.9473
	TIGIAVDPR	941.5414614	2	1.6837
gi|56466155|gb|EAL44282.1| hypothetical protein 280.t00011 [Entamoeba histolytica HM-1:IMSS]	M*EKIASVVEENAK	1463.742133	2	2.0326
gi|56466157|gb|EAL44284.1| hypothetical protein 280.t00013 [Entamoeba histolytica HM-1:IMSS]	VHKYNEIK	1030.568008	2	1.5015
gi|56466194|gb|EAL44317.1| Activator 1 140 kDa subunit, putative [Entamoeba histolytica HM-1:IMSS]	GYAAIPMMYIR	1285.643164	3	2.0548
gi|56466201|gb|EAL44324.1| 40S ribosomal protein S26, putative [Entamoeba histolytica HM-1:IMSS]	DISDNSALTK	1063.526619	2	2.8128
	DISDNSALTK	1063.526619	1	1.9944
	NIVEAAAVR	942.536708	2	3.1343
	NIVEAAAVR	942.536708	1	1.9769
	VEIPADVVTAPK	1238.699083	2	2.1974
	VEIPADVVTAPK	1238.699083	2	1.7312
gi|56466204|gb|EAL44326.1| acyl-CoA synthetase, putative [Entamoeba histolytica HM-1:IMSS]	AVIAELSGGKPFEEAIEDK	2003.033185	2	1.9334
	DPTVASIGVVLPMYEVK	1817.971765	2	2.1802
gi|56466211|gb|EAL44333.1| hypothetical protein 276.t00010 [Entamoeba histolytica HM-1:IMSS]	LDLKQQQILDSITKLNK	1998.159382	2	2.0734
	LDLKQQQILDSITKLNK	1998.159382	3	2.0547
	LDLKQQQILDSITKLNK	1998.159382	2	1.9534
	LDLKQQQILDSITKLNK	1998.159382	2	1.8744
gi|56466218|gb|EAL44339.1| hypothetical protein 275.t00020 [Entamoeba histolytica HM-1:IMSS]	KIVFICPDTDLNLLQLK	2030.114	2	1.5871
gi|56466279|gb|EAL44392.1| hypothetical protein 272.t00015 [Entamoeba histolytica HM-1:IMSS]	LFEFGLGDICVYKENNK	2045.978635	2	2.1169
gi|56466303|gb|EAL44411.1| Beige/BEACH domain protein, putative [Entamoeba histolytica HM-1:IMSS]	IYNKLLILCNLPDESIELK	2288.235583	3	1.8923
gi|56466320|gb|EAL44426.1| hypothetical protein 268.t00003 [Entamoeba histolytica HM-1:IMSS]	NLPHVLKQYTPFK	1584.889677	2	1.667
gi|56466428|gb|EAL44516.1| hypothetical protein 261.t00001 [Entamoeba histolytica HM-1:IMSS]	GFSVIKDKMQLYLIKNK	2025.156545	2	1.7068
gi|56466438|gb|EAL44526.1| Ser/Thr protein phosphatase, putative [Entamoeba histolytica HM-1:IMSS]	GRMSTEIIITLFCMK	1799.900194	2	1.8138
gi|56466451|gb|EAL44538.1| protein kinase, putative [Entamoeba histolytica HM-1:IMSS]	EIM*IEEDEYLKK	1555.757105	2	1.6069

gi|56466487|gb|EAL44567.1| hypothetical protein 257.t00010 [Entamoeba histolytica HM-1:IMSS]	GWEEEDIQAIEIEIKSR	2045.018595	2	1.9492
gi|56466506|gb|EAL44584.1| 90 kDa heat shock protein, putative [Entamoeba histolytica HM-1:IMSS]	SDVTDEEYNQFYK	1637.69659	2	1.6617
gi|56466523|gb|EAL44598.1| chromodomain-helicase-DNA-binding protein, putative [Entamoeba histolytica HM-1:IMSS]	GCVGSNVPNLQNLMMQLR	2030.9718	2	1.8272
gi|56466525|gb|EAL44600.1| hypothetical protein 254.t00009 [Entamoeba histolytica HM-1:IMSS]	ISRMFTFEK	1158.597603	1	1.6172
gi|56466528|gb|EAL44603.1| centromere/microtubule binding protein cbf5, putative [Entamoeba histolytica HM-1:IMSS]	GEAIALGIAK	942.5618648	1	1.7119
gi|56466556|gb|EAL44624.1| 60S acidic ribosomal protein P0, putative [Entamoeba histolytica HM-1:IMSS]	ESGVVFPLAEK	1175.630681	2	2.2902
gi|56466622|gb|EAL44683.1| hypothetical protein 249.t00015 [Entamoeba histolytica HM-1:IMSS]	GHIDMSQVTDMITTR	1704.804382	2	4.4668
	GHIDMSQVTDMITTR	1704.804382	2	4.2956
gi|56466628|gb|EAL44688.1| hypothetical protein 248.t00004 [Entamoeba histolytica HM-1:IMSS]	LM*IGSIYQELSYSIDIIK	2102.110106	2	2.442
gi|56466629|gb|EAL44689.1| 60S ribosomal protein L7a, putative [Entamoeba histolytica HM-1:IMSS]	STAAVVAVTEIK	1188.683443	2	1.7551
gi|56466648|gb|EAL44707.1| importin alpha re-exporter, putative [Entamoeba histolytica HM-1:IMSS]	SILPVTNQEYNEIFIK	1908.011321	2	1.7882
gi|56466731|gb|EAL44771.1| fatty acid elongase, putative [Entamoeba histolytica HM-1:IMSS]	AIIDGLQENLK	1213.678684	2	3.4051
	AIIDGLQENLK	1213.678684	2	2.866
	EIGEQNEILPSLK	1469.784617	2	3.204
	FELLTTVR	978.56186	2	2.6225
	FELLTTVR	978.56186	2	2.2952
	GM*LISNTLFRMGGAAVLLSNK	2209.184284	2	1.5078
	TGLGNETYLPR	1220.626986	2	2.8759
	VWQVAFGSGLK	1191.652087	2	3.1955
gi|56466826|gb|EAL44846.1| replication factor A protein 1, putative [Entamoeba histolytica HM-1:IMSS]	DMENGVVYVLTSLKLKEFK	2213.188635	2	2.5931
gi|56466831|gb|EAL44851.1| hypothetical protein 234.t00009 [Entamoeba histolytica HM-1:IMSS]	EEVKEDIKEK	1246.652528	2	1.5408
	KEDISVPK	915.514586	2	1.5318
gi|56466862|gb|EAL44879.1| conserved hypothetical protein [Entamoeba histolytica HM-1:IMSS]	IM*DM*ALNNFRVTMCEVR	2131.956282	2	1.5598
gi|56466879|gb|EAL44894.1| conserved hypothetical protein [Entamoeba histolytica HM-1:IMSS]	VVPIQCFFCGSTEHLAK	1992.924102	3	1.8243
gi|56466937|gb|EAL44941.1| 60S ribosomal protein L35a, putative [Entamoeba histolytica HM-1:IMSS]	VFLYPSNI	952.513857	1	1.9142
	VFLYPSNI	952.513857	1	1.5846
gi|56466973|gb|EAL44971.1| Grainin 1 [Entamoeba histolytica HM-1:IMSS]	ELQDFFVK	1025.530226	2	2.4672
	ELQDFFVK	1025.530226	2	2.2919
	ELQDFFVK	1025.530226	1	2.1707
	ELQDFFVK	1025.530226	2	2.0617
	ELQDFFVK	1025.530226	1	1.922
	FPGGIK	618.3609788	1	1.5728
	FPGGIK	618.3609788	2	1.5377
	LATAVFTK	850.503282	1	1.7267
	LATAVFTK	850.503282	2	1.7189
	LATAVFTK	850.503282	1	1.5095
	SGSLELDELK	1090.562672	2	3.0187
	SGSLELDELKK	1218.657635	2	3.4745
	TIYQHGFK	993.5152464	2	2.651
gi|56466988|gb|EAL44985.1| grainin 2 [Entamoeba histolytica HM-1:IMSS]	ALPMLGFNCNK	1264.596214	2	1.7441
	SGSLDVNELIK	1174.63142	2	3.5529
	SGSLDVNELIK	1174.63142	2	3.2591
	SGSLDVNELIK	1174.63142	2	2.9009
	SGSLDVNELIK	1174.63142	2	2.4484
	SGSLDVNELIK	1174.63142	1	2.0013
	SGTLEIGELK	1046.572835	2	2.9673
	SGTLEIGELK	1046.572835	2	2.4127
	SGTLEIGELK	1046.572835	1	1.6747
	SVDVLLK	773.476743	1	1.8722
	SVDVLLK	773.476743	2	1.8035
	SVDVLLK	773.476743	1	1.7704
gi|56466991|gb|EAL44987.1| fructose-1,6-bisphosphate aldolase, putative [Entamoeba histolytica HM-1:IMSS]	LESAIGIPEDQIR	1440.769301	2	1.6622

gi|56466992|gb|EAL44988.1| integral membrane protein, putative [Entamoeba histolytica HM-1:IMSS]	MLSNAIFK	923.501911	2	1.668
gi|56467000|gb|EAL44996.1| lipid phosphatase, putative [Entamoeba histolytica HM-1:IMSS]	PYGEGLWAPVPEK	1442.731451	2	1.7245
gi|56467001|gb|EAL44997.1| hypothetical protein 223.t00011 [Entamoeba histolytica HM-1:IMSS]	AVEQALAEK	958.52039	1	1.5536
	DIQIEELNK	1101.578634	2	3.0215
	EELVQQLMK	1117.592174	2	2.5417
	EELVQQLMK	1117.592174	2	2.1998
	EKDIQIEELNK	1358.716191	3	2.367
	EQISSAEENSNSIIENEK	2020.930598	2	4.631
	EVVIVSLQK	1014.619385	2	2.0917
	EVVIVSLQK	1014.619385	1	1.7473
	EVVIVSLQK	1014.619385	1	1.7262
	GLQLKFFFSIM*EAEM*SDIK	2266.115627	3	1.5906
	GNNEIIELDHLK	1394.727426	2	3.7181
	GNNEIIELDHLK	1394.727426	3	2.9152
	IEKDDAPFPSALDLIE	1772.895289	2	3.9872
	IEKDDAPFPSALDLIE	1772.895289	2	3.6397
	IEKDDAPFPSALDLIE	1772.895289	2	3.3802
	NAAAGSEELLK	1102.573895	2	2.9203
	NAAAGSEELLK	1102.573895	1	2.1494
	SDNLNHQSIDQSILSK	1798.893028	3	4.939
	SDNLNHQSIDQSILSK	1798.893028	2	4.4275
	SDNLNHQSIDQSILSK	1798.893028	2	4.1876
	VIVASTMK	848.491012	2	2.2656
	VIVASTMK	848.491012	1	1.7309
	YENNIPSLANEIK	1504.764214	2	2.8121
	YLSDILNK	965.530236	1	2.5622
	YLSDILNK	965.530236	2	2.1702
	YLSDILNK	965.530236	2	2.068
	YLSDILNK	965.530236	1	1.9068
gi|56467051|gb|EAL45040.1| conserved hypothetical protein [Entamoeba histolytica HM-1:IMSS]	PELLKDMLSNIAKIEYMK	2136.144324	3	1.8877
gi|56467061|gb|EAL45048.1| hypothetical protein 219.t00002 [Entamoeba histolytica HM-1:IMSS]	NM*SCIVMK	998.426414	1	1.6256
gi|56467094|gb|EAL45079.1| conserved hypothetical protein [Entamoeba histolytica HM-1:IMSS]	M*NENEVK	879.388759	2	1.7778
gi|56467115|gb|EAL45097.1| phosphatidylinositol 3-kinase, putative [Entamoeba histolytica HM-1:IMSS]	LM*LVLEAGEFEK	1394.724683	2	1.975
gi|56467136|gb|EAL45116.1| protein kinase, putative [Entamoeba histolytica HM-1:IMSS]	FTKGHKIDLNVFK	1546.874029	2	1.5333
gi|56467151|gb|EAL45130.1| hypothetical protein 214.t00007 [Entamoeba histolytica HM-1:IMSS]	EINEQNK	874.42649	2	2.0876
gi|56467201|gb|EAL45176.1| ruvB-like DNA helicase, putative [Entamoeba histolytica HM-1:IMSS]	TAM*GIVLRMVQSR	1477.798876	2	2.1272
gi|56467225|gb|EAL45198.1| protein kinase, putative [Entamoeba histolytica HM-1:IMSS]	AGSMTITCSGTIK	1326.617762	3	1.5725
gi|56467244|gb|EAL45215.1| hypothetical protein 209.t00003 [Entamoeba histolytica HM-1:IMSS]	FSTEFEYGGSK	1251.552839	2	2.7323
	IM*LQGNEK	948.4829954	2	1.7255
	NIYFAMK	886.449137	1	1.8104
	SYDKEPYVILPNK	1565.821	3	3.5969
	SYDKEPYVILPNK	1565.821	2	1.8834
	VLNFVIYTPAK	1264.729988	2	4.0387
gi|56467259|gb|EAL45229.1| hypothetical protein 208.t00006 [Entamoeba histolytica HM-1:IMSS]	VGIMVGGYK	923.5019082	2	1.5032
gi|56467263|gb|EAL45233.1| hypothetical protein 208.t00010 [Entamoeba histolytica HM-1:IMSS]	ENIQFM*KK	1053.540842	2	1.8307
gi|56467289|gb|EAL45256.1| cysteine proteinase 2 precursor [Entamoeba histolytica HM-1:IMSS]	YLNIQAPESVDWR	1590.791097	2	2.7399
gi|56467297|gb|EAL45264.1| protein disulfide isomerase, putative [Entamoeba histolytica HM-1:IMSS]	FGISGFPTLK	1066.593175	2	2.9245
	FGISGFPTLK	1066.593175	2	2.7062
	IAELDDLAK	987.535706	2	2.6027
	IAELDDLAK	987.535706	1	1.866
	NLNVLEVF	947.519661	2	2.4719
	NLNVLEVF	947.519661	1	1.839

	NLNVLEVF	947.519661	1	1.7603
gi|56467298|gb|EAL45265.1| valyl-tRNA synthetase, putative [Entamoeba histolytica HM-1:IMSS]	QTKEKEEIKFNK	1521.827138	2	2.017
gi|56467345|gb|EAL45308.1| conserved hypothetical protein [Entamoeba histolytica HM-1:IMSS]	EQNNQIEGKEKKIK	1685.918081	2	2.2711
gi|56467361|gb|EAL45324.1| protein kinase, putative [Entamoeba histolytica HM-1:IMSS]	IGFDDFSLVK	1140.593567	2	1.6076
gi|56467403|gb|EAL45364.1| hypothetical protein 201.t00014 [Entamoeba histolytica HM-1:IMSS]	IPYLGQDGYFLK	1413.741287	2	2.5077
gi|56467411|gb|EAL45372.1| hypothetical protein 201.t00024 [Entamoeba histolytica HM-1:IMSS]	ITKVSR	703.446111	2	1.5785
gi|56467460|gb|EAL45418.1| protein kinase, putative [Entamoeba histolytica HM-1:IMSS]	RWNSQDLFNAVK	1477.754651	2	1.7138
gi|56467467|gb|EAL45425.1| hypothetical protein 198.t00015 [Entamoeba histolytica HM-1:IMSS]	KKGDITK	789.4828834	1	1.658
gi|56467488|gb|EAL45445.1| Rho family GTPase [Entamoeba histolytica HM-1:IMSS]	LVFDEAVR	948.51491	2	1.8504
	LVFDEAVR	948.51491	2	1.5494
	PINLGLWDTAGQEDYDR	1962.919208	2	4.9647
	PINLGLWDTAGQEDYDR	1962.919208	2	4.5965
gi|56467490|gb|EAL45447.1| hsp70 family protein [Entamoeba histolytica HM-1:IMSS]	LEDGMIGM*KVMM*RGEQK	1984.934491	3	1.581
gi|56467522|gb|EAL45475.1| 60S ribosomal protein L24, putative [Entamoeba histolytica HM-1:IMSS]	VFWTAIYR	1055.567279	2	3.1715
	VFWTAIYR	1055.567279	1	2.0801
	VFWTAIYR	1055.567279	1	1.8459
gi|56467545|gb|EAL45496.1| pumilio family RNA-binding protein [Entamoeba histolytica HM-1:IMSS]	SRPIQIDVNNNVSKNKTR	2083.1367	2	1.5526
gi|56467577|gb|EAL45523.1| cdc48-like protein [Entamoeba histolytica HM-1:IMSS]	ILNQLLTEMDGVGK	1530.819614	2	1.9215
gi|56467595|gb|EAL45540.1| coactosin, putative [Entamoeba histolytica HM-1:IMSS]	LVVDAK	644.397754	1	1.6952
	TISGDEESKR	1121.543334	3	1.7125
gi|56467604|gb|EAL45549.1| phospholipid-transporting P-type ATPase, putative [Entamoeba histolytica HM-1:IMSS]	EFIQFALK	995.556046	2	1.6458
gi|56467606|gb|EAL45551.1| cysteine proteinase, putative [Entamoeba histolytica HM-1:IMSS]	AAAEGPVAAAIDASGVK	1497.790768	2	2.8325
gi|56467621|gb|EAL45564.1| hypothetical protein 190.t00004 [Entamoeba histolytica HM-1:IMSS]	YHLMM*IKLIILK	1531.911373	2	1.5334
gi|56467641|gb|EAL45583.1| myotubularin, putative [Entamoeba histolytica HM-1:IMSS]	NLIVPVK	782.513452	1	1.8583
	SVCDIIATEINDIRFGIR	2092.064103	2	2.8152
	SVCDIIATEINDIRFGIR	2092.064103	2	2.4368
gi|56467648|gb|EAL45590.1| hypothetical protein 189.t00012 [Entamoeba histolytica HM-1:IMSS]	AEDNYSDEM*IINLTSVFYK	2268.038782	3	1.5671
gi|56467670|gb|EAL45607.1| HMG box protein [Entamoeba histolytica HM-1:IMSS]	KSKKVTK	818.545825	2	1.5698
gi|56467700|gb|EAL45633.1| glycogen phosphorylase, putative [Entamoeba histolytica HM-1:IMSS]	MYYLSIEYLIGR	1520.781778	2	1.678
gi|56467718|gb|EAL45650.1| hypothetical protein 186.t00016 [Entamoeba histolytica HM-1:IMSS]	NYKILM*EITEVLK	1609.888058	3	1.6139
gi|56467748|gb|EAL45679.1| hypothetical protein 185.t00019 [Entamoeba histolytica HM-1:IMSS]	VDKENEKQQEESNK	1704.803515	2	1.7099
gi|56467754|gb|EAL45684.1| Ras family GTPase [Entamoeba histolytica HM-1:IMSS]	FVQGIFLVK	1050.634632	1	1.7369
	IVVLGSGAVGK	999.6197262	2	3.5679
	IVVLGSGAVGK	999.6197262	1	1.7344
gi|56467796|gb|EAL45721.1| hypothetical protein 182.t00030 [Entamoeba histolytica HM-1:IMSS]	LLNAEGIEIPWEIM*TR	1900.984815	3	1.8747
gi|56467813|gb|EAL45737.1| hypothetical protein 182.t00023 [Entamoeba histolytica HM-1:IMSS]	EIEQETIIR	1130.605183	2	1.7201
gi|56467855|gb|EAL45770.1| hypothetical protein 180.t00017 [Entamoeba histolytica HM-1:IMSS]	SVVLYCPITCVFDLLFVNK	2287.143595	2	1.829
gi|56467863|gb|EAL45776.1| 60S ribosomal protein L31, putative [Entamoeba histolytica HM-1:IMSS]	FDMGLNQFIWSQGIR	1811.889769	2	4.5689
	FDMGLNQFIWSQGIR	1811.889769	2	3.2877
	FYTLVSYVPVASFK	1620.867231	2	4.7777
	FYTLVSYVPVASFK	1620.867231	2	2.9787
	FYTLVSYVPVASFK	1620.867231	3	2.7288
gi|56467864|gb|EAL45777.1| Ras family GTPase [Entamoeba histolytica HM-1:IMSS]	DTENVPIIVVGNK	1397.763477	2	2.108
gi|56467868|gb|EAL45781.1| hypothetical protein 179.t00013 [Entamoeba histolytica HM-1:IMSS]	YKPTLPAR	945.551629	2	1.5017
gi|56467871|gb|EAL45783.1| conserved hypothetical protein [Entamoeba histolytica HM-1:IMSS]	QM*SRIIEECNNDEK	1781.758923	2	1.6844
gi|56467875|gb|EAL45787.1| hypothetical protein 179.t00006 [Entamoeba histolytica HM-1:IMSS]	ISDQCIQFLR	1279.624881	2	1.5047
gi|56467949|gb|EAL45851.1| hypothetical protein 175.t00023 [Entamoeba histolytica HM-1:IMSS]	TVIPEVQAEQLLR	1495.847873	2	1.8238
gi|56467988|gb|EAL45883.1| 60S ribosomal protein L13, putative [Entamoeba histolytica HM-1:IMSS]	SSIPEAAAIYSFNPTR	1723.865011	2	1.588
gi|56468005|gb|EAL45900.1| 60S ribosomal protein L38, putative [Entamoeba histolytica HM-1:IMSS]	DIHVVEIAK	1023.583324	3	2.8545
	DIHVVEIAK	1023.583324	1	2.7758
	DIHVVEIAK	1023.583324	2	2.4467
	DIHVVEIAK	1023.583324	2	2.2498

	DIHVVEIAK	1023.583324	2	1.8309
gi|56468015|gb|EAL45908.1| kinesin-like protein [Entamoeba histolytica HM-1:IMSS]	IQELLETIQSLNK	1528.858114	2	1.762
	QM*NANAQNK	1034.469469	2	1.5846
gi|56468020|gb|EAL45913.1| uridine-cytidine kinase, putative [Entamoeba histolytica HM-1:IMSS]	TTFCQEIANTLK	1425.682781	3	1.6365
gi|56468029|gb|EAL45920.1| conserved hypothetical protein [Entamoeba histolytica HM-1:IMSS]	FDNRITQVKDK	1363.732843	3	1.5736
gi|56468106|gb|EAL45992.1| galactose-inhibitable lectin 35 kda subunit precursor, putative [Entamoeba histolytica HM-1:IMSS	QIFIPVFA	934.539667	1	1.5164
	VIFASDYNYK	1219.599379	2	2.7868
gi|56468237|gb|EAL46108.1| ABC transporter, putative [Entamoeba histolytica HM-1:IMSS]	ILVDGIDITK	1086.640507	2	2.6038
gi|56468238|gb|EAL46109.1| hypothetical protein 162.t00009 [Entamoeba histolytica HM-1:IMSS]	KEVQTMFDLRGM*KTEPFK	2201.110426	3	1.7947
gi|56468243|gb|EAL46114.1| hypothetical protein 162.t00014 [Entamoeba histolytica HM-1:IMSS]	IYNLVNDDGDYVITTPK	1939.964758	2	1.5364
gi|56468274|gb|EAL46140.1| hypothetical protein 160.t00017 [Entamoeba histolytica HM-1:IMSS]	KRKIEATELMNEVANAK	1945.053526	2	1.8864
gi|56468289|gb|EAL46152.1| methionine aminopeptidase, putative [Entamoeba histolytica HM-1:IMSS]	LLHALDANFK	1141.636422	3	4.1323
	LLHALDANFK	1141.636422	3	3.3643
gi|56468300|gb|EAL46163.1| protein kinase, putative [Entamoeba histolytica HM-1:IMSS]	KYYLHVFGQYGLVPTTDLK	2242.190677	3	1.5675
gi|56468363|gb|EAL46218.1| BspA-like leucine rich repeat protein, putative [Entamoeba histolytica HM-1:IMSS]	IFM*NEPVLISIKIPKK	1886.119464	2	1.7808
gi|56468399|gb|EAL46246.1| hypothetical protein 154.t00004 [Entamoeba histolytica HM-1:IMSS]	KGGVAK	559.3562278	1	1.6848
gi|56468426|gb|EAL46272.1| hypothetical protein 153.t00006 [Entamoeba histolytica HM-1:IMSS]	GIFINYVNK	1067.588411	2	2.0614
	TIVLNFEK	963.550961	2	2.4148
	TIVLNFEK	963.550961	2	1.7838
	TIVLNFEK	963.550961	1	1.7737
	VVTQLPK	784.492717	1	1.5472
gi|56468427|gb|EAL46273.1| histone H3, putative [Entamoeba histolytica HM-1:IMSS]	AHPGAVALTEIK	1206.684103	1	2.7432
	AHPGAVALTEIK	1206.684103	2	2.3897
	APFQALVR	901.525414	2	2.4068
	APFQALVR	901.525414	2	2.3321
	KAPFQALVR	1029.620377	2	2.0057
	KAPFQALVR	1029.620377	2	1.8679
gi|56468432|gb|EAL46278.1| protein kinase, putative [Entamoeba histolytica HM-1:IMSS]	DFIESTVK	938.482952	1	1.6625
gi|56468435|gb|EAL46281.1| hypothetical protein 153.t00016 [Entamoeba histolytica HM-1:IMSS]	REEEQWEEQFYDEQGY	2164.873045	2	3.1195
gi|56468458|gb|EAL46302.1| actophorin, putative [Entamoeba histolytica HM-1:IMSS]	MNDGMTEVVVEK	1351.623219	3	1.753
	NATYDDFLK	1086.51022	2	1.6114
	YAVYDLEYDTPEGLR	1803.84358	2	4.449
	YAVYDLEYDTPEGLR	1803.84358	2	4.06
gi|56468480|gb|EAL46322.1| calmodulin, putative [Entamoeba histolytica HM-1:IMSS]	LTEQEVDDLLK	1302.678743	2	2.5402
gi|56468495|gb|EAL46336.1| Rho family GTPase [Entamoeba histolytica HM-1:IMSS]	EMIAKVK	818.480437	1	1.5173
gi|56468506|gb|EAL46347.1| protein kinase, putative [Entamoeba histolytica HM-1:IMSS]	PMIVCSIENKNMK	1563.747714	2	1.6026
gi|56468549|gb|EAL46386.1| conserved hypothetical protein [Entamoeba histolytica HM-1:IMSS]	NALDLVYIMVK	1278.712623	2	1.8679
gi|56468567|gb|EAL46401.1| hypothetical protein 147.t00011 [Entamoeba histolytica HM-1:IMSS]	M*KMALESSNVSK	1340.655979	3	1.644
gi|56468580|gb|EAL46413.1| Rho family GTPase [Entamoeba histolytica HM-1:IMSS]	INLGLWDTAGQEDYDR	1865.866444	2	2.5676
gi|56468613|gb|EAL46445.1| Vacuolar sorting protein 9 (VPS9) domain, putative [Entamoeba histolytica HM-1:IMSS]	AFM*ALNGTVGILKNEVIKYK	2225.237344	2	2.5566
gi|56468630|gb|EAL46461.1| hypothetical protein 144.t00010 [Entamoeba histolytica HM-1:IMSS]	AIM*NIISLEK	1147.640232	2	2.202
	AIM*NIISLEK	1147.640232	1	1.8624
	AIMNIISLEK	1131.644219	2	3.4761
	AIMNIISLEK	1131.644219	2	3.3575
	AIMNIISLEK	1131.644219	2	3.3247
	AIMNIISLEK	1131.644219	2	3.1296
	AIMNIISLEK	1131.644219	1	2.216
	AIMNIISLEK	1131.644219	1	2.2009
	INKEEIIR	1014.594223	3	2.0824
	LVPIEQYDK	1104.593555	1	1.6296
gi|56468634|gb|EAL46465.1| conserved hypothetical protein [Entamoeba histolytica HM-1:IMSS]	NVLKTDISM*K	1164.630396	2	1.922
gi|56468654|gb|EAL46483.1| translation elongation factor EF-1 alpha, putative [Entamoeba histolytica HM-1:IMSS]	ISGIGTVPVGR	1055.620789	2	2.4225

	ISGIGTVPVGR	1055.620789	2	2.0562
	ISGIGTVPVGR	1055.620789	2	1.9895
gi|56468671|gb|EAL46497.1| thioredoxin, putative [Entamoeba histolytica HM-1:IMSS]	IVEPGYDELESFMDEASK	2058.921259	2	1.6611
gi|56468708|gb|EAL46531.1| hypothetical protein 141.t00010 [Entamoeba histolytica HM-1:IMSS]	KVNEGNK	788.4260974	2	1.9233
gi|56468741|gb|EAL46560.1| hypothetical protein 139.t00012 [Entamoeba histolytica HM-1:IMSS]	EVVLFIAK	918.565882	2	1.9177
gi|56468756|gb|EAL46574.1| RNA-binding protein, putative [Entamoeba histolytica HM-1:IMSS]	KIRDVLPNIPVHIPPSLDEK	2280.307443	2	1.5793
gi|56468760|gb|EAL46577.1| hypothetical protein 138.t00014 [Entamoeba histolytica HM-1:IMSS]	AKFLNTLHVMMLLLRNQE	2171.182764	2	1.6891
gi|56468768|gb|EAL46584.1| DNA topoisomerase II, putative [Entamoeba histolytica HM-1:IMSS]	VYTSIDELR	1095.568079	2	1.7201
gi|56468769|gb|EAL46585.1| conserved hypothetical protein [Entamoeba histolytica HM-1:IMSS]	SIENAFTEIR	1179.600442	2	2.7616
gi|56468770|gb|EAL46586.1| hypothetical protein 138.t00006 [Entamoeba histolytica HM-1:IMSS]	KVLIFLNEIPK	1313.819137	2	1.7177
	KVLIFLNEIPK	1313.819137	2	1.5799
gi|56468781|gb|EAL46594.1| 40S ribosomal protein S24, putative [Entamoeba histolytica HM-1:IMSS]	QVVVDIYHPGIVQPK	1691.947923	2	3.0608
gi|56468788|gb|EAL46601.1| phenylalanyl-tRNA synthetase alpha subunit, putative [Entamoeba histolytica HM-1:IMSS]	SLDAAGIIISEMK	1347.718854	2	1.8511
gi|56468789|gb|EAL46602.1| hypothetical protein 137.t00009 [Entamoeba histolytica HM-1:IMSS]	M*YNKVLNK	1025.545927	2	1.9196
gi|56468795|gb|EAL46608.1| protein kinase, putative [Entamoeba histolytica HM-1:IMSS]	FMSEIANK	939.460441	1	1.5468
	GIIALPVQENLK	1294.772918	3	1.8856
gi|56468803|gb|EAL46614.1| protein kinase, putative [Entamoeba histolytica HM-1:IMSS]	FMKTNPM*SDLLKVR	1695.893169	2	1.9442
gi|56468853|gb|EAL46661.1| hypothetical protein 134.t00016 [Entamoeba histolytica HM-1:IMSS]	QQHMKRSLPTISQTK	1782.964336	3	2.0604
gi|56468877|gb|EAL46684.1| 60S ribosomal protein L17, putative [Entamoeba histolytica HM-1:IMSS]	GLNTALLTVSHIAVQEAR	1893.055253	2	3.7358
gi|56468896|gb|EAL46702.1| hypothetical protein 133.t00008 [Entamoeba histolytica HM-1:IMSS]	IVGVVEK	743.4661704	1	2.1763
gi|56468901|gb|EAL46705.1| hypothetical membrane-spanning protein [Entamoeba histolytica HM-1:IMSS]	DPQAEEAPAEDPEVDK	1739.760638	2	4.9232
	EHPQPVEQEESPIFIMIK	2151.079083	3	3.9721
	EHPQPVEQEESPIFIMIK	2151.079083	2	2.7658
	EHPQPVEQEESPIFIMIK	2151.079083	2	2.6876
	EHPQPVEQEESPIFIMIK	2151.079083	2	2.5075
	SMPVFLQR	977.523709	2	2.3137
gi|56468935|gb|EAL46736.1| hypothetical protein 131.t00012 [Entamoeba histolytica HM-1:IMSS]	DKVQTIAKLISLGADITIK	2027.211085	2	1.6416
gi|56468970|gb|EAL46768.1| importin beta subunit, putative [Entamoeba histolytica HM-1:IMSS]	YKEAAIFVFGCIICK	1818.885186	2	2.2689
gi|56468996|gb|EAL46793.1| hypothetical protein 129.t00022 [Entamoeba histolytica HM-1:IMSS]	AVNSVITKTFKK	1335.799475	2	1.8736
gi|56469015|gb|EAL46810.1| hypothetical protein 128.t00004 [Entamoeba histolytica HM-1:IMSS]	IEQYTYLVK	1156.624855	2	1.6887
	LSVVDVYEDK	1166.59396	2	2.7899
gi|56469039|gb|EAL46832.1| short chain dehydrogenase family protein [Entamoeba histolytica HM-1:IMSS]	LAEPLAELK	983.577176	2	3.2847
gi|56469039|gb|EAL46832.1| short chain dehydrogenase family protein [Entamoeba histolytica HM-1:IMSS]	LAEPLAELK	983.577176	2	2.8094
gi|56469044|gb|EAL46837.1| zinc finger protein, putative [Entamoeba histolytica HM-1:IMSS]	DMIEIK	748.390954	1	1.5101
gi|56469053|gb|EAL46846.1| hypothetical protein 127.t00023 [Entamoeba histolytica HM-1:IMSS]	QVIDDTKK	946.52039	1	1.6402
gi|56469101|gb|EAL46888.1| hypothetical protein 125.t00002 [Entamoeba histolytica HM-1:IMSS]	ENEGESVGPNGQK	1344.602636	3	1.9674
gi|56469103|gb|EAL46890.1| predicted protein [Entamoeba histolytica HM-1:IMSS]	IINSGTIFGGTEKVLR	1704.964317	3	1.589
gi|56469118|gb|EAL46904.1| conserved hypothetical protein [Entamoeba histolytica HM-1:IMSS]	IAEIIKMR	973.586299	2	1.7286
	YPYIFSAINNEK	1458.726371	2	1.6803
gi|56469166|gb|EAL46946.1| hypothetical protein 122.t00026 [Entamoeba histolytica HM-1:IMSS]	NISTKVK	789.482891	2	1.6712
gi|56469186|gb|EAL46961.1| DNA-directed RNA polymerase subunit, putative [Entamoeba histolytica HM-1:IMSS]	GKVLMGERGYIESVR	1693.905421	3	1.5111
gi|56469191|gb|EAL46966.1| Rab family GTPase [Entamoeba histolytica HM-1:IMSS] gi|56790054|dbj|BAD82823.1| EhRab(	ILIIGESGVGK	1085.656506	2	2.912
	ILIIGESGVGK	1085.656506	1	2.2634
gi|56469219|gb|EAL46989.1| protein kinase with WD repeats [Entamoeba histolytica HM-1:IMSS]	LRSIPDLGFVDSEIPSPK	1970.059356	3	2.1369
gi|56469223|gb|EAL46992.1| hypothetical protein 120.t00005 [Entamoeba histolytica HM-1:IMSS]	M*GITQSILNNKSNDKTK	1907.986627	3	1.8896
gi|56469226|gb|EAL46995.1| conserved hypothetical protein [Entamoeba histolytica HM-1:IMSS]	HIVLEK	738.450852	2	1.7996
gi|56469243|gb|EAL47006.1| START domain protein [Entamoeba histolytica HM-1:IMSS]	NFVRAQSLK	1062.605466	1	1.5419
gi|56469245|gb|EAL47008.1| Gal/GalNAc lectin subunit Igl2 [Entamoeba histolytica HM-1:IMSS]	DLYLPYYFSVTK	1508.767173	2	2.6767
	DVKNDCECNDK	1396.503831	2	1.7285
	GYDQYEQYLNAFK	1638.743471	2	4.2252
	GYDQYEQYLNAFK	1638.743471	2	3.7769
	LFSPTEENK	1064.52588	2	1.7185

gi|56469252|gb|EAL47015.1| receptor protein kinase, putative [Entamoeba histolytica HM-1:IMSS]	KGTANVVGDDHIEIVQKAR	2050.103988	2	2.0823
gi|56469323|gb|EAL47082.1| histone H2A, putative [Entamoeba histolytica HM-1:IMSS]	DVTISYGGVFPNVPTAVNSK	2065.060078	2	4.8278
	DVTISYGGVFPNVPTAVNSK	2065.060078	2	4.6378
	DVTISYGGVFPNVPTAVNSK	2065.060078	2	4.4115
	DVTISYGGVFPNVPTAVNSK	2065.060078	2	4.4092
	DVTISYGGVFPNVPTAVNSK	2065.060078	2	4.2425
	DVTISYGGVFPNVPTAVNSK	2065.060078	2	3.8459
	HIQLAVR	836.510098	2	2.2804
	HIQLAVR	836.510098	2	2.0424
	HIQLAVR	836.510098	2	1.8086
	HIQLAVR	836.510098	2	1.6619
	HIQLAVR	836.510098	1	1.5831
	IHAALK	652.414072	1	1.7206
	KSGLQFPVAR	1102.636768	3	1.914
	SDLELNDLLK	1159.620509	2	3.4801
	SDLELNDLLK	1159.620509	2	3.3884
	SDLELNDLLK	1159.620509	2	3.1842
	SDLELNDLLK	1159.620509	2	3.0395
	SDLELNDLLK	1159.620509	2	2.7533
	SDLELNDLLK	1159.620509	2	2.1601
	SDLELNDLLK	1159.620509	2	1.9446
	SGLQFPVAR	974.5418054	2	2.9181
	SGLQFPVAR	974.5418054	2	2.7881
	SGLQFPVAR	974.5418054	1	2.0797
	SGLQFPVAR	974.5418054	1	1.9503
	SGLQFPVAR	974.5418054	2	1.8872
	SGLQFPVAR	974.5418054	2	1.8518
	SGLQFPVAR	974.5418054	2	1.6468
gi|56469328|gb|EAL47087.1| 60S ribosomal protein L3, putative [Entamoeba histolytica HM-1:IMSS]	NDFLMIK	880.459702	2	1.9447
gi|56469342|gb|EAL47100.1| 60S ribosomal protein L9, putative [Entamoeba histolytica HM-1:IMSS]	KTEHVVIPEGVNIEINR	1947.065808	2	3.3199
gi|56469394|gb|EAL47148.1| hypothetical protein 115.t00032 [Entamoeba histolytica HM-1:IMSS]	EIAQEIYIR	1134.615353	2	2.0846
	VPEPFIEK	958.524412	2	1.5105
gi|56469398|gb|EAL47151.1| conserved hypothetical protein [Entamoeba histolytica HM-1:IMSS]	FVHLSNAQVYDSSSKPKDEK	2279.130299	3	1.919
gi|56469399|gb|EAL47152.1| 60S ribosomal protein L10, putative [Entamoeba histolytica HM-1:IMSS]	LFDIGNR	834.4468324	2	2.2181
	LVISNK	673.424313	1	1.6235
	LVISNK	673.424313	1	1.612
	LVISNK	673.424313	1	1.5897
	VGQVLISGR	928.5574578	2	2.0931
gi|56469422|gb|EAL47172.1| aminopeptidase, putative [Entamoeba histolytica HM-1:IMSS]	M*TEILPTNFIPLHYK	1832.96262	2	1.6972
gi|56469426|gb|EAL47176.1| protein kinase, putative [Entamoeba histolytica HM-1:IMSS]	EPLKGVFLTKLK	1372.856253	3	1.6756
gi|56469485|gb|EAL47231.1| hypothetical protein 112.t00021 [Entamoeba histolytica HM-1:IMSS]	TIILITLSLM*K	1261.781082	2	1.5928
gi|56469498|gb|EAL47242.1| conserved hypothetical protein [Entamoeba histolytica HM-1:IMSS]	IVQDVIDEKRVINIK	1782.048364	2	1.7218
gi|56469505|gb|EAL47248.1| leucine rich repeat protein [Entamoeba histolytica HM-1:IMSS]	LSISSKKFVGK	1193.725269	2	1.5784
gi|56469519|gb|EAL47261.1| hypothetical protein 110.t00018 [Entamoeba histolytica HM-1:IMSS]	ELLTKFTGNKGIVDFENNK	2167.139371	2	2.4173
gi|56469533|gb|EAL47274.1| Rho family GTPase [Entamoeba histolytica HM-1:IMSS]	KATNKHKCSLL	1299.698714	2	2.1616
gi|56469535|gb|EAL47276.1| hypothetical protein 110.t00008 [Entamoeba histolytica HM-1:IMSS]	ISNFTEKVM*KQVEAKSIK	2096.143125	2	2.2404
gi|56469539|gb|EAL47280.1| hypothetical protein 110.t00012 [Entamoeba histolytica HM-1:IMSS]	CGEVPKM*VKK	1191.602063	2	1.7778
gi|56469542|gb|EAL47282.1| conserved hypothetical protein [Entamoeba histolytica HM-1:IMSS]	SSTLNVQELARMGGIGTLLK	2088.148182	2	1.9906
gi|56469544|gb|EAL47284.1| protein phosphatase 2C, putative [Entamoeba histolytica HM-1:IMSS]	LNPRTIIRENIMLNQPTK	2151.206672	3	2.0892
gi|56469548|gb|EAL47288.1| patatin-like phospholipase, putative [Entamoeba histolytica HM-1:IMSS]	IISLDGGGIK	972.5724422	2	2.0007
gi|56469553|gb|EAL47293.1| conserved hypothetical protein [Entamoeba histolytica HM-1:IMSS]	FKERQIICNEAVKIVK	1975.094267	3	1.6502
gi|56469583|gb|EAL47318.1| protein kinase, putative [Entamoeba histolytica HM-1:IMSS]	M*KNSKGGHIGR	1200.627717	2	1.5398

gi|56469640|gb|EAL47370.1| hypothetical protein 106.t00028 [Entamoeba histolytica HM-1:IMSS]	SDVLGHIKWEEVGDDK	1826.89194	2	1.5404
gi|56469645|gb|EAL47374.1| 60S ribosomal protein L4, putative [Entamoeba histolytica HM-1:IMSS]	GHHIAGVPQIPLVVNSK	1766.007182	2	2.3396
	GPLVVYGNDDLQAVK	1587.837708	2	3.3367
	KGPLVVYGNDDLQAVK	1715.932671	3	4.3875
	KGPLVVYGNDDLQAVK	1715.932671	3	4.0153
	LNTIFGTQK	1021.567676	2	2.1969
	LPAVFLAPIR	1096.687728	2	1.6598
gi|56469648|gb|EAL47377.1| protein phosphatase, putative [Entamoeba histolytica HM-1:IMSS]	YNIKKYK	956.55638	2	1.6661
gi|56469662|gb|EAL47390.1| Rab family GTPase [Entamoeba histolytica HM-1:IMSS] gi|13537447|dbj|BAB40678.1| small G	IVLVGESGVGK	1057.625206	1	2.4876
	IVLVGESGVGK	1057.625206	1	2.438
	SVDGEQSNTNEIKPATEPIDLGSQNNKPEEK	3368.608876	3	4.3888
	SVDGEQSNTNEIKPATEPIDLGSQNNKPEEK	3368.608876	3	3.1037
gi|56469734|gb|EAL47455.1| eukaryotic translation initiation factor 2 alpha subunit, putative [Entamoeba histolytica HM-1:IMS	EYDDIQGMIPM*SELSRK	2027.942379	2	1.563
gi|56469738|gb|EAL47459.1| conserved hypothetical protein [Entamoeba histolytica HM-1:IMSS]	DIPENIAEMK	1159.566354	2	2.1914
	EFNDLVQFR	1167.579302	2	2.0082
	GIFSEDMIK	1039.512873	2	1.7562
	KLIVGK	657.4657754	1	1.9231
	KLIVGK	657.4657754	2	1.8952
	KLIVGK	657.4657754	2	1.8196
	KLIVGK	657.4657754	1	1.8073
	NVAVTLIK	857.545481	1	1.8822
	SIALAGVK	758.4770794	1	1.8081
	SIALAGVK	758.4770794	1	1.5746
	VIETSQR	832.45232	2	1.635
	WTEFDQAAEDAIIAK	1707.822448	2	4.9533
	WTEFDQAAEDAIIAK	1707.822448	2	3.3286
	YNTEKDVFDFDDDAKDAMK	2266.980878	2	3.2341
gi|56469772|gb|EAL47488.1| hypothetical protein 102.t00004 [Entamoeba histolytica HM-1:IMSS]	LM*KDVLSINDFIIAR	1763.973529	3	1.6601
gi|56469810|gb|EAL47520.1| cullin, putative [Entamoeba histolytica HM-1:IMSS]	KMQHNDLM*QAVVTSLSSR	2061.022702	2	1.7091
gi|56469832|gb|EAL47541.1| hypothetical protein 100.t00023 [Entamoeba histolytica HM-1:IMSS]	LNNDFNVFLQQRR	1663.866317	3	2.199
gi|56469854|gb|EAL47562.1| hypothetical protein 99.t00021 [Entamoeba histolytica HM-1:IMSS]	RSVKQNGTK	1017.579982	1	1.5091
gi|56469900|gb|EAL47603.1| conserved hypothetical protein [Entamoeba histolytica HM-1:IMSS]	RDYHYVYEALIDEFK	1960.94396	2	1.9238
gi|56469905|gb|EAL47608.1| protein kinase, putative [Entamoeba histolytica HM-1:IMSS]	M*SLKLASK	893.513584	1	1.5469
gi|56469911|gb|EAL47614.1| hypothetical protein 97.t00024 [Entamoeba histolytica HM-1:IMSS]	DKLVYVQPNK	1203.673202	2	1.5467
gi|56469936|gb|EAL47633.1| hypothetical protein 96.t00016 [Entamoeba histolytica HM-1:IMSS]	SQNQVKNAIK	1129.63241	1	1.6216
gi|56469937|gb|EAL47634.1| protein kinase, putative [Entamoeba histolytica HM-1:IMSS]	ELM*KEFKK	1068.576893	2	1.8585
gi|56469951|gb|EAL47647.1| ubiquitin-conjugating enzyme, putative [Entamoeba histolytica HM-1:IMSS]	DHPPGFVARPIM*KK	1608.868995	3	1.5639
gi|56469969|gb|EAL47664.1| hypothetical protein 95.t00026 [Entamoeba histolytica HM-1:IMSS]	YLKLENPLVALINKTQNK	2099.222306	2	1.5046
gi|56469970|gb|EAL47665.1| Rab family GTPase [Entamoeba histolytica HM-1:IMSS] gi|14423577|gb|AAK62471.1| small GT	LLLIGESGVGK	1085.656506	2	2.7695
gi|56469996|gb|EAL47688.1| hypothetical protein 94.t00003 [Entamoeba histolytica HM-1:IMSS]	KGSNVIIK	858.5407424	2	1.6205
gi|56470088|gb|EAL47773.1| 60S ribosomal protein L23, putative [Entamoeba histolytica HM-1:IMSS]	MHTAVIIR	940.539683	2	1.9319
	MHTAVIIR	940.539683	1	1.8093
	TLTIISVK	874.560807	2	2.2418
	TLTIISVK	874.560807	2	1.5128
	TLTIISVKGFHGRLNRLPR	2178.298219	2	1.5785
gi|56470089|gb|EAL47774.1| hypothetical protein 92.t00004 [Entamoeba histolytica HM-1:IMSS]	SFHKILNYSGIMTDCGK	1970.924844	2	2.0012
	TKKSIPSTPGEVKR	1527.885343	3	1.8854
gi|56470105|gb|EAL47789.1| TBP interacting protein, putative [Entamoeba histolytica HM-1:IMSS]	ELENKRQELKVTLSTTK	2017.128811	2	1.5631
gi|56470123|gb|EAL47806.1| nucleotide binding protein 2, putative [Entamoeba histolytica HM-1:IMSS]	MMGLDNQGVYQGEHGGILPAK	2215.063451	3	1.8455
gi|56470151|gb|EAL47832.1| 70 kDa heat shock protein, putative [Entamoeba histolytica HM-1:IMSS]	IVNEPTAASMAFGLNSFK	1896.952437	2	1.7667
gi|56470154|gb|EAL47835.1| hypothetical protein 90.t00022 [Entamoeba histolytica HM-1:IMSS]	IFSNTRSNGEIM*KIVR	1881.002216	3	2.2603
gi|56470178|gb|EAL47858.1| splicing factor Prp8, putative [Entamoeba histolytica HM-1:IMSS]	QILMSSSATTFTRVVNK	1883.005543	3	2.4103

gi|56470198|gb|EAL47876.1| hypothetical protein 88.t00012 [Entamoeba histolytica HM-1:IMSS]	RTSLPNALPFKINATPFIPK	2225.280499	2	1.5289
gi|56470207|gb|EAL47885.1| hypothetical protein 88.t00021 [Entamoeba histolytica HM-1:IMSS]	MEEM*NKPKAAEPIYK	1794.87757	2	1.7124
gi|56470246|gb|EAL47922.1| Rho family GTPase [Entamoeba histolytica HM-1:IMSS] gi|56464625|gb|EAL43078.1| Rho fami	VIVIGDTNVGK	1114.646658	2	2.6574
	VIVIGDTNVGK	1114.646658	1	2.0663
	VSMGLLDAVVSEAPQAK	1714.904424	2	3.1878
gi|56470268|gb|EAL47941.1| hypothetical protein 86.t00031 [Entamoeba histolytica HM-1:IMSS]	M*SVEKGGIFEETVRKAIEGK	2224.165315	2	1.835
gi|56470281|gb|EAL47953.1| DENN domain protein [Entamoeba histolytica HM-1:IMSS]	DEETQRIKISMK	1477.767918	2	1.6021
gi|56470318|gb|EAL47988.1| hypothetical protein 85.t00008 [Entamoeba histolytica HM-1:IMSS]	VSAEESAEAESKK	1364.654016	2	1.9803
gi|56470319|gb|EAL47989.1| hypothetical protein 85.t00009 [Entamoeba histolytica HM-1:IMSS]	GKGEMDENTR	1136.500071	2	2.0169
gi|56470329|gb|EAL47998.1| hypothetical protein 84.t00015 [Entamoeba histolytica HM-1:IMSS]	WCEQLKISKYPVLAR	1891.004399	3	1.9303
gi|56470330|gb|EAL47999.1| Beige protein, putative [Entamoeba histolytica HM-1:IMSS]	ARVSVLLK	885.588024	2	1.8946
gi|56470331|gb|EAL48000.1| histone H3, putative [Entamoeba histolytica HM-1:IMSS]	STELLLR	831.493456	2	2.1416
	STELLLR	831.493456	1	1.7655
	VTIMPK	688.406209	2	1.6532
gi|56470336|gb|EAL48005.1| hypothetical protein 84.t00022 [Entamoeba histolytica HM-1:IMSS]	LLLPLYQGYVEK	1435.819534	2	1.7873
gi|56470347|gb|EAL48015.1| protein kinase, putative [Entamoeba histolytica HM-1:IMSS]	KVEIEK	745.445433	2	2.3975
gi|56470406|gb|EAL48069.1| hypothetical protein 82.t00013 [Entamoeba histolytica HM-1:IMSS]	FGSIIIK	777.4869154	2	1.755
gi|56470408|gb|EAL48071.1| hypothetical protein 82.t00015 [Entamoeba histolytica HM-1:IMSS]	NKDSIKVMCDKYM*SK	1862.860559	2	1.5322
gi|56470472|gb|EAL48126.1| hypothetical protein 80.t00019 [Entamoeba histolytica HM-1:IMSS]	IVEISDIPK	1013.587751	2	1.6421
gi|56470539|gb|EAL48184.1| sulfate adenylyltransferase, putative [Entamoeba histolytica HM-1:IMSS]	DHAGVGDYYGPFDAQK	1739.766003	3	1.7367
	ILADYYQHLDK	1378.700146	3	1.9611
gi|56470551|gb|EAL48195.1| hypothetical protein 78.t00015 [Entamoeba histolytica HM-1:IMSS]	ILLSAENDVPDIK	1426.778801	2	1.6949
gi|56470573|gb|EAL48216.1| conserved hypothetical protein [Entamoeba histolytica HM-1:IMSS]	LDSLNALSVIADK	1358.752596	2	1.7314
gi|56470587|gb|EAL48229.1| Importin beta family protein [Entamoeba histolytica HM-1:IMSS]	SKAQQLNQVILTATDQKQR	2170.193871	2	1.6985
gi|56470602|gb|EAL48244.1| beta-amylase, putative [Entamoeba histolytica HM-1:IMSS]	TINQVASKK	988.578583	1	1.5293
gi|56470614|gb|EAL48253.1| hypothetical protein 76.t00003 [Entamoeba histolytica HM-1:IMSS]	TKEKYAEEIVEFLKK	1855.021147	3	1.7983
gi|56470618|gb|EAL48257.1| cation-transporting P-typeATPase, putative [Entamoeba histolytica HM-1:IMSS]	YIYNTEDGLFYPLEYPNK	2239.059387	2	2.2858
gi|56470621|gb|EAL48260.1| hypothetical protein 76.t00010 [Entamoeba histolytica HM-1:IMSS]	INEIDLKNTLYEK	1592.853019	2	1.7313
gi|56470627|gb|EAL48266.1| hypothetical protein 76.t00016 [Entamoeba histolytica HM-1:IMSS]	RKSGVNFL	920.5312404	2	1.7926
gi|56470646|gb|EAL48283.1| hypothetical protein 75.t00004 [Entamoeba histolytica HM-1:IMSS]	EKDILISTINLFK	1533.888685	2	2.1655
	EKDILISTINLFK	1533.888685	2	1.7472
	HRASVGSRNDMFNSFDERK	2253.05781	2	1.5557
gi|56470648|gb|EAL48285.1| myotubularin, putative [Entamoeba histolytica HM-1:IMSS]	LLHIYSENK	1116.604798	3	1.8516
gi|56470652|gb|EAL48289.1| protein kinase, putative [Entamoeba histolytica HM-1:IMSS]	PENVLVVSM*VK	1230.677346	2	2.1471
	PENVLVVSM*VK	1230.677346	2	2.0381
	TTLGNYTLYKTIK	1515.841727	3	2.4436
gi|56470669|gb|EAL48306.1| hypothetical protein 75.t00028 [Entamoeba histolytica HM-1:IMSS]	IDISQVFK	949.535321	2	1.9125
gi|56470691|gb|EAL48325.1| b-keto acyl reductase, putative [Entamoeba histolytica HM-1:IMSS]	VIANDLISIDENNQWNK	1985.992712	2	3.8282
	VIANDLISIDENNQWNK	1985.992712	2	2.3561
gi|56470708|gb|EAL48342.1| DnaJ family protein [Entamoeba histolytica HM-1:IMSS]	TVSDEELK	920.457132	1	1.7013
gi|56470764|gb|EAL48393.1| conserved hypothetical protein [Entamoeba histolytica HM-1:IMSS]	M*MLPM*SVR	996.469711	2	1.5238
gi|56470767|gb|EAL48396.1| hypothetical protein 72.t00038 [Entamoeba histolytica HM-1:IMSS]	MQLKEIQADARSPRR	1798.970474	3	2.0007
gi|56470825|gb|EAL48443.1| hypothetical protein 71.t00010 [Entamoeba histolytica HM-1:IMSS]	DQRCVM*TLMKLSDY	1775.792135	3	1.5524
	KHIQTFEK	1030.568008	1	1.5609
gi|56470847|gb|EAL48464.1| protein kinase, putative [Entamoeba histolytica HM-1:IMSS]	VIDTAKSDPQK	1201.642307	2	2.0622
gi|56470860|gb|EAL48476.1| hypothetical protein 70.t00012 [Entamoeba histolytica HM-1:IMSS]	KNTTIIK	817.514181	1	1.6079
gi|56470913|gb|EAL48524.1| hypothetical protein 68.t00007 [Entamoeba histolytica HM-1:IMSS]	IIITNKNM*K	1090.629991	1	1.807
gi|56470940|gb|EAL48551.1| Receptor mediated endocytosis protein, putative [Entamoeba histolytica HM-1:IMSS]	VYVSSFWDQPFK	1502.731466	2	1.6155
gi|56470967|gb|EAL48573.1| protein kinase, putative [Entamoeba histolytica HM-1:IMSS]	PLFPGSSTLNQLDK	1516.800611	2	1.6539
gi|56470972|gb|EAL48578.1| 40S ribosomal protein S15a, putative [Entamoeba histolytica HM-1:IMSS]	FNVTLSEFDK	1199.594294	1	2.0892
	FNVTLSEFDK	1199.594294	2	1.8883
	HQYITDYTVVDTHR	1747.839829	3	5.062

	HQYITDYTVVDTHR	1747.839829	3	4.3635
	ILGFFY	759.4075924	1	1.9147
	ILGFFY	759.4075924	1	1.8884
	ILGFFY	759.4075924	1	1.8617
	IVVNLNGR	884.5312304	2	1.9583
	VNVLADALK	942.56186	2	2.8968
	VNVLADALK	942.56186	2	2.5567
	VNVLADALK	942.56186	1	2.2079
	VNVLADALK	942.56186	1	2.1502
gi|56471012|gb|EAL48615.1| 60S ribosomal protein L10a, putative [Entamoeba histolytica HM-1:IMSS]	GWQNIKCLYIK	1422.734757	2	1.9392
gi|56471020|gb|EAL48621.1| autophagy protein apg7, putative [Entamoeba histolytica HM-1:IMSS]	AQRAAEMVKEVFPGMK	1791.924426	3	1.5605
gi|56471058|gb|EAL48654.1| RNA-binding protein, putative [Entamoeba histolytica HM-1:IMSS]	QTIFVGNLPFK	1263.709589	2	2.5952
gi|56471076|gb|EAL48672.1| BspA-like leucine rich repeat protein, putative [Entamoeba histolytica HM-1:IMSS]	EYFISK	786.403244	1	1.8054
gi|56471105|gb|EAL48699.1| hypothetical protein 63.t00015 [Entamoeba histolytica HM-1:IMSS]	DITM*GNQHYYALTTNNCVLVIDK	2699.260006	3	2.5879
gi|56471175|gb|EAL48765.1| hypothetical protein 61.t00020 [Entamoeba histolytica HM-1:IMSS]	FGGYANEKIDKYEMKK	1920.95242	2	2.0053
gi|56471187|gb|EAL48777.1| hypothetical protein 61.t00032 [Entamoeba histolytica HM-1:IMSS]	NECM*RLFM*KIK	1501.71312	2	1.6756
gi|56471198|gb|EAL48788.1| hypothetical protein 61.t00043 [Entamoeba histolytica HM-1:IMSS]	ITSPTTNPK	958.5204	2	1.9752
gi|56471199|gb|EAL48789.1| Glycerophosphoryl diester phosphodiesterase, putative [Entamoeba histolytica HM-1:IMSS]	DVRISIELK	1072.636098	2	1.5504
gi|56471213|gb|EAL48800.1| hypothetical protein 60.t00018 [Entamoeba histolytica HM-1:IMSS]	SLSKTNESPR	1118.580061	2	1.7312
gi|56471215|gb|EAL48802.1| hypothetical protein 60.t00020 [Entamoeba histolytica HM-1:IMSS]	TVDSLFKTVIKYNEIKSK	2113.190358	2	1.9286
gi|56471217|gb|EAL48804.1| 40S ribosomal protein S7, putative [Entamoeba histolytica HM-1:IMSS]	EAVAGIPELK	1026.582992	2	3.0656
	EAVAGIPELK	1026.582992	2	2.9712
	EAVAGIPELK	1026.582992	2	2.7839
	EAVAGIPELK	1026.582992	1	2.2709
	EAVAGIPELK	1026.582992	1	1.9848
	EAVAGIPELK	1026.582992	1	1.8359
	LPIYSAVYK	1053.597921	2	1.8886
	LPIYSAVYK	1053.597921	1	1.7414
	VIEALEK	801.471648	1	2.3643
	VIEALEK	801.471648	1	2.2269
	VIEALEK	801.471648	1	1.9856
	VIEALEK	801.471648	2	1.9479
gi|56471228|gb|EAL48814.1| myosin heavy chain [Entamoeba histolytica HM-1:IMSS]	VEDLEGQLEKAK	1358.716193	2	3.6487
	VEDLEGQLEKAK	1358.716193	2	3.1641
	VEDLEGQLEKAK	1358.716193	2	2.3018
gi|56471256|gb|EAL48839.1| DNA-directed RNA polymerase subunit N, putative [Entamoeba histolytica HM-1:IMSS]	VPMDQVFSELGIER	1619.809785	2	2.5076
gi|56471260|gb|EAL48843.1| nucleolar protein Nop56, putative [Entamoeba histolytica HM-1:IMSS]	AANSSMGFDVNEFDLQNIK	2099.970274	2	3.3383
gi|56471267|gb|EAL48850.1| ribosomal protein L7Ae-related protein [Entamoeba histolytica HM-1:IMSS]	ALNRGKADIVIIAADTK	1769.027965	3	1.7753
gi|56471273|gb|EAL48856.1| helicase, putative [Entamoeba histolytica HM-1:IMSS]	NNNINIILM*K	1202.657269	1	1.5869
gi|56471290|gb|EAL48870.1| BspA-like leucine rich repeat protein, putative [Entamoeba histolytica HM-1:IMSS]	QLKEIHLDSATDLK	1610.874827	3	1.5792
gi|56471292|gb|EAL48872.1| hypothetical protein 58.t00008 [Entamoeba histolytica HM-1:IMSS]	MKQDECIITKTGSKK	1766.892468	2	2.198
	MKQDECIITKTGSKK	1766.892468	2	1.6492
gi|56471314|gb|EAL48894.1| unconventional myosin IB [Entamoeba histolytica HM-1:IMSS]	GVDDLVMLPK	1086.586362	2	1.5655
gi|56471344|gb|EAL48921.1| hypothetical protein 57.t00005 [Entamoeba histolytica HM-1:IMSS]	TIVEFDNSAQEIK	1493.74823	2	2.132
gi|56471345|gb|EAL48922.1| hypothetical protein 57.t00007 [Entamoeba histolytica HM-1:IMSS]	YMEAIQK	882.438967	1	1.5297
gi|56471375|gb|EAL48950.1| galactose-inhibitable lectin small subunit, putative [Entamoeba histolytica HM-1:IMSS]	QGNELQFFEFAPYR	1745.828203	2	3.6445
	QGNELQFFEFAPYR	1745.828203	2	3.6394
gi|56471403|gb|EAL48974.1| 40S ribosomal protein S4, putative [Entamoeba histolytica HM-1:IMSS]	GTETLVNLPLDK	1299.715464	2	3.7435
	GTETLVNLPLDK	1299.715464	2	3.575
	KPLLQQVNETIK	1410.831494	3	2.8582
	LNLETGK	774.4355994	2	1.9465
	LNLETGK	774.4355994	2	1.7483

	LNNVFVIGK	1003.593496	2	3.0852
	LNNVFVIGK	1003.593496	2	1.6671
	YPVGFMDVLSIPR	1493.782112	2	3.6838
gi|56471426|gb|EAL48996.1| ankyrin repeat protein, putative [Entamoeba histolytica HM-1:IMSS]	TPFSSIQFKINKK	1537.873713	2	1.8512
	TPFSSIQFKINKK	1537.873713	2	1.6751
gi|56471442|gb|EAL49011.1| Rho guanine nucleotide exchange factor, putative [Entamoeba histolytica HM-1:IMSS]	LSVIGMYNIESIM*IKDIK	2083.118885	2	2.0264
gi|56471454|gb|EAL49023.1| hypothetical protein 54.t00035 [Entamoeba histolytica HM-1:IMSS]	FLEGLKDNWQNTQLK	1833.949381	2	1.6061
gi|56471496|gb|EAL49060.1| Gal/GalNAc lectin subunit Igl1 [Entamoeba histolytica HM-1:IMSS]	TAFSYYDK	994.451652	2	1.7447
	TAFSYYDK	994.451652	2	1.7385
gi|56471525|gb|EAL49089.1| heat shock protein 70, putative [Entamoeba histolytica HM-1:IMSS]	VEEILM*KKWKEIDK	1804.988835	3	1.9729
gi|56471546|gb|EAL49107.1| hypothetical protein 52.t00005 [Entamoeba histolytica HM-1:IMSS]	KYKLTEQLIANDKELLK	2047.179773	2	1.9999
gi|56471561|gb|EAL49122.1| immuno-dominant variable surface antigen [Entamoeba histolytica HM-1:IMSS]	NQGLSGWNYVSDGYSTIYK	2151.998219	2	4.5571
	SPETGEMELLEK	1362.64574	2	3.6906
	SYYGLWYENNFESK	1799.79117	2	3.6244
gi|56471604|gb|EAL49161.1| hypothetical protein 50.t00039 [Entamoeba histolytica HM-1:IMSS]	KEEGNDDIIK	1160.579365	1	1.7327
gi|56471639|gb|EAL49192.1| 40S ribosomal protein S2, putative [Entamoeba histolytica HM-1:IMSS]	ATYDAVR	795.399546	2	1.6626
	EEVMTMQSVQK	1309.612662	2	2.5067
	EVAEAIK	759.424698	1	1.7656
	EVAEAIK	759.424698	2	1.6828
	EVAEAIK	759.424698	1	1.6395
	EVAEAIK	759.424698	1	1.5733
	EYQIVDHLLPGLK	1524.842061	2	3.2408
	EYQIVDHLLPGLK	1524.842061	2	2.1305
	GAMIQAK	718.3916244	1	2.0474
	GAMIQAK	718.3916244	1	2.0408
	GAMIQAK	718.3916244	1	1.9028
	GAMIQAK	718.3916244	1	1.8839
	GTGIVAAPTPK	1011.583329	2	2.767
	ILAESK	660.392679	1	1.8349
	KDGASDGWTPVTK	1361.66959	3	2.3359
	LAMVPVR	785.470206	2	2.0301
	LVPAPR	652.414072	1	1.5901
	SIDQIFEFGIK	1296.683445	2	2.6152
	VLNSYFIGQDSTYR	1662.812239	2	3.4638
gi|56471647|gb|EAL49200.1| hypothetical protein 49.t00034 [Entamoeba histolytica HM-1:IMSS]	M*DLKEAFENKLEITNTPSK	2224.11769	2	1.7904
gi|56471693|gb|EAL49240.1| hypothetical protein 48.t00005 [Entamoeba histolytica HM-1:IMSS]	EM*KRYCVVAQNPEMMK	2029.912256	2	1.9031
	FFNVQNDEEEVAEETNAQ	2112.899258	2	5.3511
	FFNVQNDEEEVAEETNAQ	2112.899258	2	3.2722
	KDGFELK	836.4512494	2	2.0037
	KLVTFFR	910.5509	2	2.4794
	KLVTFFR	910.5509	2	2.1802
gi|56471706|gb|EAL49253.1| chromodomain-helicase-DNA-binding protein, putative [Entamoeba histolytica HM-1:IMSS]	ILAAGATITHIQR	1364.800864	3	1.5442
gi|56471720|gb|EAL49265.1| fatty acid elongase, putative [Entamoeba histolytica HM-1:IMSS]	VLQLAFGSGLKVNSAVWK	1917.095673	3	1.6718
gi|56471733|gb|EAL49278.1| pyruvate phosphate dikinase [Entamoeba histolytica HM-1:IMSS]	MPEGLM*EEVK	1178.544275	2	1.8743
	NMPANYAEAPLVK	1417.714415	2	2.313
gi|56471734|gb|EAL49279.1| conserved hypothetical protein [Entamoeba histolytica HM-1:IMSS]	SNGGSRKK	833.4588168	2	1.5155
	VKAMEFFLR	1140.623413	2	1.5951
gi|56471740|gb|EAL49285.1| hypothetical protein 47.t00023 [Entamoeba histolytica HM-1:IMSS]	HEMIFDEEVK	1276.587818	2	1.6329
	PSLVLLNK	883.561141	2	1.8279
	TPLSINFVLSK	1218.709273	1	1.6285
gi|56471742|gb|EAL49287.1| hypothetical protein 47.t00025 [Entamoeba histolytica HM-1:IMSS]	ISEFEFEDLKAR	1483.74275	3	1.7379
gi|56471745|gb|EAL49290.1| hypothetical protein 47.t00029 [Entamoeba histolytica HM-1:IMSS]	IKMSEEIEDSHENYQK	1979.901524	3	1.5964

gi|56471772|gb|EAL49316.1| amino acid transporter, putative [Entamoeba histolytica HM-1:IMSS]	KDFGKGSIKTPYTFK	1716.931952	2	1.7554
gi|56471801|gb|EAL49344.1| hypothetical protein 46.t00005 [Entamoeba histolytica HM-1:IMSS]	GIEINKEIKLK	1284.788568	2	1.6734
gi|56471809|gb|EAL49351.1| 70 kDa heat shock protein, putative [Entamoeba histolytica HM-1:IMSS]	GINPDEAVAYGAAVQGGVLSNIK	2243.166664	2	3.8254
	IAGLNVLR	855.5410664	2	1.7294
	NQIAQNPENTIFEIK	1758.902095	2	3.2535
	NQLENYAYQIK	1383.69031	2	3.3366
	VTEYLVDVFK	1212.65107	2	3.2689
	VTEYLVDVFK	1212.65107	2	2.6662
gi|56471815|gb|EAL49357.1| hypothetical protein 45.t00011 [Entamoeba histolytica HM-1:IMSS]	DAIQGPFVVPSNADEIK	1799.917423	2	4.0252
	DAIQGPFVVPSNADEIK	1799.917423	2	3.674
	DAIQGPFVVPSNADEIK	1799.917423	2	3.0873
	DAIQGPFVVPSNADEIK	1799.917423	2	2.9045
	DAIQGPFVVPSNADEIK	1799.917423	2	2.4894
	DAIQGPFVVPSNADEIK	1799.917423	2	2.194
gi|56471845|gb|EAL49385.1| hypothetical protein 44.t00033 [Entamoeba histolytica HM-1:IMSS]	LFNCFLSDTNPLIREFTAK	2286.137266	2	1.7964
gi|56471880|gb|EAL49413.1| hypothetical protein 43.t00025 [Entamoeba histolytica HM-1:IMSS]	PMAETSVAIDIPRLVSK	1827.00447	2	1.6385
gi|56471885|gb|EAL49418.1| hypothetical protein 43.t00030 [Entamoeba histolytica HM-1:IMSS]	M*KM*MKMMMMKK	1480.673107	2	1.7054
gi|56471915|gb|EAL49447.1| hypothetical protein 43.t00023 [Entamoeba histolytica HM-1:IMSS]	EKFIEMM*PAER	1396.661034	2	1.8244
gi|56471984|gb|EAL49508.1| ABC transporter, putative [Entamoeba histolytica HM-1:IMSS]	IPDLSTLPK	983.577186	1	1.5773
gi|56472018|gb|EAL49538.1| leucine rich repeat protein [Entamoeba histolytica HM-1:IMSS]	YVSKHIYSAIR	1336.737219	3	1.5176
gi|56472035|gb|EAL49554.1| steroid 5-alpha reductase, putative [Entamoeba histolytica HM-1:IMSS]	DLGAQLPFR	1016.55236	2	2.9006
	DLGAQLPFR	1016.55236	2	2.6908
	DLGAQLPFR	1016.55236	2	2.5512
	DLGAQLPFR	1016.55236	1	1.7898
	TLQESGVTEDTPIEMK	1777.85244	2	4.7256
	TLQESGVTEDTPIEMK	1777.85244	2	4.3592
	TLQESGVTEDTPIEMK	1777.85244	2	4.0298
	YLHMFPDYPK	1310.623808	3	3.2198
	YLHMFPDYPK	1310.623808	3	3.1997
gi|56472050|gb|EAL49568.1| rRNA biogenesis protein, putative [Entamoeba histolytica HM-1:IMSS]	IQEGEEIEGIISR	1472.759134	2	2.7908
gi|56472100|gb|EAL49612.1| alanyl-tRNA synthetase, putative [Entamoeba histolytica HM-1:IMSS]	GKGTGVHNLVILK	1335.810705	2	2.0027
gi|56472154|gb|EAL49662.1| enhancer binding protein 2, putative [Entamoeba histolytica HM-1:IMSS]	EGNNEM*LRGRNIR	1574.782704	2	1.5878
	NPQSFDKESK	1179.564067	2	1.6717
gi|56472159|gb|EAL49667.1| hypothetical protein 37.t00024 [Entamoeba histolytica HM-1:IMSS]	QTELQTLR	988.542188	2	1.8224
gi|56472176|gb|EAL49684.1| protein kinase, putative [Entamoeba histolytica HM-1:IMSS]	FPADIFAFGM*VIFETIK	1962.009238	3	2.3891
gi|56472185|gb|EAL49692.1| patatin-like phospholipase, putative [Entamoeba histolytica HM-1:IMSS]	IIEQEELPSLELLEK	1782.973539	2	1.6378
gi|56472209|gb|EAL49712.1| hypothetical protein 36.t00042 [Entamoeba histolytica HM-1:IMSS]	TVQAARKSR	1016.595964	2	1.7022
gi|56472240|gb|EAL49739.1| hypothetical protein 35.t00038 [Entamoeba histolytica HM-1:IMSS]	VIEIDEERFVDFATM*K	1957.958659	2	1.5427
gi|56472283|gb|EAL49780.1| histone H2B [Entamoeba histolytica HM-1:IMSS]	IATEASSLAR	1018.552773	2	2.7219
	IATEASSLAR	1018.552773	1	1.5496
gi|56472297|gb|EAL49792.1| ubiquitin [Entamoeba histolytica HM-1:IMSS]	ESTLHLVLR	1067.620782	2	2.0353
gi|56472302|gb|EAL49797.1| hypothetical protein 34.t00038 [Entamoeba histolytica HM-1:IMSS]	GNSIYETEYLIEK	1558.763548	2	4.2061
gi|56472305|gb|EAL49800.1| conserved hypothetical protein [Entamoeba histolytica HM-1:IMSS]	NKTIITSEEFEK	1438.742416	3	1.7345
gi|56472310|gb|EAL49805.1| polyadenylate-binding protein, putative [Entamoeba histolytica HM-1:IMSS]	KSVTQQK	818.473055	2	2.0055
gi|56472345|gb|EAL49836.1| dipeptidyl-peptidase, putative [Entamoeba histolytica HM-1:IMSS]	DIMNDKK	863.429131	2	1.9167
gi|56472364|gb|EAL49854.1| adenylyl cyclase-associated protein, putative [Entamoeba histolytica HM-1:IMSS]	TPAPYVADLIPGSEFYTNK	2083.038267	2	1.659
gi|56472365|gb|EAL49855.1| calreticulin, putative [Entamoeba histolytica HM-1:IMSS]	AGTIYDDILITDDIEEAEK	2124.023063	2	4.668
	AGTIYDDILITDDIEEAEK	2124.023063	2	4.4018
	AGTIYDDILITDDIEEAEK	2124.023063	2	4.3903
	AGTIYDDILITDDIEEAEK	2124.023063	2	4.2883
	FDEDWDMLAPK	1366.598383	2	4.4646
	FTPESEYNIMFGPDVCGGSK	2234.951851	2	4.5895

	FTPESEYNIMFGPDVCGGSK	2234.951851	2	3.8549
	FTPESEYNIMFGPDVCGGSK	2234.951851	2	3.6171
	KPEEWNDEDDGEWEAPTIENPEYK	2920.243183	3	4.268
gi|56472384|gb|EAL49874.1| hypothetical protein 33.t00026 [Entamoeba histolytica HM-1:IMSS]	TLLFPK	718.449789	2	1.5876
gi|56472450|gb|EAL49934.1| conserved hypothetical protein [Entamoeba histolytica HM-1:IMSS]	NTKVECVVKQLLQVNIKAK	2212.263124	2	1.8471
gi|56472462|gb|EAL49946.1| hypothetical protein 31.t00016 [Entamoeba histolytica HM-1:IMSS]	GKTWDDFNKDYMMLIK	2004.955787	3	1.8121
gi|56472496|gb|EAL49978.1| villidin, putative [Entamoeba histolytica HM-1:IMSS]	EPTLLTCEK	1090.523426	1	2.2039
	FFDTEPSITSLFK	1531.767912	2	2.3567
gi|56472516|gb|EAL49998.1| protein disulfide isomerase, putative [Entamoeba histolytica HM-1:IMSS]	FVLENVPAK	1016.57751	2	2.5625
	FVLENVPAK	1016.57751	1	2.3037
	GFPTVLVFNGEESK	1523.774055	2	2.3805
	GFPTVLVFNGEESK	1523.774055	2	2.036
	GFPTVLVFNGEESKK	1651.869018	2	1.8053
gi|56472520|gb|EAL50002.1| hypothetical protein 30.t00055 [Entamoeba histolytica HM-1:IMSS]	YVFTTFYTK	1169.587741	2	1.7548
gi|56472550|gb|EAL50029.1| galactose-inhibitable lectin 170 kda subunit [Entamoeba histolytica HM-1:IMSS]	CKVSNCTEDLVR	1480.645359	2	2.0616
	YEVDWDNVPVDEQR	1763.787126	2	4.2908
	YEVDWDNVPVDEQR	1763.787126	2	4.0492
	YEVDWDNVPVDEQR	1763.787126	2	2.5566
	YTAGTGQVDPLINIAK	1660.890472	2	4.6745
	YTAGTGQVDPLINIAK	1660.890472	2	4.6233
	YTAGTGQVDPLINIAK	1660.890472	2	3.8258
gi|56472561|gb|EAL50040.1| calcium-binding protein, putative [Entamoeba histolytica HM-1:IMSS]	KGNVVMK	775.4494734	2	1.6277
gi|56472595|gb|EAL50068.1| hypothetical protein 28.t00048 [Entamoeba histolytica HM-1:IMSS]	ENIKTVTQAVEELVHLDK	2066.112817	3	1.879
gi|56472627|gb|EAL50097.1| hypothetical protein 28.t00011 [Entamoeba histolytica HM-1:IMSS]	SEKDIVYFGM*NHGDVIDK	2082.981201	2	1.6366
gi|56472629|gb|EAL50099.1| 60S ribosomal protein L23a, putative [Entamoeba histolytica HM-1:IMSS]	FPLATETALK	1090.61429	1	2.0568
	FPLATETALK	1090.61429	2	2.0213
	FPLATETALK	1090.61429	1	1.8975
	LAPDVEAMEVATK	1373.698097	2	3.581
	LAPDVEAMEVATK	1373.698097	2	3.5587
	LAPDVEAMEVATK	1373.698097	2	2.9532
	LAPDVEAMEVATK	1373.698097	2	2.6403
	LAPDVEAMEVATK	1373.698097	1	1.8429
	MDEFSIIK	982.491407	2	2.3224
	MDEFSIIK	982.491407	2	2.263
gi|56472660|gb|EAL50126.1| translation initiation factor eIF-2B epsilon subunit, putative [Entamoeba histolytica HM-1:IMSS]	IKISTIIQNRYK	1476.889687	2	1.5119
gi|56472679|gb|EAL50145.1| DHHC-type zinc finger protein, putative [Entamoeba histolytica HM-1:IMSS]	YVRLLFGGKQWGMIK	1796.004001	2	2.1703
gi|56472722|gb|EAL50186.1| hypothetical protein 26.t00050 [Entamoeba histolytica HM-1:IMSS]	EESIIK	718.398159	1	1.9025
gi|56472725|gb|EAL50189.1| conserved hypothetical protein [Entamoeba histolytica HM-1:IMSS]	M*IDDCVSYIPSTEYK	1836.782688	3	1.9526
gi|56472738|gb|EAL50202.1| hypothetical protein 26.t00066 [Entamoeba histolytica HM-1:IMSS]	GKKIAK	644.4453744	1	1.5107
gi|56472740|gb|EAL50203.1| RNA-binding protein, putative [Entamoeba histolytica HM-1:IMSS]	EM*KKIK	792.465885	2	1.5697
gi|56472751|gb|EAL50213.1| 60S ribosomal protein L32, putative [Entamoeba histolytica HM-1:IMSS]	GTNLLPK	742.4457694	1	2.0857
	GTNLLPK	742.4457694	1	1.6943
	KYAAEISHAVSAK	1374.737614	3	2.9328
	KYAAEISHAVSAK	1374.737614	2	1.9681
	NTTDLEMIMMQNR	1596.717862	3	2.705
gi|56472775|gb|EAL50234.1| Rab geranylgeranyltransferase alpha subunit, putative [Entamoeba histolytica HM-1:IMSS]	KIDSTIELLSNEEVK	1717.921848	2	1.6061
gi|56472782|gb|EAL50241.1| Oxysterol binding protein, putative [Entamoeba histolytica HM-1:IMSS]	KPYNPILGEVFRCMWEDPK	2379.140962	2	1.6341
gi|56472783|gb|EAL50242.1| conserved hypothetical protein [Entamoeba histolytica HM-1:IMSS]	SQLENFKNK	1107.579312	2	1.8664
	CSKELIEVKNEK	1476.751205	2	2.4727
	CSKELIEVKNEK	1476.751205	2	2.1055
gi|56472807|gb|EAL50266.1| histone H4 [Entamoeba histolytica HM-1:IMSS]	INGAVYDETR	1137.553484	2	2.3397
	QFLEQVIR	1032.583658	2	2.585

	QFLEQVIR	1032.583658	2	2.2809
	TVTAMDVVYALK	1310.702453	2	3.7478
	TVTAMDVVYALK	1310.702453	2	3.5676
	TVTAMDVVYALK	1310.702453	2	3.3627
	TVTAMDVVYALK	1310.702453	2	3.3521
gi|56472811|gb|EAL50270.1| hypothetical protein 25.t00038 [Entamoeba histolytica HM-1:IMSS]	GFLCWHGTSWNNAESIMR	2165.942954	3	1.9853
gi|56472825|gb|EAL50283.1| 60S ribosomal protein L19, putative [Entamoeba histolytica HM-1:IMSS]	ALMESIFK	938.501577	2	2.6608
	ALMESIFK	938.501577	2	2.4917
	ALMESIFK	938.501577	1	1.5492
	KVWLDPNETPEISLANSR	2069.066221	3	2.3114
	TLWIIR	801.498136	2	1.625
	VWLDPNETPEISLANSR	1940.971258	2	4.6743
	VWLDPNETPEISLANSR	1940.971258	2	4.5487
	VWLDPNETPEISLANSR	1940.971258	2	3.8513
	VWLDPNETPEISLANSR	1940.971258	2	3.0676
gi|56472829|gb|EAL50287.1| hypothetical protein 24.t00011 [Entamoeba histolytica HM-1:IMSS]	EELEKKIK	1016.59864	1	1.7298
gi|56472842|gb|EAL50300.1| RNA polymerase III subunit, putative [Entamoeba histolytica HM-1:IMSS]	ESDLKAVFDEM*VK	1526.741799	2	1.5592
gi|56472855|gb|EAL50313.1| hypothetical protein 24.t00040 [Entamoeba histolytica HM-1:IMSS]	DKWLNLIDKVLLK	1597.967593	2	1.9317
	GETSIDIDTLIEEK	1562.779593	2	4.2061
	GETSIDIDTLIEEK	1562.779593	2	4.0211
	GETSIDIDTLIEEK	1562.779593	2	3.9371
	GETSIDIDTLIEEK	1562.779593	2	3.7974
	GETSIDIDTLIEEK	1562.779593	2	3.6399
gi|56472869|gb|EAL50327.1| hypothetical protein 24.t00055 [Entamoeba histolytica HM-1:IMSS]	YFLSQM*QHSSQTLFK	1860.896028	2	2.1094
gi|56472875|gb|EAL50333.1| hypothetical protein 24.t00061 [Entamoeba histolytica HM-1:IMSS]	ELPGMLFCNSLAKDVLSK	2022.018407	3	2.0567
gi|56472893|gb|EAL50348.1| glutamine synthetase, putative [Entamoeba histolytica HM-1:IMSS]	KFAPTTLK	905.545481	2	1.5288
gi|56472943|gb|EAL50397.1| hypothetical protein 23.t00026 [Entamoeba histolytica HM-1:IMSS]	NTSVAPIIIK	1055.645934	1	1.7403
gi|56472945|gb|EAL50399.1| glutamyl-tRNA synthetase, putative [Entamoeba histolytica HM-1:IMSS]	NNSLEKNMK	1077.535732	2	1.6221
gi|56472956|gb|EAL50409.1| hypothetical protein 22.t00043 [Entamoeba histolytica HM-1:IMSS]	KVTESEGPSPASVPNK	1626.833379	2	1.7065
gi|56472970|gb|EAL50423.1| hypothetical protein 22.t00057 [Entamoeba histolytica HM-1:IMSS]	DEHKRREHESR	1478.720726	2	1.5576
gi|56472997|gb|EAL50447.1| exonuclease, putative [Entamoeba histolytica HM-1:IMSS]	M*GVPSLFAWVRQICPGYVK	2224.120214	3	1.758
gi|56473040|gb|EAL50488.1| hypothetical protein 21.t00032 [Entamoeba histolytica HM-1:IMSS]	LIGM*KKAIESTISLEK	1777.015072	2	1.7134
gi|56473049|gb|EAL50497.1| Rho GTPase activating protein, putative [Entamoeba histolytica HM-1:IMSS]	TDETISYLKQIKKLFEK	2084.163799	2	1.5606
gi|56473090|gb|EAL50536.1| Rab family GTPase [Entamoeba histolytica HM-1:IMSS] gi|56790126|dbj|BAD82859.1| EhRab>	IIVIGEPSVGK	1111.672154	2	2.254
	YSDGILTSAK	1054.541542	2	1.5268
gi|56473099|gb|EAL50545.1| conserved hypothetical protein [Entamoeba histolytica HM-1:IMSS]	ISKLGGYENK	1108.599718	2	1.6987
gi|56473112|gb|EAL50558.1| hypothetical protein 20.t00054 [Entamoeba histolytica HM-1:IMSS]	ATGKAWEK	890.4730474	2	1.8039
gi|56473144|gb|EAL50587.1| conserved hypothetical protein [Entamoeba histolytica HM-1:IMSS]	QIEKLGSK	902.5305724	2	1.6586
gi|56473173|gb|EAL50616.1| hypothetical protein 19.t00056 [Entamoeba histolytica HM-1:IMSS]	YGYGNTFEFK	1225.552424	2	3.0935
gi|56473176|gb|EAL50619.1| hypothetical protein 19.t00059 [Entamoeba histolytica HM-1:IMSS]	HGCKALPYVQVQSIKR	1884.005798	2	1.6121
gi|56473196|gb|EAL50638.1| 60S ribosomal protein L18, putative [Entamoeba histolytica HM-1:IMSS]	FDELALK	835.455998	2	2.3652
	FDELALK	835.455998	2	2.2765
	FDELALK	835.455998	1	1.8219
	FDELALK	835.455998	1	1.8028
	LYGFLAR	839.4774034	2	2.1019
gi|56473197|gb|EAL50639.1| hypothetical protein 19.t00012 [Entamoeba histolytica HM-1:IMSS]	KEDDFPK	878.425427	2	1.6075
gi|56473207|gb|EAL50646.1| Rab family GTPase [Entamoeba histolytica HM-1:IMSS] gi|56790112|dbj|BAD82852.1| EhRab>	SYFVCSAKTGDGCK	1579.64504	2	1.6416
gi|56473243|gb|EAL50682.1| hypothetical protein 18.t00063 [Entamoeba histolytica HM-1:IMSS]	DPNTQKLNQVFQDKK	1802.939543	2	1.6574
gi|56473288|gb|EAL50722.1| hypothetical protein 17.t00018 [Entamoeba histolytica HM-1:IMSS]	QLEEIQK	887.483276	2	2.5985
gi|56473300|gb|EAL50734.1| hypothetical protein 17.t00031 [Entamoeba histolytica HM-1:IMSS]	NIDKVSNSKTSINSVAKK	1933.071326	3	1.6689
gi|56473301|gb|EAL50735.1| 40S ribosomal protein S13, putative [Entamoeba histolytica HM-1:IMSS]	DTHGVGLVK	925.5101638	2	2.1876
	GIAPSQIGAIMR	1213.67217	2	3.661

	GIAPSQIGAIMR	1213.67217	1	2.3132
	IPEDLYFLMK	1268.659525	2	3.4224
	IPEDLYFLMK	1268.659525	2	3.0358
	IPEDLYFLMK	1268.659525	1	2.9673
	IPEDLYFLMK	1268.659525	1	2.9601
	IPEDLYFLMK	1268.659525	2	2.7954
	IPEDLYFLMK	1268.659525	2	2.3978
	IPEDLYFLMK	1268.659525	2	2.3911
	KGIAPSQIGAIMR	1341.767133	3	3.1908
	LAGLAPK	669.4293904	1	1.8042
	LAGLAPK	669.4293904	1	1.5927
	LAGLAPK	669.4293904	1	1.5507
	YHLILIESK	1115.645934	2	3.1163
	YHLILIESK	1115.645934	1	2.7038
	YHLILIESK	1115.645934	2	2.6406
	YHLILIESK	1115.645934	1	2.6032
gi|56473302|gb|EAL50736.1| hypothetical protein 17.t00033 [Entamoeba histolytica HM-1:IMSS]	DKLKFINK	1005.609144	2	1.7939
gi|56473325|gb|EAL50759.1| galactose-inhibitable lectin 35 kda subunit precursor [Entamoeba histolytica HM-1:IMSS]	HFYLPYR	995.509764	2	2.0967
gi|56473331|gb|EAL50765.1| protein with RhoGEF and ArfGAP domains [Entamoeba histolytica HM-1:IMSS]	EDKTIRNCFKNLEM*IVK	2154.084209	2	1.8106
gi|56473334|gb|EAL50768.1| Ras GTPase activating protein, putative [Entamoeba histolytica HM-1:IMSS]	LEELLEMR	1032.53941	2	2.0045
	VIANSEAQPVTLEDVLTETEK	2286.171132	2	2.384
gi|56473363|gb|EAL50792.1| hypothetical protein 16.t00009 [Entamoeba histolytica HM-1:IMSS]	KVIQSDGSVKVALSSLRK	1915.133533	2	1.9169
gi|56473368|gb|EAL50797.1| Gal/GalNAc lectin heavy subunit [Entamoeba histolytica HM-1:IMSS]	YTAGQGQVDPLVNIAK	1673.885721	2	3.9784
gi|56473474|gb|EAL50899.1| 40S ribosomal protein S9, putative [Entamoeba histolytica HM-1:IMSS]	EENLLER	902.45779	2	1.5346
	FDAELK	722.371934	2	1.5978
	LEFVLALK	932.581532	2	2.6088
	LEFVLALK	932.581532	1	2.1392
	LHNLGILPR	1032.631278	2	2.9581
	LHNLGILPR	1032.631278	3	2.8519
	LHNLGILPR	1032.631278	3	2.7124
	LHNLGILPR	1032.631278	2	2.7012
	LHNLGILPR	1032.631278	1	2.1205
	LHNLGILPR	1032.631278	1	1.8294
	LLEGAALLR	955.5934964	2	3.5229
	LLEGAALLR	955.5934964	2	2.9427
	LLEGAALLR	955.5934964	1	1.8217
	LLEGAALLR	955.5934964	1	1.6679
	LQTLVYR	892.52508	2	2.1738
	LQTLVYR	892.52508	1	1.5944
	LVGQFGLK	861.5192708	2	2.4529
	LVGQFGLK	861.5192708	2	2.0695
	LVGQFGLK	861.5192708	1	1.7666
	QVVDVPSFLVR	1258.715411	2	2.9588
	QVVDVPSFLVR	1258.715411	2	2.879
	QVVDVPSFLVR	1258.715411	2	2.8109
	QVVDVPSFLVR	1258.715411	2	2.2537
	QVVDVPSFLVR	1258.715411	1	1.7536
	QVVDVPSFLVR	1258.715411	2	1.5569
	VESEPLIQLADNTPLTNPEINGR	2520.294043	2	4.0809
	VESEPLIQLADNTPLTNPEINGR	2520.294043	2	3.2423
	VESEPLIQLADNTPLTNPEINGR	2520.294043	2	3.0495
gi|56473494|gb|EAL50917.1| hypothetical protein 14.t00049 [Entamoeba histolytica HM-1:IMSS]	VTFGLKDQKFLLKIDGLGK	2120.247799	2	1.754

gi|56473507|gb|EAL50929.1| hypothetical protein 14.t00005 [Entamoeba histolytica HM-1:IMSS]	MMHSIFMTLQSIEM*DKK	2085.985107	2	2.0413
gi|56473526|gb|EAL50948.1| hypothetical protein 14.t00037 [Entamoeba histolytica HM-1:IMSS]	IELSKKKK	973.640454	2	2.2229
	IELSKKKK	973.640454	2	1.701
	IELSKKKK	973.640454	2	1.6558
gi|56473527|gb|EAL50949.1| hypothetical protein 14.t00038 [Entamoeba histolytica HM-1:IMSS]	GYSICQLLELAGKTK	1680.877473	3	1.677
gi|56473530|gb|EAL50952.1| hypothetical protein 14.t00041 [Entamoeba histolytica HM-1:IMSS]	GYTNDFLKIITNSFIK	1874.005843	3	1.6385
gi|56473547|gb|EAL50966.1| phosphatidylinositol-4-phosphate 5-kinase, putative [Entamoeba histolytica HM-1:IMSS]	DLDFVEQK	993.488756	2	1.6828
gi|56473554|gb|EAL50973.1| carbohydrate degrading enzyme, putative [Entamoeba histolytica HM-1:IMSS]	NNATATFFMLGNK	1428.694016	2	1.8221
gi|56473575|gb|EAL50994.1| hypothetical protein 13.t00037 [Entamoeba histolytica HM-1:IMSS]	KGNTVLHYLCEIGINKR	2015.064035	2	1.5234
gi|56473577|gb|EAL50996.1| hypothetical protein 13.t00039 [Entamoeba histolytica HM-1:IMSS]	TIQGVILKNPGM*K	1414.809752	2	1.6401
gi|56473598|gb|EAL51017.1| protein kinase, putative [Entamoeba histolytica HM-1:IMSS]	LSLRPPPNDPRR	1417.802269	2	1.6172
gi|56473603|gb|EAL51022.1| 60S ribosomal protein L24, putative [Entamoeba histolytica HM-1:IMSS]	SFTFINK	856.456342	1	1.7417
	SFTFINK	856.456342	2	1.6643
	SFTFINK	856.456342	1	1.6419
	SFTFINK	856.456342	2	1.5877
	SLMQFLR	894.486595	2	1.8437
	SLMQFLR	894.486595	2	1.8253
gi|56473612|gb|EAL51030.1| hypothetical protein 13.t00006 [Entamoeba histolytica HM-1:IMSS]	ALADAITAVTK	1073.620104	2	3.7018
	ALADAITAVTK	1073.620104	2	3.5331
	ALADAITAVTK	1073.620104	1	2.0373
	ALADAITAVTK	1073.620104	1	1.865
gi|56473639|gb|EAL51055.1| conserved hypothetical protein [Entamoeba histolytica HM-1:IMSS]	AEQEANSM*EAEVKEINK	1935.897527	2	1.7295
	GGKVNDIKAM*LK	1289.725688	3	1.6403
gi|56473649|gb|EAL51064.1| hypothetical protein 12.t00001 [Entamoeba histolytica HM-1:IMSS]	FIEDNQAPSDVEEIK	1733.822853	2	1.9557
gi|56473652|gb|EAL51067.1| Rab GTPase activating protein, putative [Entamoeba histolytica HM-1:IMSS]	KIDQDAVK	916.509825	2	2.0444
gi|56473674|gb|EAL51089.1| hypothetical protein 12.t00030 [Entamoeba histolytica HM-1:IMSS]	NLYLLPIK	973.608081	2	2.1236
gi|56473676|gb|EAL51091.1| Rho GTPase activating protein, putative [Entamoeba histolytica HM-1:IMSS]	RLEREQKEK	1215.680413	3	1.6134
gi|56473703|gb|EAL51115.1| hypothetical protein 11.t00058 [Entamoeba histolytica HM-1:IMSS]	NNNEMMVEEGCSNEIIKR	2166.936202	2	2.2408
gi|56473776|gb|EAL51181.1| hypothetical protein 10.t00070 [Entamoeba histolytica HM-1:IMSS]	KDIVVIDDLSYGLIMAK	1893.040179	2	1.5619
gi|56473789|gb|EAL51193.1| hypothetical protein 10.t00054 [Entamoeba histolytica HM-1:IMSS]	HVPQEAMPTDPNWFK	1796.84247	2	2.8812
gi|56473794|gb|EAL51197.1| structural maintenance of chromosomes protein [Entamoeba histolytica HM-1:IMSS]	YEEGIQKEQEEK	1509.706752	2	1.6346
gi|56473807|gb|EAL51210.1| conserved hypothetical protein [Entamoeba histolytica HM-1:IMSS]	VEAEKRAK	930.536708	2	1.8451
gi|56473808|gb|EAL51211.1| protein kinase, putative [Entamoeba histolytica HM-1:IMSS]	EIVAPM*K	803.434251	2	1.5653
gi|56473822|gb|EAL51224.1| actin [Entamoeba histolytica HM-1:IMSS]	AASSSELEK	921.452401	1	1.7035
	AASSSELEK	921.452401	1	1.5223
	AASSSELEK	921.452401	1	1.5144
	DLTDYLM*K	1014.482325	2	2.3766
	DLTDYLM*K	1014.482325	2	1.7491
	DLTDYLM*K	1014.482325	1	1.5861
	DLTDYLMK	998.486312	2	2.7099
	DLTDYLMK	998.486312	2	2.1676
	DLTDYLMK	998.486312	1	2.0835
	DLTDYLMK	998.486312	1	1.9625
	DLTDYLMK	998.486312	1	1.7161
	DLYGNIVLSGGTSMYPGINTR	2228.10163	2	4.51
	DLYGNIVLSGGTSMYPGINTR	2228.10163	2	4.1675
	DLYGNIVLSGGTSMYPGINTR	2228.10163	2	2.6235
	EEYDESGPAIVHR	1501.69178	2	2.8523
	EMIQLAPPTM*K	1274.649406	2	2.4796
	EMIQLAPPTM*K	1274.649406	2	2.1732
	EMIQLAPPTMK	1258.653393	2	3.3754
	EMIQLAPPTMK	1258.653393	1	2.9629

	EMIQLAPPTMK	1258.653393	2	2.8051
	EMIQLAPPTMK	1258.653393	1	2.4764
	GYAFTTTAER	1116.53202	2	3.3581
	GYAFTTTAER	1116.53202	1	2.1455
	LDLAGR	644.3726044	2	1.5905
	LDLAGR	644.3726044	2	1.5628
	SYELPDGQVITVGNER	1776.87629	2	4.1403
	SYELPDGQVITVGNER	1776.87629	2	3.8661
	SYELPDGQVITVGNER	1776.87629	2	3.8198
	SYELPDGQVITVGNER	1776.87629	2	3.5552
	SYELPDGQVITVGNER	1776.87629	2	3.0934
	SYELPDGQVITVGNER	1776.87629	2	3.0881
	SYELPDGQVITVGNER	1776.87629	2	2.6512
	TTGIVMDSGDGVSHTVPIYEGFSLPHAILR	3169.598709	3	4.2657
	VAPEEHPVLLTEAPM*NPK	1988.016842	2	3.3151
	VAPEEHPVLLTEAPM*NPK	1988.016842	3	3.0581
	VAPEEHPVLLTEAPM*NPK	1988.016842	3	2.5122
	VAPEEHPVLLTEAPMNPK	1972.020829	2	4.0782
	VAPEEHPVLLTEAPMNPK	1972.020829	2	3.7486
	VAPEEHPVLLTEAPMNPK	1972.020829	2	3.737
	VAPEEHPVLLTEAPMNPK	1972.020829	2	3.5707
	VIAPPER	781.456666	2	1.8807
	VIAPPERK	909.551629	2	1.6578
gi|56473842|gb|EAL51244.1| protein kinase, putative [Entamoeba histolytica HM-1:IMSS]	LGEGGFGIVYK	1139.609549	2	1.5036
gi|56473862|gb|EAL51261.1| hypothetical protein 9.t00023 [Entamoeba histolytica HM-1:IMSS]	IADKTNPFIIM*R	1434.778447	3	2.1483
gi|56473872|gb|EAL51271.1| hypothetical protein 9.t00033 [Entamoeba histolytica HM-1:IMSS]	ILM*SHLQVTVMSNR	1644.857128	3	1.7489
gi|56473881|gb|EAL51280.1| conserved hypothetical protein [Entamoeba histolytica HM-1:IMSS]	ELQALAIQSM*SVIYRKFNK	2255.222772	2	1.656
gi|56473888|gb|EAL51287.1| conserved hypothetical protein [Entamoeba histolytica HM-1:IMSS]	INESQIQK	959.515649	2	1.7138
gi|56473892|gb|EAL51291.1| ADP-ribosylation factor, putative [Entamoeba histolytica HM-1:IMSS]	ILMVGLDAAGK	1087.617999	2	3.3892
	ILMVGLDAAGK	1087.617999	2	2.6728
	ILMVGLDAAGK	1087.617999	2	1.5763
gi|56473961|gb|EAL51355.1| actin, putative [Entamoeba histolytica HM-1:IMSS]	AGFAGDDAPR	976.4482928	2	2.0835
	AVFPSIVGR	945.5516414	2	2.0869
	AVFPSIVGRPR	1198.705516	2	2.7094
	DAYVGDEAQSK	1182.52734	2	3.3426
	HVSVMAGM*GQK	1160.556189	2	2.1327
	HVSVMAGM*GQK	1160.556189	2	1.8472
gi|56473972|gb|EAL51366.1| copine, putative [Entamoeba histolytica HM-1:IMSS]	YNEITPSVQMSGPTNFAPLINK	2421.211901	2	2.7134
gi|56473994|gb|EAL51388.1| chorein, putative [Entamoeba histolytica HM-1:IMSS]	ENIGPIVSEVITRSDGTK	1915.013128	2	1.5313
gi|56474005|gb|EAL51399.1| conserved hypothetical protein [Entamoeba histolytica HM-1:IMSS]	EFSEKANTLMNGFQGHLR	2079.007654	2	1.5512
gi|56474013|gb|EAL51407.1| Vesicle-fusing ATPase, putative [Entamoeba histolytica HM-1:IMSS]	AFM*SRM*FPSETIKKLGIK	2116.13155	2	1.9644
gi|56474044|gb|EAL51435.1| 40S ribosomal protein S23, putative [Entamoeba histolytica HM-1:IMSS]	DGSLNYVNENDEVLISGFGR	2198.036052	2	5.4168
	DGSLNYVNENDEVLISGFGR	2198.036052	2	5.1969
	DGSLNYVNENDEVLISGFGR	2198.036052	2	4.9989
	DGSLNYVNENDEVLISGFGR	2198.036052	2	4.5735
	DGSLNYVNENDEVLISGFGR	2198.036052	3	3.5601
	DGSLNYVNENDEVLISGFGR	2198.036052	2	3.0925
	DGSLNYVNENDEVLISGFGR	2198.036052	2	2.9219
	GHAVGDIPGVR	1077.579977	2	2.1743
	ITAFVPR	803.477401	2	1.8691
gi|56474045|gb|EAL51436.1| Rab family GTPase [Entamoeba histolytica HM-1:IMSS] gi|50978604|dbj|BAD34969.1| EhRab7	IIILGDSGVGK	1071.640856	2	2.8153
gi|56474053|gb|EAL51443.1| hypothetical protein 7.t00065 [Entamoeba histolytica HM-1:IMSS]	EGTLVYKSYDVVPEVEDLR	2211.117975	2	2.4321

gi|56474058|gb|EAL51447.1| hypothetical protein 7.t00003 [Entamoeba histolytica HM-1:IMSS]	KRQGTPEYEMKQIEDDEK	2224.055047	2	1.9279
gi|56474066|gb|EAL51455.1| hypothetical protein 7.t00052 [Entamoeba histolytica HM-1:IMSS]	SLLASGLSEEEAEK	1462.727183	2	2.9935
gi|56474082|gb|EAL51470.1| RNA polymerase subunit Rpb8 [Entamoeba histolytica HM-1:IMSS]	DGSPMDIHQGYSDNFGK	1867.791586	2	2.5257
gi|56474090|gb|EAL51478.1| conserved hypothetical protein [Entamoeba histolytica HM-1:IMSS]	RKQVNANIIYNEYIQDK	2109.108734	2	1.5542
gi|56474131|gb|EAL51514.1| hypothetical protein 6.t00007 [Entamoeba histolytica HM-1:IMSS]	NIKHISVLGGGYK	1385.78998	3	1.8477
gi|56474132|gb|EAL51515.1| hypothetical protein 6.t00008 [Entamoeba histolytica HM-1:IMSS]	IEEYLQTLMEGDEEMKKK	2214.066856	3	3.3737
	KVDYYLNKTR	1299.705565	2	1.6634
gi|56474175|gb|EAL51558.1| phospholipid-transporting P-type ATPase, putative [Entamoeba histolytica HM-1:IMSS]	EEFTTKKLVEM*DEEIDK	2100.006399	3	1.9843
gi|56474198|gb|EAL51581.1| hypothetical protein 6.t00082 [Entamoeba histolytica HM-1:IMSS]	M*LPINNYTEIK	1351.693715	2	2.2704
gi|56474240|gb|EAL51620.1| hypothetical protein 5.t00080 [Entamoeba histolytica HM-1:IMSS]	HLFGLHDIVQK	1306.726636	2	1.6619
gi|56474246|gb|EAL51626.1| conserved hypothetical protein [Entamoeba histolytica HM-1:IMSS]	AFNELIEFR	1138.589138	2	3.2498
	AFNELIEFR	1138.589138	2	3.1122
	AFNELIEFR	1138.589138	1	2.5587
	DGEVLNVNFASIKPLYK	1907.027307	2	3.8776
	DIAEELLK	930.514242	2	2.4487
	DIAEELLK	930.514242	1	2.4077
	DIAEELLK	930.514242	1	1.9583
	DMLIGNNFK	1051.524096	2	2.1084
	EGLVVVDER	1015.541856	2	3.2994
	EGSLFVSGEEAK	1252.605603	2	1.67
	ELDFYVMK	1044.507047	1	1.8871
	ENMTQIYQFDTQK	1645.752653	2	2.7531
	FEVVSEATENVPK	1448.726766	2	4.8345
	FEVVSEATENVPK	1448.726766	2	4.5759
	FVNFDQAAEDAAK	1425.66449	2	4.1674
	FVNFDQAAEDAAK	1425.66449	2	3.317
	FVNFDQAAEDAAK	1425.66449	2	3.0809
	GGNILFIGK	918.5407372	1	1.5711
	GGNILFIGK	918.5407372	1	1.5549
	HFHDNSEWGVDLVAAQLQAK	2265.104724	2	2.01
	KGEETVTEVYYDVYADK	2008.938604	2	5.556
	KVDAPGQFIIETPER	1699.901368	2	3.7746
	KVDAPGQFIIETPER	1699.901368	2	3.1688
	NGLLLATK	829.5141834	2	2.1209
	NGYVTISTFEENM*ESVK	1963.896476	2	1.8894
	NGYVTISTFEENMESVK	1947.900463	2	3.8511
	NGYVTISTFEENMESVK	1947.900463	2	3.1297
	TNIAIPLLK	982.629545	2	2.821
	TNIAIPLLK	982.629545	2	2.7572
	TNIAIPLLK	982.629545	1	2.3173
	TVGLVGLK	786.5083718	2	1.6592
	TYKPVPITNYSNLDNVK	1966.028034	2	2.9255
	VDAPGQFIIETPER	1571.806405	2	3.0839
	VTGIITVDK	945.5615284	2	2.2052
	VYLTGFDGEK	1128.557175	2	2.3941
	YNVSSEIEQMK	1327.619867	2	3.7677
	YNVSSEIEQMK	1327.619867	2	3.7465
gi|56474266|gb|EAL51645.1| myosin heavy chain [Entamoeba histolytica HM-1:IMSS]	DIQQVFIGK	1047.583326	1	1.803
	FQINAPSSVAQEEEK	1676.812632	2	2.4732
	ISELEEQVNELESR	1674.818112	2	3.3985
	SLAAQVAATK	959.552034	1	1.5708
	YFVDSTVLK	1071.572101	2	1.7901

gi|56474341|gb|EAL51715.1| hypothetical protein 4.t00100 [Entamoeba histolytica HM-1:IMSS]	ELFAKILAMQNKSR	1648.920335	2	1.9108
	ELFAKILAMQNKSR	1648.920335	2	1.6981
gi|56474342|gb|EAL51716.1| hypothetical protein 4.t00101 [Entamoeba histolytica HM-1:IMSS]	ELYNILTIPK	1203.698354	1	1.6773
gi|56474395|gb|EAL51767.1| 40S ribosomal protein S7, putative [Entamoeba histolytica HM-1:IMSS]	ATVIFIPLR	1029.645529	2	2.9695
	KVTISK	675.439963	1	1.5229
	VHATLVHK	904.536313	2	2.5895
	VHATLVHK	904.536313	2	2.0885
gi|56474429|gb|EAL51799.1| chromosome segregation protein, putative [Entamoeba histolytica HM-1:IMSS]	LGKM*NGLSQSEDVIGGITIAMK	2278.179263	2	1.5347
	RRKFEEANR	1205.649781	3	1.5221
gi|56474481|gb|EAL51848.1| protein kinase, putative [Entamoeba histolytica HM-1:IMSS]	DQVFSQILDGEVLFPK	1834.958559	2	1.6637
gi|56474491|gb|EAL51858.1| hypothetical protein 3.t00139 [Entamoeba histolytica HM-1:IMSS]	YVTYCELSK	1162.523436	1	1.7667
gi|56474515|gb|EAL51880.1| cell division cycle protein 48, putative [Entamoeba histolytica HM-1:IMSS]	NSPAIIFIDEIDSIAPKR	1999.085893	2	1.5308
gi|56474531|gb|EAL51896.1| glycosyltransferase, putative [Entamoeba histolytica HM-1:IMSS]	EVSINNTNTIAR	1331.691383	2	1.5369
gi|56474534|gb|EAL51899.1| hypothetical protein 3.t00026 [Entamoeba histolytica HM-1:IMSS]	KFSLEVSK	937.535331	2	1.5058
	LIVPSDLK	884.545157	2	1.8916
	PSLNPDWNETFNFQVSPK	2120.008372	2	3.8948
	SDPYVIIK	934.524422	2	1.5652
	SDPYVIIK	934.524422	2	1.5582
	YEVFIEK	927.482213	1	2.2961
	YEVFIEK	927.482213	2	2.0751
	YEVFIEK	927.482213	1	1.9713
gi|56474536|gb|EAL51901.1| eukaryotic initiation factor 4A, putative [Entamoeba histolytica HM-1:IMSS]	NLSQYLDIK	1093.588814	2	1.6478
gi|56474538|gb|EAL51903.1| hypothetical protein 3.t00030 [Entamoeba histolytica HM-1:IMSS]	DNKM*VKELKEQM*SEFITK	2230.111593	2	1.7055
	FNDGRSNLDEAFIKR	1781.892939	2	1.751
	FNDGRSNLDEAFIKR	1781.892939	2	1.6704
gi|56474542|gb|EAL51907.1| Viral A-type inclusion protein repeat, putative [Entamoeba histolytica HM-1:IMSS]	GKIDNMMLEAM*ELVK	1737.859478	3	2.175
	IKSEKNDVQKEK	1445.795848	2	2.3947
	IKSEKNDVQKEK	1445.795848	2	1.8057
	IKSEKNDVQKEK	1445.795848	2	1.5357
gi|56474551|gb|EAL51916.1| TFIID subunit, putative [Entamoeba histolytica HM-1:IMSS]	TVETKEFEIGK	1280.673265	2	1.6188
gi|56474597|gb|EAL51960.1| hypothetical protein 2.t00121 [Entamoeba histolytica HM-1:IMSS]	TPKSIREPKR	1211.721893	2	1.5051
gi|56474611|gb|EAL51973.1| casein kinase 1, putative [Entamoeba histolytica HM-1:IMSS]	IQEVHDRFFIHR	1596.839373	3	1.7403
gi|56474617|gb|EAL51979.1| guanine deaminase, putative [Entamoeba histolytica HM-1:IMSS]	DLMKKLGQLAQER	1529.846828	3	1.8716
gi|56474648|gb|EAL52010.1| Rho guanine nucleotide exchange factor, putative [Entamoeba histolytica HM-1:IMSS]	IAGKSVNIEDGESLKK	1687.922521	2	1.5723
gi|56474653|gb|EAL52015.1| hypothetical protein 2.t00091 [Entamoeba histolytica HM-1:IMSS]	LVNAEK	673.387918	2	1.6083
	LVNAEK	673.387918	1	1.5018
gi|56474691|gb|EAL52052.1| BspA-related protein, putative [Entamoeba histolytica HM-1:IMSS]	QCFSGNTK	941.3930924	1	1.5357
gi|56474736|gb|EAL52093.1| 4-alpha-glucanotransferase, putative [Entamoeba histolytica HM-1:IMSS]	FLVNNYNGTYNLK	1559.785275	3	1.7685
gi|56474744|gb|EAL52101.1| WD-40 repeat protein, putative [Entamoeba histolytica HM-1:IMSS]	IHKSKSM*VNIAKPVVMSK	2013.135879	2	1.7644
gi|56474761|gb|EAL52118.1| 40S ribosomal protein S3, putative [Entamoeba histolytica HM-1:IMSS]	ALNVDVLK	871.524746	2	2.141
gi|56474780|gb|EAL52137.1| Ran family GTPase [Entamoeba histolytica HM-1:IMSS]	FEVWDTAGQEK	1309.605914	2	2.7654
gi|56474784|gb|EAL52141.1| cullin, putative [Entamoeba histolytica HM-1:IMSS]	EEKEKVDEKIVK	1473.815905	2	2.7497
gi|56474785|gb|EAL52142.1| hypothetical protein 1.t00142 [Entamoeba histolytica HM-1:IMSS]	NLPETETTVTGQPPINK	1838.949442	2	1.9834
gi|56474825|gb|EAL52181.1| calcium-transporting P-type ATPase, putative [Entamoeba histolytica HM-1:IMSS]	VSPEDKLTVVK	1214.699093	2	1.6615
gi|56474835|gb|EAL52191.1| hypothetical protein 1.t00057 [Entamoeba histolytica HM-1:IMSS]	KEGIENEKRAQTVIM*K	1890.012427	2	1.6545
gi|56474854|gb|EAL52208.1| hsp70 family protein [Entamoeba histolytica HM-1:IMSS]	EDM*NNVMSEILKETK	1796.84159	2	2.2076
	KNDSIKEADKEK	1404.732914	2	1.6895
	KNDSIKEADKEK	1404.732914	2	1.5732
gi|6093867|sp|O15613|R10A_ENTHI 60S ribosomal protein L10a	SSAVSRQGVATILK	1416.816939	3	1.5078
gi|6642731|gb|AAF20265.1| dipeptidyl-peptidase [Entamoeba dispar]	GLASM*TVFPGMTLEESAKK	2013.004253	2	2.0407
gi|7494130|pir||T18292 nicotinamide nucleotide transhydrogenase homolog - Entamoeba histolytica	MWPAPQIQVVSQNTPAK	1894.984393	2	3.3976
	MWPAPQIQVVSQNTPAK	1894.984393	2	3.2734

	MWPAPQIQVVSQNTPAK	1894.984393	2	2.0667
	SPILITQDMVDSMK	1577.791365	2	3.3945
gi|7579069|gb|AAF64243.1| hsp70 BiP [Entamoeba invadens]	QIEQIAQPILAK	1351.79438	2	3.7974
