# Supplementary material for: Entamoeba histolytica Phagocytosis of Human Erythrocytes Involves PATMK, a Member of the Transmembrane Kinase Family
Source: PLoS Pathog. 2008 Jan 18;4(1):e8. doi: 10.1371/journal.ppat.0040008 (PMC2211552; doi:10.1371/journal.ppat.0040008)
Supplement: Supplementary file 2 [file ppat.0040008.st002.pdf]

5 MINUTES

ANNOTATION	PEPTIDE SEQUENCE	MASS	CHARGE	Xcorr
gi|56463309|gb|EAL42451.1| 40S ribosomal protein S6, putative [Entamoeba histolytica HM-1:IMSS]	KGEGEIEGVTNDALPMR	1815.890551	3	3.2363
	KLFNLSQK	977.577854	2	1.9375
	VVSFADTDAVFK	1298.662708	2	3.1812
	VVSFADTDAVFK	1298.662708	2	3.1717
	VVSFADTDAVFK	1298.662708	2	3.1454
	YAAILNR	820.467565	2	2.1179
gi|56463315|gb|EAL42453.1| hypothetical protein 1052.t00002 [Entamoeba histolytica HM-1:IMSS]	DDKEFNK	895.415591	2	1.5046
gi|56463463|gb|EAL42482.1| hypothetical protein 900.t00001 [Entamoeba histolytica HM-1:IMSS]	YQLEEINGIR	1234.642633	2	1.8435
gi|56463710|gb|EAL42562.1| Rab family GTPase [Entamoeba histolytica HM-1:IMSS] gi|50978618|dbj|BAD34976.1| EhRab1	AQIWDTAGQER	1274.612396	2	3.6612
	IVLIGESGVGK	1071.640856	2	2.6042
	SNLLLR	715.446111	2	1.9105
	STIGVEFATR	1080.568415	2	2.5017
gi|56463804|gb|EAL42595.1| hypothetical protein 657.t00001 [Entamoeba histolytica HM-1:IMSS]	ENKDTTKDK	1078.537498	2	2.1101
	NLEIFDITK	1092.593555	2	1.8451
gi|56463851|gb|EAL42617.1| hypothetical protein 640.t00002 [Entamoeba histolytica HM-1:IMSS]	INPVVISINGLINK	1493.905005	2	1.6817
gi|56463872|gb|EAL42625.1| hypothetical protein 631.t00001 [Entamoeba histolytica HM-1:IMSS]	EIEKEVIR	1015.578239	2	2.5487
gi|56463918|gb|EAL42646.1| calcium-binding protein, putative [Entamoeba histolytica HM-1:IMSS]	TSLLLHR	839.509774	2	1.7787
gi|56463923|gb|EAL42650.1| hypothetical protein 612.t00002 [Entamoeba histolytica HM-1:IMSS]	LAEEKKK	845.509096	2	1.9716
gi|56463937|gb|EAL42658.1| hypothetical protein 607.t00001 [Entamoeba histolytica HM-1:IMSS]	TVNSQEAQTR	1133.554555	2	1.5936
gi|56463970|gb|EAL42672.1| CXXC-rich protein [Entamoeba histolytica HM-1:IMSS]	LRLKRLTNSLIHHCEK	2018.122557	3	1.5284
gi|56464043|gb|EAL42708.1| conserved hypothetical protein [Entamoeba histolytica HM-1:IMSS]	VDNDINDQK	1060.490548	2	1.6483
gi|56464167|gb|EAL42771.1| hypothetical protein 535.t00001 [Entamoeba histolytica HM-1:IMSS]	KSNHKIYEIEK	1388.753254	2	1.8255
gi|56464189|gb|EAL42779.1| snoRNA binding protein, putative [Entamoeba histolytica HM-1:IMSS]	EYVEPVVEEEIK	1462.731173	2	2.8104
gi|56464202|gb|EAL42786.1| 40S ribosomal protein S6, putative [Entamoeba histolytica HM-1:IMSS]	FDFEEEK	943.404358	2	1.8419
	IGQEVDASSLGDEFK	1594.759539	2	4.0974
	INVANPATGR	1012.553423	2	2.2858
gi|56464275|gb|EAL42833.1| conserved hypothetical protein [Entamoeba histolytica HM-1:IMSS]	M*NKQQQLQMKIK	1533.825079	2	2.438
gi|56464326|gb|EAL42866.1| serine-rich protein [Entamoeba histolytica HM-1:IMSS]	DTNIYGVFLK	1169.620106	2	2.8069
gi|56464382|gb|EAL42899.1| Truncated pyridine nucleotide transhydrogenase [Entamoeba histolytica HM-1:IMSS]	EANTIQTPELAK	1314.689976	2	2.3661
gi|56464387|gb|EAL42902.1| AIG1 family protein, putative [Entamoeba histolytica HM-1:IMSS]	QSWLPK	758.419562	2	1.7206
gi|56464408|gb|EAL42916.1| protein kinase, putative [Entamoeba histolytica HM-1:IMSS]	KFM*KTNTMNDLLK	1599.824411	3	2.0661
gi|56464476|gb|EAL42961.1| hypothetical protein 472.t00008 [Entamoeba histolytica HM-1:IMSS]	LSQKYGGYKINVSILR	1839.048723	2	1.6766
gi|56464491|gb|EAL42972.1| translation elongation factor EF-1 alpha, putative [Entamoeba histolytica HM-1:IMSS]	FEELLSK	865.466573	2	2.7033
	ISGIGTVPCGR	1116.561559	2	3.126
	IVPTKPLCVEEFAK	1630.86583	2	1.9993
	LPLQDVYK	975.550961	2	2.7688
	NGDSALVK	803.4257734	2	1.9126
	QTVAVGVVK	900.5512974	2	2.1188
	QTVAVGVVK	900.5512974	1	1.5369
	SMEGGEPEYIK	1239.556199	2	3.1544
	STTTGHLIYK	1120.599715	2	2.1714
	YAWVLDNLK	1121.598974	2	3.103
	YAWVLDNLK	1121.598974	2	2.5743
	YAWVLDNLK	1121.598974	2	2.4378
	YYFTIIDAPGHR	1452.727031	3	3.301
	YYFTIIDAPGHR	1452.727031	2	3.2323
gi|56464539|gb|EAL43009.1| hypothetical protein 464.t00003 [Entamoeba histolytica HM-1:IMSS]	NTIIILNKVIIF	1400.887551	2	2.3633
gi|56464614|gb|EAL43068.1| 40S ribosomal protein S15, putative [Entamoeba histolytica HM-1:IMSS]	SPKLKHERLVK	1334.826692	3	1.5697
gi|56464799|gb|EAL43208.1| 40S ribosomal protein S3a, putative [Entamoeba histolytica HM-1:IMSS]	ELTQFVDYLR	1283.663032	2	3.1944

	ELTQFVDYLR	1283.663032	2	2.4749
	FGAYNYK	862.4093844	2	2.1613
	IASEQLK	788.451257	2	1.9324
	IFCIGFTSR	1100.534276	2	2.5361
	LLDQHQDTSAPVEATPVVEEK	2306.151065	3	3.6136
	TTDGHLIR	912.4897604	2	2.1646
	TVGQIVVNK	957.5727614	2	2.7811
	TVGQIVVNK	957.5727614	1	1.6029
	VFSMDNVYIR	1243.613982	2	2.7385
	VFSMDNVYIR	1243.613982	2	1.8582
gi|56464869|gb|EAL43257.1| cortexillin, putative [Entamoeba histolytica HM-1:IMSS]	NQAQLELEAK	1143.600432	2	1.9286
gi|56464886|gb|EAL43273.1| actin-binding protein, putative [Entamoeba histolytica HM-1:IMSS]	SKSENLFSLQLK	1393.76859	3	1.784
gi|56465058|gb|EAL43404.1| 60S ribosomal protein L7, putative [Entamoeba histolytica HM-1:IMSS]	DIEAAKKAVYLAKK	1547.915558	3	1.6815
gi|56465061|gb|EAL43406.1| hypothetical protein 391.t00001 [Entamoeba histolytica HM-1:IMSS]	EVYKRVVSSEEKTK	1681.911951	3	1.8468
gi|56465087|gb|EAL43426.1| hypothetical protein 388.t00006 [Entamoeba histolytica HM-1:IMSS]	LFNNEIKEAK	1205.652467	2	1.6704
gi|56465137|gb|EAL43462.1| hypothetical protein 380.t00004 [Entamoeba histolytica HM-1:IMSS]	KVQTNYNEKKK	1379.764143	2	2.1935
gi|56465139|gb|EAL43464.1| hypothetical protein 380.t00006 [Entamoeba histolytica HM-1:IMSS]	DDDGNAVTFIK	1194.563715	2	3.349
gi|56465218|gb|EAL43526.1| cyclin, putative [Entamoeba histolytica HM-1:IMSS]	KRISPEFQNFCR	1581.774005	3	1.5179
gi|56465315|gb|EAL43600.1| RNA pseudouridylate synthase, putative [Entamoeba histolytica HM-1:IMSS]	KKEQEQLIK	1143.673202	2	2.0414
gi|56465397|gb|EAL43657.1| ARP2/3 complex 41 kDa subunit, putative [Entamoeba histolytica HM-1:IMSS]	VTGIDWAAQSNR	1317.654605	2	2.9176
gi|56465421|gb|EAL43679.1| 60S ribosomal protein L26, putative [Entamoeba histolytica HM-1:IMSS]	ALYTAPASQR	1077.568747	2	1.5537
	ANGQTVPVAIRPSNVIITK	1978.144402	3	2.174
gi|56465529|gb|EAL43773.1| enolase, putative [Entamoeba histolytica HM-1:IMSS]	IEEELGNIPYAGK	1432.731846	2	3.4467
gi|56465543|gb|EAL43786.1| 60S ribosomal protein L14, putative [Entamoeba histolytica HM-1:IMSS]	VLVDGPQAITGVPR	1421.811098	2	2.756
gi|56465573|gb|EAL43810.1| Rab family GTPase [Entamoeba histolytica HM-1:IMSS] gi|13537439|dbj|BAB40674.1| small GT	ATIGADFLTK	1036.567342	2	1.6413
gi|56465624|gb|EAL43848.1| hypothetical protein 328.t00002 [Entamoeba histolytica HM-1:IMSS]	AISAAVENGVAR	1157.627327	2	1.8684
	HVGLEDEGNFVDER	1615.734701	2	4.3924
	HVGLEDEGNFVDER	1615.734701	3	3.7955
	IAAQSNVAK	901.510169	2	2.3395
	INTITAQTK	989.562589	2	2.3227
	NNVETVAK	874.462875	2	1.6811
	SCVIQNR	876.414159	2	1.775
	TAIEQIVDK	1016.562255	2	2.5016
	TAIEQIVDK	1016.562255	1	2.0056
	VAAQPSVEAADK	1185.611007	2	2.9313
	VAQNIVK	771.472316	2	2.5616
	VAQNIVK	771.472316	1	1.7047
	YATLAAK	737.419218	1	1.7473
	YATVAEDR	924.44214	2	2.4403
	YATVAEDR	924.44214	2	2.0688
gi|56465657|gb|EAL43877.1| proteasome regulatory subunit, putative [Entamoeba histolytica HM-1:IMSS]	VIVHPLVLLSISDHQTR	1927.112381	3	1.5216
gi|56465661|gb|EAL43880.1| 40S ribosomal protein S24, putative [Entamoeba histolytica HM-1:IMSS]	DVQTIVLNGFVTK	1433.799862	2	2.4115
	NLIVNPLLCR	1211.671426	2	2.7022
	NLIVNPLLCR	1211.671426	2	2.1951
gi|56465739|gb|EAL43941.1| heat shock protein 70, putative [Entamoeba histolytica HM-1:IMSS]	DAGTIAGMNVMR	1235.58711	3	1.9547
	TTPSYVAFTDTER	1487.70128	2	2.5305
gi|56465790|gb|EAL43981.1| 60S ribosomal protein L9, putative [Entamoeba histolytica HM-1:IMSS]	LGKDEICLEGNDVEK	1718.805088	3	2.2289
	SIQVQNFLGEK	1262.673943	2	3.477
	VSQSAANIHAANLVR	1550.839788	3	2.9259
gi|56465886|gb|EAL44057.1| calreticulin, putative [Entamoeba histolytica HM-1:IMSS]	FEQGIDCGGGYIK	1443.635841	2	2.6344
	GLQTSEDNK	991.4690964	2	1.5691

	LDEEFSNK	981.452381	2	1.8264
gi|56465889|gb|EAL44060.1| 40S ribosomal protein S11, putative [Entamoeba histolytica HM-1:IMSS]	QLKEGDHVVIGQCR	1638.816594	3	1.8758
gi|56465908|gb|EAL44075.1| reverse transcriptase, putative [Entamoeba histolytica HM-1:IMSS]	KLEEKIK	887.556046	2	2.0996
gi|56465917|gb|EAL44081.1| kinesin, putative [Entamoeba histolytica HM-1:IMSS]	M*QADVKAYKELIR	1580.84759	2	1.5398
gi|56465934|gb|EAL44097.1| glycerophosphoryl diester phosphodiesterase, putative [Entamoeba histolytica HM-1:IMSS]	IQQLDAGYR	1063.553089	2	2.0498
gi|56465954|gb|EAL44112.1| hypothetical protein 296.t00002 [Entamoeba histolytica HM-1:IMSS]	RGEGILDRFDETFR	1710.855818	3	1.654
gi|56465985|gb|EAL44135.1| exonuclease, putative [Entamoeba histolytica HM-1:IMSS]	NGTGKSKVM*KDK	1308.695127	3	1.53
gi|56466019|gb|EAL44164.1| Truncated, galactose-specific adhesin 170kD subunit [Entamoeba histolytica HM-1:IMSS]	FVQLTDDPSLDSEHFR	1905.897759	2	4.2268
	FVQLTDDPSLDSEHFR	1905.897759	3	2.972
	IQEIVITEK	1072.624855	2	2.8679
	SGVELNNACLK	1204.577599	2	2.8847
	TCTVSEEFPTITPNQGR	1936.885471	2	3.1375
	WEIDTER	948.44214	2	1.8165
gi|56466046|gb|EAL44187.1| hypothetical protein 289.t00015 [Entamoeba histolytica HM-1:IMSS]	IEVLKTNIEMTRNR	1716.942518	3	1.7171
gi|56466065|gb|EAL44203.1| hypothetical protein 287.t00005 [Entamoeba histolytica HM-1:IMSS]	GISNALCGKQLPER	1542.784241	3	1.8478
gi|56466088|gb|EAL44223.1| Rab family GTPase [Entamoeba histolytica HM-1:IMSS]	VDISQTQSAQTQGGK	1547.766021	2	3.8937
gi|56466119|gb|EAL44251.1| hypothetical protein 283.t00010 [Entamoeba histolytica HM-1:IMSS]	KVFNILFDSNK	1324.725976	3	1.8319
gi|56466127|gb|EAL44258.1| Ras guanine nucleotide exchange factor, putative [Entamoeba histolytica HM-1:IMSS]	EAFDLSLK	922.488037	2	1.7249
gi|56466130|gb|EAL44261.1| hypothetical protein 282.t00010 [Entamoeba histolytica HM-1:IMSS]	CELEQNQNTR	1291.548079	3	1.5771
gi|56466201|gb|EAL44324.1| 40S ribosomal protein S26, putative [Entamoeba histolytica HM-1:IMSS]	DISDNSALTK	1063.526619	2	2.7843
	NIVEAAAVR	942.536708	2	2.5873
	NIVEAAAVR	942.536708	1	2.1602
	QQAVKPSTEAVK	1285.711055	2	2.3058
	VEIPADVVTAPK	1238.699083	2	1.7044
gi|56466210|gb|EAL44332.1| acid phosphatase, putative [Entamoeba histolytica HM-1:IMSS]	WFVEELNLR	1205.631337	2	1.9542
gi|56466264|gb|EAL44379.1| malate dehydrogenase, putative [Entamoeba histolytica HM-1:IMSS]	DTLIAADLR	987.546939	2	2.4571
gi|56466311|gb|EAL44418.1| hypothetical protein 269.t00007 [Entamoeba histolytica HM-1:IMSS]	EINGIDINGSM*EK	1435.674453	3	1.8067
gi|56466379|gb|EAL44475.1| hypothetical protein 265.t00002 [Entamoeba histolytica HM-1:IMSS]	M*NQQDVIALKFHK	1587.832274	2	2.3348
gi|56466440|gb|EAL44527.1| phospholipase D, putative [Entamoeba histolytica HM-1:IMSS]	KQDEKIVR	1015.589472	1	1.9127
gi|56466443|gb|EAL44530.1| oxidoreductase, short chain dehydrogenase/reductase family [Entamoeba histolytica HM-1:IMSS	LEEITGIKYPLN	1389.762414	3	1.6781
gi|56466445|gb|EAL44532.1| 60S ribosomal protein L27, putative [Entamoeba histolytica HM-1:IMSS]	AISSQFAEEYR	1300.616831	2	3.044
	MSAKVQQM*RTSVK	1509.788714	2	1.7432
gi|56466458|gb|EAL44544.1| hypothetical protein 259.t00005 [Entamoeba histolytica HM-1:IMSS]	TWADTTLENELK	1420.695456	3	1.548
gi|56466506|gb|EAL44584.1| 90 kDa heat shock protein, putative [Entamoeba histolytica HM-1:IMSS]	IGAITDVPNR	1055.584389	2	2.5317
	NFDVEVSR	965.468699	2	1.6222
	SLNIDPSSFK	1107.568099	2	1.7185
gi|56466556|gb|EAL44624.1| 60S acidic ribosomal protein P0, putative [Entamoeba histolytica HM-1:IMSS]	VGVSQAVLLQK	1141.693949	2	2.9112
gi|56466617|gb|EAL44678.1| acyl-CoA synthetase, putative [Entamoeba histolytica HM-1:IMSS]	EVSEILK	817.466573	2	1.5429
gi|56466622|gb|EAL44683.1| hypothetical protein 249.t00015 [Entamoeba histolytica HM-1:IMSS]	LIVEGEEDGEYWK	1566.732241	2	3.5623
gi|56466629|gb|EAL44689.1| 60S ribosomal protein L7a, putative [Entamoeba histolytica HM-1:IMSS]	AAFESLVQNVK	1205.652477	2	3.0794
	TNATQLFK	922.49926	2	1.8513
gi|56466636|gb|EAL44696.1| leucine rich repeat protein [Entamoeba histolytica HM-1:IMSS]	EIGKKQQKEIEMEK	1717.915303	2	1.5801
gi|56466696|gb|EAL44743.1| conserved hypothetical protein [Entamoeba histolytica HM-1:IMSS]	IPILNDELELM*K	1443.777445	2	1.7891
gi|56466709|gb|EAL44752.1| hypothetical protein 243.t00004 [Entamoeba histolytica HM-1:IMSS]	LYFTKIK	912.555317	2	1.7082
gi|56466731|gb|EAL44771.1| fatty acid elongase, putative [Entamoeba histolytica HM-1:IMSS]	VWQVAFGSGLK	1191.652087	2	1.8149
gi|56466782|gb|EAL44813.1| AAA family ATPase, putative [Entamoeba histolytica HM-1:IMSS]	TTRFIVDTGYSFK	1534.790036	2	1.5287
gi|56466843|gb|EAL44861.1| conserved hypothetical protein [Entamoeba histolytica HM-1:IMSS]	VYEQAVEKGIQVK	1490.821326	2	1.6586
gi|56466876|gb|EAL44891.1| hypothetical protein 231.t00008 [Entamoeba histolytica HM-1:IMSS]	VLNNSNSGM*SAFLNTK	1712.828345	3	1.8098
gi|56466888|gb|EAL44902.1| actin-like protein 3, putative [Entamoeba histolytica HM-1:IMSS]	HQAIDPISK	1008.547283	2	2.0317
gi|56466927|gb|EAL44933.1| Sec1 family protein [Entamoeba histolytica HM-1:IMSS]	YGVTLM*LNIKQTR	1552.852677	3	1.8476
gi|56466973|gb|EAL44971.1| Grainin 1 [Entamoeba histolytica HM-1:IMSS]	ELQDFFVK	1025.530226	2	2.5388

	ELQDFFVK	1025.530226	1	2.0246
	QAQGDFNR	935.4329744	2	1.7902
	SGSLELDELKK	1218.657635	2	3.0191
	TIYQHGFK	993.5152464	2	2.312
gi|56466980|gb|EAL44978.1| 40S ribosomal protein S26, putative [Entamoeba histolytica HM-1:IMSS]	VEVPADVVTAPK	1224.683433	2	1.8038
gi|56466988|gb|EAL44985.1| grainin 2 [Entamoeba histolytica HM-1:IMSS]	REEIDNAEFDK	1365.628105	3	3.4662
	SGSLDVNELIK	1174.63142	2	2.3139
	SGTLEIGELKK	1174.667798	2	2.9611
gi|56466991|gb|EAL44987.1| fructose-1,6-bisphosphate aldolase, putative [Entamoeba histolytica HM-1:IMSS]	LESAIGIPEDQIR	1440.769301	2	3.1592
gi|56466992|gb|EAL44988.1| integral membrane protein, putative [Entamoeba histolytica HM-1:IMSS]	EESVTLLTCEGESK	1581.709808	2	3.4187
gi|56467001|gb|EAL44997.1| hypothetical protein 223.t00011 [Entamoeba histolytica HM-1:IMSS]	AAEFEQVTAGYVPK	1509.758392	2	3.9483
	AAEFEQVTAGYVPK	1509.758392	2	1.7342
	AVEQALAEK	958.52039	2	2.8167
	IEKDDAPFPSALDLIE	1772.895289	2	3.1667
	KVEELEGEKNNEK	1545.7755	3	2.5246
	NAAAGSEELLK	1102.573895	2	2.8806
	YENNIPSLANEIK	1504.764214	2	2.5941
gi|56467079|gb|EAL45065.1| hypothetical protein 218.t00002 [Entamoeba histolytica HM-1:IMSS]	EGEYEEILK	1109.536103	2	1.9169
gi|56467082|gb|EAL45068.1| heat shock protein 70, putative [Entamoeba histolytica HM-1:IMSS]	NTLSEQFADK	1152.553158	2	2.0075
gi|56467085|gb|EAL45071.1| hypothetical protein 218.t00011 [Entamoeba histolytica HM-1:IMSS]	LGGYINER	921.4788638	2	1.502
gi|56467136|gb|EAL45116.1| protein kinase, putative [Entamoeba histolytica HM-1:IMSS]	IMGM*KIPYTVWFSKHSK	2069.072208	3	2.0764
gi|56467216|gb|EAL45189.1| EhABP-related protein [Entamoeba histolytica HM-1:IMSS]	LTSRKIK	845.556724	2	1.6513
gi|56467236|gb|EAL45208.1| RNA methyltransferase, putative [Entamoeba histolytica HM-1:IMSS]	AAKLNAELNK	1071.615687	3	1.8167
gi|56467243|gb|EAL45214.1| lysyl-tRNA synthetase, putative [Entamoeba histolytica HM-1:IMSS]	SQVSDDIVSVAGR	1332.675421	2	3.0596
gi|56467244|gb|EAL45215.1| hypothetical protein 209.t00003 [Entamoeba histolytica HM-1:IMSS]	FSTEFEYGGSK	1251.552839	2	2.5432
	IMLQGNEKSK	1147.613984	2	1.6499
	SYDKEPYVILPNK	1565.821	2	3.9006
	SYDKEPYVILPNK	1565.821	3	2.8745
gi|56467276|gb|EAL45245.1| T-complex protein 1 alpha subunit, putative [Entamoeba histolytica HM-1:IMSS]	GANATMLDEM*SR	1311.567875	3	2.0686
gi|56467297|gb|EAL45264.1| protein disulfide isomerase, putative [Entamoeba histolytica HM-1:IMSS]	FGISGFPTLK	1066.593175	2	2.698
	IAELDDLAK	987.535706	2	2.5256
	KAEAIEGGAYYVK	1398.726366	3	3.329
	KGTTEPIEYEGGR	1436.701611	3	3.1852
	KGTTEPIEYEGGR	1436.701611	2	2.4065
	KPIAYEGGR	990.5367128	2	2.0442
	TVEDLSHFIQEK	1445.7271	2	3.8684
	TVEDLSHFIQEK	1445.7271	3	2.1945
gi|56467383|gb|EAL45345.1| hypothetical protein 202.t00014 [Entamoeba histolytica HM-1:IMSS]	ILLTSSFDEMK	1283.655189	2	1.5383
gi|56467435|gb|EAL45394.1| hypothetical protein 199.t00003 [Entamoeba histolytica HM-1:IMSS]	AKDLLPRMQVEWR	1641.889359	3	2.0984
gi|56467455|gb|EAL45413.1| transcription initiation factor IIIB chain BRF, putative [Entamoeba histolytica HM-1:IMSS]	LAKGEVDIK	972.5724274	2	2.3177
gi|56467463|gb|EAL45421.1| DNA replication licensing factor, putative [Entamoeba histolytica HM-1:IMSS]	TGTLLM*QPRASKFVK	1692.94765	3	1.6729
gi|56467488|gb|EAL45445.1| Rho family GTPase [Entamoeba histolytica HM-1:IMSS]	KITPITTEQGEAK	1415.774042	3	2.8056
	LVFDEAVR	948.51491	2	2.0056
	YIECSALTQK	1212.571449	2	2.9881
gi|56467508|gb|EAL45462.1| phenylalanyl-tRNA synthetase beta subunit, putative [Entamoeba histolytica HM-1:IMSS]	PEEIKFEPLK	1229.677619	2	1.5605
gi|56467522|gb|EAL45475.1| 60S ribosomal protein L24, putative [Entamoeba histolytica HM-1:IMSS]	GQQNELVEK	1044.53202	2	2.2526
	GYSGISAEEIAK	1224.610688	2	3.5132
	QTQSSEVLAK	1090.573903	2	2.3729
gi|56467551|gb|EAL45502.1| 60S ribosomal protein L27a, putative [Entamoeba histolytica HM-1:IMSS]	VGEEVPVIDCVK	1343.66607	2	2.7359
gi|56467570|gb|EAL45517.1| conserved hypothetical protein [Entamoeba histolytica HM-1:IMSS]	M*EELISK	865.434656	1	2.1238
gi|56467606|gb|EAL45551.1| cysteine proteinase, putative [Entamoeba histolytica HM-1:IMSS]	AAAEGPVAAAIDASGVK	1497.790768	2	2.2158

gi|56467610|gb|EAL45555.1| hypothetical protein 191.t00022 [Entamoeba histolytica HM-1:IMSS]	EKGTKGSNITLKMK	1534.862157	2	1.9526
gi|56467613|gb|EAL45557.1| hypothetical protein 190.t00019 [Entamoeba histolytica HM-1:IMSS]	QTSQNTNQPVSK	1331.655008	2	1.604
gi|56467688|gb|EAL45621.1| hypothetical protein 187.t00002 [Entamoeba histolytica HM-1:IMSS]	TNKVNDDINDSK	1362.649579	2	2.2898
gi|56467714|gb|EAL45646.1| protein disulfide isomerase, putative [Entamoeba histolytica HM-1:IMSS]	GNENIEFEGPR	1261.580765	2	2.261
gi|56467717|gb|EAL45649.1| Rab family GTPase [Entamoeba histolytica HM-1:IMSS] gi|56790064|dbj|BAD82828.1| EhRabC	LQIWDTAGQER	1316.659346	2	3.2871
gi|56467754|gb|EAL45684.1| Ras family GTPase [Entamoeba histolytica HM-1:IMSS]	IVVLGSGAVGK	999.6197262	2	2.2573
	YDPTIEDSYR	1258.558638	2	2.3598
gi|56467791|gb|EAL45718.1| Rab family GTPase [Entamoeba histolytica HM-1:IMSS] gi|56790168|dbj|BAD82880.1| EhRabX	KKVAFIGDATVGK	1333.78382	3	1.7034
gi|56467864|gb|EAL45777.1| Ras family GTPase [Entamoeba histolytica HM-1:IMSS]	DTENVPIIVVGNK	1397.763477	2	2.98
	VRDTENVPIIVVGNK	1652.933002	3	2.5751
	YGAEFLEVSAK	1213.609946	2	3.9251
gi|56468005|gb|EAL45900.1| 60S ribosomal protein L38, putative [Entamoeba histolytica HM-1:IMSS]	DIHVVEIAK	1023.583324	3	2.7178
	DIHVVEIAK	1023.583324	2	2.6261
	M*PKQVQEFK	1150.593606	3	2.0971
	QVQEFK	778.409382	2	1.709
	QVQEFK	778.409382	2	1.5997
gi|56468007|gb|EAL45901.1| Plasma membrane calcium-transporting ATPase, putative [Entamoeba histolytica HM-1:IMSS]	RM*NTLVWIDKPNTIR	1873.012364	3	1.6094
gi|56468010|gb|EAL45903.1| hypothetical protein 172.t00003 [Entamoeba histolytica HM-1:IMSS]	KLISKGIR	914.6145754	2	1.5544
gi|56468069|gb|EAL45958.1| hypothetical protein 170.t00006 [Entamoeba histolytica HM-1:IMSS]	IIRIYPHPELNK	1492.863462	2	1.7177
gi|56468106|gb|EAL45992.1| galactose-inhibitable lectin 35 kda subunit precursor, putative [Entamoeba histolytica HM-1:IMSS	VIFASDYNYK	1219.599379	2	2.3826
gi|56468130|gb|EAL46014.1| conserved hypothetical protein [Entamoeba histolytica HM-1:IMSS]	ILLGIM*NNSESYQKIFSK	2101.100938	3	1.789
gi|56468165|gb|EAL46046.1| cation diffusion facilitator, putative [Entamoeba histolytica HM-1:IMSS]	KRNIYK	821.499199	2	1.623
gi|56468182|gb|EAL46059.1| conserved hypothetical protein [Entamoeba histolytica HM-1:IMSS]	M*LTMNLM*TR	1142.538844	2	1.7754
gi|56468273|gb|EAL46139.1| erythrocyte binding protein, putative [Entamoeba histolytica HM-1:IMSS]	TQEVNEQIEK	1217.600827	2	2.6489
gi|56468289|gb|EAL46152.1| methionine aminopeptidase, putative [Entamoeba histolytica HM-1:IMSS]	TYQLQPVR	1004.552358	2	1.7919
gi|56468302|gb|EAL46165.1| arginyl-tRNA synthetase [Entamoeba histolytica HM-1:IMSS]	LAEQIK	701.419218	2	1.7587
gi|56468368|gb|EAL46222.1| profilin, putative [Entamoeba histolytica HM-1:IMSS]	VDADEGTAMGK	1093.483024	2	2.1605
gi|56468422|gb|EAL46268.1| DNA mismatch repair protein mutS, putative [Entamoeba histolytica HM-1:IMSS]	LAHLPSSLISR	1193.700115	2	1.6399
gi|56468427|gb|EAL46273.1| histone H3, putative [Entamoeba histolytica HM-1:IMSS]	AHPGAVALTEIK	1206.684103	3	2.8279
	EIAQVSK	774.435607	2	1.8975
gi|56468432|gb|EAL46278.1| protein kinase, putative [Entamoeba histolytica HM-1:IMSS]	YVKEKQLGKDIK	1448.847146	2	1.6054
gi|56468458|gb|EAL46302.1| actophorin, putative [Entamoeba histolytica HM-1:IMSS]	YAVYDLEYDTPEGLR	1803.84358	2	2.9401
gi|56468537|gb|EAL46376.1| conserved hypothetical protein [Entamoeba histolytica HM-1:IMSS]	FTFQIAFYER	1321.657552	3	1.9442
gi|56468567|gb|EAL46401.1| hypothetical protein 147.t00011 [Entamoeba histolytica HM-1:IMSS]	PENIILPLK	1036.64011	2	1.7676
gi|56468580|gb|EAL46413.1| Rho family GTPase [Entamoeba histolytica HM-1:IMSS]	INLGLWDTAGQEDYDR	1865.866444	2	2.9078
	NIVPIQPPQGDELAK	1618.879904	2	2.3526
gi|56468630|gb|EAL46461.1| hypothetical protein 144.t00010 [Entamoeba histolytica HM-1:IMSS]	AIMNIISLEK	1131.644219	2	2.8974
	AIMNIISLEK	1131.644219	2	2.4924
	INKEEIIR	1014.594223	2	2.5872
	INKEEIIR	1014.594223	3	2.5696
gi|56468634|gb|EAL46465.1| conserved hypothetical protein [Entamoeba histolytica HM-1:IMSS]	NVLKTDISM*K	1164.630396	2	1.709
gi|56468643|gb|EAL46474.1| hypothetical protein 144.t00023 [Entamoeba histolytica HM-1:IMSS]	LENKTYIER	1165.621167	2	2.0909
gi|56468654|gb|EAL46483.1| translation elongation factor EF-1 alpha, putative [Entamoeba histolytica HM-1:IMSS]	ISGIGTVPVGR	1055.620789	2	2.0808
gi|56468666|gb|EAL46493.1| hypothetical protein 142.t00028 [Entamoeba histolytica HM-1:IMSS]	KGGTISGISR	975.5581992	2	1.5062
gi|56468685|gb|EAL46511.1| hypothetical protein 142.t00032 [Entamoeba histolytica HM-1:IMSS]	FLEAAKPILNY	1278.709253	3	2.3117
gi|56468760|gb|EAL46577.1| hypothetical protein 138.t00014 [Entamoeba histolytica HM-1:IMSS]	SVVDIEIKTR	1159.668127	2	1.7959
gi|56468761|gb|EAL46578.1| Rho guanine nucleotide exchange factor, putative [Entamoeba histolytica HM-1:IMSS]	IPPQVPNK	892.52508	1	1.5792
gi|56468784|gb|EAL46597.1| hypothetical protein 137.t00004 [Entamoeba histolytica HM-1:IMSS]	AEITSSKGIISLEDLFK	1851.011009	2	1.5096
gi|56468896|gb|EAL46702.1| hypothetical protein 133.t00008 [Entamoeba histolytica HM-1:IMSS]	IVGVVEK	743.4661704	1	2.3391
gi|56468901|gb|EAL46705.1| hypothetical membrane-spanning protein [Entamoeba histolytica HM-1:IMSS]	TSKDPQAEEAPAEDPEVDK	2055.935319	3	3.821
gi|56468917|gb|EAL46720.1| hypothetical protein 132.t00009 [Entamoeba histolytica HM-1:IMSS]	YLKM*SIKLATR	1339.777728	2	1.6093

gi|56468924|gb|EAL46725.1| DEAD/DEAH box helicase, putative [Entamoeba histolytica HM-1:IMSS]	DLEESFALIITNATR	1692.880307	2	1.9991
gi|56469014|gb|EAL46809.1| hypothetical protein 128.t00003 [Entamoeba histolytica HM-1:IMSS]	TYNIFEAAPDATNK	1554.743469	2	3.8205
gi|56469015|gb|EAL46810.1| hypothetical protein 128.t00004 [Entamoeba histolytica HM-1:IMSS]	IEQYTYLVK	1156.624855	2	1.745
gi|56469021|gb|EAL46816.1| hypothetical protein 128.t00010 [Entamoeba histolytica HM-1:IMSS]	GYQVTEGTTK	1083.531689	2	2.241
	LDESSSLSK	965.478626	2	1.7499
	NGDAVAEVLEK	1144.58445	2	2.6476
	VENVAGVASK	973.5313014	2	3.2001
	VGELATSPITTGIEK	1515.826485	2	3.4213
gi|56469045|gb|EAL46838.1| hypothetical protein 127.t00015 [Entamoeba histolytica HM-1:IMSS]	RHNEENIIQM*GRNK	1754.872579	3	1.605
gi|56469086|gb|EAL46875.1| hypothetical protein 125.t00016 [Entamoeba histolytica HM-1:IMSS]	LTSVEGYDKKRTLM*R	1812.964758	3	1.9832
gi|56469109|gb|EAL46895.1| conserved hypothetical protein [Entamoeba histolytica HM-1:IMSS]	PNEFAVLFPRKTAK	1617.911141	3	1.7394
gi|56469142|gb|EAL46923.1| Rab family GTPase [Entamoeba histolytica HM-1:IMSS] gi|56790050|dbj|BAD82821.1| EhRab8I	LILIGESGVGK	1085.656506	2	3.0354
gi|56469186|gb|EAL46961.1| DNA-directed RNA polymerase subunit, putative [Entamoeba histolytica HM-1:IMSS]	FRLIM*DGTEGVNDTELGK	2010.981193	2	1.6943
gi|56469194|gb|EAL46969.1| hypothetical protein 121.t00010 [Entamoeba histolytica HM-1:IMSS]	SSSGAKTTEK	995.5004164	2	1.8155
gi|56469245|gb|EAL47008.1| Gal/GalNAc lectin subunit Igl2 [Entamoeba histolytica HM-1:IMSS]	ASDNTYYCPLK	1331.572178	2	3.3209
	CNAEYLEAK	1097.471725	2	3.2113
	GYDQYEQYLNAFK	1638.743471	2	4.2079
	LFSPTEENK	1064.52588	2	2.0936
	LINNQEPR	983.526872	2	2.5282
	VNEFCDSCNSK	1359.487472	2	3.2026
gi|56469250|gb|EAL47013.1| hypothetical protein 119.t00011 [Entamoeba histolytica HM-1:IMSS]	RFGNILIDSDK	1277.684842	2	1.7355
gi|56469308|gb|EAL47068.1| Ras GTPase activating protein, putative [Entamoeba histolytica HM-1:IMSS]	IVDLYIKLGK	1161.724176	2	2.2564
gi|56469323|gb|EAL47082.1| histone H2A, putative [Entamoeba histolytica HM-1:IMSS]	DVTISYGGVFPNVPTAVNSK	2065.060078	2	4.3971
	ENEVTPIVEK	1157.604849	2	2.1027
	HIQLAVR	836.510098	2	2.2964
	IHAALK	652.414072	2	1.6876
	IHAALK	652.414072	2	1.651
	KSGLQFPVAR	1102.636768	2	2.4449
	SDLELNDLLK	1159.620509	1	2.3165
	SGLQFPVAR	974.5418054	2	2.5364
gi|56469399|gb|EAL47152.1| 60S ribosomal protein L10, putative [Entamoeba histolytica HM-1:IMSS]	LFDIGNR	834.4468324	2	2.0748
	VGQVLISGR	928.5574578	2	3.0265
gi|56469505|gb|EAL47248.1| leucine rich repeat protein [Entamoeba histolytica HM-1:IMSS]	GVEALQMVK	974.5339324	2	1.8575
gi|56469523|gb|EAL47265.1| hypothetical protein 110.t00022 [Entamoeba histolytica HM-1:IMSS]	QEEGKEYIK	1123.562986	2	1.5234
gi|56469542|gb|EAL47282.1| conserved hypothetical protein [Entamoeba histolytica HM-1:IMSS]	KFIKEIGAFK	1180.70886	2	2.1679
gi|56469572|gb|EAL47310.1| Rho family GTPase [Entamoeba histolytica HM-1:IMSS]	GEELKNRIKAVK	1384.827079	2	1.9185
gi|56469632|gb|EAL47362.1| hypothetical protein 106.t00012 [Entamoeba histolytica HM-1:IMSS]	KIEM*KEENTKELK	1635.863301	3	1.6104
gi|56469640|gb|EAL47370.1| hypothetical protein 106.t00028 [Entamoeba histolytica HM-1:IMSS]	SLLESNVIFLK	1262.735488	2	1.5554
gi|56469645|gb|EAL47374.1| 60S ribosomal protein L4, putative [Entamoeba histolytica HM-1:IMSS]	EALNQSVAK	959.515649	2	1.8336
	GDVVHFVYR	1091.563259	2	2.0034
	PFVTIYDGITGEAEK	1639.82139	2	1.5677
gi|56469662|gb|EAL47390.1| Rab family GTPase [Entamoeba histolytica HM-1:IMSS] gi|13537447|dbj|BAB40678.1| small GT	EVQTSEGEELAK	1319.632534	2	3.0433
	NEFDPDKR	1020.474503	2	2.3222
	TSFESVEK	926.446577	2	1.7103
	VVQMVIGNK	987.5655664	2	2.1731
gi|56469773|gb|EAL47489.1| DNA repair protein Rad50, putative [Entamoeba histolytica HM-1:IMSS]	KLETQSIDVQQK	1416.769299	2	1.9564
gi|56469786|gb|EAL47501.1| Rab family GTPase [Entamoeba histolytica HM-1:IMSS]	FVSDQFSEVTK	1286.626333	2	2.1543
gi|56469825|gb|EAL47534.1| hypothetical protein 100.t00015 [Entamoeba histolytica HM-1:IMSS]	AKGMADKFKK	1123.629229	2	1.8433
gi|56469826|gb|EAL47535.1| hypothetical protein 100.t00016 [Entamoeba histolytica HM-1:IMSS]	KELGVVKK	900.5876824	2	1.755
gi|56469894|gb|EAL47598.1| pyruvate:ferredoxin oxidoreductase [Entamoeba histolytica HM-1:IMSS]	APAEFVQIDGK	1174.61027	2	3.2055
gi|56469901|gb|EAL47604.1| conserved hypothetical protein [Entamoeba histolytica HM-1:IMSS]	SLKDHLIVAK	1123.683382	2	1.8384

gi|56469970|gb|EAL47665.1| Rab family GTPase [Entamoeba histolytica HM-1:IMSS] gi|14423577|gb|AAK62471.1| small GT	NIEANADQNVNK	1329.639338	2	4.1756
	VDDCFISLAR	1195.556133	2	2.5441
gi|56469979|gb|EAL47673.1| Sand family protein [Entamoeba histolytica HM-1:IMSS]	LCSIVPYTTILSLFQK	1883.013243	3	2.3837
gi|56470016|gb|EAL47708.1| hypothetical protein 94.t00023 [Entamoeba histolytica HM-1:IMSS]	ELNTRCINTQKFK	1651.83699	2	1.6138
gi|56470060|gb|EAL47746.1| heat shock protein 90, putative [Entamoeba histolytica HM-1:IMSS]	EM*LQQNK	906.436043	2	2.3477
gi|56470088|gb|EAL47773.1| 60S ribosomal protein L23, putative [Entamoeba histolytica HM-1:IMSS] gi|56468746|gb|EAL46	AGCGDMVVATCK	1268.500268	2	3.7954
	MHTAVIIR	940.539683	2	1.5787
	TLTIISVK	874.560807	2	2.2471
	VTCGLNNASTVNCADNTGAK	2066.880071	2	4.5973
gi|56470123|gb|EAL47806.1| nucleotide binding protein 2, putative [Entamoeba histolytica HM-1:IMSS]	FVGAIPIEPK	1070.624462	2	1.8597
gi|56470195|gb|EAL47873.1| hypothetical protein 88.t00009 [Entamoeba histolytica HM-1:IMSS]	KISNENTLTKKK	1403.821668	2	1.6223
gi|56470246|gb|EAL47922.1| Rho family GTPase [Entamoeba histolytica HM-1:IMSS]	VIVIGDTNVGK	1114.646658	2	2.0914
gi|56470330|gb|EAL47999.1| Beige protein, putative [Entamoeba histolytica HM-1:IMSS]	VSVLLKDAAEQR	1328.753254	2	1.5604
gi|56470331|gb|EAL48000.1| histone H3, putative [Entamoeba histolytica HM-1:IMSS]	DMQLAR	733.366136	2	1.5562
	STELLLR	831.493456	2	1.8533
gi|56470333|gb|EAL48002.1| protein with RhoGEF and ArfGAP domains [Entamoeba histolytica HM-1:IMSS]	DTENVMEELK	1207.551099	2	1.6936
gi|56470384|gb|EAL48050.1| conserved hypothetical protein [Entamoeba histolytica HM-1:IMSS]	NGSNAGYSIEQILQSK	1708.850095	2	1.5298
gi|56470398|gb|EAL48062.1| 40S ribosomal protein S3a, putative [Entamoeba histolytica HM-1:IMSS]	LLDQHQDTSAPVEAAPVAEEK	2248.1092	3	2.9178
gi|56470445|gb|EAL48102.1| DNA-directed RNA polymerase subunit, putative [Entamoeba histolytica HM-1:IMSS]	ALGTGNWPQPGGK	1282.653874	2	1.5688
gi|56470472|gb|EAL48126.1| hypothetical protein 80.t00019 [Entamoeba histolytica HM-1:IMSS]	IVEISDIPK	1013.587751	2	2.5102
	LNETIPEPK	1040.562255	2	2.0752
gi|56470526|gb|EAL48173.1| 40S ribosomal protein S14, putative [Entamoeba histolytica HM-1:IMSS]	IEDVTPIPTDCTK	1488.703577	2	3.3526
	SNVPEVIPGSTK	1227.657969	2	2.6802
	TPGPGAQAALR	1038.569076	2	2.6726
gi|56470539|gb|EAL48184.1| sulfate adenylyltransferase, putative [Entamoeba histolytica HM-1:IMSS]	DHAGVGDYYGPFDAQK	1739.766003	3	3.223
	VTTLSEGEFPIK	1320.704575	2	2.9716
gi|56470574|gb|EAL48217.1| 60S ribosomal protein L6, putative [Entamoeba histolytica HM-1:IMSS]	TVEEDFAKK	1066.54152	2	2.3437
gi|56470591|gb|EAL48233.1| U2 snRNP auxiliary factor large subunit, putative [Entamoeba histolytica HM-1:IMSS]	IRYKSSR	909.526497	2	1.6124
gi|56470595|gb|EAL48237.1| hypothetical protein 77.t00024 [Entamoeba histolytica HM-1:IMSS]	EMKDIPDVKK	1202.644938	3	2.4388
gi|56470601|gb|EAL48243.1| hypothetical protein 77.t00030 [Entamoeba histolytica HM-1:IMSS]	ILLEEEEGEAPTPK	1554.789753	2	4.7917
gi|56470638|gb|EAL48277.1| hypothetical protein 76.t00027 [Entamoeba histolytica HM-1:IMSS]	NVKFDILKAEDNK	1533.827138	2	2.6271
gi|56470665|gb|EAL48302.1| hypothetical protein 75.t00024 [Entamoeba histolytica HM-1:IMSS]	FNISLSSETKK	1253.673627	2	1.7173
gi|56470751|gb|EAL48382.1| Rho guanine nucleotide exchange factor, putative [Entamoeba histolytica HM-1:IMSS]	IKETMAVPTPEAKR	1570.862142	3	1.794
gi|56470804|gb|EAL48425.1| conserved hypothetical protein [Entamoeba histolytica HM-1:IMSS]	VNKKENKFNDK	1363.732843	2	1.8303
gi|56470826|gb|EAL48444.1| protein kinase, putative [Entamoeba histolytica HM-1:IMSS]	SEYKLTKNNNR	1366.707367	2	2.0655
gi|56470844|gb|EAL48461.1| hypothetical protein 70.t00035 [Entamoeba histolytica HM-1:IMSS]	AINLIISILMLK	1341.853817	3	1.985
gi|56470894|gb|EAL48508.1| BspA-like leucine rich repeat protein, putative [Entamoeba histolytica HM-1:IMSS]	KDYINLIMTNSR	1467.762438	2	1.5075
	LKIPKGIER	1053.677894	2	1.5252
gi|56470969|gb|EAL48575.1| hypothetical protein 67.t00007 [Entamoeba histolytica HM-1:IMSS]	M*VITKFEVTGTALSILK	1867.062012	3	1.71
gi|56470972|gb|EAL48578.1| 40S ribosomal protein S15a, putative [Entamoeba histolytica HM-1:IMSS]	VNVLADALK	942.56186	2	2.6856
gi|56470977|gb|EAL48583.1| phosphatidylinositol 3-kinase, putative [Entamoeba histolytica HM-1:IMSS]	KNM*YQYM*FDK	1399.604283	2	1.7126
gi|56471006|gb|EAL48609.1| Sphingomyelin phosphodiesterase, putative [Entamoeba histolytica HM-1:IMSS]	ITGM*M*DVNPYFTR	1576.715627	3	1.6583
gi|56471058|gb|EAL48654.1| RNA-binding protein, putative [Entamoeba histolytica HM-1:IMSS]	FLKQFGKLESYR	1515.83184	2	1.8728
gi|56471065|gb|EAL48661.1| leucine rich repeat protein [Entamoeba histolytica HM-1:IMSS]	NLALLDLSFNDLIK	1588.894499	2	1.8245
gi|56471119|gb|EAL48713.1| Sar family GTPase [Entamoeba histolytica HM-1:IMSS]	IDIPGAVSEAQLR	1368.748171	2	2.3971
gi|56471175|gb|EAL48765.1| hypothetical protein 61.t00020 [Entamoeba histolytica HM-1:IMSS]	FGGYANEKIDKYEMKK	1920.95242	2	1.8267
gi|56471191|gb|EAL48781.1| hypothetical protein 61.t00036 [Entamoeba histolytica HM-1:IMSS]	KDDCKIFNIGK	1337.666738	3	1.6826
gi|56471228|gb|EAL48814.1| myosin heavy chain [Entamoeba histolytica HM-1:IMSS]	DKIEGLTLDNEDLKKK	1859.012042	2	2.078
	EAMDKELMK	1094.522045	2	1.8169
	VEDLEGQLEKAK	1358.716193	2	3.7165
gi|56471290|gb|EAL48870.1| BspA-like leucine rich repeat protein, putative [Entamoeba histolytica HM-1:IMSS]	ECFSGSRGLKEITMNNIK	2084.004886	2	2.4232

gi|56471306|gb|EAL48886.1| hypothetical protein 58.t00022 [Entamoeba histolytica HM-1:IMSS]	KAASKINK	859.535989	2	2.0955
gi|56471327|gb|EAL48906.1| hypothetical protein 57.t00027 [Entamoeba histolytica HM-1:IMSS]	NEVTQKPLNPIQLSK	1708.959225	3	2.1771
gi|56471346|gb|EAL48923.1| Rap/Ran GTPase activating protein, putative [Entamoeba histolytica HM-1:IMSS]	QDSNDKK	834.3952	2	1.6926
gi|56471364|gb|EAL48939.1| nuclear pore protein, putative [Entamoeba histolytica HM-1:IMSS]	AVQAVAVLPFRAGIVAK	1710.042491	2	1.711
gi|56471403|gb|EAL48974.1| 40S ribosomal protein S4, putative [Entamoeba histolytica HM-1:IMSS]	FCLVPLTAEQAK	1376.702787	2	3.0644
	GTETLVNLPLDK	1299.715464	2	3.149
	KPLLQQVNETIK	1410.831494	3	2.8084
	LGGTFAPK	790.4457718	2	2.1829
	LNLETGK	774.4355994	2	2.1235
	LNNVFVIGK	1003.593496	2	3.1199
gi|56471436|gb|EAL49006.1| villidin, putative [Entamoeba histolytica HM-1:IMSS]	GGIIIKKSIYPEK	1445.872645	3	1.77
gi|56471459|gb|EAL49028.1| hypothetical protein 54.t00040 [Entamoeba histolytica HM-1:IMSS]	QSTSSKVAEKQK	1320.711804	2	2.4503
gi|56471461|gb|EAL49030.1| hypothetical protein 54.t00042 [Entamoeba histolytica HM-1:IMSS]	LKSALEELEKKK	1415.84682	2	1.9817
gi|56471496|gb|EAL49060.1| Gal/GalNAc lectin subunit Igl1 [Entamoeba histolytica HM-1:IMSS]	ASDCVSITTK	1081.49796	2	2.3835
	GTSDNTITIGCVGQLR	1691.816667	2	3.395
	HIPTSIDK	910.49927	2	1.8369
	KVECVTCTEQR	1409.608235	3	2.5989
	TANGNICTQCPVGSHVGK	1899.837084	3	2.4518
	TGFIYATECSDGFSGR	1767.742844	2	4.0147
gi|56471506|gb|EAL49070.1| 60S ribosomal protein L5, putative [Entamoeba histolytica HM-1:IMSS]	FAGAGENEEALR	1263.596415	2	2.7524
gi|56471517|gb|EAL49081.1| hypothetical protein 53.t00028 [Entamoeba histolytica HM-1:IMSS]	IM*KKADMIR	1121.618045	2	1.8703
	RINGGAYSSVYLVRKK	1811.028656	3	1.5663
gi|56471536|gb|EAL49099.1| hypothetical protein 52.t00023 [Entamoeba histolytica HM-1:IMSS]	DALNENEFIK	1192.584448	2	1.7155
gi|56471635|gb|EAL49189.1| hypothetical protein 50.t00042 [Entamoeba histolytica HM-1:IMSS]	IIPNNSFNSLSSK	1420.743114	2	1.5571
gi|56471639|gb|EAL49192.1| 40S ribosomal protein S2, putative [Entamoeba histolytica HM-1:IMSS]	GTGIVAAPTPK	1011.583329	2	2.8365
gi|56471669|gb|EAL49221.1| NOL1/NOP2/sun family protein [Entamoeba histolytica HM-1:IMSS]	DLLKTLFNNSCDEIK	1809.883677	3	1.8302
gi|56471702|gb|EAL49249.1| hypothetical protein 48.t00014 [Entamoeba histolytica HM-1:IMSS]	FFNVQNDEEEVAEETNAQ	2112.899258	2	3.8381
	KVTITTSSAK	1035.604474	2	1.7431
	VTITTSSAK	907.509511	2	2.126
gi|56471704|gb|EAL49251.1| hypothetical protein 48.t00016 [Entamoeba histolytica HM-1:IMSS]	ALKLFPNAR	1029.620377	2	1.5839
gi|56471711|gb|EAL49258.1| hypothetical protein 48.t00023 [Entamoeba histolytica HM-1:IMSS]	IGIEDLSK	874.4880394	2	1.5909
gi|56471731|gb|EAL49276.1| conserved hypothetical protein [Entamoeba histolytica HM-1:IMSS]	AQATGYASDAYTAM*EVSK	1879.838961	3	1.6362
gi|56471779|gb|EAL49323.1| hypothetical protein 46.t00041 [Entamoeba histolytica HM-1:IMSS]	DANVLIER	929.505074	2	2.1134
gi|56471809|gb|EAL49351.1| 70 kDa heat shock protein, putative [Entamoeba histolytica HM-1:IMSS]	ALSVQQQDR	1044.543261	2	2.4308
	FDLDGIQPAPR	1228.632068	2	3.3379
	NQIAQNPENTIFEIK	1758.902095	2	3.346
	NQLENYAYQIK	1383.69031	2	3.5413
	NTHIPISK	909.515254	2	2.0823
gi|56471815|gb|EAL49357.1| hypothetical protein 45.t00011 [Entamoeba histolytica HM-1:IMSS]	DAIQGPFVVPSNADEIK	1799.917423	2	3.4985
	DAIQGPFVVPSNADEIK	1799.917423	2	2.8562
	ETPVVSSK	846.456747	2	2.0568
	FGQTVEK	808.4199494	2	2.2351
gi|56471818|gb|EAL49360.1| ARP2/3 complex 34 kda subunit, putative [Entamoeba histolytica HM-1:IMSS]	SAAGCPSCDVVTR	1379.561307	2	1.6053
gi|56471823|gb|EAL49365.1| midasin, putative [Entamoeba histolytica HM-1:IMSS]	SILLTVPSKWIK	1384.856271	2	1.6273
gi|56471829|gb|EAL49371.1| hypothetical protein 45.t00025 [Entamoeba histolytica HM-1:IMSS]	IEGVNEDKVR	1158.611333	2	1.8119
gi|56471880|gb|EAL49413.1| hypothetical protein 43.t00025 [Entamoeba histolytica HM-1:IMSS]	PMAETSVAIDIPRLVSK	1827.00447	2	2.1234
gi|56471887|gb|EAL49420.1| Sir2 family transcriptional regulator, putative [Entamoeba histolytica HM-1:IMSS]	GDLKEIVPKIANKIK	1666.026173	3	2.4249
gi|56471889|gb|EAL49422.1| villin, putative [Entamoeba histolytica HM-1:IMSS]	IRQEEM*EKEHQRYVK	2019.008737	3	1.5065
gi|56471906|gb|EAL49438.1| protein phosphatase, putative [Entamoeba histolytica HM-1:IMSS]	LTAKVVVPEFFL	1362.803153	2	1.7107
gi|56471994|gb|EAL49518.1| La ribonucleoprotein, putative [Entamoeba histolytica HM-1:IMSS]	HETTHNEDTTKVEDKK	1911.904282	3	1.8649
gi|56471996|gb|EAL49520.1| hypothetical protein 41.t00028 [Entamoeba histolytica HM-1:IMSS]	VLFPQLKISKEK	1429.877725	2	1.5139

gi|56472035|gb|EAL49554.1| steroid 5-alpha reductase, putative [Entamoeba histolytica HM-1:IMSS]	DLGAQLPFR	1016.55236	2	2.9949
	GNTYTVETELTK	1355.668909	2	2.8892
	TLQESGVTEDTPIEMK	1777.85244	2	4.5344
	TPGSQEWK	932.4472374	2	1.8011
	VVLEVDK	801.471648	2	1.7567
	YLHMFPDYPK	1310.623808	2	2.4318
gi|56472090|gb|EAL49603.1| zinc finger protein, putative [Entamoeba histolytica HM-1:IMSS]	QEIYVSEGKVR	1307.695407	3	1.5771
gi|56472162|gb|EAL49670.1| hypothetical protein 37.t00027 [Entamoeba histolytica HM-1:IMSS]	KKKDSSSLDELK	1377.75842	2	1.7197
gi|56472233|gb|EAL49733.1| hypothetical protein 36.t00014 [Entamoeba histolytica HM-1:IMSS]	FVVVENIDIWEFTDDR	1996.96509	2	1.6973
gi|56472251|gb|EAL49750.1| protein kinase, putative [Entamoeba histolytica HM-1:IMSS]	YSGIIELVM*K	1168.629335	2	2.0839
gi|56472257|gb|EAL49755.1| U-box domain, putative [Entamoeba histolytica HM-1:IMSS]	LSDCVYLDKTLLEM*IKK	2085.076674	2	1.6784
gi|56472270|gb|EAL49767.1| hypothetical protein 35.t00001 [Entamoeba histolytica HM-1:IMSS]	TLDNKTDVK	1033.552419	2	2.133
gi|56472283|gb|EAL49780.1| histone H2B [Entamoeba histolytica HM-1:IMSS]	IATEASSLAR	1018.552773	2	2.779
	IATEASSLAR	1018.552773	1	1.569
gi|56472297|gb|EAL49792.1| ubiquitin [Entamoeba histolytica HM-1:IMSS]	ESTLHLVLR	1067.620782	2	1.7217
gi|56472303|gb|EAL49798.1| MAK16 protein, putative [Entamoeba histolytica HM-1:IMSS]	EERRDITR	1074.565049	2	1.8311
gi|56472336|gb|EAL49829.1| SMC3 protein, putative [Entamoeba histolytica HM-1:IMSS]	RKELIKR	942.620711	2	1.613
	SEIEKAKK	932.541135	2	1.9147
gi|56472348|gb|EAL49839.1| hypothetical protein 33.t00032 [Entamoeba histolytica HM-1:IMSS]	EQISEPVLDK	1157.604859	3	2.3487
gi|56472364|gb|EAL49854.1| adenylyl cyclase-associated protein, putative [Entamoeba histolytica HM-1:IMSS]	GGVASAQAAPVAPK	1223.67428	2	3.9386
gi|56472365|gb|EAL49855.1| calreticulin, putative [Entamoeba histolytica HM-1:IMSS]	AGTIYDDILITDDIEEAEK	2124.023063	2	4.0149
	KPEEWNDEDDGEWEAPTIENPEYK	2920.243183	3	4.2965
gi|56472374|gb|EAL49864.1| hypothetical protein 33.t00015 [Entamoeba histolytica HM-1:IMSS]	VPSKSINEIINLCFSFK	1996.03578	2	2.1313
gi|56472428|gb|EAL49914.1| hypothetical protein 32.t00021 [Entamoeba histolytica HM-1:IMSS]	NTLALTHKNLCNQGK	1711.869355	3	1.7884
gi|56472449|gb|EAL49933.1| hypothetical protein 31.t00002 [Entamoeba histolytica HM-1:IMSS]	MLECVEKIGEAAECR	1794.775379	2	1.7079
gi|56472455|gb|EAL49939.1| Rho GTPase activating protein, putative [Entamoeba histolytica HM-1:IMSS]	TM*NEETLMSINK	1426.656354	3	1.5816
gi|56472512|gb|EAL49994.1| Sec7 domain protein [Entamoeba histolytica HM-1:IMSS]	LENSIDVIK	1030.577915	2	1.6988
gi|56472515|gb|EAL49997.1| Rho guanine nucleotide exchange factor, putative [Entamoeba histolytica HM-1:IMSS]	SAIQKESDKKFSK	1495.811518	2	2.2841
gi|56472516|gb|EAL49998.1| protein disulfide isomerase, putative [Entamoeba histolytica HM-1:IMSS]	FVLENVPAKN	1130.620438	2	1.9259
	GFPTVLVFNGEESK	1523.774055	2	2.272
gi|56472550|gb|EAL50029.1| galactose-inhibitable lectin 170 kda subunit [Entamoeba histolytica HM-1:IMSS]	ATVQPTPACSVCESGK	1691.72983	2	2.8999
	CKVSNCTEDLVR	1480.645359	2	2.0086
	CKVSNCTEDLVR	1480.645359	2	2.0047
	HQICDYTGTTPK	1420.631082	2	3.6497
	TYTYDSK	877.393803	2	2.0112
	YEVDWDNVPVDEQR	1763.787126	2	4.8443
	YTAGTGQVDPLINIAK	1660.890472	2	3.4829
gi|56472629|gb|EAL50099.1| 60S ribosomal protein L23a, putative [Entamoeba histolytica HM-1:IMSS]	FPLATETALK	1090.61429	2	2.6992
	LAPDVEAMEVATK	1373.698097	2	1.7005
	NKMDEFSIIK	1224.629298	2	2.0895
gi|56472643|gb|EAL50113.1| hypothetical protein 28.t00027 [Entamoeba histolytica HM-1:IMSS]	IYLKYKYQTYEYEK	1931.978948	3	1.622
gi|56472695|gb|EAL50160.1| hypothetical protein 27.t00007 [Entamoeba histolytica HM-1:IMSS]	NGFISINDINK	1234.642643	2	1.5372
gi|56472744|gb|EAL50207.1| protein kinase, putative [Entamoeba histolytica HM-1:IMSS]	IAQGSFSIVFKGK	1381.78384	2	1.7383
gi|56472798|gb|EAL50257.1| conserved hypothetical protein [Entamoeba histolytica HM-1:IMSS]	CSKELIEVKNEK	1476.751205	2	2.5671
gi|56472807|gb|EAL50266.1| histone H4 [Entamoeba histolytica HM-1:IMSS]	DSVTYTEHAK	1150.537508	2	2.7437
	GITKPAIR	855.5410664	2	1.6569
	QFLEQVIR	1032.583658	2	2.2553
gi|56472825|gb|EAL50283.1| 60S ribosomal protein L19, putative [Entamoeba histolytica HM-1:IMSS]	ASVQVTK	732.425042	2	2.1164
	KPVVAEEPK	996.572425	2	2.0832
	LAASVLK	701.455613	2	1.5841
	VWLDPNETPEISLANSR	1940.971258	2	3.4263

	VWLDPNETPEISLANSR	1940.971258	2	3.4111
	VWLDPNETPEISLANSR	1940.971258	2	2.8362
gi|56472855|gb|EAL50313.1| hypothetical protein 24.t00040 [Entamoeba histolytica HM-1:IMSS]	AAAAYGDVEK	994.4840074	2	2.5434
	EKEEIEKEEVSIK	1589.826875	2	2.9152
	EKEEIEKEEVSIK	1589.826875	3	2.1517
	FGDYSCNGEK	1176.441169	2	2.3972
	GETSIDIDTLIEEK	1562.779593	2	4.2087
	KAAAAYGDVEK	1122.57897	2	2.0233
gi|56472943|gb|EAL50397.1| hypothetical protein 23.t00026 [Entamoeba histolytica HM-1:IMSS]	NNSIIVEEPTGK	1300.674338	2	2.2248
gi|56472985|gb|EAL50436.1| villin-related protein [Entamoeba histolytica HM-1:IMSS]	KNPMIPFDDQK	1332.661651	2	1.6144
gi|56473057|gb|EAL50505.1| hypothetical protein 21.t00051 [Entamoeba histolytica HM-1:IMSS]	KASSLKKSMK	1107.655472	2	1.845
gi|56473090|gb|EAL50536.1| Rab family GTPase [Entamoeba histolytica HM-1:IMSS] gi|56790126|dbj|BAD82859.1| EhRabX	FNEQSIPALLVGNK	1529.832235	2	2.5834
	FNEQSIPALLVGNK	1529.832235	2	2.002
	FTTVDER	867.420676	2	1.5625
gi|56473093|gb|EAL50539.1| Rab family GTPase [Entamoeba histolytica HM-1:IMSS] gi|13537451|dbj|BAB40680.1| small GT	SLTSIPM*WITEK	1421.7356	2	2.2487
gi|56473102|gb|EAL50548.1| hypothetical protein 20.t00044 [Entamoeba histolytica HM-1:IMSS]	KERSVAVNK	1030.600381	2	1.5725
gi|56473114|gb|EAL50560.1| hypothetical protein 20.t00058 [Entamoeba histolytica HM-1:IMSS]	GGEEGYYSKMKVK	1475.719912	2	2.1293
gi|56473156|gb|EAL50599.1| ubiquitin-like protein [Entamoeba histolytica HM-1:IMSS]	VVTQDSTEVFFK	1399.710387	2	1.744
gi|56473173|gb|EAL50616.1| hypothetical protein 19.t00056 [Entamoeba histolytica HM-1:IMSS]	YGYGNTFEFK	1225.552424	2	2.7827
gi|56473193|gb|EAL50635.1| phosphatidylinositol-3,4,5-trisphosphate 3-phosphatase, putative [Entamoeba histolytica HM-1:IM	DPQNVIALHCKAGK	1550.789313	3	1.8689
gi|56473196|gb|EAL50638.1| 60S ribosomal protein L18, putative [Entamoeba histolytica HM-1:IMSS]	VNRPIVSTSK	1100.642256	3	2.9216
gi|56473199|gb|EAL50641.1| hypothetical protein 19.t00014 [Entamoeba histolytica HM-1:IMSS]	NTFIKSGM*SDLGCSSK	1747.77863	3	1.7189
gi|56473238|gb|EAL50677.1| hypothetical protein 18.t00058 [Entamoeba histolytica HM-1:IMSS]	GKKDEVISQEDEK	1504.748961	3	1.6057
gi|56473301|gb|EAL50735.1| 40S ribosomal protein S13, putative [Entamoeba histolytica HM-1:IMSS]	YHLILIESK	1115.645934	2	2.6435
gi|56473304|gb|EAL50738.1| hypothetical protein 17.t00035 [Entamoeba histolytica HM-1:IMSS]	AGQNIK	630.3569544	2	1.5886
gi|56473325|gb|EAL50759.1| galactose-inhibitable lectin 35 kda subunit precursor [Entamoeba histolytica HM-1:IMSS]	GGANEGATLIIWPK	1426.768901	2	3.3043
	HFYLPYR	995.509764	2	2.091
	SYQIIASR	937.510179	2	2.5546
gi|56473368|gb|EAL50797.1| Gal/GalNAc lectin heavy subunit [Entamoeba histolytica HM-1:IMSS]	TCSLTETK	939.423722	2	1.9886
	YTAGQGQVDPLVNIAK	1673.885721	2	3.2259
gi|56473377|gb|EAL50806.1| hypothetical protein 16.t00023 [Entamoeba histolytica HM-1:IMSS]	QPSPYIQIDGSK	1332.679433	3	1.8138
gi|56473385|gb|EAL50814.1| hypothetical protein 16.t00031 [Entamoeba histolytica HM-1:IMSS]	ETTTKELFNINNFSKR	1942.002882	2	1.7
	QAIEEMILNPSK	1372.714091	3	1.5233
gi|56473405|gb|EAL50834.1| ubiquitin carboxyl-terminal hydrolase, putative [Entamoeba histolytica HM-1:IMSS]	EDPQIYYLR	1196.594618	3	1.5485
	KDIELELNK	1101.615019	2	1.923
gi|56473474|gb|EAL50899.1| 40S ribosomal protein S9, putative [Entamoeba histolytica HM-1:IMSS]	LLEGAALLR	955.5934964	2	3.4095
	LVGQFGLK	861.5192708	2	1.5055
	QVVDVPSFLVR	1258.715411	2	2.0995
gi|56473526|gb|EAL50948.1| hypothetical protein 14.t00037 [Entamoeba histolytica HM-1:IMSS]	IELSKKKK	973.640454	2	2.2163
gi|56473552|gb|EAL50971.1| acyl-CoA synthetase, putative [Entamoeba histolytica HM-1:IMSS]	GDACGIM*TR	996.4033668	2	1.5896
gi|56473599|gb|EAL51018.1| hypothetical protein 13.t00064 [Entamoeba histolytica HM-1:IMSS]	WCQAIKMNAVK	1348.66496	3	1.6768
gi|56473610|gb|EAL51028.1| hypothetical protein 13.t00004 [Entamoeba histolytica HM-1:IMSS]	EKGPKPNKNEDK	1383.722675	2	2.0789
gi|56473612|gb|EAL51030.1| hypothetical protein 13.t00006 [Entamoeba histolytica HM-1:IMSS]	DLLDQLEASR	1159.595357	2	3.3833
gi|56473650|gb|EAL51065.1| ubiquitin carboxyl-terminal hydrolase, putative [Entamoeba histolytica HM-1:IMSS]	ITNNDIEEAIMK	1390.688261	2	1.556
gi|56473818|gb|EAL51221.1| hypothetical protein 10.t00051 [Entamoeba histolytica HM-1:IMSS]	SLDEVCNTLGCK	1395.581364	2	3.819
	YCNFITPK	1042.481166	2	1.9992
	YPTSDDICR	1126.461899	2	2.2322
gi|56473822|gb|EAL51224.1| actin [Entamoeba histolytica HM-1:IMSS]	EEYDESGPAIVHR	1501.69178	2	2.9577
	GYAFTTTAER	1116.53202	2	2.9442
	LCYVAEDFNEEMQK	1775.740039	2	4.618
	SYELPDGQVITVGNER	1776.87629	2	4.5123

	SYELPDGQVITVGNER	1776.87629	2	3.4406
	VAPEEHPVLLTEAPMNPK	1972.020829	2	4.2097
	VIAPPER	781.456666	2	1.6428
gi|56473886|gb|EAL51285.1| histone H2A, putative [Entamoeba histolytica HM-1:IMSS]	EKEVTPVVEK	1157.641234	2	2.4385
gi|56473892|gb|EAL51291.1| ADP-ribosylation factor, putative [Entamoeba histolytica HM-1:IMSS]	NISFTVWDVGGQDK	1565.759468	2	3.182
gi|56473899|gb|EAL51298.1| hypothetical protein 9.t00060 [Entamoeba histolytica HM-1:IMSS]	KFDEGEIKRK	1249.689917	3	2.2879
gi|56473961|gb|EAL51355.1| actin, putative [Entamoeba histolytica HM-1:IMSS]	AGFAGDDAPR	976.4482928	2	2.3545
	DAYVGDEAQSK	1182.52734	2	2.7654
	DAYVGDEAQSK	1182.52734	2	2.4927
	YPIEHGIVNNWDDMEK	1959.890546	3	3.3074
gi|56473974|gb|EAL51368.1| Rho GTPase activating protein, putative [Entamoeba histolytica HM-1:IMSS]	KLQNVLK	842.545815	2	1.5059
gi|56474013|gb|EAL51407.1| Vesicle-fusing ATPase, putative [Entamoeba histolytica HM-1:IMSS]	STQSVIIDDLER	1488.790439	2	1.9351
gi|56474044|gb|EAL51435.1| 40S ribosomal protein S23, putative [Entamoeba histolytica HM-1:IMSS]	DGSLNYVNENDEVLISGFGR	2198.036052	2	4.6988
	DGSLNYVNENDEVLISGFGR	2198.036052	2	2.8796
	DGSLNYVNENDEVLISGFGR	2198.036052	3	2.1889
	GHAVGDIPGVR	1077.579977	2	2.8412
	ITAFVPR	803.477401	2	1.781
	RGHAVGDIPGVR	1233.681088	3	3.004
gi|56474045|gb|EAL51436.1| Rab family GTPase [Entamoeba histolytica HM-1:IMSS] gi|50978604|dbj|BAD34969.1| EhRab7I	IIILGDSGVGK	1071.640856	2	2.2811
gi|56474066|gb|EAL51455.1| hypothetical protein 7.t00052 [Entamoeba histolytica HM-1:IMSS]	SLLASGLSEEEAEK	1462.727183	2	2.9275
gi|56474108|gb|EAL51496.1| Sec7 domain protein [Entamoeba histolytica HM-1:IMSS]	LIDFTGLNIDEALR	1589.853355	3	1.8685
gi|56474110|gb|EAL51498.1| hypothetical protein 7.t00096 [Entamoeba histolytica HM-1:IMSS]	LQLEQIQPATEQEVK	1753.933061	2	3.0807
gi|56474130|gb|EAL51513.1| casein kinase, putative [Entamoeba histolytica HM-1:IMSS]	LYGLIHARFILSPNGM*KK	2074.164128	3	1.9634
gi|56474132|gb|EAL51515.1| hypothetical protein 6.t00008 [Entamoeba histolytica HM-1:IMSS]	M*PLLNGDKWK	1217.635807	2	1.8865
gi|56474188|gb|EAL51571.1| Ras guanine nucleotide exchange factor, putative [Entamoeba histolytica HM-1:IMSS]	KDDKINK	860.48361	2	1.5114
gi|56474194|gb|EAL51577.1| hypothetical protein 6.t00078 [Entamoeba histolytica HM-1:IMSS]	KFIEFNTIRKLVR	1664.000624	3	2.0797
gi|56474237|gb|EAL51617.1| hypothetical protein 5.t00077 [Entamoeba histolytica HM-1:IMSS]	NKKYIGDAQLNK	1391.764145	3	2.1252
gi|56474246|gb|EAL51626.1| conserved hypothetical protein [Entamoeba histolytica HM-1:IMSS]	AFNELIEFR	1138.589138	2	3.3446
	AFNELIEFR	1138.589138	2	2.2597
	EGLVVVDER	1015.541856	2	2.2599
	EVTEVIEK	946.509157	2	1.6563
	FEVVSEATENVPK	1448.726766	2	4.0438
	FVNFDQAAEDAAK	1425.66449	2	4.0896
	QFEFDDIAK	1112.52587	2	1.8198
	QNKEETFAYECGELLR	1986.901111	3	2.7418
gi|56474249|gb|EAL51629.1| protein kinase, putative [Entamoeba histolytica HM-1:IMSS]	TSDKSKSDSNNK	1310.618309	2	1.8301
gi|56474266|gb|EAL51645.1| myosin heavy chain [Entamoeba histolytica HM-1:IMSS]	EAAENALASATAK	1246.627386	2	2.925
	FNLEDVTNQR	1235.601495	2	2.465
	FQINAPSSVAQEEEK	1676.812632	2	2.5352
gi|56474285|gb|EAL51664.1| hypothetical protein 5.t00057 [Entamoeba histolytica HM-1:IMSS]	NTAKNNQEEFAK	1393.670638	2	1.9023
gi|56474301|gb|EAL51679.1| calcineurin catalytic subunit, putative [Entamoeba histolytica HM-1:IMSS]	KAVSDILKR	1029.641517	2	1.6185
gi|56474455|gb|EAL51822.1| chaperonin containing TCP-1 eta subunit, putative [Entamoeba histolytica HM-1:IMSS]	VEEDDMKR	1021.461889	2	1.5878
gi|56474489|gb|EAL51856.1| hypothetical protein 3.t00137 [Entamoeba histolytica HM-1:IMSS]	SKVNSPKQETELEKVLK	1957.096458	3	1.8636
gi|56474504|gb|EAL51870.1| hypothetical protein 3.t00088 [Entamoeba histolytica HM-1:IMSS]	TCIVNLSSLNSKK	1463.767209	2	1.7024
gi|56474534|gb|EAL51899.1| hypothetical protein 3.t00026 [Entamoeba histolytica HM-1:IMSS]	SVTFLNNQSPK	1234.642651	2	1.7854
gi|56474536|gb|EAL51901.1| eukaryotic initiation factor 4A, putative [Entamoeba histolytica HM-1:IMSS]	NLIAQAQSGTGK	1187.637895	2	2.0053
	NNVLNEFR	1005.511222	2	1.6513
gi|56474538|gb|EAL51903.1| hypothetical protein 3.t00030 [Entamoeba histolytica HM-1:IMSS]	DIEEMRK	920.450595	2	1.5122
gi|56474579|gb|EAL51944.1| U5 snRNP-specific 200kd protein, putative [Entamoeba histolytica HM-1:IMSS]	TRVDNNLVISNRRGK	1741.978005	2	1.7404
gi|56474653|gb|EAL52015.1| hypothetical protein 2.t00091 [Entamoeba histolytica HM-1:IMSS]	LVNAEK	673.387918	2	1.7974
gi|56474731|gb|EAL52088.1| hypothetical protein 1.t00093 [Entamoeba histolytica HM-1:IMSS]	NMLSPSELKTDGK	1419.714832	3	1.6335

gi|56474774|gb|EAL52131.1| hypothetical protein 1.t00136 [Entamoeba histolytica HM-1:IMSS]	VEIIEQKVYR	1276.725966	2	1.5048
gi|56474796|gb|EAL52153.1| proteasome beta subunit, putative [Entamoeba histolytica HM-1:IMSS]	NGVEEKLNIEHPNDRK	1891.962072	3	1.6724
gi|56474797|gb|EAL52154.1| hypothetical protein 1.t00154 [Entamoeba histolytica HM-1:IMSS]	EEIKKSEPILPK	1410.820271	2	1.5168
gi|56474856|gb|EAL52210.1| DNA ligase, putative [Entamoeba histolytica HM-1:IMSS]	EVNDITTNK	1033.516034	2	1.9772
gi|7494130|pir||T18292 nicotinamide nucleotide transhydrogenase homolog - Entamoeba histolytica	SGETYLYDNR	1217.543324	2	2.9241
gi|7579069|gb|AAF64243.1| hsp70 BiP [Entamoeba invadens]	QIEQIAQPILAK	1351.79438	2	2.8936
