# Supplementary material for: Entamoeba histolytica Phagocytosis of Human Erythrocytes Involves PATMK, a Member of the Transmembrane Kinase Family
Source: PLoS Pathog. 2008 Jan 18;4(1):e8. doi: 10.1371/journal.ppat.0040008 (PMC2211552; doi:10.1371/journal.ppat.0040008)
Supplement: Supplementary file 3 [file ppat.0040008.st003.pdf]

10 MINUTES

ANNOTATION	PEPTIDE SEQUENCE	MASS	CHARGE	Xcorr
gi|10312090|gb|AAG16651.1| mitochondrial-type hsp70 [Entamoeba histolytica]	DISDVVLVGGMTRMPLIQTLSR	2401.294192	2	1.5315
gi|1905981|gb|AAB50229.1| Gal/GalNAc lectin heavy subunit [Entamoeba dispar]	SLQEKLSTLNQLTTVK	1803.02223	3	3.4321
	SLQEKLSTLNQLTTVK	1803.02223	2	2.2696
gi|20454165|gb|AAM22192.1| Gal/GalNAc lectin heavy subunit region B [Entamoeba histolytica]	YETLVQYTAGTGQVDPLINIAK	2394.25513	2	4.6386
gi|2350917|dbj|BAA21988.1| actin [Entamoeba histolytica]	YPLEHGIVNNWDDMEK	1959.890546	2	5.8475
	YPLEHGIVNNWDDMEK	1959.890546	3	4.5653
	YPLEHGIVNNWDDMEK	1959.890546	2	4.3883
	YPLEHGIVNNWDDMEK	1959.890546	3	4.2506
	YPLEHGIVNNWDDMEK	1959.890546	2	2.9015
gi|2498767|sp|Q24824|PFPB_ENTHI Pore-forming peptide ameobapore B precursor (EH-APP)	ILSFGVDELVK	1219.693281	2	2.7961
gi|31414729|gb|AAP50909.1| chaperonin 60 [Entamoeba dispar]	IEDAVYAVKAALA	1333.736197	3	1.5943
gi|34392537|dbj|BAC82596.1| reverse transcriptase [Entamoeba histolytica]	VKHVLYMDDIKVTSETK	2006.062704	2	1.7018
gi|51102320|gb|AAT95873.1| prolyl-tRNA synthetase [Entamoeba terrapinae]	VMDEM*HNDM*YAKALK	1827.809602	2	1.9062
gi|56463309|gb|EAL42451.1| 40S ribosomal protein S6, putative [Entamoeba histolytica HM-1:IMSS]	DGFPMMTGVATNNR	1510.677717	2	2.0601
	GEGEIEGVTNDALPMR	1687.795588	2	1.9059
	KGEGEIEGVTNDALPMR	1815.890551	3	3.0159
	KGEGEIEGVTNDALPMR	1815.890551	2	2.3424
	LFNLSQK	849.482891	2	2.3191
	LFNLSQK	849.482891	2	1.9226
	LFNLSQK	849.482891	1	1.8512
	VVSFADTDAVFK	1298.662708	2	3.8737
	YAAILNR	820.467565	2	2.2342
	YAAILNR	820.467565	2	1.9703
	YAAILNR	820.467565	1	1.8916
gi|56463457|gb|EAL42479.1| splicing factor 3B subunit 1, putative [Entamoeba histolytica HM-1:IMSS]	M*SNSSESESRHRRR	1734.806005	3	1.6343
gi|56463528|gb|EAL42505.1| hypothetical protein 856.t00001 [Entamoeba histolytica HM-1:IMSS]	QQQPNPAGLFAELNK	1654.854752	2	1.7476
gi|56463554|gb|EAL42513.1| phage integrase, putative [Entamoeba histolytica HM-1:IMSS]	SLSARLYEK	1066.589158	2	1.858
gi|56463561|gb|EAL42516.1| GTP-binding protein, putative [Entamoeba histolytica HM-1:IMSS]	GADEEK	648.2835164	2	1.6857
gi|56463575|gb|EAL42521.1| peroxiredoxin [Entamoeba histolytica HM-1:IMSS]	FINTFEK	898.466897	1	2.1523
	FINTFEK	898.466897	2	2.0917
	FINTFEK	898.466897	2	2.0078
	GKVRYIQMNDDGIGR	1721.875174	2	1.7043
	GYVIIDDK	922.4880294	1	2.6825
	LTFPLVSDIK	1132.66125	2	2.8713
	LTFPLVSDIKR	1288.762361	2	1.5629
	YIQMNDDGIGR	1281.58922	2	3.1912
	YIQMNDDGIGR	1281.58922	2	3.0166
gi|56463666|gb|EAL42549.1| Ras guanine nucleotide exchange factor, putative [Entamoeba histolytica HM-1:IMSS]	VVEGAVAAVIK	1055.645926	2	1.8937
gi|56463710|gb|EAL42562.1| Rab family GTPase [Entamoeba histolytica HM-1:IMSS] gi|50978618|dbj|BAD34976.1| EhRab1	AQIWDTAGQER	1274.612396	2	3.0773
	SNLLLR	715.446111	2	1.9632
	WLSELKDNADPK	1415.716535	2	2.1706
gi|56463761|gb|EAL42580.1| hypothetical protein 692.t00003 [Entamoeba histolytica HM-1:IMSS]	ALDQEETGDLIK	1331.668909	3	1.6357
gi|56463820|gb|EAL42602.1| protein kinase, putative [Entamoeba histolytica HM-1:IMSS]	ISKNGSTSAR	1020.543283	2	1.7318
gi|56463825|gb|EAL42604.1| hypothetical protein 650.t00002 [Entamoeba histolytica HM-1:IMSS]	FDKNITSVEIK	1293.704907	3	1.6223
gi|56463826|gb|EAL42605.1| protein kinase, putative [Entamoeba histolytica HM-1:IMSS]	TNPQLMTIPK	1142.623808	2	1.8989
gi|56463845|gb|EAL42613.1| hypothetical protein 641.t00001 [Entamoeba histolytica HM-1:IMSS]	IQVNSPSSSFSSPDYIENVK	2198.061237	2	4.1974
	IQVNSPSSSFSSPDYIENVK	2198.061237	2	2.1046
	EIEKEVIR	1015.578239	1	2.37
gi|56463888|gb|EAL42631.1| hypothetical protein 626.t00002 [Entamoeba histolytica HM-1:IMSS]	MQEGVSM*TGSIKSK	1498.725127	2	1.6275

gi|56463918|gb|EAL42646.1| calcium-binding protein, putative [Entamoeba histolytica HM-1:IMSS]	PFNPMEFGK	1066.502632	2	2.0274
	TSLLLHR	839.509774	2	2.3096
gi|56463946|gb|EAL42661.1| hypothetical protein 603.t00002 [Entamoeba histolytica HM-1:IMSS]	LIVMIVLYLS	1163.710841	2	1.7335
gi|56463954|gb|EAL42664.1| RNA pseudouridylate synthase, putative [Entamoeba histolytica HM-1:IMSS]	QQDGEINRENK	1330.634589	3	1.924
gi|56463993|gb|EAL42683.1| hypothetical protein 586.t00002 [Entamoeba histolytica HM-1:IMSS]	EHFTPKRIIVIQMK	1739.998909	3	1.8881
gi|56464041|gb|EAL42707.1| hypothetical protein 573.t00004 [Entamoeba histolytica HM-1:IMSS]	ETQNRIK	888.489758	2	1.8767
gi|56464053|gb|EAL42712.1| hypothetical protein 568.t00002 [Entamoeba histolytica HM-1:IMSS]	LNGINILNK	998.5993104	1	1.5144
gi|56464071|gb|EAL42720.1| hypothetical protein 564.t00002 [Entamoeba histolytica HM-1:IMSS]	ETKKEVK	861.504011	2	1.9292
gi|56464126|gb|EAL42749.1| heat shock protein 70, putative [Entamoeba histolytica HM-1:IMSS]	IINEPTAAAIAYGLDK	1659.89522	2	3.9451
gi|56464158|gb|EAL42768.1| ubiquitin [Entamoeba histolytica HM-1:IMSS]	MQIFVK	765.432758	1	1.5515
	TITLEVEPNDSIDAIK	1757.916753	2	2.3518
	TITLEVEPNDSIDAIK	1757.916753	2	2.2824
	TITLEVEPNDSIDAIK	1757.916753	2	1.7593
	TITLEVEPNDSIDAIK	1757.916753	2	1.576
gi|56464189|gb|EAL42779.1| snoRNA binding protein, putative [Entamoeba histolytica HM-1:IMSS]	VIASKAALCAR	1159.640136	3	1.8145
gi|56464202|gb|EAL42786.1| 40S ribosomal protein S6, putative [Entamoeba histolytica HM-1:IMSS]	IGGQEVDASSLGDEFK	1594.759539	2	4.0258
	IGGQEVDASSLGDEFK	1594.759539	2	3.9255
gi|56464231|gb|EAL42808.1| hypothetical protein 521.t00002 [Entamoeba histolytica HM-1:IMSS]	DIWKHVCIVWTK	1584.814068	2	1.607
gi|56464235|gb|EAL42810.1| DNA polymerase, putative [Entamoeba histolytica HM-1:IMSS]	KAYLDILTNEDGVEEYLIR	2254.160164	2	2.7781
gi|56464240|gb|EAL42814.1| coatomer protein gamma subunit, putative [Entamoeba histolytica HM-1:IMSS]	PLRSPYAHVELLK	1522.874037	3	1.8809
gi|56464317|gb|EAL42862.1| hypothetical protein 502.t00006 [Entamoeba histolytica HM-1:IMSS]	KEEETNMSQLIDKMKILYK	2341.214196	3	2.0365
gi|56464322|gb|EAL42865.1| hypothetical protein 501.t00002 [Entamoeba histolytica HM-1:IMSS]	MEEVVNNQLYK	1366.667131	3	2.0213
gi|56464326|gb|EAL42866.1| serine-rich protein [Entamoeba histolytica HM-1:IMSS]	NEASPEKLEEAEEK	1602.749354	2	1.6702
gi|56464382|gb|EAL42899.1| Truncated pyridine nucleotide transhydrogenase [Entamoeba histolytica HM-1:IMSS]	LPGHMNVLLAEANVPYK	1865.994221	2	4.1067
gi|56464491|gb|EAL42972.1| translation elongation factor EF-1 alpha, putative [Entamoeba histolytica HM-1:IMSS]	GPTLIGALDSVTPPER	1622.874832	2	4.3097
	GPTLIGALDSVTPPERPVDK	2062.117917	2	4.2965
	GPTLIGALDSVTPPERPVDK	2062.117917	2	3.3381
	GPTLIGALDSVTPPERPVDK	2062.117917	2	1.9597
	GPTLIGALDSVTPPERPVDKPLR	2428.355856	3	2.2894
	LPLQDVYK	975.550961	2	2.8379
	LPLQDVYK	975.550961	2	2.7547
	LPLQDVYK	975.550961	2	2.4217
	LPLQDVYK	975.550961	1	1.9047
	LPLQDVYK	975.550961	1	1.899
	MDAIQYK	868.423317	1	2.0546
	MDAIQYK	868.423317	2	1.8579
	QMIVGVNK	888.4971524	2	2.0198
	QMIVGVNK	888.4971524	1	1.9149
	QTVAVGVVK	900.5512974	2	2.1868
	SM*EGGEPEYIK	1255.552212	2	2.2631
	SMEGGEPEYIK	1239.556199	2	3.5352
	STTTGHLIYK	1120.599715	2	2.9535
	STTTGHLIYK	1120.599715	1	1.9127
	SVEMHHTALAQAIPGDNVGFNVR	2463.219773	2	4.9046
	SVEMHHTALAQAIPGDNVGFNVR	2463.219773	2	4.1398
	YAWVLDNLK	1121.598974	1	3.0864
	YAWVLDNLK	1121.598974	2	3.0079
	YAWVLDNLK	1121.598974	2	2.9604
	YAWVLDNLK	1121.598974	1	2.9154
	YAWVLDNLK	1121.598974	2	2.5183
	YYFTIIDAPGHR	1452.727031	2	3.5649

	YYFTIIDAPGHR	1452.727031	2	3.5149
gi|56464518|gb|EAL42993.1| Sec61 alpha subunit, putative [Entamoeba histolytica HM-1:IMSS]	GSLMELGISPIVTASMVMQLLQGAK	2574.370405	2	3.6498
	PIVSLIPTINEPTK	1521.888685	2	3.1468
gi|56464535|gb|EAL43006.1| hypothetical protein 465.t00009 [Entamoeba histolytica HM-1:IMSS]	EVSMEDVKTLVEM*TGVSK	1997.978096	3	1.6456
gi|56464545|gb|EAL43014.1| Ras guanine nucleotide exchange factor, putative [Entamoeba histolytica HM-1:IMSS]	LDRHSLSLTFRK	1472.833245	3	1.6575
gi|56464598|gb|EAL43055.1| hypothetical protein 456.t00003 [Entamoeba histolytica HM-1:IMSS]	DCCYYTKKEGGK	1508.607906	2	2.0258
gi|56464614|gb|EAL43068.1| 40S ribosomal protein S15, putative [Entamoeba histolytica HM-1:IMSS]	SLEEITAMPEADFVK	1679.81968	2	1.9823
gi|56464675|gb|EAL43119.1| cysteine protease 13 [Entamoeba histolytica HM-1:IMSS]	PEEFARRFNK	1293.669848	2	1.8545
gi|56464682|gb|EAL43124.1| hypothetical protein 444.t00004 [Entamoeba histolytica HM-1:IMSS]	REMPLSQQEILLIKKIDK	2182.2628	2	1.5793
gi|56464739|gb|EAL43164.1| hypothetical protein 434.t00004 [Entamoeba histolytica HM-1:IMSS]	AREVQSTPQTPTTPKGNR	1968.025745	2	1.8073
gi|56464766|gb|EAL43182.1| Plasma membrane calcium-transporting ATPase, putative [Entamoeba histolytica HM-1:IMSS]	QDATTPFEISGNSLYNLVGNK	2268.1143	3	1.6508
gi|56464799|gb|EAL43208.1| 40S ribosomal protein S3a, putative [Entamoeba histolytica HM-1:IMSS]	ELTQFVDYLR	1283.663032	2	3.4791
	ELTQFVDYLR	1283.663032	2	3.3294
	ELTQFVDYLR	1283.663032	2	3.088
	FGAYNYK	862.4093844	2	2.1007
	FGAYNYK	862.4093844	2	1.6725
	IAEPFSK	791.429793	1	1.6959
	IMNNLITK	946.539015	2	2.2529
	IMNNLITK	946.539015	1	1.6291
	KEWYNVK	966.504345	2	2.0004
	TTDGHLIR	912.4897604	2	2.285
	TTDGHLIR	912.4897604	2	1.951
	TVGQIVVNK	957.5727614	2	2.5122
	TVGQIVVNK	957.5727614	2	2.4227
	TVGQIVVNK	957.5727614	1	1.8636
	TVGQIVVNK	957.5727614	1	1.7443
	VFSMDNVYIR	1243.613982	2	3.3313
	VFSMDNVYIR	1243.613982	2	2.8842
	VFSMDNVYIR	1243.613982	1	2.2519
	VFSMDNVYIR	1243.613982	1	1.7227
	VVEVNLADLMENEK	1602.804354	2	5.2615
	VVEVNLADLMENEK	1602.804354	2	5.0723
	VVEVNLADLMENEK	1602.804354	2	4.8093
	VVEVNLADLMENEK	1602.804354	2	4.7222
	VVEVNLADLMENEK	1602.804354	2	2.615
gi|56464830|gb|EAL43231.1| hypothetical protein 420.t00002 [Entamoeba histolytica HM-1:IMSS]	DPLLSFAILQKNEISIR	1957.111713	2	1.6042
gi|56464866|gb|EAL43255.1| hypothetical protein 416.t00006 [Entamoeba histolytica HM-1:IMSS]	KDISDLK	818.461822	2	1.9069
gi|56464869|gb|EAL43257.1| cortexillin, putative [Entamoeba histolytica HM-1:IMSS]	TQHETLTNELNDMEK	1802.822525	2	2.384
gi|56464881|gb|EAL43268.1| DEAD/DEAH box helicase, putative [Entamoeba histolytica HM-1:IMSS]	ENRFLLPGEYTQM*AGRAGR	2182.083306	2	1.8748
gi|56464975|gb|EAL43340.1| hypothetical protein 401.t00006 [Entamoeba histolytica HM-1:IMSS]	NETVINGEWIEVSSRNKTENK	2447.216137	3	1.8299
gi|56464998|gb|EAL43357.1| hypothetical protein 398.t00003 [Entamoeba histolytica HM-1:IMSS]	EVNLKVDVPPK	1237.715067	2	1.9957
gi|56465001|gb|EAL43360.1| interaptin, putative [Entamoeba histolytica HM-1:IMSS]	NDVNALLGQINDYVK	1675.864983	2	1.8145
	NDVNALLGQINDYVK	1675.864983	2	1.6543
gi|56465052|gb|EAL43399.1| hypothetical protein 393.t00006 [Entamoeba histolytica HM-1:IMSS]	IIVNEEFNKEYSR	1640.827877	2	1.5297
gi|56465062|gb|EAL43407.1| hypothetical protein 391.t00002 [Entamoeba histolytica HM-1:IMSS]	NYKKIGGLNEVIK	1475.858048	2	2.4343
gi|56465086|gb|EAL43425.1| hypothetical protein 388.t00005 [Entamoeba histolytica HM-1:IMSS]	SFIHLALRISGENEAMGVNIK	2299.222742	2	1.7046
gi|56465087|gb|EAL43426.1| hypothetical protein 388.t00006 [Entamoeba histolytica HM-1:IMSS]	EEEYLFADIILDNEEIK	2083.011767	2	2.0792
	EQEIIQNNQIITIK	1683.927581	2	1.9186
	MIELIKKKEMEQLINDK	2103.155213	2	2.3654
gi|56465099|gb|EAL43433.1| phospholipid-transporting P-type ATPase, putative [Entamoeba histolytica HM-1:IMSS]	FKHLGKLILYHGR	1581.937634	2	1.6649
gi|56465108|gb|EAL43438.1| acyl-CoA synthetase, putative [Entamoeba histolytica HM-1:IMSS]	TGDIGEIVDGQIKIIDRK	1970.091694	2	1.7617

gi|56465135|gb|EAL43460.1| hypothetical protein 380.t00002 [Entamoeba histolytica HM-1:IMSS]	GNILILFEGNEGNVFGISLENM*K	2524.276326	3	1.8129
gi|56465220|gb|EAL43528.1| I/LWEQ domain protein [Entamoeba histolytica HM-1:IMSS]	KVIGRMK	831.5233064	2	1.5031
gi|56465234|gb|EAL43540.1| pescadillo, putative [Entamoeba histolytica HM-1:IMSS]	TKPVIDLASTIQER	1570.879912	3	2.5203
gi|56465319|gb|EAL43604.1| hypothetical protein 359.t00008 [Entamoeba histolytica HM-1:IMSS]	QAEYYK	801.377748	2	1.626
gi|56465344|gb|EAL43619.1| metallo-beta-lactamase superfamily protein [Entamoeba histolytica HM-1:IMSS]	LM*HNLYTPRGAKVRNGK	1971.071615	2	1.6766
	LM*HNLYTPRGAKVRNGK	1971.071615	2	1.6069
gi|56465348|gb|EAL43623.1| hypothetical protein 355.t00005 [Entamoeba histolytica HM-1:IMSS]	DYGGESNMK	1000.404055	1	2.4041
	DYGGESNMK	1000.404055	1	2.161
gi|56465387|gb|EAL43649.1| phospholipase D, putative [Entamoeba histolytica HM-1:IMSS]	KIVTSFERGKK	1292.768511	3	1.6749
gi|56465388|gb|EAL43650.1| 40S ribosomal protein S19, putative [Entamoeba histolytica HM-1:IMSS]	YILQQLEK	1034.588075	2	1.6431
gi|56465391|gb|EAL43653.1| kinase-like protein, putative [Entamoeba histolytica HM-1:IMSS]	MIGKKSSTSQSK	1281.683157	3	1.5316
	PVQKVNIDLWNVSPQR	1893.034121	3	1.7091
gi|56465421|gb|EAL43679.1| 60S ribosomal protein L26, putative [Entamoeba histolytica HM-1:IMSS]	PSNVIITK	871.524756	2	1.9262
	YNVIAIPIHK	1167.688457	3	2.624
gi|56465451|gb|EAL43706.1| hypothetical protein 344.t00003 [Entamoeba histolytica HM-1:IMSS]	M*EIIYNSDVDGFETR	1804.806922	2	1.5869
gi|56465483|gb|EAL43735.1| ubiquitin fusion degradation protein, putative [Entamoeba histolytica HM-1:IMSS]	QYLKEHMNNVCPKR	1816.873057	2	1.7868
gi|56465532|gb|EAL43776.1| hypothetical protein 337.t00004 [Entamoeba histolytica HM-1:IMSS]	DLLYLIIK	990.623397	2	1.6224
gi|56465534|gb|EAL43778.1| hypothetical protein 337.t00006 [Entamoeba histolytica HM-1:IMSS]	ISLLTTHLK	1025.635369	2	1.6427
gi|56465542|gb|EAL43785.1| hypothetical protein 336.t00004 [Entamoeba histolytica HM-1:IMSS]	LLYKFPSLNK	1222.719433	2	1.5365
gi|56465543|gb|EAL43786.1| 60S ribosomal protein L14, putative [Entamoeba histolytica HM-1:IMSS]	VLVDGPQAITGVPR	1421.811098	2	3.5354
gi|56465545|gb|EAL43788.1| conserved hypothetical protein [Entamoeba histolytica HM-1:IMSS]	KLPLGFKFLQVIQEKDFEK	2307.311123	2	1.5115
gi|56465580|gb|EAL43817.1| 60S ribosomal protein L30, putative [Entamoeba histolytica HM-1:IMSS]	LALVTKSGKYVLGTK	1577.962523	2	1.5464
gi|56465608|gb|EAL43835.1| hypothetical protein 330.t00002 [Entamoeba histolytica HM-1:IMSS]	M*PLKYFNM*VITK	1516.792417	3	1.6205
gi|56465617|gb|EAL43842.1| conserved hypothetical protein [Entamoeba histolytica HM-1:IMSS]	LDAVTDKVNLYTER	1636.854082	3	1.677
gi|56465624|gb|EAL43848.1| hypothetical protein 328.t00002 [Entamoeba histolytica HM-1:IMSS]	AISAAVENGVAR	1157.627327	2	2.399
	HVGLEDEGNFVDER	1615.734701	2	5.2103
	HVGLEDEGNFVDER	1615.734701	2	4.5285
	LELEIEAIQK	1185.672534	1	2.63
	TAIEQIVDK	1016.562255	2	2.8286
	TAIEQIVDK	1016.562255	1	2.0945
	TAIEQIVDK	1016.562255	1	1.8812
	VAAQPSVEAADK	1185.611007	2	2.8544
	VAAQPSVEAADK	1185.611007	1	2.5806
	VAQNIVK	771.472316	2	1.9557
	VAQNIVK	771.472316	1	1.531
	VQAFVAK	762.450852	1	1.8689
	VQAFVAK	762.450852	2	1.7601
gi|56465628|gb|EAL43852.1| Sec1 family protein [Entamoeba histolytica HM-1:IMSS]	PIEIFNKVKSIVGFVKK	1946.183746	2	1.7557
gi|56465661|gb|EAL43880.1| 40S ribosomal protein S24, putative [Entamoeba histolytica HM-1:IMSS]	DVQTIVLNGFVTK	1433.799862	2	2.1804
	EM*KKASR	865.457122	2	1.6322
	TSGFALIYDTLSALK	1599.862877	2	2.3017
gi|56465672|gb|EAL43889.1| hypothetical protein 324.t00001 [Entamoeba histolytica HM-1:IMSS]	ERLDNLK	887.494509	1	2.0812
gi|56465688|gb|EAL43903.1| hypothetical protein 323.t00008 [Entamoeba histolytica HM-1:IMSS]	AKIAVIEEIGNKDEQK	1784.975262	2	1.8019
gi|56465700|gb|EAL43911.1| LIM domain protein [Entamoeba histolytica HM-1:IMSS]	NQIDSILK	930.525485	1	2.1138
gi|56465724|gb|EAL43931.1| BspA-like leucine rich repeat protein, putative [Entamoeba histolytica HM-1:IMSS]	FIIPSSITK	1005.597931	2	1.5992
gi|56465738|gb|EAL43940.1| protein kinase, putative [Entamoeba histolytica HM-1:IMSS]	KAEDFIGSPLFM*SPEVIKK	2152.136979	3	2.2783
gi|56465790|gb|EAL43981.1| 60S ribosomal protein L9, putative [Entamoeba histolytica HM-1:IMSS]	HIEMYDGVVVK	1289.655839	2	3.4271
	SIQVQNFLGEK	1262.673943	2	3.64
	SIQVQNFLGEK	1262.673943	2	3.39
	SIQVQNFLGEK	1262.673943	2	3.3228
	VDIYASK	795.424708	2	1.73

	VSQSAANIHAANLVR	1550.839788	2	3.6907
	VSQSAANIHAANLVR	1550.839788	2	3.5328
	VSQSAANIHAANLVR	1550.839788	3	2.531
	VVYAHFPVTLVIADDGK	1843.995268	2	4.0086
gi|56465811|gb|EAL43996.1| RNA polymerase I largest subunit, putative [Entamoeba histolytica HM-1:IMSS]	EQIEDEENLTVEREVDK	2074.977508	2	2.056
	VSGM*CLTLLSRLLTTYLQR	2241.18903	2	2.4429
	VSGM*CLTLLSRLLTTYLQR	2241.18903	2	2.2894
gi|56465851|gb|EAL44027.1| inositol polyphosphate 5-phosphatase, putative [Entamoeba histolytica HM-1:IMSS]	PKVKFGQLVFDEK	1534.862796	3	1.5819
gi|56465884|gb|EAL44055.1| protein kinase, putative [Entamoeba histolytica HM-1:IMSS]	ITDFGTSRFIQRLNK	1795.981359	3	1.9412
gi|56465920|gb|EAL44084.1| Ribosome biogenesis protein BMS1, putative [Entamoeba histolytica HM-1:IMSS]	VCIPGLGDYR	1149.550648	3	1.5809
	VEDLTEPQDIAEDPDVDR	2055.935309	2	3.0863
gi|56465923|gb|EAL44086.1| BspA-related protein, putative [Entamoeba histolytica HM-1:IMSS]	NLRKVIMK	1001.628832	2	1.9993
gi|56465956|gb|EAL44114.1| adenylate kinase [Entamoeba histolytica HM-1:IMSS]	AVSQLEALLK	1071.640849	2	1.7598
gi|56465959|gb|EAL44117.1| Clathrin coat assembly protein, putative [Entamoeba histolytica HM-1:IMSS]	ALFSELQYGENVR	1525.76455	2	2.8226
	DELQSLITFGK	1250.66271	2	3.0982
gi|56465995|gb|EAL44144.1| hypothetical protein 292.t00013 [Entamoeba histolytica HM-1:IMSS]	VLNNGEPM*DDLKR	1516.743522	2	1.8051
gi|56465996|gb|EAL44145.1| hypothetical protein 292.t00014 [Entamoeba histolytica HM-1:IMSS]	GFESSIQLHLKSNLK	1700.933032	2	1.5887
gi|56466006|gb|EAL44152.1| Rap/Ran GTPase activating protein, putative [Entamoeba histolytica HM-1:IMSS]	TIIINKTEDYNLK	1564.858104	2	1.7811
	VGLDGNNEMK	1076.504093	2	1.8471
gi|56466015|gb|EAL44160.1| hypothetical protein 290.t00010 [Entamoeba histolytica HM-1:IMSS]	SPDGQSITFQVIEFGK	1752.880322	2	3.2496
gi|56466019|gb|EAL44164.1| Truncated, galactose-specific adhesin 170kD subunit [Entamoeba histolytica HM-1:IMSS]	FVQLTDDPSLDSEHFR	1905.897759	2	4.5911
	FVQLTDDPSLDSEHFR	1905.897759	2	4.1106
	FVQLTDDPSLDSEHFR	1905.897759	3	3.6154
	IQEIVITEK	1072.624855	2	2.6071
	LHLPQSEEFMK	1358.677311	3	2.4829
gi|56466047|gb|EAL44188.1| conserved hypothetical protein [Entamoeba histolytica HM-1:IMSS]	FDEALLEGFQSGR	1468.706704	2	3.4861
	GAIIEIDPAPFR	1298.710318	2	3.7562
	ILNVVYNASDNDFVR	1738.87589	2	2.9075
	ILNVVYNASDNDFVR	1738.87589	2	2.4271
	LENGNFSWASQSITK	1681.818063	2	2.6413
gi|56466051|gb|EAL44191.1| 40S ribosomal protein S25, putative [Entamoeba histolytica HM-1:IMSS]	ELIVPVK	797.513118	2	1.9059
	KSSGGKAPK	859.4996188	2	1.696
	LNNAVLFDK	1033.567674	2	2.4438
gi|56466056|gb|EAL44196.1| Ras family GTPase [Entamoeba histolytica HM-1:IMSS]	DIIDTKTK	933.525141	2	1.6752
gi|56466059|gb|EAL44199.1| hypothetical protein 288.t00011 [Entamoeba histolytica HM-1:IMSS]	RWIGLKNKIMM*LFK	1794.029199	2	2.6955
	RWIGLKNKIMM*LFK	1794.029199	2	2.2462
	RWIGLKNKIMM*LFK	1794.029199	2	2.2293
	RWIGLKNKIMM*LFK	1794.029199	2	1.9881
	RWIGLKNKIMM*LFK	1794.029199	2	1.9805
	RWIGLKNKIMM*LFK	1794.029199	2	1.8975
	RWIGLKNKIMM*LFK	1794.029199	2	1.8388
	RWIGLKNKIMM*LFK	1794.029199	2	1.776
	RWIGLKNKIMM*LFK	1794.029199	2	1.6449
	RWIGLKNKIMM*LFK	1794.029199	2	1.5667
gi|56466088|gb|EAL44223.1| Rab family GTPase [Entamoeba histolytica HM-1:IMSS]	HSAFFIETSALDGTNVEK	1965.955276	2	3.2401
	LVIIGESGVGK	1071.640856	2	2.8535
	LVIIGESGVGK	1071.640856	2	2.5183
gi|56466106|gb|EAL44239.1| hypothetical protein 284.t00011 [Entamoeba histolytica HM-1:IMSS]	IHIERSLK	995.599652	2	1.5145
	LFFCRIHIERSLKK	1847.025802	2	1.5117
gi|56466113|gb|EAL44245.1| hypothetical protein 283.t00003 [Entamoeba histolytica HM-1:IMSS]	LPKKTITELPK	1267.798402	2	1.5208
	PFSDINSVQLSSINTSK	1836.93384	2	2.1314

	PFSDINSVQLSSINTSK	1836.93384	2	1.9987
gi|56466117|gb|EAL44249.1| hypothetical protein 283.t00007 [Entamoeba histolytica HM-1:IMSS]	DIVSSEKIFKK	1293.741302	2	1.8447
gi|56466124|gb|EAL44255.1| dynamin-like protein [Entamoeba histolytica HM-1:IMSS]	KSIEDFVPK	1062.583	2	1.7184
	TLNEILM*QHIMKTLPSLR	2154.178453	2	1.706
gi|56466126|gb|EAL44257.1| phosphoesterase, putative [Entamoeba histolytica HM-1:IMSS]	NLITIFSIESSN	1337.694757	2	1.6729
gi|56466127|gb|EAL44258.1| Ras guanine nucleotide exchange factor, putative [Entamoeba histolytica HM-1:IMSS]	EAFDLSLK	922.488037	2	1.9262
gi|56466131|gb|EAL44262.1| beta-adaptin, putative [Entamoeba histolytica HM-1:IMSS]	LFIQQPDSQDLVQK	1658.874827	2	2.6762
gi|56466152|gb|EAL44279.1| 60S ribosomal protein L13, putative [Entamoeba histolytica HM-1:IMSS]	LTEYMNR	926.44003	2	1.8076
gi|56466155|gb|EAL44282.1| hypothetical protein 280.t00011 [Entamoeba histolytica HM-1:IMSS]	VQGAKLER	900.5261454	2	1.6494
gi|56466156|gb|EAL44283.1| SMC domain protein [Entamoeba histolytica HM-1:IMSS]	KGGIFDFSTFNINSARK	1901.986852	3	2.3093
	KGGIFDFSTFNINSARK	1901.986852	3	1.8578
gi|56466181|gb|EAL44306.1| Ras family GTPase [Entamoeba histolytica HM-1:IMSS]	EVFSVEYSPTIEDTYNK	2020.938622	2	1.6183
	SYGIDFFETSAAK	1435.674014	2	1.8767
gi|56466201|gb|EAL44324.1| 40S ribosomal protein S26, putative [Entamoeba histolytica HM-1:IMSS]	DISDNSALTK	1063.526619	2	2.482
	NIVEAAAVR	942.536708	2	3.2674
	NIVEAAAVR	942.536708	2	1.6422
	NIVEAAAVR	942.536708	1	1.5039
gi|56466247|gb|EAL44363.1| iron-sulfur flavoprotein, putative [Entamoeba histolytica HM-1:IMSS]	SKTCLQGNPLVNELIKKM*K	2217.18902	3	2.2187
gi|56466264|gb|EAL44379.1| malate dehydrogenase, putative [Entamoeba histolytica HM-1:IMSS]	EFMYQVLLK	1170.622745	2	2.0631
gi|56466282|gb|EAL44394.1| Ras guanine nucleotide exchange factor, putative [Entamoeba histolytica HM-1:IMSS]	IFDIWADGK	1064.541127	2	1.6381
gi|56466283|gb|EAL44395.1| hypothetical protein 271.t00008 [Entamoeba histolytica HM-1:IMSS]	KDVIEFSIQK	1206.672878	2	1.5516
gi|56466318|gb|EAL44424.1| hypothetical protein 268.t00001 [Entamoeba histolytica HM-1:IMSS]	NQDIPR	742.38423	1	1.582
gi|56466324|gb|EAL44430.1| receptor protein kinase, putative [Entamoeba histolytica HM-1:IMSS]	FPLKTDKWELTFNLAK	1951.068765	2	1.6283
	GREVAIKKM*K	1175.69399	2	1.6866
gi|56466401|gb|EAL44494.1| hypothetical protein 264.t00009 [Entamoeba histolytica HM-1:IMSS]	ARPM*RRHHHR	1369.725396	3	1.6097
	KCQTQLK	905.46585	2	1.504
gi|56466416|gb|EAL44505.1| peptidase, putative [Entamoeba histolytica HM-1:IMSS]	FILNVPSK	917.545491	2	1.6082
	NIDETYILK	1108.58847	1	1.8185
gi|56466421|gb|EAL44510.1| guanylate binding protein, putative [Entamoeba histolytica HM-1:IMSS]	QM*YGLDNLLSTQSLRTKLIK	2338.281018	3	1.5598
gi|56466434|gb|EAL44522.1| TPR repeat protein [Entamoeba histolytica HM-1:IMSS]	LALENEIDK	1044.55717	2	1.7398
gi|56466440|gb|EAL44527.1| phospholipase D, putative [Entamoeba histolytica HM-1:IMSS]	KQDEKIVR	1015.589472	1	1.8938
gi|56466445|gb|EAL44532.1| 60S ribosomal protein L27, putative [Entamoeba histolytica HM-1:IMSS]	AVVSSVNLQGTK	1202.673953	2	3.797
	ESWLFTK	910.466907	1	1.505
	VINVNHVMPTK	1251.687805	3	1.9991
gi|56466447|gb|EAL44534.1| calcineurin catalytic subunit, putative [Entamoeba histolytica HM-1:IMSS]	LLAVNDK	772.456332	2	1.6055
gi|56466455|gb|EAL44541.1| hypothetical protein 259.t00002 [Entamoeba histolytica HM-1:IMSS]	IM*AKMM*LTRSHQKK	1734.919769	2	1.6433
gi|56466469|gb|EAL44552.1| hypothetical protein 258.t00005 [Entamoeba histolytica HM-1:IMSS]	NLAVVDK	758.440682	1	1.6832
gi|56466487|gb|EAL44567.1| hypothetical protein 257.t00010 [Entamoeba histolytica HM-1:IMSS]	GWEEEDIQAIEIEIKSR	2045.018595	2	2.2515
	GWEEEDIQAIEIEIKSR	2045.018595	2	1.9479
	GWEEEDIQAIEIEIKSR	2045.018595	2	1.8676
gi|56466523|gb|EAL44598.1| chromodomain-helicase-DNA-binding protein, putative [Entamoeba histolytica HM-1:IMSS]	FM*NPEMNRFIFLLCTR	2104.992555	2	1.7196
gi|56466556|gb|EAL44624.1| 60S acidic ribosomal protein P0, putative [Entamoeba histolytica HM-1:IMSS]	ALDLTESDIVK	1203.646724	2	2.8138
	VGVSQAVLLQK	1141.693949	2	3.2348
gi|56466616|gb|EAL44677.1| hypothetical protein 249.t00009 [Entamoeba histolytica HM-1:IMSS]	LINAPQGAQSAEMQK	1585.800283	2	1.8766
gi|56466620|gb|EAL44681.1| tubulin alpha chain, putative [Entamoeba histolytica HM-1:IMSS]	TGYEPLLNQTMDAIR	1721.852703	2	1.6031
gi|56466623|gb|EAL44684.1| hypothetical protein 249.t00016 [Entamoeba histolytica HM-1:IMSS]	ATPKHSVLTGHTNLIR	1744.981693	2	1.5928
gi|56466629|gb|EAL44689.1| 60S ribosomal protein L7a, putative [Entamoeba histolytica HM-1:IMSS]	TNATQLFK	922.49926	2	2.5965
	TPPAVNIFANHTLDK	1637.864586	3	1.6044
gi|56466702|gb|EAL44748.1| predicted protein [Entamoeba histolytica HM-1:IMSS]	KMSEVNNLTQQLLFLKDK	2149.168566	2	1.6737
gi|56466710|gb|EAL44753.1| hypothetical protein 243.t00005 [Entamoeba histolytica HM-1:IMSS]	MEEINTDDSSNRR	1566.6813	2	1.9602
gi|56466716|gb|EAL44758.1| protein kinase, putative [Entamoeba histolytica HM-1:IMSS]	ATSSKSNENDKSNPK	1606.766765	3	1.5256

gi|56466731|gb|EAL44771.1| fatty acid elongase, putative [Entamoeba histolytica HM-1:IMSS]	AIIDGLQENLK	1213.678684	2	2.2057
	AIIDGLQENLK	1213.678684	2	2.0689
	EIGEQNEILPSLK	1469.784617	2	3.3025
	TGLGNETYLPR	1220.626986	2	2.3974
	VGNTSSSSVWYEMK	1574.715581	2	2.7426
gi|56466735|gb|EAL44775.1| hypothetical protein 242.t00006 [Entamoeba histolytica HM-1:IMSS]	ELKGDKK	817.4777984	2	1.6064
gi|56466774|gb|EAL44808.1| protein kinase, putative [Entamoeba histolytica HM-1:IMSS]	NKECIKYSDINK	1511.730804	2	1.9302
gi|56466817|gb|EAL44839.1| hypothetical protein 235.t00006 [Entamoeba histolytica HM-1:IMSS]	FYYCM*KDPQYCDWR	2047.771724	2	1.6882
gi|56466826|gb|EAL44846.1| replication factor A protein 1, putative [Entamoeba histolytica HM-1:IMSS]	DMENGVVYVLTSLKLKEFK	2213.188635	3	3.0428
gi|56466888|gb|EAL44902.1| actin-like protein 3, putative [Entamoeba histolytica HM-1:IMSS]	NIILSGGSTMFK	1267.671512	2	2.2441
gi|56466908|gb|EAL44918.1| hypothetical protein 229.t00010 [Entamoeba histolytica HM-1:IMSS]	KKLDNLLISK	1171.740897	1	1.5769
gi|56466923|gb|EAL44929.1| AIG1 family protein, putative [Entamoeba histolytica HM-1:IMSS]	EQFKEDLIKFLKEK	1795.000017	2	2.3216
gi|56466937|gb|EAL44941.1| 60S ribosomal protein L35a, putative [Entamoeba histolytica HM-1:IMSS]	NQYPNVALLQISGVNTK	1859.002155	2	4.2662
	VFLYPSNI	952.513857	1	1.6675
gi|56466941|gb|EAL44945.1| conserved hypothetical protein [Entamoeba histolytica HM-1:IMSS]	VFDLNAICFVASSSNVK	1870.915331	3	1.5708
gi|56466948|gb|EAL44950.1| hypothetical protein 226.t00004 [Entamoeba histolytica HM-1:IMSS]	IATVQYILLSCIGHK	1715.929839	3	1.9246
gi|56466973|gb|EAL44971.1| Grainin 1 [Entamoeba histolytica HM-1:IMSS]	ELQDFFVK	1025.530226	2	2.6143
	ELQDFFVK	1025.530226	2	2.3148
	ELQDFFVK	1025.530226	2	2.2643
	ELQDFFVK	1025.530226	1	2.213
	ELQDFFVK	1025.530226	1	2.1524
	ELQDFFVK	1025.530226	1	2.1488
	FPGGIK	618.3609788	2	1.7998
	LATAVFTK	850.503282	2	2.1376
	LATAVFTK	850.503282	1	1.7239
	LATAVFTK	850.503282	1	1.5143
	SGSLELDELK	1090.562672	2	1.8817
	TIYQHGFK	993.5152464	2	2.3968
	TIYQHGFK	993.5152464	1	2.0881
gi|56466988|gb|EAL44985.1| grainin 2 [Entamoeba histolytica HM-1:IMSS]	SGSLDVNELIK	1174.63142	2	3.5924
	SGSLDVNELIK	1174.63142	2	3.2679
	SGSLDVNELIK	1174.63142	2	2.5875
	SGSLDVNELIK	1174.63142	1	1.6768
	SGSLDVNELIK	1174.63142	1	1.5641
	SGTLEIGELK	1046.572835	2	2.5139
	SGTLEIGELK	1046.572835	1	1.823
	SVDVLLK	773.476743	2	2.1787
	SVDVLLK	773.476743	1	1.8081
	SVDVLLK	773.476743	2	1.7649
	SVDVLLK	773.476743	1	1.7455
gi|56466991|gb|EAL44987.1| fructose-1,6-bisphosphate aldolase, putative [Entamoeba histolytica HM-1:IMSS]	LESAIGIPEDQIR	1440.769301	2	2.4485
gi|56466997|gb|EAL44993.1| DEAD/DEAH box helicase, putative [Entamoeba histolytica HM-1:IMSS]	TGSGKTLAFLIPIVEILNK	2014.194709	3	1.9208
gi|56467001|gb|EAL44997.1| hypothetical protein 223.t00011 [Entamoeba histolytica HM-1:IMSS]	AAEFEQVTAGYVPK	1509.758392	2	3.936
	GLQLKFFFSIM*EAEM*SDIK	2266.115627	3	1.5215
	GNNEIIELDHLK	1394.727426	2	3.4936
	GNNEIIELDHLK	1394.727426	2	2.6264
	IEKDDAPFPSALDLIE	1772.895289	2	3.5955
	IEKDDAPFPSALDLIE	1772.895289	2	2.1979
	SDNLNHQSIDQSILSK	1798.893028	3	1.5531
	SLFAASMSDVK	1155.571469	2	2.7394
	SLFAASMSDVK	1155.571469	2	2.256

gi|56467003|gb|EAL44999.1| ribonuclease, putative [Entamoeba histolytica HM-1:IMSS]	YLETCNNSIIFPK	1598.766855	2	1.8834
gi|56467036|gb|EAL45028.1| hypothetical protein 221.t00014 [Entamoeba histolytica HM-1:IMSS]	GYPFM*IEGLAIM*AKKKMK	2088.107625	2	1.6296
gi|56467053|gb|EAL45042.1| protein kinase, putative [Entamoeba histolytica HM-1:IMSS]	KDIPEDQLEMIK	1458.750861	3	1.8508
gi|56467082|gb|EAL45068.1| heat shock protein 70, putative [Entamoeba histolytica HM-1:IMSS]	ETLDWIDNNQNASTEEYDNK	2399.026989	2	3.3683
	FTPEEISSMVLTK	1481.755632	2	1.6129
gi|56467084|gb|EAL45070.1| hypothetical protein 218.t00010 [Entamoeba histolytica HM-1:IMSS]	QQSNSTKAIKLIQNTIEKSDK	2374.293666	3	1.7011
gi|56467085|gb|EAL45071.1| hypothetical protein 218.t00011 [Entamoeba histolytica HM-1:IMSS]	EDGIKFVEGVNDTTK	1651.817368	3	1.8255
gi|56467087|gb|EAL45073.1| ENTH domain protein, putative [Entamoeba histolytica HM-1:IMSS]	PMVLFLGQYSTGK	1440.755566	3	2.0491
gi|56467121|gb|EAL45102.1| hypothetical protein 216.t00003 [Entamoeba histolytica HM-1:IMSS]	KQIDGIASTKNYFEGI	1783.922511	2	2.1933
gi|56467152|gb|EAL45131.1| proteasome alpha subunit, putative [Entamoeba histolytica HM-1:IMSS]	FTYGEEIPVEMLVQRICDM*K	2474.156056	3	1.5396
gi|56467165|gb|EAL45143.1| elongation factor 2, putative [Entamoeba histolytica HM-1:IMSS]	GIQYLNEVK	1063.578241	2	1.7727
gi|56467184|gb|EAL45160.1| glutaminyl-tRNA synthetase, putative [Entamoeba histolytica HM-1:IMSS]	FPPEPNGYLHIGHAK	1676.85436	2	1.8298
gi|56467243|gb|EAL45214.1| lysyl-tRNA synthetase, putative [Entamoeba histolytica HM-1:IMSS]	EIANAYTELNNPIVQR	1844.950108	2	4.2019
gi|56467244|gb|EAL45215.1| hypothetical protein 209.t00003 [Entamoeba histolytica HM-1:IMSS]	FSTEFEYGGSK	1251.552839	2	2.4592
	IMLQGNEK	932.4869824	2	2.335
	NIYFAMK	886.449137	1	1.6291
	SYDKEPYVILPNK	1565.821	3	2.7389
	VLNFVIYTPAK	1264.729988	2	3.3298
gi|56467249|gb|EAL45220.1| RNA methyltransferase, putative [Entamoeba histolytica HM-1:IMSS]	PELIVLDPPRTGIHPTALSK	2154.228132	2	1.6834
gi|56467264|gb|EAL45234.1| Rab GTPase activating protein, putative [Entamoeba histolytica HM-1:IMSS]	VLLEFGKM*NITDRDNKGR	2122.108456	3	1.8478
gi|56467277|gb|EAL45246.1| hypothetical protein 207.t00008 [Entamoeba histolytica HM-1:IMSS]	ETPTKIEVDNQEDIPIR	1997.018583	2	1.7559
	KYPMTTINTDNK	1425.704245	3	1.5999
gi|56467289|gb|EAL45256.1| cysteine proteinase 2 precursor [Entamoeba histolytica HM-1:IMSS]	LSVDGPFAAMTNEEYR	1799.826893	2	2.0086
gi|56467297|gb|EAL45264.1| protein disulfide isomerase, putative [Entamoeba histolytica HM-1:IMSS]	FGISGFPTLK	1066.593175	2	3.3122
	IAELDDLAK	987.535706	2	2.6707
	IAELDDLAK	987.535706	2	2.4545
gi|56467300|gb|EAL45267.1| hypothetical protein 206.t00016 [Entamoeba histolytica HM-1:IMSS]	MISICMYLYLQNKIIGDK	2203.110916	3	1.606
gi|56467314|gb|EAL45279.1| hypothetical protein 205.t00011 [Entamoeba histolytica HM-1:IMSS]	TNNLIVK	801.482881	2	1.577
gi|56467327|gb|EAL45291.1| hypothetical protein 204.t00004 [Entamoeba histolytica HM-1:IMSS]	M*IESEDVDLLQMKFVK	1940.971875	2	1.8559
gi|56467381|gb|EAL45343.1| WD repeat protein [Entamoeba histolytica HM-1:IMSS]	LTVERPVK	941.577844	2	1.5349
gi|56467402|gb|EAL45363.1| DEAD/DEAH box helicase, putative [Entamoeba histolytica HM-1:IMSS]	M*RDLIALAETGTGK	1491.784661	2	1.776
gi|56467422|gb|EAL45382.1| hypothetical protein 200.t00010 [Entamoeba histolytica HM-1:IMSS]	QLNDIKEVTK	1187.663032	2	1.6956
gi|56467423|gb|EAL45383.1| hypothetical protein 200.t00011 [Entamoeba histolytica HM-1:IMSS]	PKTFALSLNK	1118.656833	1	1.5133
gi|56467427|gb|EAL45387.1| hypothetical protein 200.t00015 [Entamoeba histolytica HM-1:IMSS]	NQSLVLNLFGRQLDSLTQPEK	2400.288178	3	2.061
gi|56467447|gb|EAL45406.1| hypothetical protein 199.t00015 [Entamoeba histolytica HM-1:IMSS]	HYGNLIDK	959.4945114	2	1.5156
	TLKRLGHGIAK	1193.747708	3	1.6025
gi|56467465|gb|EAL45423.1| hypothetical protein 198.t00013 [Entamoeba histolytica HM-1:IMSS]	INAVRTSSDNTVDSVDRNIR	2232.132749	3	1.8612
gi|56467488|gb|EAL45445.1| Rho family GTPase [Entamoeba histolytica HM-1:IMSS]	LVFDEAVR	948.51491	2	1.8492
	PINLGLWDTAGQEDYDR	1962.919208	2	4.7367
	PINLGLWDTAGQEDYDR	1962.919208	2	3.2335
gi|56467489|gb|EAL45446.1| DNA repair helicase, putative [Entamoeba histolytica HM-1:IMSS]	RFTDISADQSITLAKEFLM*K	2330.207183	3	2.5817
gi|56467491|gb|EAL45448.1| ankyrin repeat protein [Entamoeba histolytica HM-1:IMSS]	ATPLFIAAQYKNSK	1551.852968	2	1.5589
gi|56467515|gb|EAL45468.1| actin related protein, putative [Entamoeba histolytica HM-1:IMSS]	KADNLEVILLR	1283.768165	3	2.7317
gi|56467521|gb|EAL45474.1| 60S ribosomal protein L44, putative [Entamoeba histolytica HM-1:IMSS]	HFELGNEK	973.4737764	2	2.0653
gi|56467522|gb|EAL45475.1| 60S ribosomal protein L24, putative [Entamoeba histolytica HM-1:IMSS]	QTQSSEVLAK	1090.573903	2	1.8674
	VFWTAIYR	1055.567279	2	3.1232
gi|56467543|gb|EAL45494.1| hypothetical protein 194.t00014 [Entamoeba histolytica HM-1:IMSS]	DIIQVSTADFDK	1351.674002	2	2.7618
gi|56467570|gb|EAL45517.1| conserved hypothetical protein [Entamoeba histolytica HM-1:IMSS]	M*EELISK	865.434656	2	2.5871
	M*EELISK	865.434656	1	2.1258
gi|56467575|gb|EAL45521.1| WD repeat protein [Entamoeba histolytica HM-1:IMSS]	VDFDNSIFM*KYKGIK	1820.926247	2	1.8478
gi|56467595|gb|EAL45540.1| coactosin, putative [Entamoeba histolytica HM-1:IMSS]	EDALQFAYYR	1275.600432	2	2.6538

gi|56467606|gb|EAL45551.1| cysteine proteinase, putative [Entamoeba histolytica HM-1:IMSS]	AAAEGPVAAAIDASGVK	1497.790768	2	2.9298
gi|56467641|gb|EAL45583.1| myotubularin, putative [Entamoeba histolytica HM-1:IMSS]	NLIVPVK	782.513452	1	2.2258
gi|56467644|gb|EAL45586.1| D-hydantoinase, putative [Entamoeba histolytica HM-1:IMSS]	EDAIKYVCSPPM*K	1553.713474	2	1.662
gi|56467670|gb|EAL45607.1| HMG box protein [Entamoeba histolytica HM-1:IMSS]	KDEIKKEDEK	1261.663427	2	1.7519
gi|56467678|gb|EAL45615.1| hypothetical protein 188.t00027 [Entamoeba histolytica HM-1:IMSS]	GINIYDGIKGEKER	1591.843858	3	1.6954
gi|56467717|gb|EAL45649.1| Rab family GTPase [Entamoeba histolytica HM-1:IMSS] gi|56790064|dbj|BAD82828.1| EhRabC	LQIWDTAGQER	1316.659346	2	2.1643
gi|56467718|gb|EAL45650.1| hypothetical protein 186.t00016 [Entamoeba histolytica HM-1:IMSS]	NSILM*TITGLAGNNLNR	1817.954925	3	2.0758
gi|56467721|gb|EAL45653.1| conserved hypothetical protein [Entamoeba histolytica HM-1:IMSS]	MVDLETKVK	1062.58636	1	1.9515
gi|56467774|gb|EAL45701.1| acyl-CoA synthetase, putative [Entamoeba histolytica HM-1:IMSS]	EVLEMTDSLASGIMNK	1737.839775	2	2.1689
gi|56467780|gb|EAL45707.1| conserved hypothetical protein [Entamoeba histolytica HM-1:IMSS]	ESNNSVAK	848.41086	2	1.6819
gi|56467795|gb|EAL45720.1| hypothetical protein 182.t00029 [Entamoeba histolytica HM-1:IMSS]	KVSSEILKGFEDK	1479.805362	2	1.7129
gi|56467813|gb|EAL45737.1| hypothetical protein 182.t00023 [Entamoeba histolytica HM-1:IMSS]	EIEQETIIR	1130.605183	2	1.5574
gi|56467856|gb|EAL45771.1| hypothetical protein 180.t00018 [Entamoeba histolytica HM-1:IMSS]	EIISNIK	816.482557	2	1.5128
gi|56467863|gb|EAL45776.1| 60S ribosomal protein L31, putative [Entamoeba histolytica HM-1:IMSS]	FYTLVSYVPVASFK	1620.867231	2	3.9689
gi|56467864|gb|EAL45777.1| Ras family GTPase [Entamoeba histolytica HM-1:IMSS]	DTENVPIIVVGNK	1397.763477	2	3.0846
	DTENVPIIVVGNK	1397.763477	2	3.033
gi|56467886|gb|EAL45796.1| conserved hypothetical protein [Entamoeba histolytica HM-1:IMSS]	LTRTVFDNPNTPHSNCR	2028.945383	2	1.554
gi|56467907|gb|EAL45816.1| Rab family GTPase [Entamoeba histolytica HM-1:IMSS] gi|50978610|dbj|BAD34972.1| EhRab7I	MILIGDSGVGK	1089.597276	2	1.883
gi|56467953|gb|EAL45854.1| hypothetical protein 175.t00013 [Entamoeba histolytica HM-1:IMSS]	DIDEMIKMKINK	1477.7753	2	2.0173
gi|56467989|gb|EAL45884.1| FEN-1 nuclease, putative [Entamoeba histolytica HM-1:IMSS]	RKKPTTK	858.551963	2	1.5424
gi|56467994|gb|EAL45889.1| hypothetical protein 173.t00014 [Entamoeba histolytica HM-1:IMSS]	LDLSANAEFGKNNK	1520.770364	2	2.6045
	LDLSANAEFGKNNK	1520.770364	2	1.9403
gi|56468005|gb|EAL45900.1| 60S ribosomal protein L38, putative [Entamoeba histolytica HM-1:IMSS]	DIHVVEIAK	1023.583324	2	2.8663
	DIHVVEIAK	1023.583324	2	2.7476
	DIHVVEIAK	1023.583324	1	2.7096
	DIHVVEIAK	1023.583324	3	2.6347
	DIHVVEIAK	1023.583324	1	2.4612
	DIHVVEIAK	1023.583324	1	2.4529
gi|56468007|gb|EAL45901.1| Plasma membrane calcium-transporting ATPase, putative [Entamoeba histolytica HM-1:IMSS]	RNENNIYQMFAFSSAK	1919.906882	2	1.5081
gi|56468009|gb|EAL45902.1| conserved hypothetical protein [Entamoeba histolytica HM-1:IMSS]	YNEGKKGMANAESMLKK	1898.946298	2	1.8152
gi|56468029|gb|EAL45920.1| conserved hypothetical protein [Entamoeba histolytica HM-1:IMSS]	NVVIVQSK	886.535655	2	1.9808
gi|56468029|gb|EAL45920.1| conserved hypothetical protein [Entamoeba histolytica HM-1:IMSS]	STEEVEDELNK	1292.585248	3	1.7478
gi|56468058|gb|EAL45948.1| Rab family GTPase [Entamoeba histolytica HM-1:IMSS]	AVTVEQGGQELANSLNVSFMEASAK	2523.239584	2	2.0517
gi|56468106|gb|EAL45992.1| galactose-inhibitable lectin 35 kda subunit precursor, putative [Entamoeba histolytica HM-1:IMSS	VIFASDYNYK	1219.599379	2	2.8949
	VIFASDYNYK	1219.599379	1	2.2699
gi|56468159|gb|EAL46040.1| hypothetical protein 166.t00004 [Entamoeba histolytica HM-1:IMSS]	INTSTFNNEISFLASVQK	2013.028783	2	2.024
gi|56468201|gb|EAL46076.1| hypothetical protein 164.t00002 [Entamoeba histolytica HM-1:IMSS]	KLKTNTLVATQLLIK	1684.073121	2	2.0217
gi|56468203|gb|EAL46078.1| hypothetical protein 164.t00004 [Entamoeba histolytica HM-1:IMSS]	LVIDLNTFK	1062.619375	1	1.7138
gi|56468231|gb|EAL46102.1| hypothetical protein 162.t00002 [Entamoeba histolytica HM-1:IMSS]	PVCHLNAIDVSGAKLK	1721.915252	3	1.5907
	RLMKIIQR	1057.66628	2	1.6668
gi|56468240|gb|EAL46111.1| hypothetical protein 162.t00011 [Entamoeba histolytica HM-1:IMSS]	AVDEDDEIK	1033.468416	2	1.6297
gi|56468247|gb|EAL46118.1| predicted protein [Entamoeba histolytica HM-1:IMSS]	LYLLKKEYLSDNQINDIR	2238.212875	3	2.0333
gi|56468265|gb|EAL46133.1| hypothetical protein 161.t00005 [Entamoeba histolytica HM-1:IMSS]	M*YFSLIQPFSNK	1490.735934	2	1.9077
gi|56468269|gb|EAL46136.1| hypothetical protein 160.t00020 [Entamoeba histolytica HM-1:IMSS]	SQLLISKENKTFIFNGEFNK	2357.250001	3	1.9593
gi|56468284|gb|EAL46149.1| protein kinase, putative [Entamoeba histolytica HM-1:IMSS]	ALTYLHSFHCVHR	1640.789989	2	1.6535
gi|56468289|gb|EAL46152.1| methionine aminopeptidase, putative [Entamoeba histolytica HM-1:IMSS]	ALIDIEDYR	1107.568069	2	3.3963
	ALIDIEDYR	1107.568069	2	3.0588
	LLHALDANFK	1141.636422	3	3.8466
	LLHALDANFK	1141.636422	3	3.7509
	LLHALDANFK	1141.636422	2	2.7527
	PGMSDLFVAENIER	1577.762835	2	4.2314

	PGMSDLFVAENIER	1577.762835	2	3.6027
gi|56468328|gb|EAL46188.1| hypothetical protein 158.t00019 [Entamoeba histolytica HM-1:IMSS]	IFDGKVNPQVFNVK	1604.879509	3	1.7367
gi|56468368|gb|EAL46222.1| profilin, putative [Entamoeba histolytica HM-1:IMSS]	GAEGISIYK	937.4989408	1	1.8294
	YLMDAGY	832.3545714	1	1.7684
gi|56468426|gb|EAL46272.1| hypothetical protein 153.t00006 [Entamoeba histolytica HM-1:IMSS]	GIFINYVNK	1067.588411	2	2.1184
	TIVLNFEK	963.550961	1	1.7595
gi|56468427|gb|EAL46273.1| histone H3, putative [Entamoeba histolytica HM-1:IMSS]	AHPGAVALTEIK	1206.684103	3	2.8808
	AHPGAVALTEIK	1206.684103	1	2.8245
	AHPGAVALTEIK	1206.684103	2	2.0451
	APFQALVR	901.525414	2	2.3434
	ITIMPK	702.421859	1	1.9176
gi|56468432|gb|EAL46278.1| protein kinase, putative [Entamoeba histolytica HM-1:IMSS]	HYQLIDETHLEMDEFKR	2204.044085	2	1.5044
gi|56468458|gb|EAL46302.1| actophorin, putative [Entamoeba histolytica HM-1:IMSS]	MNDGMTEVVVEK	1351.623219	2	3.7367
	NATYDDFLK	1086.51022	2	2.0917
	NATYDDFLK	1086.51022	2	2.076
	NATYDDFLK	1086.51022	1	1.6611
	YAVYDLEYDTPEGLR	1803.84358	2	4.3655
	YAVYDLEYDTPEGLR	1803.84358	2	4.1606
	YAVYDLEYDTPEGLR	1803.84358	2	3.9586
	YAVYDLEYDTPEGLR	1803.84358	2	3.8015
gi|56468474|gb|EAL46316.1| multidrug resistance protein, putative [Entamoeba histolytica HM-1:IMSS]	FTSILSEK	924.503697	2	1.7591
gi|56468485|gb|EAL46327.1| hypothetical protein 151.t00017 [Entamoeba histolytica HM-1:IMSS]	QNKENKK	888.489758	2	1.9059
gi|56468489|gb|EAL46331.1| acid sphingomyelinase-like phosphodiesterase, putative [Entamoeba histolytica HM-1:IMSS]	VKLVYK	749.491988	1	1.6262
gi|56468511|gb|EAL46352.1| 60S ribosomal protein L31, putative [Entamoeba histolytica HM-1:IMSS]	LPIEGEEGK	971.5044108	2	2.0471
gi|56468526|gb|EAL46365.1| hypothetical protein 149.t00003 [Entamoeba histolytica HM-1:IMSS]	IMSSIDENSINPSIER	1804.8746	2	2.1554
gi|56468543|gb|EAL46380.1| protein phosphatase, putative [Entamoeba histolytica HM-1:IMSS]	FIVRSHEVKK	1242.731729	2	1.6684
	NGSSKSPRLIGRTFGPDILLR	2284.288466	2	1.9556
	NGSSKSPRLIGRTFGPDILLR	2284.288466	2	1.766
gi|56468561|gb|EAL46395.1| DNA-directed RNA polymerase II large subunit, putative [Entamoeba histolytica HM-1:IMSS]	TIPYTKLK	963.587346	2	1.6514
gi|56468580|gb|EAL46413.1| Rho family GTPase [Entamoeba histolytica HM-1:IMSS]	INLGLWDTAGQEDYDR	1865.866444	2	4.4831
	INLGLWDTAGQEDYDR	1865.866444	2	3.8012
	INLGLWDTAGQEDYDR	1865.866444	2	3.4313
	INLGLWDTAGQEDYDR	1865.866444	2	2.5857
	LVIVGDGAVGK	1027.614631	2	2.1292
	NIVPIQPPQGDELAK	1618.879904	2	2.415
gi|56468630|gb|EAL46461.1| hypothetical protein 144.t00010 [Entamoeba histolytica HM-1:IMSS]	AIMNIISLEK	1131.644219	2	3.5201
	AIMNIISLEK	1131.644219	2	3.4555
	AIMNIISLEK	1131.644219	1	2.2488
	AIMNIISLEK	1131.644219	1	2.2298
	INKEEIIR	1014.594223	2	2.943
	INKEEIIR	1014.594223	3	2.5741
	LVPIEQYDK	1104.593555	2	1.8737
	LVPIEQYDK	1104.593555	1	1.5854
	QQPVDDMDIEGSDDK	1691.706505	2	2.5902
	QQPVDDMDIEGSDDKK	1819.801468	2	2.5174
gi|56468631|gb|EAL46462.1| hypothetical protein 144.t00011 [Entamoeba histolytica HM-1:IMSS]	LQRPQAISTIKETIKR	1882.12327	2	2.2954
	LQRPQAISTIKETIKR	1882.12327	2	1.6641
	QRELLSHFGK	1214.664046	3	1.8412
gi|56468634|gb|EAL46465.1| conserved hypothetical protein [Entamoeba histolytica HM-1:IMSS]	NVLKTDISM*K	1164.630396	2	2.0153
	NVLKTDISM*K	1164.630396	2	1.8725
gi|56468643|gb|EAL46474.1| hypothetical protein 144.t00023 [Entamoeba histolytica HM-1:IMSS]	LENKTYIER	1165.621167	2	2.1908

gi|56468653|gb|EAL46482.1| hypothetical protein 143.t00005 [Entamoeba histolytica HM-1:IMSS]	MQTSNTTNDIDTIITTM*KR	2200.059523	3	1.5206
gi|56468654|gb|EAL46483.1| translation elongation factor EF-1 alpha, putative [Entamoeba histolytica HM-1:IMSS]	ISGIGTVPVGR	1055.620789	2	2.6938
	ISGIGTVPVGR	1055.620789	2	2.1753
	ISGIGTVPVGR	1055.620789	1	1.7001
	SIEMHHTALAQAIPGDNVGFNVR	2477.235423	2	4.9361
	SIEMHHTALAQAIPGDNVGFNVR	2477.235423	2	4.2403
	SIEMHHTALAQAIPGDNVGFNVR	2477.235423	3	2.1423
gi|56468721|gb|EAL46543.1| conserved hypothetical protein [Entamoeba histolytica HM-1:IMSS]	LKSMNGSSK	951.4928264	2	1.6252
gi|56468758|gb|EAL46575.1| conserved hypothetical protein [Entamoeba histolytica HM-1:IMSS]	DENEEKM*K	1038.441918	2	1.5186
gi|56468760|gb|EAL46577.1| hypothetical protein 138.t00014 [Entamoeba histolytica HM-1:IMSS]	AKFLNTLHVMMLLLRNQE	2171.182764	2	2.011
gi|56468761|gb|EAL46578.1| Rho guanine nucleotide exchange factor, putative [Entamoeba histolytica HM-1:IMSS]	KPPLPPNK	890.545815	2	1.6048
gi|56468763|gb|EAL46580.1| UDP-N-acetylglucosamine pyrophosphorylase, putative [Entamoeba histolytica HM-1:IMSS]	RDWYRLNIYYLKK	1831.001353	2	1.5465
gi|56468770|gb|EAL46586.1| hypothetical protein 138.t00006 [Entamoeba histolytica HM-1:IMSS]	KVLIFLNEIPK	1313.819137	2	1.7524
gi|56468772|gb|EAL46588.1| hypothetical protein 138.t00008 [Entamoeba histolytica HM-1:IMSS]	AINAVGGVAK	899.5308988	2	2.6137
	GVVGIFNK	833.4879708	1	1.566
gi|56468781|gb|EAL46594.1| 40S ribosomal protein S24, putative [Entamoeba histolytica HM-1:IMSS]	QVVVDIYHPGIVQPK	1691.947923	2	4.0958
	QVVVDIYHPGIVQPK	1691.947923	2	3.2191
gi|56468832|gb|EAL46642.1| cytosolic regulator pianissimo, putative [Entamoeba histolytica HM-1:IMSS]	LVDEDIIK	944.529892	2	1.6331
gi|56468869|gb|EAL46676.1| apyrase, putative [Entamoeba histolytica HM-1:IMSS]	YKDPQYVNERCFSSIK	2033.953498	3	1.8094
gi|56468877|gb|EAL46684.1| 60S ribosomal protein L17, putative [Entamoeba histolytica HM-1:IMSS]	AQEFLK	735.403568	2	1.6879
	NTYETADAIR	1153.548397	2	2.8591
	SAQFILK	806.477077	1	2.2042
gi|56468890|gb|EAL46696.1| phosphatidylinositol-3,4,5-trisphosphate 3-phosphatase, putative [Entamoeba histolytica HM-1:IM	RSKIM*K	778.461478	2	1.6194
gi|56468896|gb|EAL46702.1| hypothetical protein 133.t00008 [Entamoeba histolytica HM-1:IMSS]	IEVTEEK	847.440743	2	2.1084
	IVGVVEK	743.4661704	1	2.2117
gi|56468901|gb|EAL46705.1| hypothetical membrane-spanning protein [Entamoeba histolytica HM-1:IMSS]	SMPVFLQR	977.523709	2	1.5803
gi|56468917|gb|EAL46720.1| hypothetical protein 132.t00009 [Entamoeba histolytica HM-1:IMSS]	EERNENNILSQPKKIR	1968.062128	2	1.7832
gi|56468967|gb|EAL46765.1| Beige/BEACH domain protein, putative [Entamoeba histolytica HM-1:IMSS]	YLM*KYEGIEIFFM*NGK	2014.967488	3	2.0849
gi|56468969|gb|EAL46767.1| cullin, putative [Entamoeba histolytica HM-1:IMSS]	IVSGKIK	744.4978144	2	1.6151
gi|56468970|gb|EAL46768.1| importin beta subunit, putative [Entamoeba histolytica HM-1:IMSS]	YKEAAIFVFGCIICK	1818.885186	2	2.203
gi|56469007|gb|EAL46803.1| protein kinase, putative [Entamoeba histolytica HM-1:IMSS]	DKVKVIIETPNNILK	1724.031651	2	2.018
gi|56469018|gb|EAL46813.1| Rho guanine nucleotide exchange factor, putative [Entamoeba histolytica HM-1:IMSS]	KQAFFLATDISPTEFIEK	2085.0903	2	2.2221
gi|56469020|gb|EAL46815.1| mRNA capping enzyme, putative [Entamoeba histolytica HM-1:IMSS]	M*LETIKYNTQSNSSIEVESR	2345.130077	3	1.7918
	VTVSCNVTFDMFDDSKAFK	2210.988229	2	1.6054
gi|56469021|gb|EAL46816.1| hypothetical protein 128.t00010 [Entamoeba histolytica HM-1:IMSS]	VENVAGVASK	973.5313014	1	1.9925
	VENVAGVASK	973.5313014	2	1.8757
gi|56469022|gb|EAL46817.1| hypothetical protein 128.t00011 [Entamoeba histolytica HM-1:IMSS]	LIINSILPNIK	1237.787847	2	1.6109
gi|56469053|gb|EAL46846.1| hypothetical protein 127.t00023 [Entamoeba histolytica HM-1:IMSS]	LESAKIK	788.487642	1	1.7431
gi|56469066|gb|EAL46857.1| conserved hypothetical protein [Entamoeba histolytica HM-1:IMSS]	KNM*SNPK	834.414923	1	1.5508
gi|56469096|gb|EAL46884.1| hypothetical protein 125.t00011 [Entamoeba histolytica HM-1:IMSS]	DNIDRIGFCLFITTQIEK	2183.095059	2	1.5675
gi|56469098|gb|EAL46886.1| protein kinase, putative [Entamoeba histolytica HM-1:IMSS]	YVPFAYDPDALK	1398.693998	2	1.6229
gi|56469110|gb|EAL46896.1| hypothetical protein 124.t00008 [Entamoeba histolytica HM-1:IMSS]	GEDDYNKFDELTK	1573.701667	2	1.8423
gi|56469162|gb|EAL46942.1| 60S ribosomal protein L34, putative [Entamoeba histolytica HM-1:IMSS]	AFLINEHNLIK	1311.74195	3	2.1197
	AFLINEHNLIK	1311.74195	3	1.9338
gi|56469191|gb|EAL46966.1| Rab family GTPase [Entamoeba histolytica HM-1:IMSS] gi|56790054|dbj|BAD82823.1| EhRabC	MQLWDTAGQDR	1320.600116	2	2.4161
	NITASYYR	987.489434	2	1.8527
gi|56469198|gb|EAL46973.1| UMP-CMP kinase, putative [Entamoeba histolytica HM-1:IMSS]	IDSSLAKFFADNHILL	1803.963986	2	1.836
gi|56469204|gb|EAL46979.1| hypothetical protein 121.t00023 [Entamoeba histolytica HM-1:IMSS]	LSNQVIDIKKATDK	1572.895562	2	1.8902
gi|56469215|gb|EAL46985.1| elongation factor-1alpha, putative [Entamoeba histolytica HM-1:IMSS]	RHSEIINKVKEM*HK	1764.954859	3	2.2038
gi|56469245|gb|EAL47008.1| Gal/GalNAc lectin subunit Igl2 [Entamoeba histolytica HM-1:IMSS]	DLYLPYYFSVTK	1508.767173	2	3.3042
	DLYLPYYFSVTK	1508.767173	2	1.6569

	GYDQYEQYLNAFK	1638.743471	2	4.2597
	GYDQYEQYLNAFK	1638.743471	2	4.2338
	LFSPTEENK	1064.52588	2	1.9145
	SAFSYYDGSDSNSPK	1624.676231	2	1.8727
	SAFSYYDGSDSNSPK	1624.676231	2	1.8549
gi|56469308|gb|EAL47068.1| Ras GTPase activating protein, putative [Entamoeba histolytica HM-1:IMSS]	IVDLYIKLGK	1161.724176	2	2.1228
gi|56469323|gb|EAL47082.1| histone H2A, putative [Entamoeba histolytica HM-1:IMSS]	DVTISYGGVFPNVPTAVNSK	2065.060078	2	4.821
	DVTISYGGVFPNVPTAVNSK	2065.060078	2	4.4791
	DVTISYGGVFPNVPTAVNSK	2065.060078	2	3.6491
	DVTISYGGVFPNVPTAVNSK	2065.060078	2	2.8024
	DVTISYGGVFPNVPTAVNSK	2065.060078	2	2.7378
	DVTISYGGVFPNVPTAVNSK	2065.060078	2	1.6486
	DVTISYGGVFPNVPTAVNSK	2065.060078	2	1.576
	HIQLAVR	836.510098	2	2.3094
	HIQLAVR	836.510098	2	2.2933
	SDLELNDLLK	1159.620509	2	3.7495
	SDLELNDLLK	1159.620509	2	3.2739
	SDLELNDLLK	1159.620509	2	3.0196
	SDLELNDLLK	1159.620509	1	2.6792
	SDLELNDLLK	1159.620509	2	2.3476
	SDLELNDLLK	1159.620509	1	2.2782
	SGLQFPVAR	974.5418054	2	2.9109
	SGLQFPVAR	974.5418054	2	2.6802
	SGLQFPVAR	974.5418054	1	2.2594
	SGLQFPVAR	974.5418054	1	2.0884
gi|56469328|gb|EAL47087.1| 60S ribosomal protein L3, putative [Entamoeba histolytica HM-1:IMSS]	DNAAEKPHLTGFLGFK	1744.901705	3	3.043
	NDFLMIK	880.459702	2	2.245
	NDFLMIK	880.459702	1	1.9272
gi|56469342|gb|EAL47100.1| 60S ribosomal protein L9, putative [Entamoeba histolytica HM-1:IMSS]	KTEHVVIPEGVNIEINR	1947.065808	2	2.517
gi|56469351|gb|EAL47108.1| hypothetical protein 116.t00011 [Entamoeba histolytica HM-1:IMSS]	GLYITVEFEFDGSR	1632.790434	2	1.5055
gi|56469392|gb|EAL47146.1| DNA mismatch repair protein mutS, putative [Entamoeba histolytica HM-1:IMSS]	QFHM*EVALNDHGLCPK	1911.863653	2	1.5323
gi|56469399|gb|EAL47152.1| 60S ribosomal protein L10, putative [Entamoeba histolytica HM-1:IMSS]	LFDIGNR	834.4468324	2	1.956
	VGQVLISGR	928.5574578	2	3.3037
gi|56469415|gb|EAL47166.1| hypothetical protein 114.t00029 [Entamoeba histolytica HM-1:IMSS]	IPPSDSSRIILSETVAMK	1944.047084	2	1.7118
gi|56469459|gb|EAL47206.1| hypothetical protein 113.t00008 [Entamoeba histolytica HM-1:IMSS]	HCM*PKVKTIKINNEEIEVM*K	2473.241884	3	1.7046
gi|56469495|gb|EAL47239.1| V-type ATPase, A subunit, putative [Entamoeba histolytica HM-1:IMSS]	LAEMPADSGYPAYLAAR	1795.868363	2	1.5422
gi|56469511|gb|EAL47254.1| hypothetical protein 111.t00011 [Entamoeba histolytica HM-1:IMSS]	KIDLQLIDIILPFK	1669.029859	3	2.0052
gi|56469519|gb|EAL47261.1| hypothetical protein 110.t00018 [Entamoeba histolytica HM-1:IMSS]	ELLTKFTGNKGIVDFENNK	2167.139371	2	1.8117
gi|56469523|gb|EAL47265.1| hypothetical protein 110.t00022 [Entamoeba histolytica HM-1:IMSS]	KSNIENELTKKQEEGK	1874.981815	3	1.5889
gi|56469533|gb|EAL47274.1| Rho family GTPase [Entamoeba histolytica HM-1:IMSS]	KATNKHKCSLL	1299.698714	2	1.9341
gi|56469536|gb|EAL47277.1| hypothetical protein 110.t00009 [Entamoeba histolytica HM-1:IMSS]	ENLHQRIK	1037.585055	2	1.8322
gi|56469544|gb|EAL47284.1| protein phosphatase 2C, putative [Entamoeba histolytica HM-1:IMSS]	KITTANIGDTEIMFCKR	1997.993235	2	1.5046
gi|56469555|gb|EAL47295.1| hypothetical protein 109.t00016 [Entamoeba histolytica HM-1:IMSS]	VLQTLHPNQYPK	1437.784878	3	1.7986
gi|56469580|gb|EAL47315.1| Sec7 domain protein [Entamoeba histolytica HM-1:IMSS]	PQILLLKSQITEAK	1581.957433	3	1.8763
gi|56469582|gb|EAL47317.1| hypothetical protein 108.t00008 [Entamoeba histolytica HM-1:IMSS]	LGEVAMR	775.4130884	2	1.9005
gi|56469601|gb|EAL47333.1| hypothetical protein 107.t00003 [Entamoeba histolytica HM-1:IMSS]	SPKIMCTNEGGVFFVTGANK	2157.025279	2	1.5062
gi|56469609|gb|EAL47341.1| actin, putative [Entamoeba histolytica HM-1:IMSS]	ETGLVVEIGNR	1186.642636	2	1.664
gi|56469614|gb|EAL47346.1| 60S ribosomal protein L12, putative [Entamoeba histolytica HM-1:IMSS]	ITVVNR	701.430451	2	1.5896
gi|56469615|gb|EAL47347.1| SNF7 family protein [Entamoeba histolytica HM-1:IMSS]	KGDKENAK	889.4737764	2	1.5254
gi|56469645|gb|EAL47374.1| 60S ribosomal protein L4, putative [Entamoeba histolytica HM-1:IMSS]	INAYSDVLK	1022.5517	2	2.1609
	LNTIFGTQK	1021.567676	2	2.5497

	LPAVFLAPIR	1096.687728	2	3.0386
	LPAVFLAPIR	1096.687728	2	2.7757
gi|56469660|gb|EAL47388.1| protein kinase, putative [Entamoeba histolytica HM-1:IMSS]	ERIQIAISIAK	1241.75761	2	1.5062
gi|56469662|gb|EAL47390.1| Rab family GTPase [Entamoeba histolytica HM-1:IMSS] gi|13537447|dbj|BAB40678.1| small GT	GALGALVVYDITK	1319.756937	2	2.591
gi|56469673|gb|EAL47400.1| hypothetical protein 105.t00002 [Entamoeba histolytica HM-1:IMSS]	LATSVGIK	788.4876444	2	1.94
gi|56469699|gb|EAL47424.1| hypothetical protein 104.t00017 [Entamoeba histolytica HM-1:IMSS]	TEM*EQVNR	1022.458236	2	1.5195
gi|56469738|gb|EAL47459.1| conserved hypothetical protein [Entamoeba histolytica HM-1:IMSS]	GIFSEDMIK	1039.512873	1	1.7899
	NVAVTLIK	857.545481	2	1.5962
	NVAVTLIK	857.545481	1	1.5449
	VVLYGVNQMLR	1291.719107	2	3.5896
	WTEFDQAAEDAIIAK	1707.822448	2	4.1925
	WTEFDQAAEDAIIAK	1707.822448	2	4.0601
	WTEFDQAAEDAIIAK	1707.822448	2	3.425
	WTEFDQAAEDAIIAK	1707.822448	2	3.2109
gi|56469758|gb|EAL47478.1| hypothetical protein 103.t00013 [Entamoeba histolytica HM-1:IMSS]	TPKIVKAIMPK	1225.770077	2	2.1601
gi|56469802|gb|EAL47515.1| conserved hypothetical protein [Entamoeba histolytica HM-1:IMSS]	M*ALGCCGYNEDITHCLSTR	2273.877344	3	1.5873
gi|56469819|gb|EAL47528.1| RNA-binding protein, putative [Entamoeba histolytica HM-1:IMSS]	NDAALAM*K	849.414579	2	1.6686
gi|56469824|gb|EAL47533.1| acyl-CoA synthetase, putative [Entamoeba histolytica HM-1:IMSS]	KIMAENEIGLSQREVVK	1944.058289	2	1.551
gi|56469825|gb|EAL47534.1| hypothetical protein 100.t00015 [Entamoeba histolytica HM-1:IMSS]	KVQSIAKK	901.582939	2	1.5104
gi|56469852|gb|EAL47560.1| 14-3-3 protein 1 [Entamoeba histolytica HM-1:IMSS]	HVEIIK	738.450852	2	1.5488
gi|56469856|gb|EAL47564.1| inositol polyphosphate kinase, putative [Entamoeba histolytica HM-1:IMSS]	RLIVDSTSTTTSLGVRFSGMRR	2440.308952	2	1.6751
gi|56469859|gb|EAL47567.1| long-chain-fatty-acid--CoA ligase, putative [Entamoeba histolytica HM-1:IMSS]	EPALTIK	771.461083	2	1.586
gi|56469863|gb|EAL47571.1| hypothetical protein 99.t00030 [Entamoeba histolytica HM-1:IMSS]	MIKELTYSINK	1339.729012	2	1.5589
gi|56469884|gb|EAL47588.1| hypothetical protein 98.t00013 [Entamoeba histolytica HM-1:IMSS]	KDGFVYVKNDENHFVFTLR	2328.17715	3	1.7147
gi|56469893|gb|EAL47597.1| inositol polyphosphate kinase, putative [Entamoeba histolytica HM-1:IMSS]	M*IDFAHWKDK	1306.625969	3	2.206
gi|56469894|gb|EAL47598.1| pyruvate:ferredoxin oxidoreductase [Entamoeba histolytica HM-1:IMSS]	HFLEQLPK	1011.562194	2	2.2291
	VPAEWANAPLETVTK	1625.853353	2	3.5058
gi|56469896|gb|EAL47600.1| BspA-like leucine rich repeat protein, putative [Entamoeba histolytica HM-1:IMSS]	LSKINLVTSLKEIGTR	1772.064036	2	1.5387
gi|56469897|gb|EAL47601.1| hypothetical protein 98.t00026 [Entamoeba histolytica HM-1:IMSS]	ESLSPISHRK	1153.63243	2	1.9233
gi|56469913|gb|EAL47616.1| hypothetical protein 97.t00026 [Entamoeba histolytica HM-1:IMSS]	SIYLSFHQLPK	1332.731071	3	1.5623
gi|56469922|gb|EAL47624.1| 60S ribosomal protein L2/L8, putative [Entamoeba histolytica HM-1:IMSS]	AMVGIIAGGGR	1001.556072	2	2.591
gi|56469954|gb|EAL47650.1| hypothetical protein 96.t00007 [Entamoeba histolytica HM-1:IMSS]	SFPAVVEGNTLAGDSTK	1692.843937	2	2.4881
gi|56469969|gb|EAL47664.1| hypothetical protein 95.t00026 [Entamoeba histolytica HM-1:IMSS]	QLFIEQIKSVDNVDK	1775.953806	2	1.5446
gi|56469970|gb|EAL47665.1| Rab family GTPase [Entamoeba histolytica HM-1:IMSS] gi|14423577|gb|AAK62471.1| small GT	LLLIGESGVGK	1085.656506	2	2.7005
gi|56470003|gb|EAL47695.1| hypothetical protein 94.t00010 [Entamoeba histolytica HM-1:IMSS]	ISSIVNDITQK	1217.673617	2	1.6281
gi|56470046|gb|EAL47735.1| hypothetical protein 93.t00010 [Entamoeba histolytica HM-1:IMSS]	IVQIPEKYK	1117.661574	2	1.8472
gi|56470066|gb|EAL47752.1| hypothetical protein 92.t00015 [Entamoeba histolytica HM-1:IMSS]	QLPEQFYKDLFK	1555.81551	2	2.0416
gi|56470084|gb|EAL47770.1| hypothetical protein 92.t00038 [Entamoeba histolytica HM-1:IMSS]	KILAEM*TKMKNER	1607.861859	3	1.564
gi|56470085|gb|EAL47771.1| 40S ribosomal protein S10, putative [Entamoeba histolytica HM-1:IMSS]	LALPEDAVPSTLK	1353.762422	2	2.1727
gi|56470088|gb|EAL47773.1| 60S ribosomal protein L23, putative [Entamoeba histolytica HM-1:IMSS]	MHTAVIIR	940.539683	2	1.9437
gi|56470108|gb|EAL47792.1| chromodomain-helicase-DNA-binding protein, putative [Entamoeba histolytica HM-1:IMSS]	KGGRDDILKQGHVYSLR	1942.06174	2	1.7931
gi|56470117|gb|EAL47800.1| Myb family DNA-binding protein [Entamoeba histolytica HM-1:IMSS]	AAGIQVSYSHK	1160.605873	3	1.5747
gi|56470123|gb|EAL47806.1| nucleotide binding protein 2, putative [Entamoeba histolytica HM-1:IMSS]	NFVGVDHVK	1014.53671	2	1.8214
	STIATVLAR	931.557119	2	2.0997
gi|56470136|gb|EAL47817.1| hypothetical protein 90.t00004 [Entamoeba histolytica HM-1:IMSS]	ISNNSRR	846.454061	2	1.6088
gi|56470144|gb|EAL47825.1| hypothetical protein 90.t00012 [Entamoeba histolytica HM-1:IMSS]	RGLMEM*IYKLLLKDDIK	2095.166483	2	1.5795
gi|56470146|gb|EAL47827.1| hypothetical protein 90.t00014 [Entamoeba histolytica HM-1:IMSS]	ENSM*TLK	838.398605	2	1.7515
	KTIHTIK	840.530165	2	2.122
gi|56470151|gb|EAL47832.1| 70 kDa heat shock protein, putative [Entamoeba histolytica HM-1:IMSS]	ITPSVVAFTDHDILVGEAAR	2111.113163	2	1.6129
gi|56470154|gb|EAL47835.1| hypothetical protein 90.t00022 [Entamoeba histolytica HM-1:IMSS]	IFSNTRSNGEIM*KIVR	1881.002216	3	1.8286
	IGRSLELRFNEIK	1574.901317	2	1.6407

gi|56470176|gb|EAL47856.1| hypothetical protein 89.t00014 [Entamoeba histolytica HM-1:IMSS]	NKKEEISK	975.546949	2	1.9543
gi|56470178|gb|EAL47858.1| splicing factor Prp8, putative [Entamoeba histolytica HM-1:IMSS]	LTLEDLEDSWDK	1463.690047	2	1.6992
	VDLTLLNR	943.557109	2	1.5325
gi|56470188|gb|EAL47866.1| myristoyl CoA:protein N-myristoyltransferase, putative [Entamoeba histolytica HM-1:IMSS]	YEPVTGTGVIVPQK	1487.81043	2	2.2694
gi|56470201|gb|EAL47879.1| ameobapore A precursor [Entamoeba histolytica HM-1:IMSS]	LIQLIEDK	971.577176	2	1.9934
gi|56470214|gb|EAL47892.1| conserved hypothetical protein [Entamoeba histolytica HM-1:IMSS]	NDEEHKSLTWTVPVKSEEVK	2355.182709	3	1.6247
gi|56470240|gb|EAL47916.1| hypothetical protein 87.t00006 [Entamoeba histolytica HM-1:IMSS]	QVNEKEVQVNDKEKQVNEK	2285.173187	3	1.726
gi|56470246|gb|EAL47922.1| Rho family GTPase [Entamoeba histolytica HM-1:IMSS]	EIFELAAK	920.508762	1	2.4163
	EIFELAAK	920.508762	2	2.0196
	EIFELAAK	920.508762	2	1.94
	ESIEVMAK	906.460107	1	1.9945
	VIVIGDTNVGK	1114.646658	2	2.778
gi|56470287|gb|EAL47959.1| proteasome beta subunit, putative [Entamoeba histolytica HM-1:IMSS]	AVTYNYDAVGSFEERK	1848.876287	2	1.7502
gi|56470309|gb|EAL47980.1| PH domain protein [Entamoeba histolytica HM-1:IMSS]	GYIDVPVGTEVNDVSGAK	1819.907258	2	1.5296
gi|56470312|gb|EAL47982.1| conserved hypothetical protein [Entamoeba histolytica HM-1:IMSS]	LIYM*EK	812.423352	2	1.7975
gi|56470329|gb|EAL47998.1| hypothetical protein 84.t00015 [Entamoeba histolytica HM-1:IMSS]	NANGRREAR	1043.545318	2	1.6307
gi|56470331|gb|EAL48000.1| histone H3, putative [Entamoeba histolytica HM-1:IMSS]	DMQLAR	733.366136	2	1.745
	STELLLR	831.493456	2	2.2747
	STELLLR	831.493456	1	1.7683
	VTIMPK	688.406209	1	1.6105
	VTIMPK	688.406209	2	1.5624
gi|56470383|gb|EAL48049.1| OFR 1.5 protein-related [Entamoeba histolytica HM-1:IMSS]	KM*LVCNDIIFIVVDNCKK	2225.107251	2	2.8223
	KM*LVCNDIIFIVVDNCKK	2225.107251	2	2.5623
	KM*LVCNDIIFIVVDNCKK	2225.107251	2	1.8512
gi|56470384|gb|EAL48050.1| conserved hypothetical protein [Entamoeba histolytica HM-1:IMSS]	NGSNAGYSIEQILQSK	1708.850095	2	1.7868
gi|56470397|gb|EAL48061.1| Viral A-type inclusion protein repeat, putative [Entamoeba histolytica HM-1:IMSS]	DRVISELNDIK	1301.70597	3	1.5694
	ENEITKLNEDK	1332.664156	2	2.1458
gi|56470398|gb|EAL48062.1| 40S ribosomal protein S3a, putative [Entamoeba histolytica HM-1:IMSS]	LLDQHQDTSAPVEAAPVAEEK	2248.1092	2	5.3324
gi|56470414|gb|EAL48077.1| hypothetical protein 82.t00021 [Entamoeba histolytica HM-1:IMSS]	KEKYKESHVQIR	1544.854365	3	1.673
gi|56470445|gb|EAL48102.1| DNA-directed RNA polymerase subunit, putative [Entamoeba histolytica HM-1:IMSS]	NKTLKDCSLR	1234.63578	3	1.6778
gi|56470472|gb|EAL48126.1| hypothetical protein 80.t00019 [Entamoeba histolytica HM-1:IMSS]	IVEISDIPK	1013.587751	2	2.326
gi|56470484|gb|EAL48138.1| hypothetical protein 80.t00031 [Entamoeba histolytica HM-1:IMSS]	DNIIIPTLLSRYINLIIR	2140.28525	3	1.7768
gi|56470522|gb|EAL48169.1| hypothetical protein 79.t00022 [Entamoeba histolytica HM-1:IMSS]	VGTIENIIKIMNCIIIK	1972.111892	2	2.4424
gi|56470526|gb|EAL48173.1| 40S ribosomal protein S14, putative [Entamoeba histolytica HM-1:IMSS]	DLGVTSLHIK	1082.62045	2	3.0435
	DLGVTSLHIK	1082.62045	2	2.3906
	TPGPGAQAALR	1038.569076	2	2.9193
gi|56470539|gb|EAL48184.1| sulfate adenylyltransferase, putative [Entamoeba histolytica HM-1:IMSS]	DHAGVGDYYGPFDAQK	1739.766003	2	2.4217
	NVLQGGYPLDMR	1362.683454	2	1.9818
	VTTLSEGEFPIK	1320.704575	2	2.5447
gi|56470552|gb|EAL48196.1| hypothetical protein 78.t00016 [Entamoeba histolytica HM-1:IMSS]	SLFVESPQIPITRVRK	1870.090917	3	1.6997
gi|56470555|gb|EAL48199.1| ribophorin I, putative [Entamoeba histolytica HM-1:IMSS]	NDFNYEGIYLK	1375.652864	2	1.573
	SYIEETVYNQPSFGTYYK	2189.007372	2	2.0959
gi|56470560|gb|EAL48204.1| hypothetical protein 78.t00024 [Entamoeba histolytica HM-1:IMSS]	RMDLESISFNDYSPLRSQK	2286.118353	3	2.3135
gi|56470595|gb|EAL48237.1| hypothetical protein 77.t00024 [Entamoeba histolytica HM-1:IMSS]	EFKELFEPILM*LTKK	1882.040536	2	1.9278
gi|56470601|gb|EAL48243.1| hypothetical protein 77.t00030 [Entamoeba histolytica HM-1:IMSS]	ILLEEEEGEAPTPK	1554.789753	2	3.9835
	TITILNAQPPLK	1308.788566	2	2.6352
gi|56470625|gb|EAL48264.1| 60S ribosomal protein L30, putative [Entamoeba histolytica HM-1:IMSS]	IEYYAVLAK	1069.592826	2	2.5628
	IEYYAVLAK	1069.592826	1	1.815
	IEYYAVLAK	1069.592826	1	1.7409
gi|56470646|gb|EAL48283.1| hypothetical protein 75.t00004 [Entamoeba histolytica HM-1:IMSS]	HRASVGSRNDMFNSFDERK	2253.05781	2	1.5418
gi|56470650|gb|EAL48287.1| conserved hypothetical protein [Entamoeba histolytica HM-1:IMSS]	ALPKPSASAQSTPK	1382.763839	2	1.7914

gi|56470660|gb|EAL48297.1| hypothetical protein 75.t00019 [Entamoeba histolytica HM-1:IMSS]	SAIDGLIR	844.4887074	2	1.9918
	TLLALENR	929.541459	2	2.3361
	TLLALENR	929.541459	2	2.2988
gi|56470665|gb|EAL48302.1| hypothetical protein 75.t00024 [Entamoeba histolytica HM-1:IMSS]	SSSFAQRFNISLSSETKK	2017.034961	2	1.6869
gi|56470688|gb|EAL48322.1| exosome complex exonuclease RRP4, putative [Entamoeba histolytica HM-1:IMSS]	TEIDNLNIK	1059.568069	2	1.554
gi|56470696|gb|EAL48330.1| actin, putative [Entamoeba histolytica HM-1:IMSS]	EKMAQIMFEGFGMK	1646.773924	2	1.8912
gi|56470751|gb|EAL48382.1| Rho guanine nucleotide exchange factor, putative [Entamoeba histolytica HM-1:IMSS]	SIIGSNRTHLPSVAQSAPVM*K	2209.176907	3	1.8615
gi|56470779|gb|EAL48404.1| hypothetical protein 72.t00025 [Entamoeba histolytica HM-1:IMSS]	LKLTNSLLSDLLFNLHK	1969.148098	2	2.0177
gi|56470811|gb|EAL48431.1| conserved hypothetical protein [Entamoeba histolytica HM-1:IMSS]	VKEFPKQVTEYIYRDPPK	2237.196486	3	1.8738
gi|56470857|gb|EAL48473.1| hypothetical protein 70.t00009 [Entamoeba histolytica HM-1:IMSS]	SKMGNIIEMFYKKK	1716.917559	2	2.2992
gi|56470875|gb|EAL48490.1| hypothetical protein 69.t00020 [Entamoeba histolytica HM-1:IMSS]	EYTSNTFK	989.457466	1	1.7237
gi|56470969|gb|EAL48575.1| hypothetical protein 67.t00007 [Entamoeba histolytica HM-1:IMSS]	DKALTISGEIETSVK	1590.858521	2	1.8398
gi|56470972|gb|EAL48578.1| 40S ribosomal protein S15a, putative [Entamoeba histolytica HM-1:IMSS]	HQYITDYTVVDTHR	1747.839829	3	4.2081
	ILGFFY	759.4075924	1	1.966
	IVVNLNGR	884.5312304	2	1.9031
	IVVNLNGR	884.5312304	2	1.7441
	VNVLADALK	942.56186	2	2.6912
	VNVLADALK	942.56186	1	2.1698
	VNVLADALK	942.56186	1	1.9968
	VNVLADALK	942.56186	2	1.9743
	VNVLADALK	942.56186	1	1.9033
	VNVLADALK	942.56186	1	1.7221
gi|56470981|gb|EAL48586.1| hypothetical protein 67.t00001 [Entamoeba histolytica HM-1:IMSS]	ISRKGNRR	986.5966344	2	1.5018
gi|56471008|gb|EAL48611.1| acyl-CoA synthetase, putative [Entamoeba histolytica HM-1:IMSS]	NIKKIYCIGRSVDLSELNK	2250.206026	2	2.3688
gi|56471034|gb|EAL48633.1| protein kinase, putative [Entamoeba histolytica HM-1:IMSS]	IENNMINK	975.492794	2	2.0078
gi|56471042|gb|EAL48641.1| type A flavoprotein [Entamoeba histolytica HM-1:IMSS]	TIDSTKNKQFK	1309.711055	3	1.5194
gi|56471055|gb|EAL48651.1| protein with RhoGEF and ArfGAP domains [Entamoeba histolytica HM-1:IMSS]	VEQLQDGTKLIEIVEK	1842.021878	2	1.551
gi|56471071|gb|EAL48667.1| WD repeat protein [Entamoeba histolytica HM-1:IMSS]	KVINVNDTM*VVR	1403.768611	2	1.5736
gi|56471091|gb|EAL48685.1| hypothetical protein 63.t00001 [Entamoeba histolytica HM-1:IMSS]	GKTNEFLITDK	1265.673599	2	1.5198
gi|56471106|gb|EAL48700.1| hypothetical protein 63.t00016 [Entamoeba histolytica HM-1:IMSS]	LFSDISKKK	1065.630294	2	1.5916
gi|56471106|gb|EAL48700.1| hypothetical protein 63.t00016 [Entamoeba histolytica HM-1:IMSS]	LFSDISKKK	1065.630294	2	1.5221
gi|56471119|gb|EAL48713.1| Sar family GTPase [Entamoeba histolytica HM-1:IMSS]	IDIPGAVSEAQLR	1368.748171	2	2.2482
	IDIPGAVSEAQLR	1368.748171	2	2.0581
gi|56471133|gb|EAL48725.1| fibrillarin, putative [Entamoeba histolytica HM-1:IMSS]	GGLIIESIK	929.5666258	2	1.8704
gi|56471135|gb|EAL48727.1| conserved hypothetical protein [Entamoeba histolytica HM-1:IMSS]	YLQDNIDYINTQIK	1740.880297	2	3.8218
gi|56471151|gb|EAL48743.1| hypothetical protein 62.t00028 [Entamoeba histolytica HM-1:IMSS]	NNTDKQDLALNQEIVNGK	2014.019982	3	2.0838
gi|56471152|gb|EAL48744.1| hypothetical protein 62.t00029 [Entamoeba histolytica HM-1:IMSS]	GKIINQYLCELFVSSR	1926.989156	2	1.688
	PAYFKDIR	1009.546544	2	1.5091
gi|56471186|gb|EAL48776.1| hypothetical protein 61.t00031 [Entamoeba histolytica HM-1:IMSS]	SENIRQELDK	1231.62772	2	2.3377
gi|56471217|gb|EAL48804.1| 40S ribosomal protein S7, putative [Entamoeba histolytica HM-1:IMSS]	EAVAGIPELK	1026.582992	2	2.9357
	EAVAGIPELK	1026.582992	2	2.8861
	EAVAGIPELK	1026.582992	1	2.6824
	EAVAGIPELK	1026.582992	1	2.392
	LPIYSAVYK	1053.597921	2	1.7101
gi|56471228|gb|EAL48814.1| myosin heavy chain [Entamoeba histolytica HM-1:IMSS]	VEDLEGQLEKAK	1358.716193	2	3.9088
	VEDLEGQLEKAK	1358.716193	2	2.3176
gi|56471243|gb|EAL48827.1| conserved hypothetical protein [Entamoeba histolytica HM-1:IMSS]	FEYVIEK	927.482213	2	1.5442
gi|56471259|gb|EAL48842.1| hypothetical protein 59.t00018 [Entamoeba histolytica HM-1:IMSS]	KNEEESSEEEK	1466.612931	2	1.5197
gi|56471272|gb|EAL48855.1| protein kinase, putative [Entamoeba histolytica HM-1:IMSS]	ICGLSDEK	921.4131594	2	1.6879
gi|56471273|gb|EAL48856.1| helicase, putative [Entamoeba histolytica HM-1:IMSS]	AFKEAM*DSTTKK	1372.678804	3	1.5805
gi|56471292|gb|EAL48872.1| hypothetical protein 58.t00008 [Entamoeba histolytica HM-1:IMSS]	MKQDECIITKTGSKK	1766.892468	2	1.9339

	MKQDECIITKTGSKK	1766.892468	2	1.5812
gi|56471307|gb|EAL48887.1| dipeptidyl-peptidase III, putative [Entamoeba histolytica HM-1:IMSS]	ELIPKGIEALKELLLK	1807.130304	3	1.9866
gi|56471310|gb|EAL48890.1| hypothetical protein 58.t00026 [Entamoeba histolytica HM-1:IMSS]	DANSEIK	776.378487	2	1.9799
gi|56471315|gb|EAL48895.1| hypothetical protein 58.t00031 [Entamoeba histolytica HM-1:IMSS]	IVGLFNQTKKETEEANTRK	2206.18263	3	1.7878
gi|56471327|gb|EAL48906.1| hypothetical protein 57.t00027 [Entamoeba histolytica HM-1:IMSS]	NEVTQKPLNPIQLSK	1708.959225	3	1.6975
gi|56471344|gb|EAL48921.1| hypothetical protein 57.t00005 [Entamoeba histolytica HM-1:IMSS]	TIVEFDNSAQEIK	1493.74823	2	1.8215
gi|56471364|gb|EAL48939.1| nuclear pore protein, putative [Entamoeba histolytica HM-1:IMSS]	VTTDNSNVFLEKKGIKK	1921.07532	2	1.5317
gi|56471386|gb|EAL48958.1| AMP deaminase, putative [Entamoeba histolytica HM-1:IMSS]	KDININK	844.488695	2	2.2397
gi|56471403|gb|EAL48974.1| 40S ribosomal protein S4, putative [Entamoeba histolytica HM-1:IMSS]	FDIGNTAMMIGGNGMGR	1741.781871	2	2.6103
	FDIGNTAMMIGGNGMGR	1741.781871	2	2.1836
	GTETLVNLPLDK	1299.715464	2	3.7572
	GTETLVNLPLDK	1299.715464	2	3.5728
	GTETLVNLPLDK	1299.715464	2	3.2225
	GTETLVNLPLDK	1299.715464	1	2.5815
	IVDFVK	720.429054	2	1.7376
	KPLLQQVNETIK	1410.831494	3	3.586
	KPLLQQVNETIK	1410.831494	2	3.4178
	KPLLQQVNETIK	1410.831494	3	2.7784
	LGGTFAPK	790.4457718	2	2.5978
	LNLETGK	774.4355994	1	1.7352
	LNNVFVIGK	1003.593496	2	3.1505
	LNNVFVIGK	1003.593496	2	1.8868
	LNNVFVIGK	1003.593496	1	1.5864
	YPHPELQANDTIK	1525.764538	2	2.3446
gi|56471426|gb|EAL48996.1| ankyrin repeat protein, putative [Entamoeba histolytica HM-1:IMSS]	TPFSSIQFKINKK	1537.873713	2	1.9923
gi|56471436|gb|EAL49006.1| villidin, putative [Entamoeba histolytica HM-1:IMSS]	NITLNPLFIR	1200.709921	2	1.572
gi|56471438|gb|EAL49007.1| exportin, putative [Entamoeba histolytica HM-1:IMSS]	KNNETINK	960.510888	1	1.5032
gi|56471452|gb|EAL49021.1| hypothetical protein 54.t00033 [Entamoeba histolytica HM-1:IMSS]	KKRSVEDILK	1215.74196	2	2.2222
	KKRSVEDILK	1215.74196	1	1.9757
gi|56471454|gb|EAL49023.1| hypothetical protein 54.t00035 [Entamoeba histolytica HM-1:IMSS]	LPFEEEDFAK	1224.5783	2	1.6486
	MDLTEQPQLPNDILSNK	1955.974284	2	2.8397
gi|56471480|gb|EAL49047.1| 60S ribosomal protein L35, putative [Entamoeba histolytica HM-1:IMSS]	LLTVMNTQR	1075.592842	2	2.4782
gi|56471496|gb|EAL49060.1| Gal/GalNAc lectin subunit Igl1 [Entamoeba histolytica HM-1:IMSS]	HIPTSIDK	910.49927	2	1.5714
gi|56471502|gb|EAL49066.1| ribulose-phosphate 3-epimerase, putative [Entamoeba histolytica HM-1:IMSS]	VLIMTVEPGFGGQSFM*QDMMPK	2459.148876	3	1.5806
gi|56471520|gb|EAL49084.1| RNA methyltransferase, TrmH family, putative [Entamoeba histolytica HM-1:IMSS]	NKRILM*TM*QEAGDM*LSTM*K	2262.064079	2	1.6459
gi|56471527|gb|EAL49091.1| hypothetical protein 53.t00040 [Entamoeba histolytica HM-1:IMSS]	CSGLGRFKIMHQGLLNNK	2073.063	2	1.8167
gi|56471531|gb|EAL49095.1| hypothetical protein 53.t00045 [Entamoeba histolytica HM-1:IMSS]	ELIQQNDDKQSDKDDLM*K	2179.019434	2	1.5963
gi|56471553|gb|EAL49114.1| conserved hypothetical protein [Entamoeba histolytica HM-1:IMSS]	MSMFFAYSSPMM*K	1573.656905	3	1.6156
gi|56471556|gb|EAL49117.1| sodium/sulfate symporter, putative [Entamoeba histolytica HM-1:IMSS]	DM*YQELWSFNDKK	1719.769411	2	1.7106
gi|56471561|gb|EAL49122.1| immuno-dominant variable surface antigen [Entamoeba histolytica HM-1:IMSS]	NQGLSGWNYVSDGYSTIYK	2151.998219	2	3.6163
gi|56471578|gb|EAL49136.1| Rho GDP exchange inhibitor, putative [Entamoeba histolytica HM-1:IMSS]	TPIIIKEGAEFKMR	1632.914179	2	1.7133
gi|56471588|gb|EAL49146.1| conserved hypothetical protein [Entamoeba histolytica HM-1:IMSS]	MVPNKDVLFILM*K	1563.864818	2	1.7808
gi|56471604|gb|EAL49161.1| hypothetical protein 50.t00039 [Entamoeba histolytica HM-1:IMSS]	EM*AINK	721.356001	1	1.5602
gi|56471613|gb|EAL49169.1| protein kinase, putative [Entamoeba histolytica HM-1:IMSS]	KGTSCPK	777.3708994	2	1.5384
gi|56471629|gb|EAL49184.1| hypothetical protein 50.t00015 [Entamoeba histolytica HM-1:IMSS]	TIGLEGVNSLTR	1259.69541	2	2.1502
gi|56471639|gb|EAL49192.1| 40S ribosomal protein S2, putative [Entamoeba histolytica HM-1:IMSS]	EEVMTMQSVQK	1309.612662	2	3.6943
	EYQIVDHLLPGLK	1524.842061	2	3.7577
	LAMVPVR	785.470206	2	1.8196
	LAMVPVR	785.470206	2	1.7855
	SIDQIFEFGIK	1296.683445	2	3.6153
	TLGNFVK	778.4457694	1	1.6215

gi|56471641|gb|EAL49194.1| 60S ribosomal protein L15, putative [Entamoeba histolytica HM-1:IMSS]	VLNSYFIGQDSTYR	1662.812239	2	3.3021
	VLNSYFIGQDSTYR	1662.812239	2	2.6424
gi|56471644|gb|EAL49197.1| amino acid transporter, putative [Entamoeba histolytica HM-1:IMSS]	QIVPHQNIVNYLK	1565.879841	3	1.6842
gi|56471654|gb|EAL49206.1| helicase, putative [Entamoeba histolytica HM-1:IMSS]	LKGVCSFLDKMIIEGKK	1966.064955	2	1.5744
gi|56471697|gb|EAL49244.1| 60S ribosomal protein L7, putative [Entamoeba histolytica HM-1:IMSS]	QPMETRIIVGTPENM*SKLK	2188.14755	2	1.6079
gi|56471698|gb|EAL49245.1| hypothetical protein 48.t00010 [Entamoeba histolytica HM-1:IMSS]	LILSNPSTLQTK	1314.762766	3	2.493
gi|56471702|gb|EAL49249.1| hypothetical protein 48.t00014 [Entamoeba histolytica HM-1:IMSS]	DGFELK	708.3562864	2	2.2784
	DGFELK	708.3562864	1	1.8329
	FFNVQNDEEEVAEETNAQ	2112.899258	2	4.7422
	FFNVQNDEEEVAEETNAQ	2112.899258	2	4.1618
	FFNVQNDEEEVAEETNAQ	2112.899258	2	2.8641
	FFNVQNDEEEVAEETNAQ	2112.899258	2	2.3126
	KLVTFFR	910.5509	2	1.9524
	KLVTFFR	910.5509	2	1.8034
	LVTFFR	782.455937	2	1.5003
	VTITTSSAK	907.509511	2	2.1093
	VTITTSSAK	907.509511	2	2.0322
gi|56471711|gb|EAL49258.1| hypothetical protein 48.t00023 [Entamoeba histolytica HM-1:IMSS]	ISPNQNYISILEK	1518.816259	2	1.5182
gi|56471713|gb|EAL49260.1| protein kinase, putative [Entamoeba histolytica HM-1:IMSS]	IGFIPPISDCYIIILSFDR	2239.161701	2	1.936
gi|56471733|gb|EAL49278.1| pyruvate phosphate dikinase [Entamoeba histolytica HM-1:IMSS]	MILSNTLEER	1205.619462	2	2.4063
	TEGLAEKEVVLK	1315.746764	2	1.5545
gi|56471739|gb|EAL49284.1| cell division control protein 7, putative [Entamoeba histolytica HM-1:IMSS]	KNFAM*LLKSLIK	1421.855978	2	1.6512
gi|56471740|gb|EAL49285.1| hypothetical protein 47.t00023 [Entamoeba histolytica HM-1:IMSS]	HEMIFDEEVK	1276.587818	2	1.8055
	LPLVAGRHGLDYK	1438.816517	2	1.6077
	LPLVAGRHGLDYK	1438.816517	2	1.5998
gi|56471746|gb|EAL49291.1| 40S ribosomal protein S18, putative [Entamoeba histolytica HM-1:IMSS]	EDIEALK	817.430178	1	1.7101
gi|56471752|gb|EAL49297.1| hypothetical protein 47.t00036 [Entamoeba histolytica HM-1:IMSS]	KLVTTTKEQLSQFIK	1764.026576	2	1.9011
gi|56471777|gb|EAL49321.1| protein phosphatase, putative [Entamoeba histolytica HM-1:IMSS]	IIKSSGVSLPKLSNSYLLKR	2203.317321	3	1.6529
gi|56471797|gb|EAL49340.1| protein with RhoGEF and ArfGAP domains [Entamoeba histolytica HM-1:IMSS]	LIQFGNGFGNILR	1448.800869	3	1.5688
gi|56471798|gb|EAL49341.1| hypothetical protein 46.t00002 [Entamoeba histolytica HM-1:IMSS]	LIPDITIK	912.576447	2	1.6633
gi|56471809|gb|EAL49351.1| 70 kDa heat shock protein, putative [Entamoeba histolytica HM-1:IMSS]	IAGLNVLR	855.5410664	2	2.3473
	NQIAQNPENTIFEIK	1758.902095	2	2.3224
	NQIAQNPENTIFEIK	1758.902095	2	1.8276
gi|56471813|gb|EAL49355.1| hypothetical protein 45.t00008 [Entamoeba histolytica HM-1:IMSS]	MILGSELFK	1037.569993	2	1.5189
gi|56471815|gb|EAL49357.1| hypothetical protein 45.t00011 [Entamoeba histolytica HM-1:IMSS]	DAIQGPFVVPSNADEIK	1799.917423	2	3.3498
	DAIQGPFVVPSNADEIK	1799.917423	2	2.8903
	DAIQGPFVVPSNADEIK	1799.917423	2	2.2189
gi|56471819|gb|EAL49361.1| SMC4 protein, putative [Entamoeba histolytica HM-1:IMSS]	IEENAQKIK	1072.599703	2	1.5418
gi|56471833|gb|EAL49375.1| hypothetical protein 45.t00029 [Entamoeba histolytica HM-1:IMSS]	ILLEEYGK	964.5349794	1	1.5431
gi|56471835|gb|EAL49377.1| hypothetical protein 45.t00031 [Entamoeba histolytica HM-1:IMSS]	QLTIKNKRLSGM*TLDLLCK	2248.231219	3	1.6248
gi|56471843|gb|EAL49383.1| hypothetical protein 44.t00025 [Entamoeba histolytica HM-1:IMSS]	RYTLLLTNLPHKR	1624.964573	2	1.5557
gi|56471852|gb|EAL49391.1| filopodin, putative [Entamoeba histolytica HM-1:IMSS]	LPERPQGEFILWQPTR	1967.049763	3	1.8788
gi|56471880|gb|EAL49413.1| hypothetical protein 43.t00025 [Entamoeba histolytica HM-1:IMSS]	PMAETSVAIDIPRLVSK	1827.00447	2	2.0388
	PMAETSVAIDIPRLVSK	1827.00447	2	1.6471
gi|56471886|gb|EAL49419.1| hypothetical protein 43.t00031 [Entamoeba histolytica HM-1:IMSS]	MM*MTMKK	916.413395	1	1.6918
gi|56471888|gb|EAL49421.1| hypothetical protein 43.t00033 [Entamoeba histolytica HM-1:IMSS]	KNIKIENIVNAWSIINK	1997.154236	2	1.5208
gi|56471933|gb|EAL49460.1| hypothetical protein 42.t00022 [Entamoeba histolytica HM-1:IMSS]	SSTEITVLDK	1092.57832	2	1.7507
gi|56471939|gb|EAL49466.1| protein disulfide isomerase, putative [Entamoeba histolytica HM-1:IMSS]	TVDNLVEFSKK	1279.689257	2	1.6713
gi|56471944|gb|EAL49471.1| 60S ribosomal protein L36, putative [Entamoeba histolytica HM-1:IMSS]	HMMDLLR	915.453904	2	2.1629
	HMMDLLR	915.453904	2	1.805
	HVVNDVIR	951.537042	2	2.1498

gi|56471978|gb|EAL49502.1| DNA-directed RNA polymerase subunit, putative [Entamoeba histolytica HM-1:IMSS]	IIYIALM*VNWILK	1605.944783	3	1.5875
gi|56472000|gb|EAL49524.1| hypothetical protein 41.t00043 [Entamoeba histolytica HM-1:IMSS]	M*THLENVFLKNVLLYLPTLK	2402.352703	2	1.7169
gi|56472035|gb|EAL49554.1| steroid 5-alpha reductase, putative [Entamoeba histolytica HM-1:IMSS]	DLGAQLPFR	1016.55236	2	2.2174
	DLGAQLPFR	1016.55236	1	1.6515
	TLQESGVTEDTPIEMK	1777.85244	2	4.774
	TLQESGVTEDTPIEMK	1777.85244	2	4.5242
	TLQESGVTEDTPIEMK	1777.85244	2	4.2624
	TLQESGVTEDTPIEMK	1777.85244	2	3.1091
	YLHMFPDYPK	1310.623808	3	3.1097
	YLHMFPDYPK	1310.623808	3	2.9678
gi|56472100|gb|EAL49612.1| alanyl-tRNA synthetase, putative [Entamoeba histolytica HM-1:IMSS]	EVKAM*TPK	919.492829	2	1.7293
gi|56472114|gb|EAL49624.1| Nonsense-mediated mRNA decay protein 3, putative [Entamoeba histolytica HM-1:IMSS]	GLNKLKLIMQHSKR	1665.994504	2	1.6105
gi|56472138|gb|EAL49647.1| hypothetical protein 38.t00019 [Entamoeba histolytica HM-1:IMSS]	TPQILKVQMKYYSKK	1855.051015	2	1.5293
gi|56472184|gb|EAL49691.1| GTP-binding protein, putative [Entamoeba histolytica HM-1:IMSS]	INDIELELSR	1201.642307	2	1.6635
gi|56472246|gb|EAL49745.1| Rap/Ran GTPase activating protein, putative [Entamoeba histolytica HM-1:IMSS]	VELDLDSR	946.484015	2	1.5271
gi|56472247|gb|EAL49746.1| hypothetical protein 35.t00045 [Entamoeba histolytica HM-1:IMSS]	RKLLEIKPSTESLPFFSSVK	2306.31189	3	1.9596
gi|56472252|gb|EAL49751.1| glycogen phosphorylase, putative [Entamoeba histolytica HM-1:IMSS]	NALFDVM*VK	1052.545593	2	1.9054
gi|56472276|gb|EAL49773.1| hypothetical protein 35.t00007 [Entamoeba histolytica HM-1:IMSS]	LPISLQTVDVDNIK	1554.873764	2	2.2262
gi|56472283|gb|EAL49780.1| histone H2B [Entamoeba histolytica HM-1:IMSS]	IATEASSLAR	1018.552773	1	1.5202
	SVFQDIGITLPSISIMDSFVR	2325.215943	3	1.7459
gi|56472299|gb|EAL49794.1| hypothetical protein 34.t00035 [Entamoeba histolytica HM-1:IMSS]	IDVNKETENAKR	1416.744137	3	2.0773
gi|56472305|gb|EAL49800.1| conserved hypothetical protein [Entamoeba histolytica HM-1:IMSS]	NKTIITSEEFEK	1438.742416	3	2.2034
gi|56472321|gb|EAL49815.1| hypothetical protein 34.t00051 [Entamoeba histolytica HM-1:IMSS]	VITFENEVLK	1191.661969	2	1.7019
gi|56472337|gb|EAL49830.1| adaptor-related protein, putative [Entamoeba histolytica HM-1:IMSS]	KAFTLSKK	922.57204	1	1.6733
	YLNDEILLPVNPK	1527.841725	2	1.9963
gi|56472348|gb|EAL49839.1| hypothetical protein 33.t00032 [Entamoeba histolytica HM-1:IMSS]	PEANNGNEGRTKLK	1527.787404	2	1.9221
gi|56472365|gb|EAL49855.1| calreticulin, putative [Entamoeba histolytica HM-1:IMSS]	FDEDWDMLAPK	1366.598383	2	4.8418
	KPEEWNDEDDGEWEAPTIENPEYK	2920.243183	3	3.099
	KPEEWNDEDDGEWEAPTIENPEYK	2920.243183	3	2.6693
	THVIMNYK	1005.518614	2	2.0415
gi|56472404|gb|EAL49892.1| DNA (cytosine-5)-methyltransferase, putative [Entamoeba histolytica HM-1:IMSS]	YYVM*ARLTPFK	1404.735519	2	1.5341
gi|56472413|gb|EAL49899.1| ARF GTPase activating protein, putative [Entamoeba histolytica HM-1:IMSS]	KSAHLAEK	883.499604	2	2.2202
gi|56472426|gb|EAL49912.1| cullin, putative [Entamoeba histolytica HM-1:IMSS]	KYCKTLIQLHSIK	1631.908707	2	1.5134
gi|56472427|gb|EAL49913.1| protein kinase, putative [Entamoeba histolytica HM-1:IMSS]	NKLPTESKK	1044.604798	2	1.6006
gi|56472456|gb|EAL49940.1| hypothetical protein 31.t00009 [Entamoeba histolytica HM-1:IMSS]	IVEYM*NKEITFIVAK	1813.977936	3	1.7465
gi|56472457|gb|EAL49941.1| hypothetical protein 31.t00010 [Entamoeba histolytica HM-1:IMSS]	YIETDNTIK	1096.552085	1	1.7034
gi|56472492|gb|EAL49974.1| Rho guanine nucleotide exchange factor, putative [Entamoeba histolytica HM-1:IMSS]	TLPRTLVREGILR	1523.938026	2	1.5964
gi|56472506|gb|EAL49988.1| hypothetical protein 30.t00039 [Entamoeba histolytica HM-1:IMSS]	LMNFEIISTK	1195.639134	2	2.1367
gi|56472550|gb|EAL50029.1| galactose-inhibitable lectin 170 kda subunit [Entamoeba histolytica HM-1:IMSS]	CKVSNCTEDLVR	1480.645359	2	2.4927
	CKVSNCTEDLVR	1480.645359	2	2.2077
	MDFIGGR	795.3817908	2	2.1309
	SLQENLSLLSQLK	1472.831919	2	3.8742
	YEVDWDNVPVDEQR	1763.787126	2	4.0432
	YEVDWDNVPVDEQR	1763.787126	2	3.6302
	YEVDWDNVPVDEQR	1763.787126	2	2.5427
	YTAGTGQVDPLINIAK	1660.890472	2	4.0664
	YTAGTGQVDPLINIAK	1660.890472	2	3.8173
	YTAGTGQVDPLINIAK	1660.890472	2	3.5746
	YTAGTGQVDPLINIAK	1660.890472	2	3.4187
gi|56472559|gb|EAL50038.1| regulator of nonsense transcripts, putative [Entamoeba histolytica HM-1:IMSS]	FVIHRTR	928.547546	1	1.5816
gi|56472605|gb|EAL50076.1| hypothetical protein 28.t00031 [Entamoeba histolytica HM-1:IMSS]	RKEMIEKQNQEYWK	1909.958897	2	1.6003

gi|56472606|gb|EAL50077.1| hypothetical protein 28.t00032 [Entamoeba histolytica HM-1:IMSS]	QLKDQIVQLQSSLKK	1756.032734	2	1.8955
gi|56472628|gb|EAL50098.1| hypothetical protein 28.t00012 [Entamoeba histolytica HM-1:IMSS]	FVLEQIKYKYCNIKTR	2103.120482	2	2.313
gi|56472629|gb|EAL50099.1| 60S ribosomal protein L23a, putative [Entamoeba histolytica HM-1:IMSS]	FPLATETALK	1090.61429	2	2.591
	FPLATETALK	1090.61429	2	2.5346
	FPLATETALK	1090.61429	2	2.516
	FPLATETALK	1090.61429	1	2.1123
	FPLATETALK	1090.61429	1	1.6609
	LAPDVEAMEVATK	1373.698097	2	3.5677
	LAPDVEAMEVATK	1373.698097	2	3.0594
	MDEFSIIK	982.491407	2	2.3811
	MDEFSIIK	982.491407	2	2.1024
	MDEFSIIK	982.491407	1	1.7507
	MDEFSIIK	982.491407	1	1.7323
	MDEFSIIK	982.491407	1	1.5273
gi|56472660|gb|EAL50126.1| translation initiation factor eIF-2B epsilon subunit, putative [Entamoeba histolytica HM-1:IMSS]	DGIEKSTKPNDQLFTSR	1935.977074	2	1.6939
gi|56472666|gb|EAL50132.1| DEAD/DEAH box helicase, putative [Entamoeba histolytica HM-1:IMSS]	GKQVIVFVHTR	1283.75827	2	1.5759
gi|56472695|gb|EAL50160.1| hypothetical protein 27.t00007 [Entamoeba histolytica HM-1:IMSS]	KTPSLK	673.424313	1	1.5286
gi|56472715|gb|EAL50179.1| histidyl-tRNA synthetase, putative [Entamoeba histolytica HM-1:IMSS]	QLTIDDDKLIPALVYR	1873.042945	2	1.6655
gi|56472724|gb|EAL50188.1| hypothetical protein 26.t00052 [Entamoeba histolytica HM-1:IMSS]	NEWDAIVNETDLILSSEPK	2173.065948	2	2.5465
gi|56472729|gb|EAL50193.1| hypothetical protein 26.t00057 [Entamoeba histolytica HM-1:IMSS]	IPLKTVQIKNVSFSGR	1787.053805	2	1.5218
	KQLPSLPQK	1038.630618	2	1.5869
	KQLPSLPQK	1038.630618	2	1.5335
gi|56472744|gb|EAL50207.1| protein kinase, putative [Entamoeba histolytica HM-1:IMSS]	IAQGSFSIVFKGK	1381.78384	2	1.7116
gi|56472751|gb|EAL50213.1| 60S ribosomal protein L32, putative [Entamoeba histolytica HM-1:IMSS]	NTTDLEMIMM*QNR	1612.713875	2	1.9278
	YAAEISHAVSAK	1246.642651	3	2.8151
gi|56472775|gb|EAL50234.1| Rab geranylgeranyltransferase alpha subunit, putative [Entamoeba histolytica HM-1:IMSS]	KIDSTIELLSNEEVK	1717.921848	2	1.7737
gi|56472788|gb|EAL50247.1| conserved hypothetical protein [Entamoeba histolytica HM-1:IMSS]	LKTEIDK	846.493112	1	1.6613
gi|56472797|gb|EAL50256.1| hypothetical protein 25.t00024 [Entamoeba histolytica HM-1:IMSS]	GVSNIWENVAQTLVFCK	1964.968411	2	1.572
	LSNLLKKK	943.629889	2	2.2393
gi|56472798|gb|EAL50257.1| conserved hypothetical protein [Entamoeba histolytica HM-1:IMSS]	ILVEMNNPCNDFMK	1724.758998	2	1.7269
gi|56472807|gb|EAL50266.1| histone H4 [Entamoeba histolytica HM-1:IMSS]	DSVTYTEHAK	1150.537508	2	2.5797
	GITKPAIR	855.5410664	2	1.7395
	QFLEQVIR	1032.583658	2	2.4976
	QFLEQVIR	1032.583658	2	2.1376
	QFLEQVIR	1032.583658	2	2.1375
	TVTAMDVVYALK	1310.702453	2	3.7528
gi|56472809|gb|EAL50268.1| 60S ribosomal protein L29, putative [Entamoeba histolytica HM-1:IMSS]	GMNVQVLK	888.4971524	2	2.0423
	GMNVQVLK	888.4971524	1	1.571
gi|56472823|gb|EAL50281.1| ARP2/3 complex 20 kDa subunit, putative [Entamoeba histolytica HM-1:IMSS]	IAVAGITGTSPAPGFFK	1633.89484	2	3.1882
gi|56472825|gb|EAL50283.1| 60S ribosomal protein L19, putative [Entamoeba histolytica HM-1:IMSS]	ALMESIFK	938.501577	2	2.5632
	ALMESIFK	938.501577	2	2.5057
	ALMESIFK	938.501577	1	1.6248
	ALMESIFK	938.501577	1	1.6183
	ALMESIFK	938.501577	1	1.5607
	LAASVLK	701.455613	2	1.5675
	TLWIIR	801.498136	2	1.7576
gi|56472826|gb|EAL50284.1| CorA family transporter, putative [Entamoeba histolytica HM-1:IMSS]	SNMVSKNLK	1020.550663	2	1.7184
gi|56472835|gb|EAL50293.1| cysteine protease 10 [Entamoeba histolytica HM-1:IMSS]	KPVIKIK	825.592036	2	1.5666
gi|56472837|gb|EAL50295.1| EH-domain containing protein, putative [Entamoeba histolytica HM-1:IMSS]	YNLAAGDFPPIQLYKEK	1967.027297	2	1.6276
gi|56472869|gb|EAL50327.1| hypothetical protein 24.t00055 [Entamoeba histolytica HM-1:IMSS]	ANLVAETINFLADVIEMK	1991.051795	3	1.8351
gi|56472872|gb|EAL50330.1| hypothetical protein 24.t00058 [Entamoeba histolytica HM-1:IMSS]	CSFNEIVIDSHFM*EIM*KM*AK	2478.09904	2	2.0194

gi|56472873|gb|EAL50331.1| hypothetical protein 24.t00059 [Entamoeba histolytica HM-1:IMSS]	KLEENDENINTIKNDKK	2045.050946	2	1.7638
gi|56472881|gb|EAL50337.1| chaperone protein dnaK, putative [Entamoeba histolytica HM-1:IMSS]	INENIKTK	959.552024	2	1.6553
gi|56472940|gb|EAL50394.1| hypothetical protein 23.t00023 [Entamoeba histolytica HM-1:IMSS]	M*DIVFIK	881.481201	2	1.5107
gi|56472953|gb|EAL50406.1| helicase, putative [Entamoeba histolytica HM-1:IMSS]	YLKNQLEVFIDDGEK	1810.922164	2	1.8347
gi|56472956|gb|EAL50409.1| hypothetical protein 22.t00043 [Entamoeba histolytica HM-1:IMSS]	SINLALM*LLVK	1230.750116	2	1.5441
gi|56472969|gb|EAL50422.1| conserved hypothetical protein [Entamoeba histolytica HM-1:IMSS]	FLVYGGKGWIGGIIINM*LKK	2223.273341	2	2.0754
gi|56472989|gb|EAL50440.1| aspartate ammonia-lyase, putative [Entamoeba histolytica HM-1:IMSS]	TNLVHDLK	939.525809	2	1.7473
gi|56472997|gb|EAL50447.1| exonuclease, putative [Entamoeba histolytica HM-1:IMSS]	MIFIGVDGVCPVAKIIQQR	2144.150406	2	1.5838
gi|56473002|gb|EAL50452.1| Phosphatidylinositol 3-kinase, putative [Entamoeba histolytica HM-1:IMSS]	MTTQMKWSDK	1255.580967	2	1.7161
gi|56473043|gb|EAL50491.1| phosphoinositide kinase, putative [Entamoeba histolytica HM-1:IMSS]	EPTKTYDLK	1094.57282	2	1.6681
gi|56473053|gb|EAL50501.1| Rho guanine nucleotide exchange factor, putative [Entamoeba histolytica HM-1:IMSS]	ITSGKSHIFVGLK	1386.810389	2	1.6925
gi|56473057|gb|EAL50505.1| hypothetical protein 21.t00051 [Entamoeba histolytica HM-1:IMSS]	AKLVKGMK	874.5542724	2	1.6574
	VSILEAELSR	1116.625938	2	2.2123
gi|56473089|gb|EAL50535.1| hypothetical protein 20.t00031 [Entamoeba histolytica HM-1:IMSS]	NCRTENIKTNIK	1490.752926	3	1.7349
gi|56473090|gb|EAL50536.1| Rab family GTPase [Entamoeba histolytica HM-1:IMSS] gi|56790126|dbj|BAD82859.1| EhRabX	EALQLK	701.419218	1	1.7162
	EALQLK	701.419218	1	1.6939
	IIVIGEPSVGK	1111.672154	2	1.8628
gi|56473130|gb|EAL50574.1| conserved hypothetical protein [Entamoeba histolytica HM-1:IMSS]	SKEEIKAEIKR	1330.768904	3	1.9765
gi|56473133|gb|EAL50576.1| conserved hypothetical protein [Entamoeba histolytica HM-1:IMSS]	RGSHLITNEVIKCVSQQLK	2210.185959	2	2.399
gi|56473173|gb|EAL50616.1| hypothetical protein 19.t00056 [Entamoeba histolytica HM-1:IMSS]	YGYGNTFEFK	1225.552424	1	1.939
gi|56473196|gb|EAL50638.1| 60S ribosomal protein L18, putative [Entamoeba histolytica HM-1:IMSS]	FDELALK	835.455998	2	2.2591
	FDELALK	835.455998	2	2.0856
	FDELALK	835.455998	1	1.9703
	FDELALK	835.455998	1	1.8339
	LYGFLAR	839.4774034	2	2.2462
	VNRPIVSTSK	1100.642256	2	2.8775
	VNRPIVSTSK	1100.642256	3	2.3228
gi|56473218|gb|EAL50657.1| D-tyrosyl-tRNA(Tyr) deacylase, putative [Entamoeba histolytica HM-1:IMSS]	GLLLYFGINEEDK	1510.778796	3	1.6814
	ISTEVDGPVNIIVDYPK	1858.979689	2	1.7148
gi|56473238|gb|EAL50677.1| hypothetical protein 18.t00058 [Entamoeba histolytica HM-1:IMSS]	KKQRLK	800.546483	2	1.7421
gi|56473247|gb|EAL50686.1| hypothetical protein 18.t00068 [Entamoeba histolytica HM-1:IMSS]	IDHAQLDYENALPSAK	1784.88137	3	3.1072
	KAVKKAVK	871.608749	2	1.598
gi|56473248|gb|EAL50687.1| hypothetical protein 18.t00069 [Entamoeba histolytica HM-1:IMSS]	NEFVTILLNGEK	1376.742013	2	1.9596
gi|56473284|gb|EAL50718.1| hypothetical protein 17.t00014 [Entamoeba histolytica HM-1:IMSS]	VIDSPLQNIHLK	1376.789639	2	1.7726
	VIDSPLQNIHLK	1376.789639	2	1.6011
gi|56473286|gb|EAL50720.1| hypothetical protein 17.t00016 [Entamoeba histolytica HM-1:IMSS]	HLALEGDQK	1010.52654	2	2.3866
gi|56473295|gb|EAL50729.1| hypothetical protein 17.t00026 [Entamoeba histolytica HM-1:IMSS]	ALRDM*LASSNDPNAQK	1746.845046	2	1.6035
	VLINVNERYDK	1362.737594	2	1.6032
gi|56473296|gb|EAL50730.1| 60S ribosomal protein L4, putative [Entamoeba histolytica HM-1:IMSS]	VENLSVLNLAPGCHMGR	1866.909852	3	2.4374
gi|56473301|gb|EAL50735.1| 40S ribosomal protein S13, putative [Entamoeba histolytica HM-1:IMSS]	DTHGVGLVK	925.5101638	3	2.4163
	GIAPSQIGAIMR	1213.67217	2	4.1
	GIAPSQIGAIMR	1213.67217	2	3.442
	GIAPSQIGAIMR	1213.67217	1	2.3447
	KGIAPSQIGAIMR	1341.767133	3	2.605
	LAGLAPK	669.4293904	2	2.579
	LAGLAPK	669.4293904	1	1.5418
	YHLILIESK	1115.645934	2	3.0435
	YHLILIESK	1115.645934	2	2.9466
	YHLILIESK	1115.645934	1	2.4859
	YHLILIESK	1115.645934	1	2.2828
gi|56473313|gb|EAL50747.1| protein kinase, putative [Entamoeba histolytica HM-1:IMSS]	GKEM*KKIGR	1062.609929	2	1.5047

gi|56473317|gb|EAL50751.1| mRNA capping enzyme, putative [Entamoeba histolytica HM-1:IMSS]	M*EVLENFKLEPK	1492.772694	2	1.9748
gi|56473325|gb|EAL50759.1| galactose-inhibitable lectin 35 kda subunit precursor [Entamoeba histolytica HM-1:IMSS]	DQLSPNYPYGKM*NK	1670.785397	2	1.7882
	HFYLPYR	995.509764	2	2.3136
	HFYLPYR	995.509764	2	1.9017
	SYQIIASR	937.510179	2	2.4648
gi|56473331|gb|EAL50765.1| protein with RhoGEF and ArfGAP domains [Entamoeba histolytica HM-1:IMSS]	EDKTIRNCFKNLEM*IVK	2154.084209	2	1.9311
	EDKTIRNCFKNLEM*IVK	2154.084209	2	1.7666
gi|56473334|gb|EAL50768.1| Ras GTPase activating protein, putative [Entamoeba histolytica HM-1:IMSS]	EISDLLK	817.466573	2	1.6785
	ILQQLFQQQSPFPVNDTNK	2245.161174	2	4.174
	ILQQLFQQQSPFPVNDTNK	2245.161174	2	3.4357
	ILQQLFQQQSPFPVNDTNK	2245.161174	2	2.7163
	NIKTLEEKNLVSKEDGYK	2108.123394	3	2.1902
	YISQSLEDEDQTLYNR	1973.908719	2	4.1157
gi|56473353|gb|EAL50783.1| long-chain-fatty-acid--CoA ligase, putative [Entamoeba histolytica HM-1:IMSS]	VEAFNQTLPR	1174.621501	2	1.6776
gi|56473368|gb|EAL50797.1| Gal/GalNAc lectin heavy subunit [Entamoeba histolytica HM-1:IMSS]	YTAGQGQVDPLVNIAK	1673.885721	2	4.5937
	YTAGQGQVDPLVNIAK	1673.885721	2	3.5432
gi|56473376|gb|EAL50805.1| Brix domain protein, putative [Entamoeba histolytica HM-1:IMSS]	LYDYDDDEAWMK	1563.630807	2	1.6845
gi|56473405|gb|EAL50834.1| ubiquitin carboxyl-terminal hydrolase, putative [Entamoeba histolytica HM-1:IMSS]	DIELELNK	973.520056	2	2.0986
gi|56473423|gb|EAL50849.1| hypothetical protein 15.t00033 [Entamoeba histolytica HM-1:IMSS]	KSNVFNIDFKSSTR	1642.85478	2	1.6104
gi|56473426|gb|EAL50852.1| hypothetical protein 15.t00036 [Entamoeba histolytica HM-1:IMSS]	IDLIEEVTPKIMVTR	1756.987737	3	2.1903
gi|56473438|gb|EAL50864.1| clathrin assembly protein, putative [Entamoeba histolytica HM-1:IMSS]	YVVILDK	849.508033	1	1.6971
gi|56473474|gb|EAL50899.1| 40S ribosomal protein S9, putative [Entamoeba histolytica HM-1:IMSS]	EENLLER	902.45779	2	1.5082
	LEFVLALK	932.581532	1	2.0278
	LHNLGILPR	1032.631278	3	3.034
	LHNLGILPR	1032.631278	3	2.6555
	LHNLGILPR	1032.631278	2	2.4296
	LHNLGILPR	1032.631278	2	1.9017
	LLEGAALLR	955.5934964	2	3.3058
	LLEGAALLR	955.5934964	2	3.1797
	LLEGAALLR	955.5934964	2	3.0859
	LLEGAALLR	955.5934964	1	1.8993
	LLEGAALLR	955.5934964	1	1.795
	LLEGAALLR	955.5934964	1	1.5927
	LQTLVYR	892.52508	2	2.1247
	LVGQFGLK	861.5192708	2	1.9917
	LVGQFGLK	861.5192708	2	1.6842
	LVGQFGLK	861.5192708	1	1.5771
	QVVDVPSFLVR	1258.715411	2	2.4703
	QVVDVPSFLVR	1258.715411	2	2.3393
	RLQTLVYR	1048.626191	2	2.0299
	VESEPLIQLADNTPLTNPEINGR	2520.294043	2	3.0396
gi|56473520|gb|EAL50942.1| hypothetical protein 14.t00029 [Entamoeba histolytica HM-1:IMSS]	YEKDLMNVK	1139.576524	2	2.1128
gi|56473566|gb|EAL50985.1| translation initiation factor 2 beta subunit, putative [Entamoeba histolytica HM-1:IMSS]	SLDTELIK	918.514252	2	1.796
gi|56473575|gb|EAL50994.1| hypothetical protein 13.t00037 [Entamoeba histolytica HM-1:IMSS]	KGNTVLHYLCEIGINKR	2015.064035	2	1.5107
gi|56473594|gb|EAL51013.1| hypothetical protein 13.t00058 [Entamoeba histolytica HM-1:IMSS]	HIIGSEDLRK	1167.648062	2	1.8321
	M*DQSCQVGLFPQIEKPLLGR	2332.158452	3	1.8253
gi|56473603|gb|EAL51022.1| 60S ribosomal protein L24, putative [Entamoeba histolytica HM-1:IMSS]	SFTFINK	856.456342	2	1.717
	SFTFINK	856.456342	1	1.6675
	SFTFINK	856.456342	1	1.5742
	SLMQFLR	894.486595	2	2.2833
	SLMQFLR	894.486595	1	1.7456

	SLMQFLR	894.486595	2	1.7028
gi|56473612|gb|EAL51030.1| hypothetical protein 13.t00006 [Entamoeba histolytica HM-1:IMSS]	ALADAITAVTK	1073.620104	2	2.9511
	ALADAITAVTK	1073.620104	2	2.6879
	VIEVEDIVVPDR	1382.752576	2	2.0172
gi|56473639|gb|EAL51055.1| conserved hypothetical protein [Entamoeba histolytica HM-1:IMSS]	EITAINQLEDMLKAMTIR	2090.098427	2	1.5042
gi|56473676|gb|EAL51091.1| Rho GTPase activating protein, putative [Entamoeba histolytica HM-1:IMSS]	ALFNSITVK	992.57752	2	1.7585
gi|56473707|gb|EAL51119.1| hypothetical protein 11.t00062 [Entamoeba histolytica HM-1:IMSS]	RM*M*AEAILNEK	1337.657381	2	1.5513
gi|56473713|gb|EAL51125.1| DnaJ family protein [Entamoeba histolytica HM-1:IMSS]	MNDDYYK	948.376762	2	1.7145
gi|56473716|gb|EAL51128.1| hypothetical protein 11.t00071 [Entamoeba histolytica HM-1:IMSS]	FFNGNFEFDGK	1321.584787	3	1.9668
gi|56473743|gb|EAL51152.1| protein phosphatase, putative [Entamoeba histolytica HM-1:IMSS]	DM*AFARGSTDNVSCVVCKF	2179.915117	3	1.5771
gi|56473794|gb|EAL51197.1| structural maintenance of chromosomes protein [Entamoeba histolytica HM-1:IMSS]	IDEFEKQKSEKEER	1794.88685	2	1.8871
	IDEFEKQKSEKEER	1794.88685	2	1.5769
gi|56473807|gb|EAL51210.1| conserved hypothetical protein [Entamoeba histolytica HM-1:IMSS]	TLKGLQEGLK	1086.651743	1	1.6372
	VEAEKRAK	930.536708	2	1.6914
gi|56473809|gb|EAL51212.1| Truncated P-glycoprotein 6 [Entamoeba histolytica HM-1:IMSS]	NFTIDQVELDNIK	1548.790419	2	1.519
gi|56473820|gb|EAL51223.1| conserved hypothetical protein [Entamoeba histolytica HM-1:IMSS]	DVMDEKIIDLYDK	1596.782556	3	1.7815
gi|56473822|gb|EAL51224.1| actin [Entamoeba histolytica HM-1:IMSS]	DLTDYLM*K	1014.482325	2	1.8128
	DLTDYLM*K	1014.482325	2	1.8054
	DLTDYLMK	998.486312	1	2.7151
	DLTDYLMK	998.486312	2	2.5113
	DLTDYLMK	998.486312	2	2.4466
	DLTDYLMK	998.486312	2	2.2117
	DLTDYLMK	998.486312	1	2.0128
	DLYGNIVLSGGTSMYPGINTR	2228.10163	2	5.5286
	DLYGNIVLSGGTSMYPGINTR	2228.10163	2	4.6525
	DLYGNIVLSGGTSMYPGINTR	2228.10163	2	4.2206
	DLYGNIVLSGGTSMYPGINTR	2228.10163	2	4.0475
	DLYGNIVLSGGTSMYPGINTR	2228.10163	2	3.6273
	DLYGNIVLSGGTSMYPGINTR	2228.10163	3	3.6147
	EEYDESGPAIVHR	1501.69178	2	3.9161
	EMIQLAPPTMK	1258.653393	2	3.6054
	EMIQLAPPTMK	1258.653393	2	3.0573
	EMIQLAPPTMK	1258.653393	2	2.9688
	EMIQLAPPTMK	1258.653393	1	2.6548
	EMIQLAPPTMK	1258.653393	1	2.3832
	GYAFTTTAER	1116.53202	2	3.6303
	GYAFTTTAER	1116.53202	2	3.2186
	LCYVAEDFNEEMQK	1775.740039	2	1.6709
	LDLAGR	644.3726044	2	1.6863
	LEKEMIQLAPPTMK	1628.875014	2	1.6277
	SYELPDGQVITVGNER	1776.87629	2	4.4451
	SYELPDGQVITVGNER	1776.87629	2	4.1385
	SYELPDGQVITVGNER	1776.87629	2	3.9651
	SYELPDGQVITVGNER	1776.87629	2	3.8787
	SYELPDGQVITVGNER	1776.87629	2	3.535
	SYELPDGQVITVGNER	1776.87629	2	3.346
	SYELPDGQVITVGNER	1776.87629	2	3.2174
	SYELPDGQVITVGNER	1776.87629	2	3.1876
	SYELPDGQVITVGNER	1776.87629	2	3.0777
	SYELPDGQVITVGNER	1776.87629	3	2.439
	SYELPDGQVITVGNER	1776.87629	2	2.4078

	VAPEEHPVLLTEAPM*NPK	1988.016842	3	3.1238
	VAPEEHPVLLTEAPMNPK	1972.020829	3	4.0414
	VAPEEHPVLLTEAPMNPK	1972.020829	2	4.0153
	VAPEEHPVLLTEAPMNPK	1972.020829	2	3.9119
	VAPEEHPVLLTEAPMNPK	1972.020829	2	3.4826
	VAPEEHPVLLTEAPMNPK	1972.020829	3	2.9884
	VAPEEHPVLLTEAPMNPK	1972.020829	3	2.6794
gi|56473830|gb|EAL51232.1| aminopeptidase, putative [Entamoeba histolytica HM-1:IMSS]	LVAEVMK	789.453888	2	1.5698
gi|56473842|gb|EAL51244.1| protein kinase, putative [Entamoeba histolytica HM-1:IMSS]	LGEGGFGIVYK	1139.609549	2	1.6057
gi|56473881|gb|EAL51280.1| conserved hypothetical protein [Entamoeba histolytica HM-1:IMSS]	LIIQALEK	927.587346	2	2.3938
	LIIQALEK	927.587346	2	2.01
	MAESFHAKQLSNPM*VK	1833.899722	2	1.7881
	QKRNWGMLVFQLHQQHRR	2362.25742	3	1.7213
gi|56473886|gb|EAL51285.1| histone H2A, putative [Entamoeba histolytica HM-1:IMSS]	EKEVTPVVEK	1157.641234	2	2.7453
	EVTPVVEK	900.503677	1	2.1129
gi|56473892|gb|EAL51291.1| ADP-ribosylation factor, putative [Entamoeba histolytica HM-1:IMSS]	ILMVGLDAAGK	1087.617999	2	2.9491
gi|56473898|gb|EAL51297.1| Dopey domain protein, putative [Entamoeba histolytica HM-1:IMSS]	NQLNNK	730.38423	1	1.6776
gi|56473901|gb|EAL51300.1| carbonic anhydrase, putative [Entamoeba histolytica HM-1:IMSS]	RKELVNK	886.546878	2	1.6988
gi|56473913|gb|EAL51311.1| hypothetical protein 9.t00002 [Entamoeba histolytica HM-1:IMSS]	IM*YKLYYEK	1266.644973	2	1.8594
gi|56473945|gb|EAL51340.1| thioredoxin, putative [Entamoeba histolytica HM-1:IMSS]	SISPYFEELAGQYNNIK	1972.965112	2	2.9703
gi|56473955|gb|EAL51349.1| cysteine protease 12-related [Entamoeba histolytica HM-1:IMSS]	GGILRGLKCEK	1230.677247	2	1.9109
	KLIFM*KNLMEIK	1523.869903	3	2.1599
gi|56473961|gb|EAL51355.1| actin, putative [Entamoeba histolytica HM-1:IMSS]	AGFAGDDAPR	976.4482928	2	2.1979
	AVFPSIVGRPR	1198.705516	2	2.9472
	AVFPSIVGRPR	1198.705516	2	2.2718
	DAYVGDEAQSK	1182.52734	2	2.5072
	GILTLK	644.4341414	2	1.576
	YPIEHGIVNNWDDMEK	1959.890546	2	5.2901
	YPIEHGIVNNWDDMEK	1959.890546	3	4.4074
	YPIEHGIVNNWDDMEK	1959.890546	3	4.266
	YPIEHGIVNNWDDMEK	1959.890546	3	2.8057
gi|56473964|gb|EAL51358.1| chromosome partition protein, putative [Entamoeba histolytica HM-1:IMSS]	EM*NKIITEKETMKLQIDMK	2339.203004	3	1.9628
	TDDIFEIKK	1108.58847	1	1.9354
gi|56473994|gb|EAL51388.1| chorein, putative [Entamoeba histolytica HM-1:IMSS]	LVELIYKQANFTDIK	1795.000017	2	2.1947
gi|56474013|gb|EAL51407.1| Vesicle-fusing ATPase, putative [Entamoeba histolytica HM-1:IMSS]	NDSTGVSDTIVNQLLSK	1790.913087	2	1.776
gi|56474025|gb|EAL51417.1| 40S ribosomal protein SA, putative [Entamoeba histolytica HM-1:IMSS]	LIIVSDPSVDHQALR	1662.91737	3	1.9355
	VIAGISNETPSDVVAVAGR	1854.991998	2	5.3766
	VIAGISNETPSDVVAVAGR	1854.991998	2	4.7092
gi|56474026|gb|EAL51418.1| Sec6 protein, putative [Entamoeba histolytica HM-1:IMSS]	MISTSLVKTFVLAK	1537.902235	2	2.2605
gi|56474044|gb|EAL51435.1| 40S ribosomal protein S23, putative [Entamoeba histolytica HM-1:IMSS]	DGSLNYVNENDEVLISGFGR	2198.036052	2	3.5779
	DGSLNYVNENDEVLISGFGR	2198.036052	2	2.0681
	GHAVGDIPGVR	1077.579977	3	2.0023
	ITAFVPR	803.477401	2	1.5721
	LGIETK	660.3926714	2	1.693
gi|56474046|gb|EAL51437.1| Ser/Thr protein phosphatase, putative [Entamoeba histolytica HM-1:IMSS]	IFAIGDIHLNFDLVTKAK	2015.13243	2	1.6987
	YPGFDSNPSKKIDYLKITK	2214.180524	2	2.4481
gi|56474053|gb|EAL51443.1| hypothetical protein 7.t00065 [Entamoeba histolytica HM-1:IMSS]	EGTLVYKSYDVVPEVEDLR	2211.117975	2	2.8096
gi|56474085|gb|EAL51473.1| hypothetical protein 7.t00019 [Entamoeba histolytica HM-1:IMSS]	QIQKQK	772.467565	2	1.9605
gi|56474108|gb|EAL51496.1| Sec7 domain protein [Entamoeba histolytica HM-1:IMSS]	M*NNQHQHKIIMK	1537.773712	2	1.5179
gi|56474131|gb|EAL51514.1| hypothetical protein 6.t00007 [Entamoeba histolytica HM-1:IMSS]	RSLSNSLSELVK	1332.748199	2	1.7457
gi|56474150|gb|EAL51533.1| hypothetical protein 6.t00031 [Entamoeba histolytica HM-1:IMSS]	KAANEKKK	916.557443	2	1.6686

gi|56474152|gb|EAL51535.1| hypothetical protein 6.t00033 [Entamoeba histolytica HM-1:IMSS]	DILMVK	718.416774	2	1.5693
gi|56474198|gb|EAL51581.1| hypothetical protein 6.t00082 [Entamoeba histolytica HM-1:IMSS]	M*LPINNYTEIK	1351.693715	2	2.1827
gi|56474202|gb|EAL51585.1| hypothetical protein 6.t00086 [Entamoeba histolytica HM-1:IMSS]	VVAKIDGKNN	1057.600039	2	1.5243
gi|56474223|gb|EAL51605.1| protein kinase, putative [Entamoeba histolytica HM-1:IMSS]	MHQNSSFTDRLIIIQTKTTK	2362.254766	3	1.9591
gi|56474232|gb|EAL51612.1| hypothetical protein 5.t00072 [Entamoeba histolytica HM-1:IMSS]	QEKEVVVDDGEEKREISK	2117.072088	2	1.5416
gi|56474240|gb|EAL51620.1| hypothetical protein 5.t00080 [Entamoeba histolytica HM-1:IMSS]	HLFGLHDIVQK	1306.726636	2	1.6011
gi|56474241|gb|EAL51621.1| SMC5 protein, putative [Entamoeba histolytica HM-1:IMSS]	IGQLYDDAKNEAVK	1563.80132	3	1.8308
	RNIEENRK	1058.570134	2	1.5263
gi|56474246|gb|EAL51626.1| conserved hypothetical protein [Entamoeba histolytica HM-1:IMSS]	AFNELIEFR	1138.589138	2	3.1191
	DIAEELLK	930.514242	1	2.1597
	DIAEELLK	930.514242	2	2.0077
	DIAEELLK	930.514242	1	1.7832
	EGLVVVDER	1015.541856	2	2.6851
	EIAEYIK	865.466563	1	1.5736
	ELDFYVMK	1044.507047	2	1.5702
	ENMTQIYQFDTQK	1645.752653	2	3.6066
	EVTEVIEK	946.509157	2	1.9968
	FVNFDQAAEDAAK	1425.66449	2	3.695
	GGNILFIGK	918.5407372	2	2.8484
	GGNILFIGK	918.5407372	1	1.7608
	IEKDGEVLNVNFASIKPLYKK	2405.343891	3	1.7862
	IIEEVNAM*CK	1222.560257	2	1.6139
	KVDAPGGQFIIETPER	1699.901368	2	1.683
	NGYVTISTFEENMESVK	1947.900463	2	2.4639
	TNIAIPLLK	982.629545	2	2.8551
	TNIAIPLLK	982.629545	2	2.6958
	TNIAIPLLK	982.629545	2	2.4272
	TNIAIPLLK	982.629545	1	2.067
	TNIAIPLLK	982.629545	2	1.7304
	VTGIITVDK	945.5615284	2	1.6954
	VYLTGFDGEK	1128.557175	2	2.45
	VYLTGFDGEK	1128.557175	2	2.3412
	VYLTGFDGEK	1128.557175	1	1.7086
gi|56474266|gb|EAL51645.1| myosin heavy chain [Entamoeba histolytica HM-1:IMSS]	DLTNELQQTQLK	1430.748554	2	3.5178
	ELEIQSLQEK	1216.641973	2	2.0102
	FQINAPSSVAQEEEK	1676.812632	2	3.8304
	ISELEEQVNELESR	1674.818112	2	3.1023
	NQSILITGESGAGK	1374.722366	2	3.4625
	RYYFLGDKINR	1444.769564	2	1.7884
	TMNITDDEQNQIFK	1696.784682	2	3.6041
	TMNITDDEQNQIFK	1696.784682	2	3.0847
gi|56474322|gb|EAL51697.1| hypothetical protein 4.t00120 [Entamoeba histolytica HM-1:IMSS]	YLQRNKIISEGIEM*R	1865.991307	3	1.6356
gi|56474340|gb|EAL51714.1| hypothetical protein 4.t00099 [Entamoeba histolytica HM-1:IMSS]	PLHPHFNPIFSK	1433.768843	2	1.5729
gi|56474341|gb|EAL51715.1| hypothetical protein 4.t00100 [Entamoeba histolytica HM-1:IMSS]	ELFAKILAMQNKSR	1648.920335	2	2.9922
gi|56474348|gb|EAL51722.1| clathrin coat assembly protein, putative [Entamoeba histolytica HM-1:IMSS]	HTNFLEFHNLK	1399.711713	3	2.538
gi|56474362|gb|EAL51735.1| Rab family GTPase [Entamoeba histolytica HM-1:IMSS] gi|56790132|dbj|BAD82862.1| EhRabX	AESEQIPPSQPEK	1439.70129	3	2.3223
	AESEQIPPSQPEK	1439.70129	3	2.1915
gi|56474395|gb|EAL51767.1| 40S ribosomal protein S7, putative [Entamoeba histolytica HM-1:IMSS]	ATVIFIPLR	1029.645529	2	2.3981
	KVGQSGKTDYK	1210.642646	2	1.5802
	TVFVAVDDAK	1064.562255	2	2.5964
	TVFVAVDDAK	1064.562255	2	1.6616

gi|56474464|gb|EAL51831.1| 60S ribosomal protein L10, putative [Entamoeba histolytica HM-1:IMSS]	ENISSEAMEAAR	1307.58963	2	3.7519
gi|56474491|gb|EAL51858.1| hypothetical protein 3.t00139 [Entamoeba histolytica HM-1:IMSS]	YVTYCELSK	1162.523436	1	1.5895
gi|56474516|gb|EAL51881.1| hypothetical protein 3.t00011 [Entamoeba histolytica HM-1:IMSS]	MNNPIKTLGK	1115.624144	2	1.5937
gi|56474518|gb|EAL51883.1| conserved hypothetical protein [Entamoeba histolytica HM-1:IMSS]	EILISENR	973.531299	2	2.2486
gi|56474534|gb|EAL51899.1| hypothetical protein 3.t00026 [Entamoeba histolytica HM-1:IMSS]	LIVPSDLK	884.545157	2	1.893
gi|56474536|gb|EAL51901.1| eukaryotic initiation factor 4A, putative [Entamoeba histolytica HM-1:IMSS]	NNVLNEFR	1005.511222	2	2.2146
	QFYIDVVKDEYK	1546.778791	3	2.1735
	SLPSTVQVGMFSATMPADALDISK	2466.225522	2	3.2844
gi|56474541|gb|EAL51906.1| MIT domain protein [Entamoeba histolytica HM-1:IMSS]	FSRGFSGEMKGETTKTEMAK	2222.058047	3	1.6888
gi|56474542|gb|EAL51907.1| Viral A-type inclusion protein repeat, putative [Entamoeba histolytica HM-1:IMSS]	GKIDNMMLEAM*ELVK	1737.859478	3	2.1143
gi|56474578|gb|EAL51943.1| hypothetical protein 3.t00071 [Entamoeba histolytica HM-1:IMSS]	ILNNNQDRLGLITGK	1668.939153	3	1.6067
gi|56474648|gb|EAL52010.1| Rho guanine nucleotide exchange factor, putative [Entamoeba histolytica HM-1:IMSS]	ECIFNTETYVEYCSHENVCK	2581.998861	3	1.8373
gi|56474660|gb|EAL52022.1| protein kinase, putative [Entamoeba histolytica HM-1:IMSS]	TNHENIVKMKGVFK	1644.889027	2	1.874
gi|56474661|gb|EAL52023.1| hypothetical protein 2.t00099 [Entamoeba histolytica HM-1:IMSS]	EIEEEM*KMVTK	1382.655281	3	1.8605
gi|56474687|gb|EAL52048.1| conserved hypothetical protein [Entamoeba histolytica HM-1:IMSS]	M*FVQPPPAQYIKR	1590.847195	3	2.5525
	LVGNAMEIQEDKKTRIK	1973.084828	3	2.5613
	RSREIMSIMNDIEINLSMIR	2421.241115	3	1.8924
gi|56474761|gb|EAL52118.1| 40S ribosomal protein S3, putative [Entamoeba histolytica HM-1:IMSS]	ALNVDVLK	871.524746	2	1.5221
	IPDVVEIK	912.540062	2	2.0917
gi|56474835|gb|EAL52191.1| hypothetical protein 1.t00057 [Entamoeba histolytica HM-1:IMSS]	LISSSDSKK	964.530995	2	1.6095
gi|56474842|gb|EAL52198.1| interaptin, putative [Entamoeba histolytica HM-1:IMSS]	AQQIQAQNKEQEQQK	1798.904221	3	1.8956
	RKEEITRLQK	1300.769562	2	1.736
gi|7494130|pir||T18292 nicotinamide nucleotide transhydrogenase homolog - Entamoeba histolytica	LLDLSVQK	915.550971	2	2.0886
	LLDLSVQK	915.550971	1	1.9712
	M*GNIYGIIGMTVAFIAAVVDK	2199.156318	3	1.8875
	MWPAPQIQVVSQNTPAK	1894.984393	2	3.5701
	MWPAPQIQVVSQNTPAK	1894.984393	2	2.7668
	MWPAPQIQVVSQNTPAK	1894.984393	2	2.3567
	QLLPETDIVITTANVPGK	1909.064077	2	2.0691
	QLLPETDIVITTANVPGK	1909.064077	2	1.6297
	SPILITQDMVDSMK	1577.791365	2	3.5077
gi|7579069|gb|AAF64243.1| hsp70 BiP [Entamoeba invadens]	QIEQIAQPILAK	1351.79438	2	3.0109
