# Supplementary material for: Entamoeba histolytica Phagocytosis of Human Erythrocytes Involves PATMK, a Member of the Transmembrane Kinase Family
Source: PLoS Pathog. 2008 Jan 18;4(1):e8. doi: 10.1371/journal.ppat.0040008 (PMC2211552; doi:10.1371/journal.ppat.0040008)
Supplement: Supplementary file 4 [file ppat.0040008.st004.pdf]

30 MINUTES

ANNOTATION	PEPTIDE SEQUENCE	MASS	CHARGE	Xcorr
gi|56465240|gb|EAL43543.1| Rho GTPase activating protein, putative [Entamoeba histolytica HM-1:IMSS]	ENLCFELFYDK	1477.645333	2	2.0779
gi|56465292|gb|EAL43582.1| ubiquitin-activating enzyme, putative [Entamoeba histolytica HM-1:IMSS]	QFKSIVEELHIDLDENIIK	2283.223106	3	2.6059
gi|56465610|gb|EAL43837.1| hypothetical protein 330.t00004 [Entamoeba histolytica HM-1:IMSS]	FDSIWM*QEHDR	1479.633251	2	1.8011
gi|56465672|gb|EAL43889.1| hypothetical protein 324.t00001 [Entamoeba histolytica HM-1:IMSS]	SVNDGLTGISFGQIAK	1606.843544	3	1.6289
gi|56465821|gb|EAL44004.1| hypothetical protein 309.t00001 [Entamoeba histolytica HM-1:IMSS]	YCKVTSLSPLNQTK	1638.830528	2	1.5164
gi|56465851|gb|EAL44027.1| inositol polyphosphate 5-phosphatase, putative [Entamoeba histolytica HM-1:IMSS]	VGAM*SGALANKGGVAYRM*K	1912.975382	3	1.6288
gi|56466015|gb|EAL44160.1| hypothetical protein 290.t00010 [Entamoeba histolytica HM-1:IMSS]	EIPDYIGYFGLDEDDNK	2002.891657	2	2.2465
gi|56466019|gb|EAL44164.1| Truncated, galactose-specific adhesin 170kD subunit [Entamoeba histolytica HM-1:IMSS]	FVQLTDDPSLDSEHFR	1905.897759	3	3.4714
gi|56466506|gb|EAL44584.1| 90 kDa heat shock protein, putative [Entamoeba histolytica HM-1:IMSS]	ALLYIPAEGPNPFAPK	1697.926125	2	3.1582
gi|56466710|gb|EAL44753.1| hypothetical protein 243.t00005 [Entamoeba histolytica HM-1:IMSS]	MEEINTDDSSNRR	1566.6813	2	2.0083
	MEEINTDDSSNRR	1566.6813	2	1.796
gi|56467087|gb|EAL45073.1| ENTH domain protein, putative [Entamoeba histolytica HM-1:IMSS]	PMVLFLGQYSTGK	1440.755566	3	2.1612
gi|56467282|gb|EAL45251.1| FKBP-rapamycin associated protein (FRAP), putative [Entamoeba histolytica HM-1:IMSS]	AEM*ITELEEIIEM*KK	1838.91478	2	3.3701
gi|56467297|gb|EAL45264.1| protein disulfide isomerase, putative [Entamoeba histolytica HM-1:IMSS]	FGISGFPTLK	1066.593175	2	2.5377
	NLNVLEVF	947.519661	1	1.6485
	NLNVLEVF	947.519661	1	1.6376
gi|56467402|gb|EAL45363.1| DEAD/DEAH box helicase, putative [Entamoeba histolytica HM-1:IMSS]	ELLPVEFDCKLNK	1604.813795	3	1.7373
gi|56467435|gb|EAL45394.1| hypothetical protein 199.t00003 [Entamoeba histolytica HM-1:IMSS]	AKDLLPRMQVEWR	1641.889359	3	1.7523
gi|56467520|gb|EAL45473.1| ubiquitin-protein ligase E3 component [Entamoeba histolytica HM-1:IMSS]	IINNMFSDPM*LSNK	1639.782961	3	1.5854
gi|56467575|gb|EAL45521.1| WD repeat protein [Entamoeba histolytica HM-1:IMSS]	DSMTFVNKVR	1196.609231	2	1.5263
gi|56468007|gb|EAL45901.1| Plasma membrane calcium-transporting ATPase, putative [Entamoeba histolytica HM-1:IMSS]	RNENNIYQMFAFSSAK	1919.906882	2	1.8804
gi|56468069|gb|EAL45958.1| hypothetical protein 170.t00006 [Entamoeba histolytica HM-1:IMSS]	IIRIYPHPELNK	1492.863462	2	1.8001
gi|56468213|gb|EAL46087.1| protein phosphatase 2C, putative [Entamoeba histolytica HM-1:IMSS]	SGGMFNIAFDTLK	1400.687881	2	2.1546
gi|56468256|gb|EAL46125.1| protein kinase, putative [Entamoeba histolytica HM-1:IMSS]	SLFEIKDYVLQGKR	1695.942848	3	1.5399
gi|56468265|gb|EAL46133.1| hypothetical protein 161.t00005 [Entamoeba histolytica HM-1:IMSS]	M*YFSLIQPFSNK	1490.735934	2	1.8129
gi|56468903|gb|EAL46707.1| protein kinase, putative [Entamoeba histolytica HM-1:IMSS]	CKKSLEM*IADLLK	1564.823358	2	1.6258
	YM*KLNKM*TTLM*K	1549.781964	2	1.8503
gi|56469115|gb|EAL46901.1| BspA-like leucine rich repeat protein, putative [Entamoeba histolytica HM-1:IMSS]	RYGNEIPPMISVIEQK	1873.984061	3	1.6115
gi|56469194|gb|EAL46969.1| hypothetical protein 121.t00010 [Entamoeba histolytica HM-1:IMSS]	LGLPRGSLKFEKK	1472.894777	2	1.5456
gi|56469245|gb|EAL47008.1| Gal/GalNAc lectin subunit Igl2 [Entamoeba histolytica HM-1:IMSS]	DLYLPYYFSVTK	1508.767173	2	3.1539
	DLYLPYYFSVTK	1508.767173	2	2.3492
gi|56469607|gb|EAL47339.1| protein kinase, putative [Entamoeba histolytica HM-1:IMSS]	ILITLKQCNGLLK	1513.891986	2	1.5377
gi|56469653|gb|EAL47381.1| peroxiredoxin, putative [Entamoeba histolytica HM-1:IMSS]	SMDVRNKEEGGM*IK	1609.768369	3	1.6264
gi|56470210|gb|EAL47888.1| Rho GTPase activating protein, putative [Entamoeba histolytica HM-1:IMSS]	MLVIYTNEKRLR	1535.872646	2	1.6404
gi|56470295|gb|EAL47966.1| DNA repair protein RAD54, putative [Entamoeba histolytica HM-1:IMSS]	SQAPSVRLAKRGVK	1496.901995	2	1.661
gi|56470839|gb|EAL48456.1| auxin efflux carrier family protein, putative [Entamoeba histolytica HM-1:IMSS]	GIAFVSKM*NTKDSR	1569.806476	2	1.9677
gi|56471058|gb|EAL48654.1| RNA-binding protein, putative [Entamoeba histolytica HM-1:IMSS]	ERVKANMGKLAK	1344.778019	2	1.5533
gi|56471175|gb|EAL48765.1| hypothetical protein 61.t00020 [Entamoeba histolytica HM-1:IMSS]	FGGYANEKIDKYEMKK	1920.95242	2	1.5031
gi|56471217|gb|EAL48804.1| 40S ribosomal protein S7, putative [Entamoeba histolytica HM-1:IMSS]	TVFVTVDDAKLKNSVK	1763.990191	3	1.6777
gi|56471375|gb|EAL48950.1| galactose-inhibitable lectin small subunit, putative [Entamoeba histolytica HM-1:IMSS]	QGNELQFFEFAPYR	1745.828203	2	3.7948
gi|56471635|gb|EAL49189.1| hypothetical protein 50.t00042 [Entamoeba histolytica HM-1:IMSS]	SKPSPPLKEQNVVNLSHK	2002.108035	3	1.6552
gi|56471713|gb|EAL49260.1| protein kinase, putative [Entamoeba histolytica HM-1:IMSS]	TIVATNSFETLPNK	1534.811164	2	1.6999
gi|56471809|gb|EAL49351.1| 70 kDa heat shock protein, putative [Entamoeba histolytica HM-1:IMSS]	FEELNLELFK	1281.672534	2	3.2912
	GINPDEAVAYGAAVQGGVLSNIK	2243.166664	2	5.9316
	HAVITVPAYFNDAQR	1701.870734	3	3.4526
	NQIAQNPENTIFEIK	1758.902095	2	1.8805
	QLEQIAQPILAK	1351.79438	2	1.985
gi|56471815|gb|EAL49357.1| hypothetical protein 45.t00011 [Entamoeba histolytica HM-1:IMSS]	DAIQGPFVVPSNADEIK	1799.917423	2	3.431
	DAIQGPFVVPSNADEIK	1799.917423	2	2.7552

	DAIQGPFVVPSNADEIK	1799.917423	2	2.4467
	DAIQGPFVVPSNADEIK	1799.917423	2	1.5399
gi|56471888|gb|EAL49421.1| hypothetical protein 43.t00033 [Entamoeba histolytica HM-1:IMSS]	QSPLVNTIQSQNPILLR	1921.086561	2	2.0499
gi|56471890|gb|EAL49423.1| hypothetical protein 43.t00035 [Entamoeba histolytica HM-1:IMSS]	KLQTLNVYGKM*RIIDK	1936.105932	2	1.9014
gi|56471949|gb|EAL49476.1| hypothetical protein 42.t00046 [Entamoeba histolytica HM-1:IMSS]	FMRVKEISKLM*K	1525.860411	2	1.731
gi|56472203|gb|EAL49706.1| ribonuclease, putative [Entamoeba histolytica HM-1:IMSS]	IIEEKVPMK	1086.622745	2	1.8178
gi|56472365|gb|EAL49855.1| calreticulin, putative [Entamoeba histolytica HM-1:IMSS]	AGTIYDDILITDDIEEAEK	2124.023063	2	4.8517
	AGTIYDDILITDDIEEAEK	2124.023063	2	4.0042
gi|56472516|gb|EAL49998.1| protein disulfide isomerase, putative [Entamoeba histolytica HM-1:IMSS]	GFPTVLVFNGEESK	1523.774055	2	2.1222
	GFPTVLVFNGEESK	1523.774055	2	1.8946
gi|56472550|gb|EAL50029.1| galactose-inhibitable lectin 170 kda subunit [Entamoeba histolytica HM-1:IMSS]	CKVSNCTEDLVR	1480.645359	2	1.9222
	CKVSNCTEDLVR	1480.645359	2	1.7381
	SLQENLSLLSQLK	1472.831919	2	3.4135
gi|56473090|gb|EAL50536.1| Rab family GTPase [Entamoeba histolytica HM-1:IMSS] gi|56790126|dbj|BAD82859.1| EhRabX	FNEQSIPALLVGNK	1529.832235	2	1.7419
gi|56473107|gb|EAL50553.1| Rap/Ran GTPase activating protein, putative [Entamoeba histolytica HM-1:IMSS]	NANNINIQIKDWKMVR	1957.043629	2	1.6051
gi|56473325|gb|EAL50759.1| galactose-inhibitable lectin 35 kda subunit precursor [Entamoeba histolytica HM-1:IMSS]	GGANEGATLIIWPK	1426.768901	2	1.9203
gi|56473649|gb|EAL51064.1| hypothetical protein 12.t00001 [Entamoeba histolytica HM-1:IMSS]	DMRIM*EGLKIGAK	1477.787636	2	1.9483
gi|56473661|gb|EAL51076.1| transcription initiation factor SPT5, putative [Entamoeba histolytica HM-1:IMSS]	DRTMHLFSVFVPK	1576.830457	2	1.6116
gi|56473809|gb|EAL51212.1| Truncated P-glycoprotein 6 [Entamoeba histolytica HM-1:IMSS]	NFTIDQVELDNIK	1548.790419	2	2.1589
gi|56473822|gb|EAL51224.1| actin [Entamoeba histolytica HM-1:IMSS]	DLYGNIVLSGGTSMYPGINTR	2228.10163	2	3.9734
	EMIQLAPPTMK	1258.653393	2	2.7241
	EMIQLAPPTMK	1258.653393	2	2.54
	SYELPDGQVITVGNER	1776.87629	2	3.8889
	SYELPDGQVITVGNER	1776.87629	2	3.6317
	VAPEEHPVLLTEAPMNPK	1972.020829	3	2.8184
gi|56473863|gb|EAL51262.1| hypothetical protein 9.t00024 [Entamoeba histolytica HM-1:IMSS]	PVEYTEAWQTSMVR	1696.799947	3	1.5739
gi|56473893|gb|EAL51292.1| glycyl-tRNA synthetase, putative [Entamoeba histolytica HM-1:IMSS]	MQRPATKFNKEK	1477.794396	2	2.1332
gi|56473947|gb|EAL51342.1| protein kinase, putative [Entamoeba histolytica HM-1:IMSS]	PENVLFSGTKSEIK	1548.826826	2	1.8249
gi|56473961|gb|EAL51355.1| actin, putative [Entamoeba histolytica HM-1:IMSS]	AVFPSIVGR	945.5516414	2	1.8701
	AVFPSIVGR	945.5516414	2	1.528
gi|56474160|gb|EAL51543.1| Rho GTPase activating protein, putative [Entamoeba histolytica HM-1:IMSS]	IKASLILRQKINK	1524.99482	2	1.5327
gi|56474207|gb|EAL51590.1| protein kinase, putative [Entamoeba histolytica HM-1:IMSS]	M*NEMSEIVLKDDK	1567.735333	2	1.7769
	M*NEMSEIVLKDDK	1567.735333	2	1.7455
	M*NEMSEIVLKDDK	1567.735333	2	1.6674
	MNEM*SEIVLKDDK	1567.735333	2	1.9693
gi|56474248|gb|EAL51628.1| Ras family GTPase [Entamoeba histolytica HM-1:IMSS]	QKGAVGFLETSAK	1335.72671	2	1.5662
gi|56474266|gb|EAL51645.1| myosin heavy chain [Entamoeba histolytica HM-1:IMSS]	AAELFGVDAESLR	1377.700887	2	2.408
	AAELFGVDAESLR	1377.700887	2	1.7119
	DLTNELQQTQLK	1430.748554	2	2.3417
	VGILAEIEEVR	1227.694334	2	2.0503
gi|56474362|gb|EAL51735.1| Rab family GTPase [Entamoeba histolytica HM-1:IMSS]	AESEQIPPSQPEK	1439.70129	3	2.2784
	AESEQIPPSQPEK	1439.70129	3	2.1008
gi|7494130|pir||T18292 nicotinamide nucleotide transhydrogenase homolog - Entamoeba histolytica	QGQTLISFFYPAK	1499.789307	2	1.6926
gi|7579069|gb|AAF64243.1| hsp70 BiP [Entamoeba invadens]	QIEQIAQPILAK	1351.79438	2	1.9405
